# HFIP Mediates a Direct C−C Coupling between Michael Acceptors and Eschenmoser's salt

**DOI:** 10.1002/anie.202109933

**Published:** 2022-02-03

**Authors:** Miran Lemmerer, Margaux Riomet, Ricardo Meyrelles, Boris Maryasin, Leticia González, Nuno Maulide

**Affiliations:** ^1^ Institute of Organic Chemistry University of Vienna Währinger Strasse 38 1090 Vienna Austria; ^2^ Institute of Theoretical Chemistry University of Vienna Währinger Strasse 17 1090 Vienna Austria

**Keywords:** Amines, Hydrogen bonds, Iodine, Quantum chemistry, Solvent effects

## Abstract

A direct C−C coupling process that merges Michael acceptors and Eschenmoser's salt is presented. Although reminiscent of the Morita–Baylis–Hillman reaction, this process requires no Lewis base catalyst. The underlying mechanism was unveiled by a combination of kinetic, isotopic labelling experiments as well as computational investigations, which showcased the critical role of HFIP as a superior mediator for proton‐transfer events as well as the decisive role of the halide counterion.

## Graphical abstract

Michael acceptors efficiently couple with Eschenmoser's salt in hexafluoro‐2‐propanol (HFIP) without the need for additives or catalysts. The role of iodide and the solvent HFIP in this transformation have been studied in detail experimentally and computationally to obtain a full mechanistic picture. The products were further transformed through rearrangements and Michael additions.

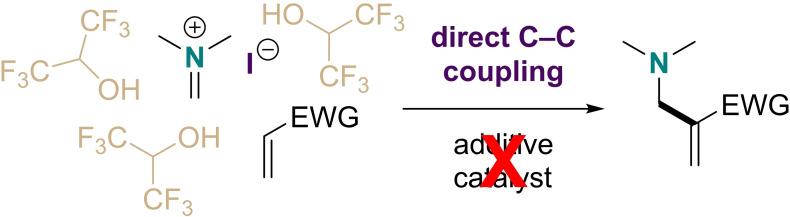

## Introduction

**Scheme 1:**
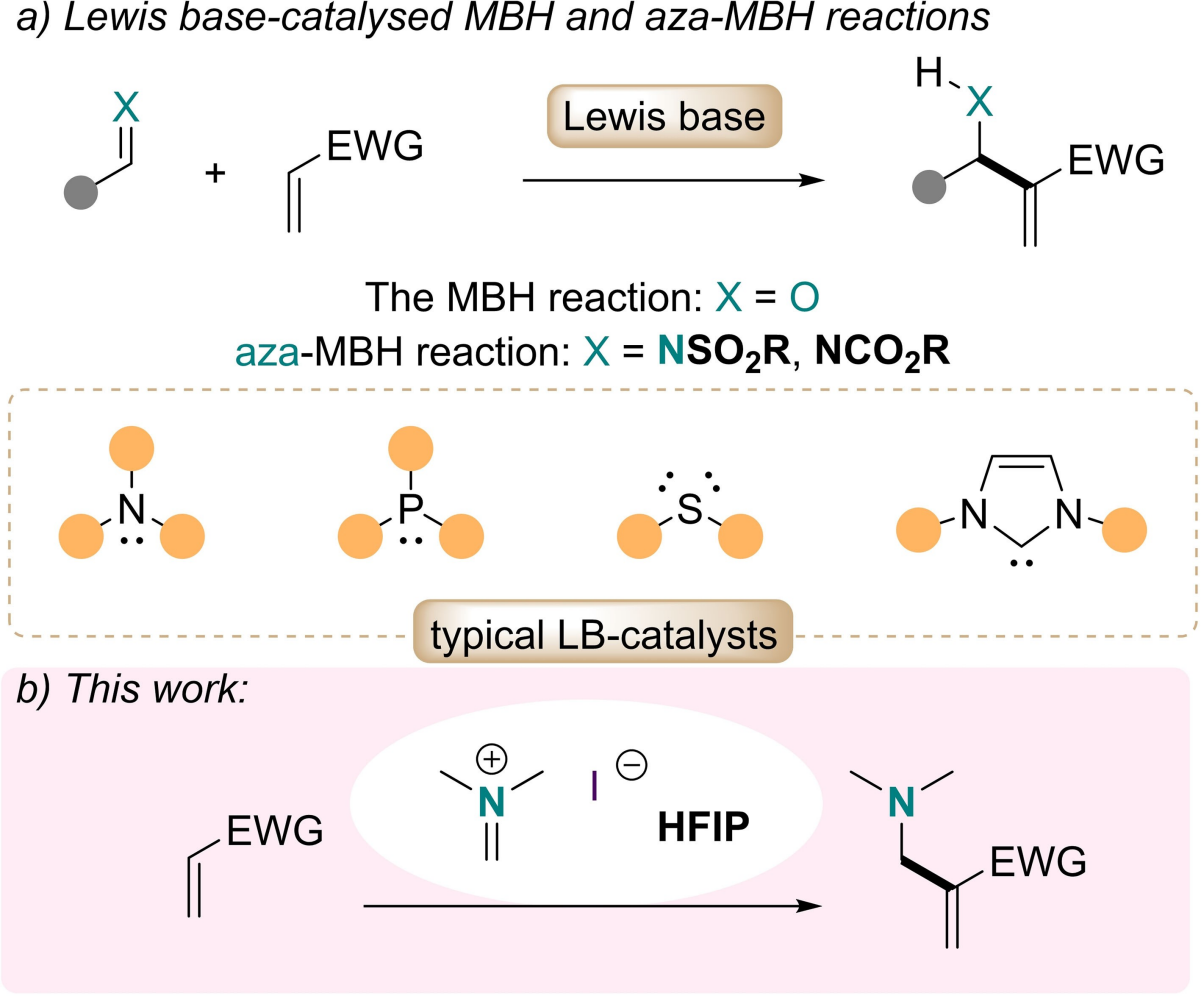
a) The Morita–Baylis–Hillman reaction. b) A catalyst‐free variant relying on Eschenmoser's salt and HFIP.

Since its disclosure in 1968, the Morita–Baylis–Hillman (MBH)^[1]^ reaction has become a privileged tool for C−C bond formation, attracting considerable interest and with numerous variations generated.^[2]^ In this reaction, originally between an aldehyde and a Michael acceptor, a catalytic amount of a Lewis base, such as an amine^[3]^ or a phosphine, is usually deployed (Scheme 1a).[^1^, ^4^, ^5^, ^6^] Less studied is the aza‐variation, which employs imine derivatives as electrophiles and is, therefore, of particular interest for the synthesis of β‐amino carbonyl compounds.^[7]^ Notably, whereas the vast majority of aza‐MBH reactions require the use of imines bearing electron‐withdrawing substituents, the use of *N*‐dialkyl iminium ions is mostly anecdotal, even though the resulting products could be of significant interest to polymer science.^[8]^ In 1992, Koldovski et al. reported the direct aza‐MBH reaction of dimethyl(methylidene)ammonium chloride (also known as Böhme's salt) with methyl vinyl ketone.^[9]^ This reaction was later found to be irreproducible.^[10]^

The recent report of an MBH‐type direct addition of tropylium halides to electron‐poor alkenes sparked our interest in the influence of halide ions in these reactions.^[11]^ Herein, we report an apparently catalyst‐free, HFIP (hexafluoro‐2‐propanol) mediated α‐aminomethylation of Michael acceptors.

## Results and Discussion

Employing homobenzylacrylate **1 a** as a model substrate, we envisaged its direct combination with different iminium ions. In agreement with the observations of Porzelle and Williams,[^9^, ^10^] Böhme's salt was completely unreactive with **1 a** even at elevated temperatures (Table 1, entry 1). Furthermore, no product **2 a** formed with the bromide derivative (instead of the chloride; entry 2). Strikingly, the use of the iodide, commonly known as Eschenmoser's salt, resulted in the formation of **2 a**, with the combination of the iodide counterion and hexafluoro‐2‐propanol (HFIP) as a solvent proving crucial and affording the product in 82 % yield (entries 3–7, see the Supporting Information for full optimisation).^[12]^ The important role of iodide as the counterion was confirmed by the addition of tetrabutylammonium iodide (TBAI) to the reaction with Böhme's salt, which afforded **2 a** in 64 % yield (entry 8).

**Table 1:** Solvent and counterion optimisation using **1 a** as a model substrate.

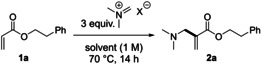			
Entry	X	Solvent	Yield [%]^[a]^
1	Cl	MeCN	0
2	Br	MeCN	0
3	I	MeCN	31
4	I	*i*PrOH	15
5	Cl	HFIP	0
6	Br	HFIP	5
**7**	**I**	**HFIP**	**82**
8	Cl	HFIP^[b]^	64

[a] Yield determined by ^1^H‐ NMR spectroscopy using mesitylene as an internal standard. [b] 4 equiv. tetrabutylammonium iodide were added. HFIP=hexafluoro‐2‐propanol.

**Scheme 2:**
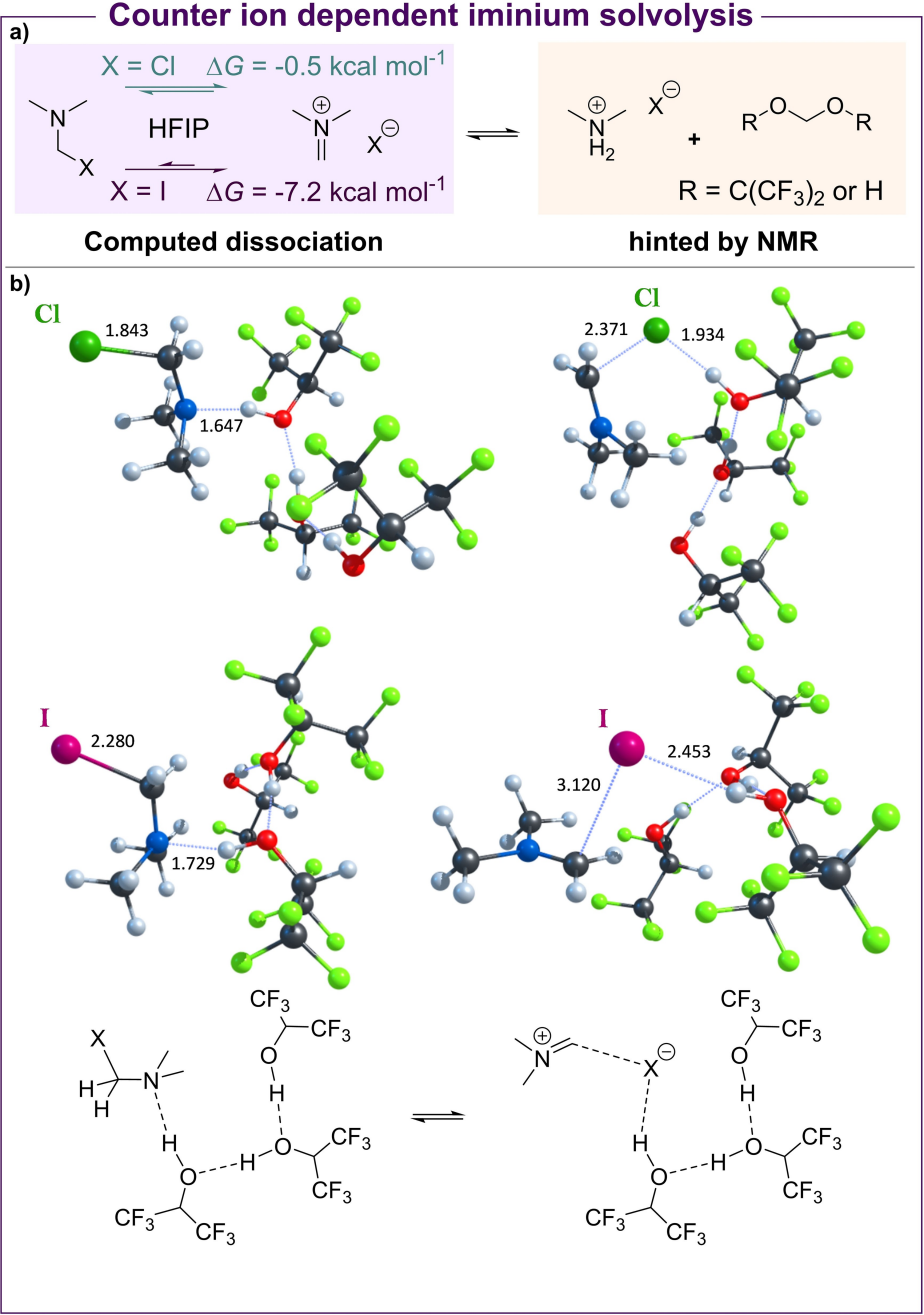
a) Solvolysis of Eschenmoser's and Böhme's salt in HFIP at the optimised concentration and the computed relative Gibbs free energies for the dissociation event. b) DFT‐optimized structures of the (CH_3_)_2_NCH_2_X*3HFIP molecular clusters found through the use of extensive metadynamic sampling.

To elucidate the role of the halide counterion, the interaction of the solvent with Eschenmoser's salt and with Böhme's salt was studied at the DFT level of theory, which indicated a strong effect of HFIP (PBE0‐D3BJ/def2‐TZVP//PBE0‐D3BJ/def2‐SVP (Scheme 2a); see the Supporting Information for computational details). We modelled molecular clusters containing one solute molecule (CH_3_)_2_NCH_2_X and three molecules of solvent HFIP for X=Cl (Böhme's salt) and X=I (Eschenmoser's salt). Scheme 2b presents the most favourable conformations of these clusters for two possible states: X is either covalently bonded (left) or dissociated (right). The calculations indicate a facilitated dissociation of the halide in the presence of HFIP—these results are further supported by related reports,[^13^, ^14^] in which HFIP has been shown to enable rapid halide abstraction to form sulfonium salts.

Although a chain of hydrogen bonds is established between successive HFIP molecules, an additional hydrogen bond can be formed either with the lone pair of electrons on the nitrogen atom of the neutral, covalently bound molecule or with the halide X^−^. In the first case, this intermolecular interaction induces sp^3^ hybridisation of the nitrogen atom. In contrast, in the second case, it increases the halogen–carbon distance (by 0.528 Å and 0.840 Å in Böhme's and Eschenmoser's salts, respectively), leading to a dissociated species. Computed Gibbs free energies suggest that the dissociation of Eschenmoser's salt in HFIP is thermodynamically favourable (Δ*G*=−7.2 kcal mol^−1^). At the same time, this process seems to be highly reversible in the case of Böhme's salt (Δ*G*=−0.5 kcal mol^−1^), thus suggesting that the higher dissociation probability for Eschenmoser's salt is responsible for the experimentally observed halide effect.

Further studies of the salt's behavior were conducted in HFIP‐*d*_2_ using ^1^H– and ^13^C– NMR spectroscopy, which confirmed the prevalence of the dissociated iminium iodide over the α‐iodoamine. Additionally, this analysis revealed the salt to be in equilibrium with its solvolysis product dimethylamine.

**Scheme 3:**
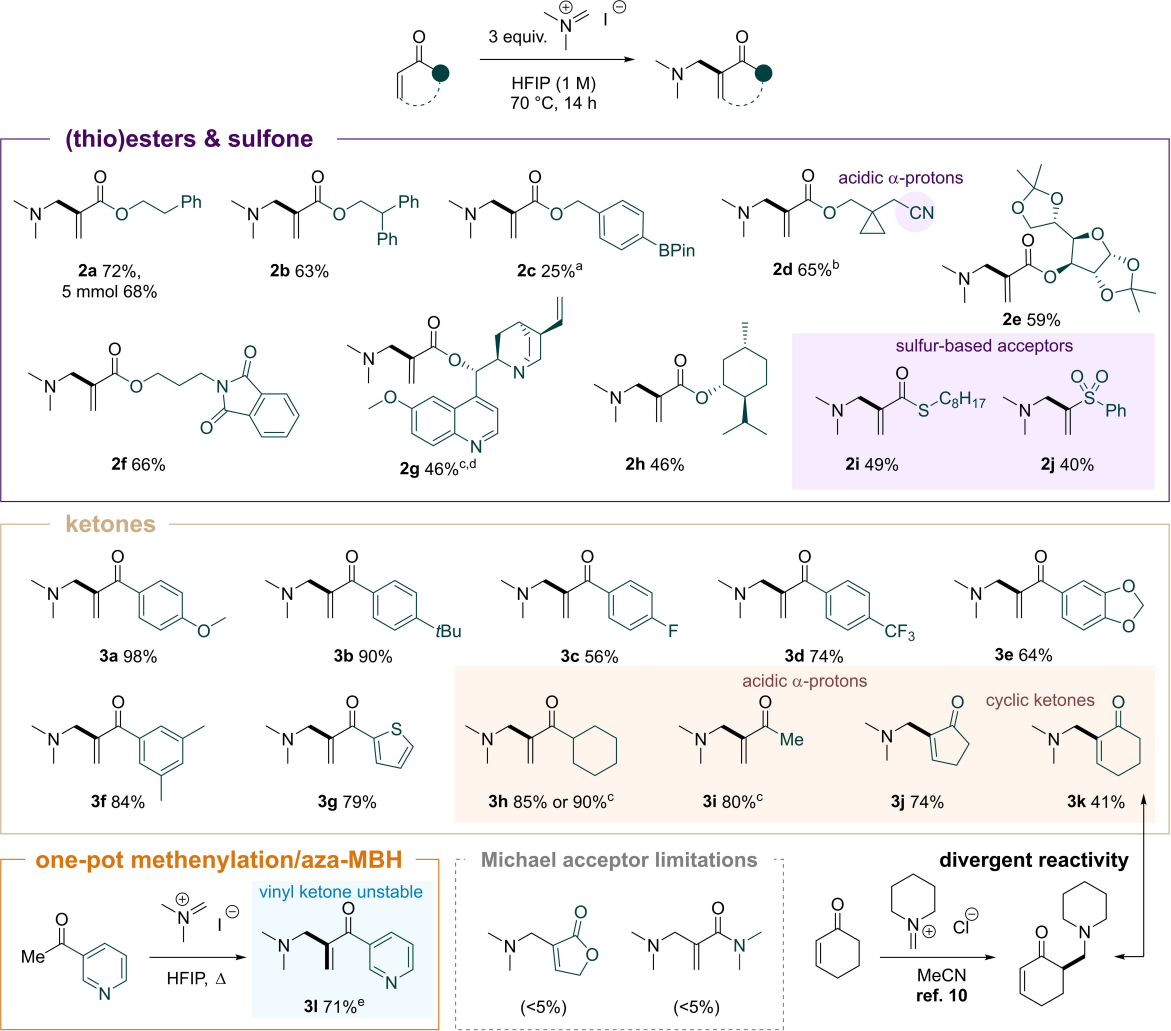
Reaction scope of the MBH‐type coupling of Michael acceptors and Eschenmoser's salt. The reaction was carried out on a 0.2 mmol scale; a) 79 % NMR yield, b) 84 % NMR yield, c) reaction conducted at room temperature, d) 75 % NMR yield, e) 4 equiv Eschenmoser's salt were used.

The high yield and simple C−C bond‐forming reaction encouraged us to investigate the scope of Michael acceptors in the process (Scheme 3). A 25‐fold scale up for **2 a** resulted in a comparable yield of 68 %. We found that bulkier esters were not detrimental to the reaction (**2 b**). Functional groups such as a boronate (**2 c**), a nitrile (**2 d**), and a phthalimide (**2 f**) were well‐tolerated under the reaction conditions. Despite the acidity of HFIP, acetal protecting groups also remained intact (**2 e**). In contrast to most acrylates, a quinine derivative was found to react at room temperature (**2 g**), likely benefiting from additional catalysis enabled by the quinuclidine moiety. A menthol‐derived substrate could also be easily coupled to product **2 h**. Additionally, the scope of the reaction could be expanded to other types of electron‐deficient olefins such as a thioester (**2 i**) and a sulfone (**2 j**). Vinyl ketones also appear to be suitable substrates. The reaction occurred with both electron‐rich (**3 a**, **b**, **e**, **f**) and electron‐deficient (**3 c**, **d**) aryl ketones. A thiophene substituent (**3 g**) showed the applicability of the reaction to heterocycles. Products bearing aliphatic enolisable ketones such as cyclohexyl (**3 h**) and methyl groups (**3 i**) could also be synthesised this way without the interference of the additional reactive α‐position. Substitution at the β‐position was also tolerated, as showcased by products **3 j**/**3 k**, derived from cyclopentenone/cyclohexenone, respectively. A direct methenylation/aza‐MBH domino sequence^[15]^ could be accomplished with ketone **3 l**, the vinyl ketone fragment of which had been previously shown to be prone to polymerisation.^[16]^

The limitations of this approach were met when some other electron‐deficient alkenes were employed, such as a lactone or dimethyl acrylamide (see the Supporting Information for more examples). Bulkier iminium iodide salts based on diethylamine, pyrrolidine, and piperidine showed a different reactivity and led mainly to 1,4‐addition. We ascribe this behaviour to a higher preponderance of solvolysis, which increasingly liberates the parent amine and reduces the amount of available iminium ions (Scheme 2a, see the Supporting Information for details).

**Scheme 4:**
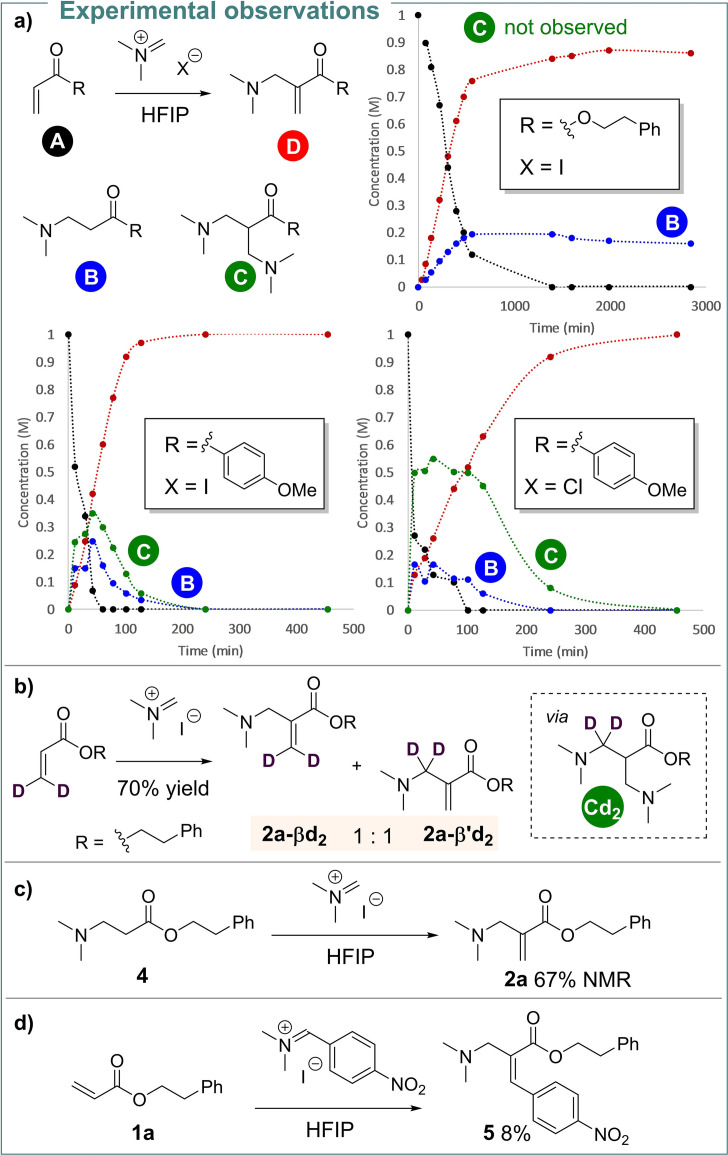
Experimental observations regarding the mechanism.

Kinetic measurements provided insight into the nature of this seemingly uncatalysed process (Scheme 4). Notably, acrylates and vinyl ketones showed significant differences in terms of their kinetic profile. The coupling of acrylate **1 a** displayed a quasi‐linear formation of product **2 a** and much slower formation of the Michael by‐product **B** (Scheme 4a). In contrast, the reaction of vinyl ketone **1 ′a** proved more intricate. For this substrate, fast formation of a similar Michael adduct **B** and an additional intermediate (**C**) was observed, with both compounds disappearing over time and converging into the observed product **2 a**. The reaction between **1 ′a** and Böhme's salt was also studied. Although the formation of **3 a** appears to be much slower (estimated *t*_1/2_ of 100 min) than using Eschenmoser's salt (estimated *t*_1/2_ of 50 min), the rate of formation of **C** is in the same range. This suggests that the counterion plays a significant role in the consumption of this intermediate.

The kinetic picture depicted is not entirely conclusive as to whether acrylates follow the same mechanistic pathway as vinyl ketones, as no intermediate **C** could be observed for acrylates. Therefore, we subjected an isotopically labelled acrylate **1 a**‐***d***_**2**_ to the reaction conditions (Scheme 4b). The symmetrical intermediate **C*d***_**2**_ would lead to a statistical mixture of products **2 a**‐***d***_**2**_, deuterated in the β‐ or β′‐position, while any other distribution would indicate a mechanism without the involvement of **C**. The obtained ratio of 1 : 1 strongly suggests that intermediate **C** is also a viable transient species for acrylates, although shorter‐lived (to the point of evading direct observation) than in the ketone case. This conclusion was further reinforced by the conversion of by‐product **4** into aza‐MBH‐product **2 a** under the standard reaction conditions (Scheme 4c). Additionally, when *p*‐nitrobenzaldiminium iodide was employed, **5** was obtained as the sole aminomethylated product (Scheme 4d). In this case, elimination of dimethylamine from an intermediate such as **C** is the most reasonable pathway to account for the formation of **5**.

**Scheme 5:**
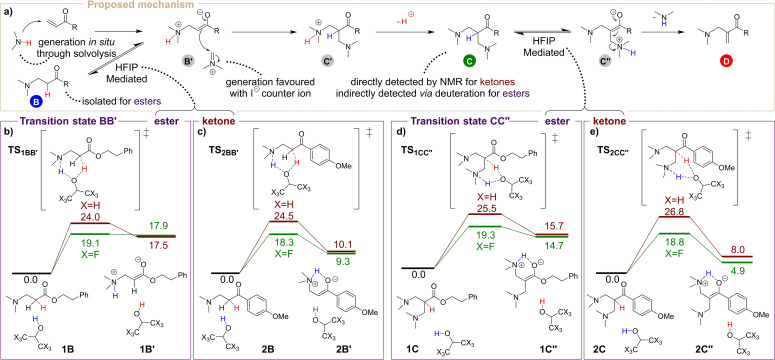
Proposed mechanism (a) and computed relative Gibbs free energy profiles (b)–(e) in the proton‐transfer steps for HFIP (green) and *i*PrOH (brown). The relative free energies are presented in kcal mol^−1^ for each proton‐transfer step individually having the respective reactant complex as a reference (0.0 kcal mol^−1^).

A mechanistic picture for this intriguing, HFIP‐mediated process thus begins to emerge (Scheme 5a). In the first stage, the amine generated *in situ* through solvolysis of the Eschenmoser salt undergoes nucleophilic addition to the Michael acceptor (**A**) to form a zwitterionic intermediate **B′**.^[17]^ This intermediate **B′** can react with the dissociated iminium cation to afford the intermediate **C′**. Deprotonation of the ammonium moiety forms the diamine **C**. This species can be converted into the final product by a proton transfer from the α‐carbon atom to one of the amine groups (species **C′′**),^[18]^ followed by a β‐elimination, which releases dimethylamine and forms the product **D**. The experimentally observed Michael adduct **B** can be reversibly formed from the zwitterionic intermediate **B′** by proton transfer. The experimental results (cf. Table 1) strongly suggest a unique ability of HFIP to mediate the proton‐transfers steps **B′**→**B** and **C**→**C′′**. To better understand this feature, we performed DFT calculations for these steps (see the Supporting Information for computational details).[^19^, ^20^]

The computed relative Gibbs free energy profiles are shown in Scheme 5b–e. These are displayed for proton‐transfer events involving the protic solvent (CX_3_)_2_CHOH, where X=F (green line) or X=H (brown line), and adducts **B/C** leading to the zwitterionic intermediates **B′/C′′**. Two different substituents, R=OC_2_H_4_Ph (ester) and R=C_6_H_4_OMe (ketone), are compared (Scheme 5b,c).

All the obtained transition states describe two concerted proton transfers, in which the protic solvent acts as both a Brønsted acid and base, protonating an amine moiety while simultaneously removing a proton from the α‐carbon atom to the carbonyl group. In this manner, the solvent‐mediated proton transfer allows formation of a six‐membered transition state, in accordance to Aggarwal's proposed mechanism for the MBH reaction.[^18^, ^20a^]

For step **B′**→**B**, the calculated barriers for the solvent‐mediated proton transfer are lower with HFIP than with *i*PrOH for both considered substituents: ΔΔ*G* ^≠^(ester)=4.9 kcal mol^−1^ and ΔΔ*G* ^≠^(ketone)=6.2 kcal mol^−1^, thus showcasing the “hidden” role of HFIP as a catalyst for proton transfer. Although this step is endergonic for both substituents, stabilisation of the zwitterionic intermediate **B′** is critically dependent on the specific substituent R, with Δ*G*_HFIP_(ketone, **2 B**→**2 B′**)=9.3 kcal mol^−1^ and Δ*G*_HFIP_ (ester, **1 B**→**1 B′**)=17.9 kcal mol^−1^.

The same trend can be observed for the conversion of the diamine **C** into the final intermediate **C′′** (Scheme 5d,e). As shown, the ketone moiety stabilises the zwitterionic intermediate **C′′** analogously to the stabilisation of the intermediate **B′**: Δ*G*_HFIP_(ketone, **2 C**→**2 C′′**)=4.9 kcal mol^−1^ and Δ*G*_HFIP_(ester, **1 C**→**1 C′′**)=14.7 kcal mol^−1^. The activation barriers for this concerted proton transfer are again lower with HFIP than *i*PrOH, by 6.2 kcal mol^−1^ and 8.0 kcal mol^−1^ for the ester and ketone, respectively. These results accentuate the importance of HFIP in the reaction conditions as an acidic protic solvent that can easily form and stabilise the key zwitterionic intermediates of the studied transformation.

**Scheme 6:**
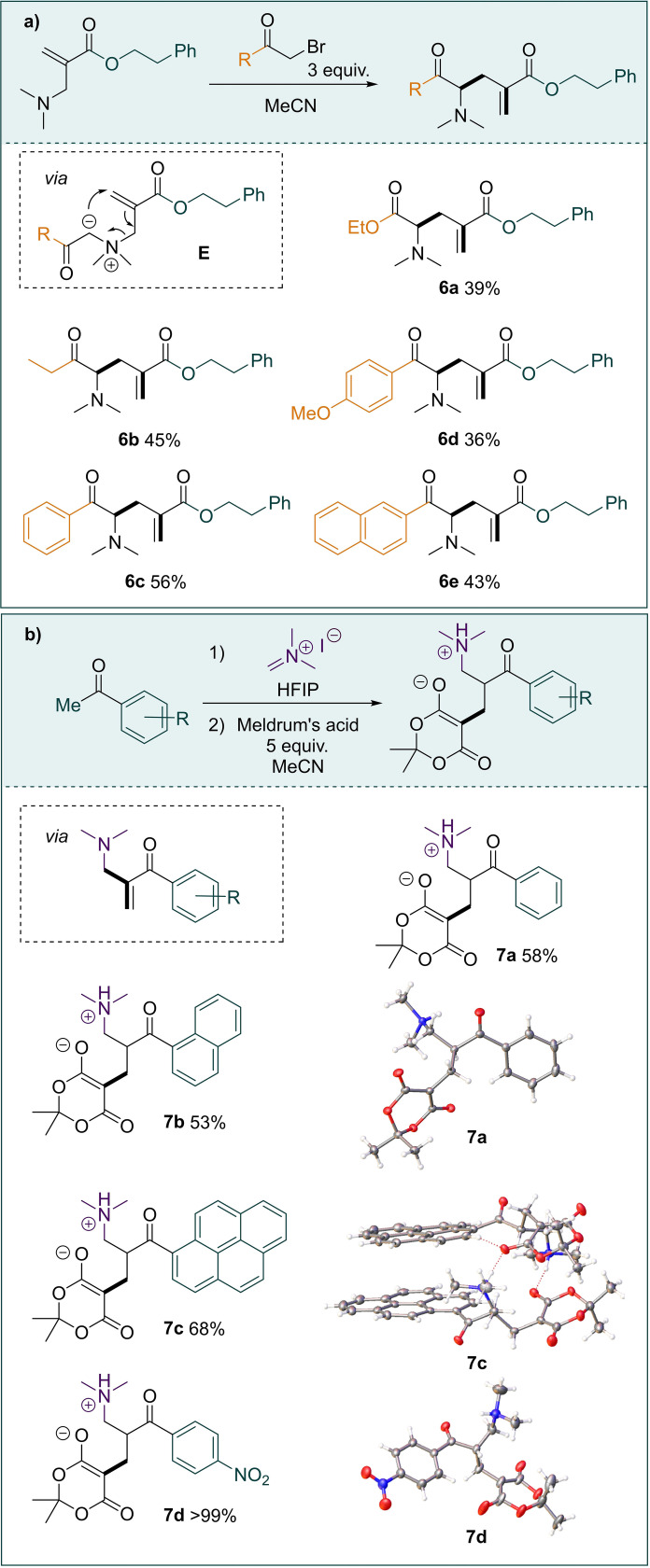
Derivatisation of aza‐MBH products. a) A [2,3]‐Stevens‐type alkylation/rearrangement cascade. b) A direct synthesis of zwitterionic amino acids. 7a_CCDC_2079100; 7c_CCDC_2079101; 7d_CCDC 2079099.

The synthesised aza‐MBH‐adducts are useful compounds for further transformation (Scheme 6). A [2,3]‐Stevens‐type rearrangement could be triggered by nitrogen alkylation with α‐bromo carbonyl compounds,^[21]^ thereby capitalising on the *in situ* formation of a nitrogen ylide **E** (see the Supporting Information for further details). The reaction was compatible with aryl and alkyl ketones as well as esters, readily affording the α‐amino derivatives **6 a**–**e** (Scheme 6a).

The direct domino methenylation/aza‐MBH sequence mentioned above enabled direct capture of these products using Meldrum's acid, thereby affording zwitterionic amino acids that could be isolated in high yields without the need for chromatographic purification (Scheme 6b). Three of these salts (**7 a**, **7 c**, and **7 b**) yielded crystals suitable for X‐ray crystallography, whereby the solid‐state arrangements were dominated by intermolecular hydrogen bonding.

## Conclusion

In conclusion, we have uncovered a C−C coupling process that merges Michael acceptors and Eschenmoser's salt and is reminiscent of the aza‐Morita–Baylis–Hillman reaction.^[22]^ In contrast to common (aza‐)MBH reaction conditions, no additional reagents, in particular the common Lewis base catalysts associated with the process, need to be added. The intrinsic features of the underlying mechanism could be unveiled by a combination of kinetics and isotope labelling experiments as well as computational investigations, which showcased the critical role of HFIP as a superior mediator for proton‐transfer events as well as the decisive role of the halide counterion.

## Conflict of interest

The authors declare no conflict of interest.

## Supporting information

As a service to our authors and readers, this journal provides supporting information supplied by the authors. Such materials are peer reviewed and may be re‐organized for online delivery, but are not copy‐edited or typeset. Technical support issues arising from supporting information (other than missing files) should be addressed to the authors.

Supporting Information

Supporting Information

Supporting Information

Supporting Information

Supporting Information
