# Supplementary material for: HFIP Mediates a Direct C−C Coupling between Michael Acceptors and Eschenmoser's salt
Source: Angew Chem Int Ed Engl. 2022 Feb 3;61(11):e202109933. doi: 10.1002/anie.202109933 (PMC9306631; doi:10.1002/anie.202109933)

Angewandte Chemie
Eine Zeitschrift der Gesellschaft Deutscher Chemiker

Supporting Information

HFIP Mediates a Direct C–C Coupling between Michael Acceptors and Eschenmoser's salt

*M. Lemmerer, M. Riomet, R. Meyrelles, B. Maryasin, L. González, N. Maulide**

Table of Contents

General information 4

Optimisation tables 5

Counterion and solvent optimisation table 5

Equivalent, concentration and temperature optimisation table 6

Impact of water, oxygen or amine additives 6

Optimisation of the Stevens rearrangement 7

Limitations of Michael Acceptors and methyl nucleophiles 7

Limitations of methylene iminium iodides 8

Starting material synthesis 9

General procedure A: esterification 9

General Procedure B: Addition of Grignard reagents to Weinreb amides 9

General Procedure C: Mannich reaction with methyl ketones 10

1a: Phenethyl acrylate 11

1a-d_2: Phenethyl acrylate-3,3-d_2 11

1b: 2,2-Diphenylethyl acrylate 12

1c: 4-(4,4,5,5-Tetramethyl-1,3,2-dioxaborolan-2-yl)benzyl acrylate 12

1d: (1-(cyanomethyl)cyclopropyl)methyl acrylate 13

1e: (3a*R*,5*R*,6*S*,6a*R*)-5-((*S*)-2,2-Dimethyl-1,3-dioxolan-4-yl)-2,2-dimethyltetrahydrofuro[2,3-d][1,3]dioxol-6-yl acrylate 13

1f: 3-(1,3-Dioxoisoindolin-2-yl)propyl acrylate 14

1g: (1*R*)-(6-Methoxyquinolin-4-yl)((1*S*,4*S*,5*R*)-5-vinylquinuclidin-2-yl)methyl acrylate 14

1h: (1*R*,2*S*,5*R*)-2-Isopropyl-5-methylcyclohexyl acrylate 15

1i: *S*-Octyl prop-2-enethioate 15

1’a: 1-(4-Methoxyphenyl)prop-2-en-1-one 16

1’b: 1-(4-(*tert*-Butyl)phenyl)prop-2-en-1-one 16

1’c: 1-(4-Fluorophenyl)prop-2-en-1-one 17

1’d: 1-(4-(Trifluoromethyl)phenyl)prop-2-en-1-one 17

1’e: 1-(Benzo[d][1,3]dioxol-5-yl)prop-2-en-1-one 18

1’f: 1-(3,5-Dimethylphenyl)prop-2-en-1-one 18

1’g: 1-(Thiophen-2-yl)prop-2-en-1-one 19

1’h: 1-Cyclohexylprop-2-en-1-one 19

Characterisation of C–C Coupling Products 20

General procedure D: aza-MBH reaction 20

2a: Phenethyl 2-((dimethylamino)methyl)acrylate 20

2a-d_2: Phenethyl 2-((dimethylamino)methyl)acrylate-d_2 21

2b: 2,2-Diphenylethyl 2-((dimethylamino)methyl)acrylate 21
2c: 4-(4,4,5,5-Tetramethyl-1,3,2-dioxaborolan-2-yl)benzyl 2-((dimethylamino)methyl)acrylate 22
2d: (1-(Cyanomethyl)cyclopropyl)methyl 2-((dimethylamino)methyl)acrylate 22
2e: (3a*R*,5*R*,6*S*,6a*R*)-5-((*S*)-2,2-Dimethyl-1,3-dioxolan-4-yl)-2,2-dimethyltetrahydrofuro[2,3-d][1,3]dioxol-6-yl 2-((dimethylamino)methyl)acrylate 23
2f: 3-(1,3-Dioxoisoindolin-2-yl)propyl 2-((dimethylamino)methyl)acrylate 23
2g: (*S*)-(6-Methoxyquinolin-4-yl)((1*S*,2*R*,4*S*,5*R*)-5-vinylquinuclidin-2-yl)methyl 2-((dimethylamino)methyl)acrylate 24
2h: (1*R*,2*S*,5*R*)-2-Isopropyl-5-methylcyclohexyl 2-((dimethylamino)methyl)acrylate 24
2i: *S*-Octyl 2-((dimethylamino)methyl)prop-2-enethioate 25
2j: *N*,*N*-dimethyl-2-(phenylsulfonyl)prop-2-en-1-amine 25
3a: 2-((Dimethylamino)methyl)-1-(4-methoxyphenyl)prop-2-en-1-one 26
3b: 1-(4-(*tert*-Butyl)phenyl)-2-((dimethylamino)methyl)prop-2-en-1-one 26
3c: 2-((Dimethylamino)methyl)-1-(4-fluorophenyl)prop-2-en-1-one 27
3d: 2-((Dimethylamino)methyl)-1-(4-(trifluoromethyl)phenyl)prop-2-en-1-one 27
3e: 1-(Benzo[d][1,3]dioxol-5-yl)-2-((dimethylamino)methyl)prop-2-en-1-one 28
3f: 2-((Dimethylamino)methyl)-1-(3,5-dimethylphenyl)prop-2-en-1-one 28
3g: 2-((Dimethylamino)methyl)-1-(thiophen-2-yl)prop-2-en-1-one 29
3h: 1-Cyclohexyl-2-((dimethylamino)methyl)prop-2-en-1-one 29
3i: 3-((Dimethylamino)methyl)but-3-en-2-one 30
3j: 2-((Dimethylamino)methyl)cyclopent-2-en-1-one 30
3k: 2-((Dimethylamino)methyl)cyclohex-2-en-1-one 31
3l: 2-((dimethylamino)methyl)-1-(pyridin-3-yl)prop-2-en-1-one 31
General procedure E: Stevens rearrangement 32
6a: 1-Ethyl 5-phenethyl 2-(dimethylamino)-4-methylenepentanedioate 32
6b: Phenethyl 4-(dimethylamino)-2-methylene-5-oxoheptanoate 33
6c: Phenethyl 4-(dimethylamino)-2-methylene-5-oxo-5-phenylpentanoate 33
6d: Phenethyl 4-(dimethylamino)-5-(4-methoxyphenyl)-2-methylene-5-oxopentanoate 34
6e: Phenethyl 4-(dimethylamino)-2-methylene-5-(naphthalen-2-yl)-5-oxopentanoate 34
General procedure F: 35
7a: 5-(2-((Dimethylammonio)methyl)-3-oxo-3-phenylpropyl)-2,2-dimethyl-4-oxo-4H-1,3-dioxin-6-olate 35
7b: 5-(2-((Dimethylammonio)methyl)-3-(naphthalen-1-yl)-3-oxopropyl)-2,2-dimethyl-4-oxo-4H-1,3-dioxin-6-olate 36
7c: 5-(2-((Dimethylammonio)methyl)-3-oxo-3-(pyren-1-yl)propyl)-2,2-dimethyl-4-oxo-4H-1,3-dioxin-6-olate 36
7d: 5-(2-((dimethylammonio)methyl)-3-(4-nitrophenyl)-3-oxopropyl)-2,2-dimethyl-4-oxo-4H-1,3-dioxin-6-olate 37

Characterisation of by-products 38
4: Phenethyl 2-((dimethylamino)methyl)acrylate 38
S1: Phenethyl 2-(hydroxymethyl)acrylate 38
S2: Phenethyl 2-(acetoxymethyl)acrylate 39
S3: Phenethyl 3-(pyrrolidin-1-yl)propanoate 39
Mechanistic and Kinetic studies 40
Control experiment 40
Kinetics–General 40
Kinetic experiment with 1a with or without workup 41
Kinetic experiment with 1'a with workup with iminium iodide and iminium chloride 42
Kinetic experiment using 4 as starting material 43
Determination of the partial order in Michael acceptor 44
5: Phenethyl (*Z*)-2-((dimethylamino)methyl)-3-(4-nitrophenyl)acrylate 45
Computations 46
Bohme's and Eschenmoser's salts dissociation 47
Formation of enol 47
Solvent interactions 48
X-ray Analysis 50
7a: 5-(2-((Dimethylammonio)methyl)-3-oxo-3-phenylpropyl)-2,2-dimethyl-4-oxo-4H-1,3-dioxin-6-olate 51
7c: 5-(2-((dimethylammonio)methyl)-3-oxo-3-(pyren-1-yl)propyl)-2,2-dimethyl-4-oxo-4H-1,3-dioxin-6-olate 53
7d: 5-(2-((Dimethylammonio)methyl)-3-(4-nitrophenyl)-3-oxopropyl)-2,2-dimethyl-4-oxo-4H-1,3-dioxin-6-olate 56
NMR analysis in deuterated HFIP 58
Böhme's salt spectrum 58
Eschenmoser's salt spectrum 60
NMR spectra 62

General information

Unless otherwise stated, all glassware was flame-dried before use and all reactions were performed under an atmosphere of argon. Hexafluoro-2-propanol (HFIP) was purchased from Fluorochem and used without any particular precaution. All other solvents were distilled from appropriate drying agents prior to use. All reagents were used as received from commercial suppliers unless otherwise stated. Eschenmoser's salt was purchased from TCI, stored in smaller containers when received (ca. 5 g each) and used as such. Reaction progress was monitored by thin layer chromatography (TLC) performed on aluminium plates coated with silica gel F254 with 0.2 mm thickness. Chromatograms were visualised by fluorescence quenching with UV light at 254 nm or by staining using potassium permanganate. Flash column chromatography was performed on an Isolera 4 or Select medium pressure chromatography system (Biotage) using silica gel 60 (230-400 mesh, Merck and co.) or aluminium oxide 90 active neutral (70-230 mesh, Merck and co.). Neat infrared spectra were recorded using a Perkin-Elmer Spectrum 100 FT-IR spectrometer. Wavenumbers (ν_{max}) are reported in cm^{-1}. Mass spectra were obtained using a Bruker maXis UHR-TOF (Qq-TOF) spectrometer, using electrospray ionization (ESI). All ^{1}H NMR and ^{13}C NMR spectra were recorded using a Bruker AV-400, AV-500, AV-600 or AV-700 spectrometer at 300K. Chemical shifts are given in parts per million (ppm, δ), referenced to the solvent peak of $CDCl_3$, defined at δ = 7.26 ppm (^{1}H-NMR) and δ = 77.16 (^{13}C-NMR), or DMSO-d_6, defined at δ = 2.50 ppm (^{1}H-NMR) and δ = 39.52 (^{13}C-NMR). Coupling constants are quoted in Hz (J). ^{1}H NMR splitting patterns are designated as singlet (s), doublet (d), triplet (t), quartet (q) as they appeared in the spectrum. If the appearance of a signal differs from the expected splitting pattern, the observed pattern is designated as apparent (app). Splitting patterns that could not be interpreted or easily visualized are designated as multiplet (m) or broad (br).

Optimisation tables

Counterion and solvent optimisation table

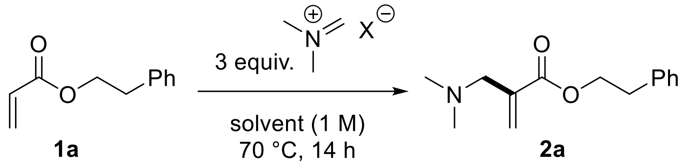

Entry	X	Solvent	Yield[a]
1	Cl	MeCN	0%
2	Br	MeCN	0%
3	I	MeCN	31%
4	I	$MeNO_2$	1%
5	I	EtOH	13%
6	I	*i*PrOH	15%
7	I	*t*BuOH	2%
8	Cl	HFIP	0%
9	Br	HFIP	5%
10	I	HFIP	82%
11	I	HFIP/MeCN (1/1)	67%
12	Cl	HFIP + 4 equiv. TBAI	64%
13	I	HFIP + 0.1 equiv. TFA	46%
14	I	HFIP/DCM (1/1)	82%
15	I	HFIP/DCE (1/1)	81%
16	I	HFIP/toluene (1/1)	82%
17	I	HFIP/DCE (1/10)	42%
18	I	HFIP/toluene (1/10)	38%

Table S1: Solvent and iminium source optimisation. Reactions were carried out on a 0.2 mmol scale.
[a] Yield was measured by ^1H NMR using mesitylene as an internal standard.

Equivalent, concentration and temperature optimisation table

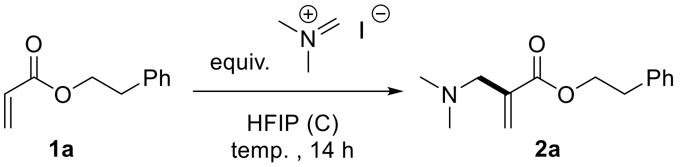

Entry	equiv.	Concentration (M)	temp. (°C)	Yield[a]
1	4	1	70	80%
2	2	1	70	74%
3	1	1	70	29%
4	0.5	1	70	58% (based on ES)
5	3	1.5	70	82%
6	3	0.5	70	28%
7	3	0.2	70	0%
8	3	1	100	47%
9	3	1	40	16%
10	3	1	r.t.	0%

Table S2: Stoichiometry, concentration and temperature optimisation. Reactions were carried out on a 0.2 mmol scale. [a] Yield was measured by ^{1}H NMR using mesitylene as an internal standard.

Impact of water, oxygen or amine additives

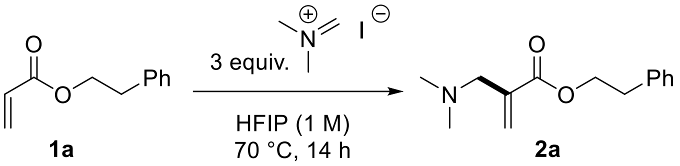

Entry	Deviations from standard set up	Yield[a]
1	5 equiv. H_2O added	42%
2	10 equiv. H_2O added	26%
3	10 mol% H_2O added	75%
4	reaction performed in flame-dried Schlenk tube under Ar atmosphere with degassed HFIP	39%
5	reaction performed under O_2 atmosphere in O_2 sparged HFIP	73%
6	Böhme's salt 3 equiv.; 10 mol% H_2O added	9%
7	Böhme's salt 3 equiv.; 10 mol% Me_2NH added	77%

Table S3: Screening of the reaction robustness and the effect of additives. Reactions were carried out on a 0.2 mmol scale. [a] Yield was measured by ^{1}H NMR using mesitylene as an internal standard.

Optimisation of the Stevens rearrangement

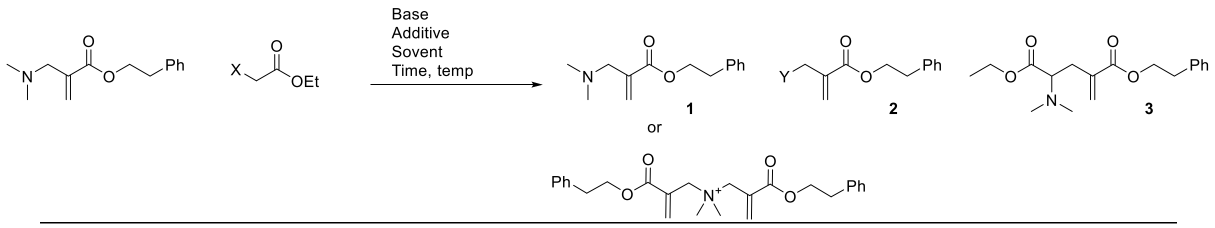

Equiv. Alkylating agent (X=)	Base (equiv.)	Additive (equiv.)	Solvent	Time	Temp. (°C)	Y	1/2/3 ratio in crude
1.5 (Br)	Cs_2CO_3 (2.5)		THF	4h30	rt	Br	1/1/0
1.5 (I)	Cs_2CO_3 (2.5)		THF	4h30	rt		0/1/0
1.5 (Cl)	Cs_2CO_3 (2.5)		THF	16h	rt		1/0/0
1.5 (Br)	Ag_2CO_3 (2.5)		MeCN	20h	60		1.4/1.6/1
3 (Br)	Ag_2CO_3 (3)		MeCN	16h	rt		0/0/1/7(S1)
1.5 (Br)	Ag_2CO_3 (2.5)	MS, 4 Å	DMF	24h	80	OH	1/1.5/0
1.5 (Br)	Ag(OAc) (2.5)		MeCN	16h	60	OAc	1.4/1/0
3 (Br)	Ag_2CO_3 (3)	MS	MeCN	4h	60		1/0/16 39% isol.

Table S4: Optimisation of the Stevens rearrangement reaction. Reactions were carried out on a 0.2 mmol scale.

Limitations of Michael Acceptors and methyl nucleophiles

The following electron poor alkenes and methyl nucleophiles (for tandem methenylation-aza-MBH reaction) did not yield the aminomethylated products under the standard conditions.

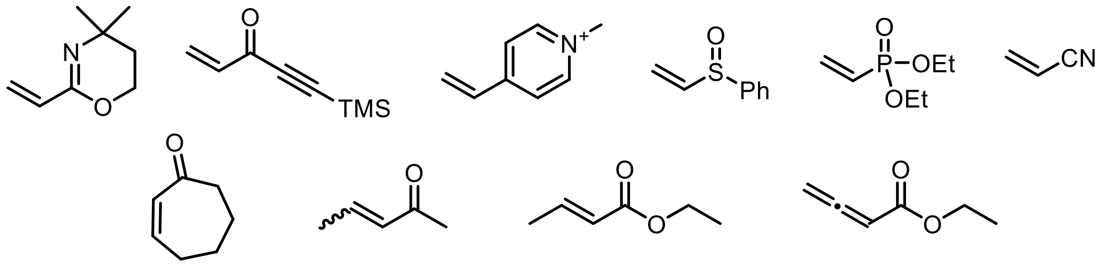

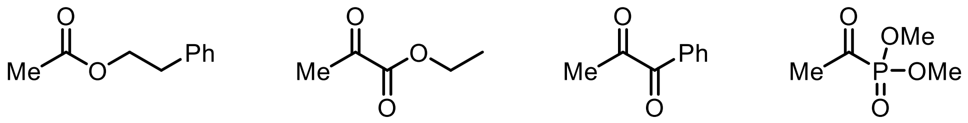

Limitations of methylene iminium iodides

The following salts did not yield the aminomethylated products under the standard conditions.

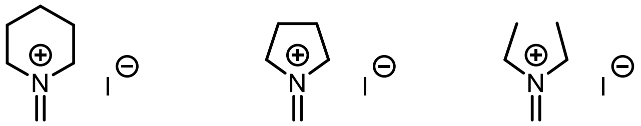

The main observed product resulted from the Michael addition of the corresponding amine. A typical example is shown below.

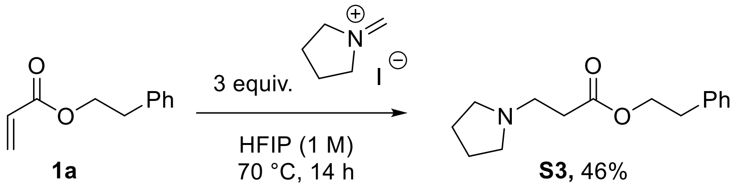

Starting material synthesis

General procedure A: esterification

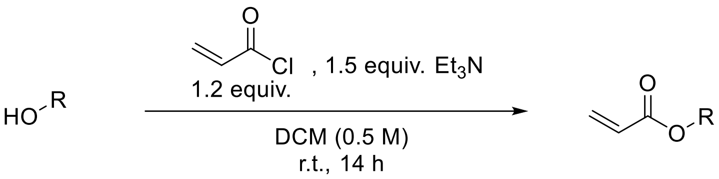

A mixture of alcohol (1.0 equiv.) and Et_3N (1.5 equiv.) in dry CH_2Cl_2 (0.5 M) was cooled to 0 °C in an ice-water bath and acryloyl chloride (1.2 equiv.) was added dropwise. The mixture was warmed to room temperature and stirred for 16 h. The reaction mixture was diluted with Et_2O, transferred to a separation funnel, washed with a saturated aqueous solution of NH_4Cl and then with a saturated aqueous solution of $NaHCO_3$. The combined organic layers were dried over $MgSO_4$, filtered and the solvent was evaporated under reduced pressure. The crude residue was purified by column chromatography (silica, Heptane/EtOAc).

General Procedure B: Addition of Grignard reagents to Weinreb amides

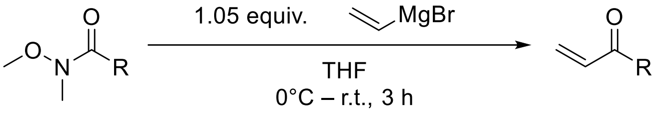

A solution of vinylmagnesium bromide (1 M in THF, 1.05 equiv.) was added over 1 h to a solution of the corresponding Weinreb amide[1] (1.0 equiv.) in dry THF (0.2 M) at 0 °C. The reaction mixture was allowed to warm to room temperature and was stirred for 3 h. The pH of the reaction mixture was adjusted to pH = 2 by the addition of HCl_{aq} (1 M). The phases were separated and the aqueous layer was extracted three times with Et_2O. The combined organic layers were dried over $MgSO_4$, filtered and the solvent was evaporated under reduced pressure. Purification by flash column chromatography (silica, Heptane/EtOAc) afforded the arylvinylketone.

[1] Weinreb amides were synthesized according to a reported procedure: Bauer, A.; Mauro, G. D.; Li, J.; Maulide, N. *Angew. Chem. Int. Ed.* **2020**, *59* (41), 18208–18212.

General Procedure C: Mannich reaction with methyl ketones[2]

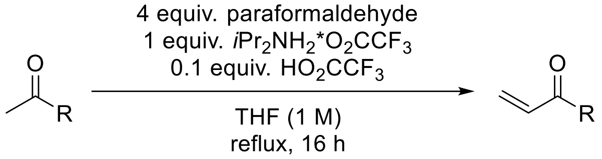

Mannich reactions were performed according to a modified literature procdure.[2] To a mixture of a carbonyl compound (1.0 equiv.) and paraformaldehyde (2.0 equiv.) in dry THF (1 M) was added diisopropylammonium 2,2,2-trifluoroacetate (1.0 equiv.) and trifluoroacetic acid (0.1 equiv.). The reaction mixture was stirred open to the atmosphere at reflux for 2 h. A second addition of paraformaldehyde (2.0 equiv.) was performed and the reaction mixture was stirred at reflux for an additional 16 h open to the atmosphere. The reaction mixture was allowed to cool to room temperature and volatilities were removed under reduced pressure. The crude residue was dissolved in Et_2O and washed with HCl_{aq} (1 M), $NaOH_{aq}$ (1 M), and brine. The combined organic layers were dried over $MgSO_4$, filtered and the solvent was removed under reduced pressure. Purification by flash column chromatography (silica, Heptane/EtOAc) afforded the arylvinylketone.

[2] Bugarin, A.; Jones, K. D.; Connell, B. T. *Chem. Commun.* **2010**, *46* (10), 1715–1717.

1a: Phenethyl acrylate

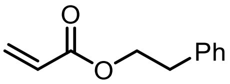

$C_{11}H_{12}O_2$
MW: 176 g mol^{-1}
Yield: 81%
Colourless oil

The compound was prepared using general procedure A from acryloyl chloride (1.95 mL, 24 mmol), homobenzyl alcohol (2.44 g, 20 mmol), Et_3N (4.18 mL, 30 mmol). The product was obtained as a colourless oil (2.85 g, 81%).

Spectral properties were in accordance to those reported in the literature.[3]

^{1}H NMR (700 MHz, $CDCl_3$) δ 7.35–7.27 (m, 2H), 7.26–7.20 (m, 3H), 6.39 (dd, *J* = 17.3, 1.4 Hz, 1H), 6.11 (dd, *J* = 17.3, 10.4 Hz, 1H), 5.82 (dd, *J* = 10.4, 1.4 Hz, 1H), 4.38 (t, *J* = 7.1 Hz, 2H), 2.99 (t, *J* = 7.1 Hz, 2H) ppm.

1a-d_2: Phenethyl acrylate-3,3-d_2

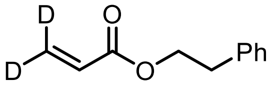

$C_{11}H_{10}D_2O_2$
MW: 178 g mol^{-1}
Yield: 95%
Colourless oil

Phenethyl 2-(triphenyl-λ^5-phosphaneylidene)acetate (637 mg, 1.5 mmol) and paraformaldehyde-d_2 (96 mg, 3.0 mmol) were mixed in an flame-dried Schlenk tube under Ar upon which 1 mL toluene was added. The mixture was stirred at 80 °C for 4 h. The reaction mixture was allowed to cool to room temperature, hexane was added and the resulting slurry was filtered over celite. The solvents were removed under reduced pressure and the product was purified by column chromatography. The product was obtained as a colourless oil (255 mg, 95%).

^{1}H NMR (700 MHz, $CDCl_3$) δ 7.35–7.27 (m, 2H), 7.26–7.20 (m, 3H), 6.09 (bs, 1H), 4.36 (t, *J* = 7.1 Hz, 2H), 2.97 (t, *J* = 7.1 Hz, 2H) ppm.

^{13}C NMR (101 MHz, $CDCl_3$) δ 166.3, 137.9, 129.1 (2C), 128.7 (2C), 128.4, 126.7, 65.2, 35.3 ppm. *CD_2 carbon not observable*

IR (neat) ν_{max}: 3030, 2953, 1719, 1298, 1267, 1173, 1025, 922, 748, 697 cm^{-1}.

HRMS (ESI$^+$): exact mass calculated for $[M+Na]^+$ ($C_{11}H_{10}D_2O_2Na^+$) requires 201.0855, found 201.0860.

[3] R. Zeng, H. Sheng, Y. Zhang, Y. Feng, Z. Chen, J. Wang, M. Chen, M. Zhu, Q. Guo *J. Org. Chem.* **2014**, *19*, 9246–9252.

1b: 2,2-Diphenylethyl acrylate

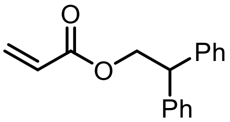

$C_{17}H_{16}O_2$
MW: 252 g mol^{-1}
Yield: 90%
Colourless oil

The compound was prepared using general procedure A from acryloyl chloride (292 µL, 3.6 mmol), 2,2-diphenylethanol (595 mg, 3.0 mmol), Et_3N (627 µL, 4.5 mmol). The product was obtained as a colourless oil (683 mg, 90%).

^{1}H NMR (700 MHz, $CDCl_3$) δ 7.39–7.21 (m, 10H), 6.33 (dd, *J* = 17.3, 1.4 Hz, 1H), 6.06 (dd, *J* = 17.3, 10.4 Hz, 1H), 5.79 (dd, *J* = 10.4, 1.4 Hz, 1H), 4.74 (d, *J* = 7.6 Hz, 2H), 4.44 (t, *J* = 7.6 Hz, 1H) ppm.

^{13}C NMR (101 MHz, $CDCl_3$) δ 166.2, 141.2, 131.0 (2C), 128.7 (4C), 128.5, 128.4 (4C), 127.0 (2C), 66.9, 50.0 ppm.

IR (neat) ν_{max}: 3028, 1720, 1493, 1451, 1405, 1267, 1175, 1063, 982, 696 cm^{-1}.

HRMS (ESI$^+$): exact mass calculated for $[M+Na]^+$ ($C_{17}H_{16}O_2Na^+$) requires 275.1043, found 275.1052.

1c: 4-(4,4,5,5-Tetramethyl-1,3,2-dioxaborolan-2-yl)benzyl acrylate

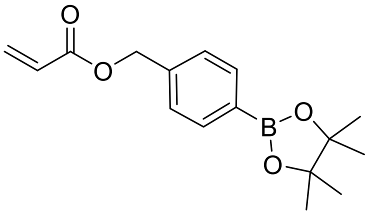

$C_{16}H_{21}BO_4$
MW: 288 g mol^{-1}
Yield: 28%
White solid

The compound was prepared using general procedure A from acryloyl chloride (405 µL, 5.0 mmol), 4-(4,4,5,5-Tetramethyl-1,3,2-dioxaborolan-2-yl)benzyl alcohol (1.00 g, 4.2 mmol), Et_3N (808 µL, 6.2 mmol). The compound was obtained as a white solid (338 mg, 28%).

Spectral properties were in accordance to those reported in the literature.[4]

^{1}H NMR (700 MHz, $CDCl_3$) δ 7.81 (d, *J* = 8.0 Hz, 2H), 7.37 (d, *J* = 8.0 Hz, 2H), 6.45 (dd, *J* = 17.4, 1.3 Hz, 1H), 6.17 (dd, *J* = 17.3, 10.5 Hz, 1H), 5.85 (dd, *J* = 10.5, 1.3 Hz, 1H), 5.21 (s, 2H), 1.34 (s, 12H) ppm.

[4] Y. Yasu, T. Koike, M. Akita *Adv. Synth. Catal.* **2012**, *18*, 3414–3420.

1d: (1-(cyanomethyl)cyclopropyl)methyl acrylate

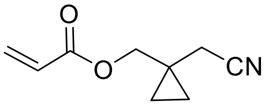

$C_9H_{11}NO_2$
MW: 165 g mol^{-1}
Yield: 88%
Colourless oil

The compound was prepared using general procedure A from acryloyl chloride (487 µL, 6.0 mmol), 2-[1-(hydroxymethyl)cyclopropyl]acetonitrile (585 mg, 5.0 mmol), Et_3N (1.05 mL, 7.5 mmol). The product was obtained as a colourless oil (723 mg, 88%).

^{1}H NMR (400 MHz, $CDCl_3$) δ 6.46 (dd, *J* = 17.3, 1.1 Hz, 1H), 6.16 (dd, *J* = 17.3, 10.4 Hz, 1H), 5.89 (dd, *J* = 10.4, 1.1 Hz, 1H), 4.09 (s, 2H), 2.54 (s, 2H), 0.82–0.67 (m, 4H) ppm.

^{13}C NMR (101 MHz, $CDCl_3$) δ 166.0, 131.6, 128.0, 117.7, 69.4, 23.5, 17.0, 10.7 (2C) ppm.

IR (neat) ν_{max}: 1719, 1409, 1267, 1177, 1054, 982, 809 cm^{-1}.

HRMS (ESI$^+$): exact mass calculated for $[M+Na]^+$ ($C_9H_{11}NO_2Na^+$) requires 188.0682, found 188.0680.

1e: (3a*R*,5*R*,6*S*,6a*R*)-5-((*S*)-2,2-Dimethyl-1,3-dioxolan-4-yl)-2,2-dimethyltetrahydrofuro[2,3-d][1,3]dioxol-6-yl acrylate

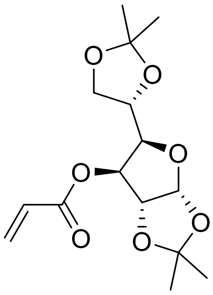

$C_{15}H_{22}O_7$
MW: 314 g mol^{-1}
Yield: 83%
White solid

The compound was prepared using general procedure A from acryloyl chloride (487 µL, 6.0 mmol), Diacetone-D-glucose (1.30 g, 5.0 mmol), Et_3N (1.05 mL, 7.5 mmol). The product was obtained as a white solid (1.31g, 83%).

Spectral properties were in accordance to those reported in the literature.[5]

^{1}H NMR (400 MHz, $CDCl_3$) δ 6.45 (d, *J* = 18.4 Hz, 1H), 6.13 (dd, *J* = 17.3, 10.5 Hz, 1H), 5.97–5.82 (m, 2H), 5.34 (s, 1H), 4.53 (d, *J* = 3.6 Hz, 1H), 4.30–4.21 (m, 2H), 4.13–4.00 (m, 2H), 1.53 (s, 3H), 1.41 (s, 3H), 1.31 (s, 6H) ppm.

[5] P. R. Krishna, V. Kannan, A. Ilangovan, G. V. M. Sharma, *Tetrahedron Asym.* **2001**, *6*, 829–837.

1f: 3-(1,3-Dioxoisoindolin-2-yl)propyl acrylate

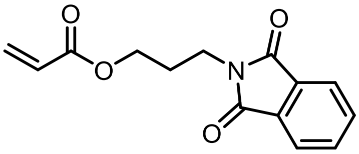

$C_{14}H_{13}NO_4$
MW: 259 g mol^{-1}
Yield: 84%
Colourless solid

To acrylic acid (432 mg, 6.0 mmol) in 3 mL DMF in a flame dried Schlenk under argon were added K_2CO_3 (829 mg, 6.0 mmol), *N*-(3-bromopropyl)phthalimide (804 mg, 3.0 mmol) and potassium iodide (49.8 mg, 0.30 mmol). The mixture was heated to 50 °C for 14 h. Then, after cooling to room temperature, EtOAc was added and the solution was washed first with a saturated aqueous solution of NH_4Cl and then with a saturated aqueous solution of $NaHCO_3$. The organic phase was dried over $MgSO_4$ and the solvent was removed under reduced pressure. Purification by column chromatography on silica (0–40% EtOAc in heptane), afforded the desired compound as a colourless solid (656 mg, 84%).

^{1}H NMR (400 MHz, CDCl$_3$): δ 7.90–7.80 (m, 2H), 7.76–7.67 (m, 2H), 6.42–6.33 (m, 1H), 6.05 (app.ddd, *J* = 17.3, 10.4, 1.3 Hz, 1H), 5.78 (dd, *J* = 10.4, 1.3 Hz, 1H), 4.25–4.18 (m, 2H), 3.87–3.78 (m, 2H), 2.13–2.04 (m, 2H) ppm.

^{13}C NMR (100 MHz, CDCl$_3$): δ 168.4 (2C), 166.2, 134.1 (2C), 132.2, 131.0 (2C), 128.3, 123.4 (2C), 62.1, 35.3, 27.7 ppm.

IR (neat) ν_{max}: 2957, 1772, 1706, 1396, 1272, 1190, 720 cm^{-1}.

HRMS (ESI$^+$): exact mass calculated for $[M+Na]^+$ ($C_{14}H_{13}NNaO_4^+$) requires 282.0737, found 282.0744.

1g: (1*R*)-(6-Methoxyquinolin-4-yl)((1*S*,4*S*,5*R*)-5-vinylquinuclidin-2-yl)methyl acrylate

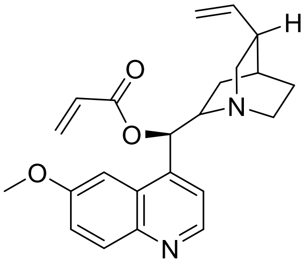

$C_{23}H_{26}N_2O_3$
MW: 378 g mol^{-1}
Yield: 83%
Yellow oil

The compound was prepared using general procedure A from acryloyl chloride (487 µL, 6.0 mmol), quinine (1.62 g, 5.0 mmol), Et_3N (1.05 mL, 7.5 mmol). The product was obtained as a yellow oil (1.57 g, 83%).

^{1}H NMR (400 MHz, CDCl$_3$) δ 8.74 (d, *J* = 4.5 Hz, 1H), 8.01 (d, *J* = 9.2 Hz, 1H), 7.47–7.41 (m, 1H), 7.41–7.33 (m, 2H), 6.56 (d, *J* = 7.1 Hz, 1H), 6.47 (d, *J* = 17.3 Hz, 1H), 6.19 (dd, *J* = 17.3, 10.4 Hz, 1H), 5.90 (d, *J* = 10.6 Hz, 1H), 5.88–5.78 (m, 1H), 5.06–4.97 (m, 2H), 3.96 (s, 3H), 3.49–3.36 (m, 1H), 3.18–3.02 (m, 2H), 2.73–2.58 (m, 2H), 2.34–2.23 (m, 1H), 1.94–1.84 (m, 2H), 1.77–1.67 (m, 1H), 1.63–1.53 (m, 2H) ppm.

^{13}C NMR (101 MHz, CDCl$_3$) δ 165.3, 158.1, 147.6, 144.9, 143.6, 141.9, 132.0, 131.9, 128.2, 127.1, 122.0, 118.9, 114.7, 101.5, 74.1, 59.3, 56.8, 55.8, 42.6, 39.8, 27.9, 27.7, 24.3 ppm.

IR (neat) ν_{max}: 2940, 1725, 1621, 1507, 1260, 1226, 1177, 1030, 851, 807 cm^{-1}.

HRMS (ESI$^+$) (m/z): exact mass calculated for $[M+H]^+$ ($C_{23}H_{27}N_2O_3$) requires 379.2016, found 379.2024.

1h: (1*R*,2*S*,5*R*)-2-Isopropyl-5-methylcyclohexyl acrylate

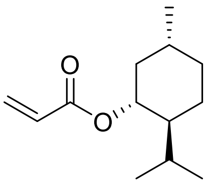

$C_{13}H_{22}O_2$
MW: 210 g mol^{-1}
Yield: 91%
Colourless oil

The compound was prepared using general procedure A from acryloyl chloride (487 µL, 6.0 mmol), (–)-menthol (781 mg, 5.0 mmol), Et_3N (1.05 mL, 7.5 mmol). The product was obtained as a colourless oil (955 mg, 91%).

Spectral properties were in accordance to those reported in the literature.[6]

^{1}H NMR (400 MHz, CDCl$_3$) δ 6.38 (dd, *J* = 17.3, 1.4 Hz, 1H), 6.10 (dd, *J* = 17.3, 10.4 Hz, 1H), 5.79 (dd, *J* = 10.4, 1.4 Hz, 1H), 4.76 (td, *J* = 10.9, 4.4 Hz, 1H), 2.07–1.99 (m, 1H), 1.87 (dtd, *J* = 13.9, 7.0, 2.6 Hz, 1H), 1.73–1.65 (m, 2H), 1.58–1.47 (m, 1H), 1.47–1.37 (m, 1H), 1.15–0.95 (m, 2H), 0.94–0.85 (m, 7H), 0.77 (d, *J* = 7.0 Hz, 3H) ppm.

1i: *S*-Octyl prop-2-enethioate

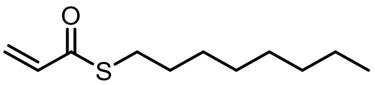

$C_{11}H_{20}OS$
MW: 200 g mol^{-1}
Yield: 16%
Colourless oil

The compound was prepared using general procedure A from acryloyl chloride (292 µL, 3.6 mmol), 1-octanethiol (521 µL, 3.0 mmol), Et_3N (502 µL, 3.6 mmol). The product was obtained as a colourless oil (95.3 mg, 16%).

^{1}H NMR (400 MHz, CDCl$_3$): δ 6.43–6.24 (m, 2H), 5.65 (d, *J* = 9.9 Hz, 1H), 2.96 (t, *J* = 7.3 Hz, 2H), 1.65–1.56 (m, 2H), 1.43–1.20 (m, 10H), 0.88 (t, *J* = 7.0 Hz, 3H) ppm.

^{13}C NMR (100 MHz, CDCl$_3$): δ 190.7, 135.3, 126.1, 31.9, 29.6, 29.3, 29.2, 29.0, 28.9, 22.8, 14.2 ppm.

IR (neat) ν_{max}: 2923, 2853, 1672, 1614, 1463, 1394, 1168, 1007, 975, 947, 727 cm^{-1}.

HRMS (ESI$^+$): exact mass calculated for $[M+Na]^+$ ($C_{11}H_{20}NaOS^+$) requires 223.1127, found 223.1120.

[6] E. Lee-Ruff, F. Xi, J. H. Qie, *J. Org. Chem.* **1996**, *4*, 1547–1550

1'a: 1-(4-Methoxyphenyl)prop-2-en-1-one

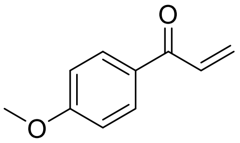

$C_{10}H_{10}O$
MW: 162 g mol^{-1}
Yield: 40%
Colourless solid

The compound was prepared using general procedure C from 4'-methoxyacetophenone (451 mg, 3.0 mmol), diisopropylammonium 2,2,2-trifluoroacetate (646 mg, 3.0 mmol), TFA (22 µL, 0.3 mmol), paraformaldehyde (2 × 180 mg, 2 × 6.0 mmol). The product was obtained as a colourless solid (192 mg, 40%).

Spectral properties were in accordance to those reported in the literature.[7]

^{1}H NMR (600 MHz, CDCl$_3$) δ 8.00–7.94 (m, 2H), 7.17 (dd, *J* = 17.0, 10.5 Hz, 1H), 7.00–6.92 (m, 2H), 6.42 (dd, *J* = 17.0, 1.7 Hz, 1H), 5.87 (dd, *J* = 10.5, 1.7 Hz, 1H), 3.88 (s, 3H).

1'b: 1-(4-(*tert*-Butyl)phenyl)prop-2-en-1-one

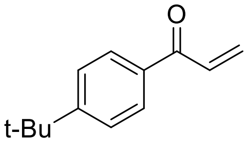

$C_{13}H_{16}O$
MW: 188 g mol^{-1}
Yield: 73%
Colourless oil

The compound was prepared using general procedure B from the corresponding Weinreb amide (314 mg, 1.42 mmol) and vinylmagnesium bromide 1 M in THF (1.49 mL, 1.49 mmol). The product was obtained as a colourless oil (196 mg, 73%).

Spectral properties were in accordance to those reported in the literature.[7]

^{1}H NMR (600 MHz, CDCl$_3$) δ 7.93–7.87 (m, 2H), 7.52–7.48 (m, 2H), 7.17 (dd, *J* = 17.1, 10.6 Hz, 1H), 6.44 (dd, *J* = 17.1, 1.7 Hz, 1H), 5.90 (dd, *J* = 10.6, 1.7 Hz, 1H), 1.35 (s, 9H) ppm.

[7] G. Pandey, J. Vaitla, *Org. Lett.* **2015**, *19*, 4890–4893

1'c: 1-(4-Fluorophenyl)prop-2-en-1-one

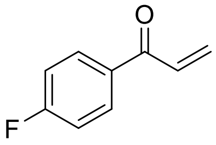

C_9H_7FO
MW: 150 g mol^{-1}
Yield: 41%
Colourless oil

The compound was prepared using general procedure B from the corresponding Weinreb amide (319 mg, 1.74 mmol) and vinylmagnesium bromide 1 M in THF (1.83 mL). The product was obtained as a colourless oil (107 mg, 41%).

Spectral properties were in accordance to those reported in the literature.[8]

^{1}H NMR (500 MHz, $CDCl_3$) δ 8.02–7.94 (m, 2H), 7.19–7.09 (m, 3H), 6.44 (dd, *J* = 17.1, 1.5 Hz, 1H), 5.94 (dd, *J* = 10.6, 1.5 Hz, 1H) ppm.

1'd: 1-(4-(Trifluoromethyl)phenyl)prop-2-en-1-one

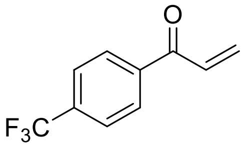

$C_{10}H_7F_3O$
MW: 200 g mol^{-1}
Yield: 27%
Colourless oil

The compound was prepared using general procedure B from the corresponding Weinreb amide (366 mg, 1.57 mmol) and vinylmagnesium bromide 1 M in THF (1.65 mL, 1.65 mmol). The product was obtained as a colourless oil (84 mg, 27%).

Spectral properties were in accordance to those reported in the literature.[8]

^{1}H NMR (500 MHz, $CDCl_3$) δ 8.03 (d, *J* = 8.1 Hz, 2H), 7.75 (d, *J* = 8.2 Hz, 2H), 7.13 (dd, *J* = 17.2, 10.6 Hz, 1H), 6.47 (dd, *J* = 17.2, 1.4 Hz, 1H), 6.02 (dd, *J* = 10.6, 1.4 Hz, 1H) ppm.

[8] Y. Lan, C. Yang, Y. Xu, T. Loh, *Org. Chem. Front.* **2017**, *7,* 1411–1415.

1’e: 1-(Benzo[d][1,3]dioxol-5-yl)prop-2-en-1-one

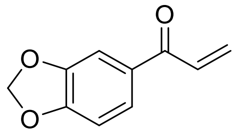

$C_{10}H_8O_3$
MW: 176 g mol^{-1}
Yield: 60%
Colourless oil

The compound was prepared using general procedure B from the corresponding Weinreb amide (316 mg, 1.51 mmol) and vinylmagnesium bromide 1 M in THF (1.59 mL, 2.08 mmol). The product was obtained as a colourless oil (160 mg, 60%).

Spectral properties were in accordance to those reported in the literature.[9]

1’f: 1-(3,5-Dimethylphenyl)prop-2-en-1-one

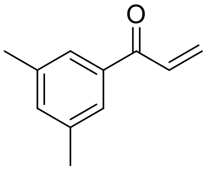

$C_{11}H_{12}O$
MW: 160 g mol^{-1}
Yield: 63%
Colourless oil

The compound was prepared using general procedure B from the corresponding Weinreb amide (383 mg, 1.98 mmol) and vinylmagnesium bromide 1 M in THF (2.08 mL, 2.08 mmol). The product was obtained as a colourless oil (199 mg, 63%).

Spectral properties were in accordance to those reported in the literature.[10]

^{1}H NMR (500 MHz, CDCl$_3$) δ 7.55 (s, 2H), 7.21 (s, 1H), 7.14 (dd, *J* = 17.1, 10.6 Hz, 1H), 6.41 (dd, *J* = 17.1, 1.7 Hz, 1H), 5.90 (dd, *J* = 10.6, 1.7 Hz, 1H), 2.38 (s, 6H) ppm.

[9] M. L. N. Rao, B. S. Ramakrishna, *Eur. J. Org. Chem.* **2019**, *46*, 7545–7554.
[10] A. Claraz, G. Sahoo, D. Berta, A. Madarász, I. Pápai, P. M. Pihko, *Angew. Chem. Int. Ed.* **2016**, *2*, 669–673.

1'g: 1-(Thiophen-2-yl)prop-2-en-1-one

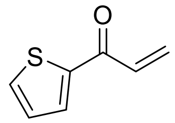

C_7H_6OS
MW: 138 g mol^{-1}
Yield: 33%
Brown oil

The compound was prepared using general procedure B from the corresponding Weinreb amide (256 mg, 1.51 mmol) and vinylmagnesium bromide 1 M in THF (1.59 mL, 1.59 mmol). The product was obtained as a brown oil (68 mg, 33%).

Spectral properties were in accordance to those reported in the literature.[8]

^{1}H NMR (600 MHz, CDCl$_3$) δ 7.79 (dd, *J* = 3.8, 1.1 Hz, 1H), 7.68 (dd, *J* = 4.9, 1.1 Hz, 1H), 7.17 (dd, *J* = 4.9, 3.8 Hz, 1H), 7.08 (dd, *J* = 17.0, 10.4 Hz, 1H), 6.51 (dd, *J* = 17.0, 1.5 Hz, 1H), 5.88 (dd, *J* = 10.4, 1.5 Hz, 1H) ppm.

1'h: 1-Cyclohexylprop-2-en-1-one

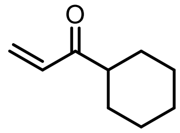

$C_9H_{14}O$
MW: 138 g mol^{-1}
Yield: 24%
Colourless oil

The compound was prepared using general procedure C from 1-cyclohexylethan-1-one (413 µL, 3.0 mmol), diisopropylammonium 2,2,2-trifluoroacetate (646 mg, 3.0 mmol), TFA (22 µL, 0.3 mmol), paraformaldehyde (2 × 180 mg, 2 × 6.0 mmol). The product was obtained as a colourless oil (100 mg, 24%).

Spectral properties were in accordance to those reported in the literature.[7]

^{1}H NMR (400 MHz, CDCl$_3$) δ 6.43 (dd, *J* = 17.5, 10.5 Hz, 1H), 6.25 (d, *J* = 17.5 Hz, 1H), 5.74 (d, *J* = 10.5 Hz, 1H), 2.61 (app.t, *J* = 10.1 Hz, 1H), 1.89 – 1.77 (m, 4H), 1.69 (d, *J* = 12.0 Hz, 1H), 1.41 – 1.20 (m, 5H) ppm.

Characterisation of C–C Coupling Products

General procedure D: aza-MBH reaction

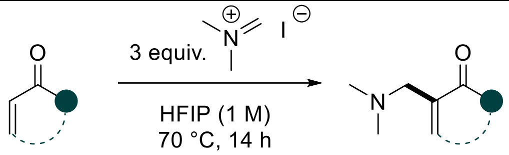

Michael acceptor (0.20 mmol, 1.0 equiv.) was placed in an oven-dried vial charged with a magnetic stir bar. Then, 0.2 mL HFIP (1 M) and Eschenmoser's salt (0.60 mmol, 111 mg, 3.0 equiv.) were added. The vial was capped and placed in an oil bath at 70 °C. The mixture was stirred for 14 h upon which it was allowed to cool to room temperature. CH_2Cl_2 was added and the excess of Eschenmoser's salt was quenched with 5 mL 1 M NaOH (aq.) solution. The phases were separated and the aqueous layer was extracted three times with 10 mL CH_2Cl_2. The combined organic phases were dried over K_2CO_3, filtered and the solvent was removed under reduced pressure. The product was purified *via* LPLC on silica (for esters) or neutral alox (for ketones) using CH_2Cl_2 and DMA (DMA = CH_2Cl_2:MeOH:NH_4OH (aq.25%) 90:10:0.5; typical gradient: from 95/5 to 50/50 CH_2Cl_2/DMA).

2a: Phenethyl 2-((dimethylamino)methyl)acrylate

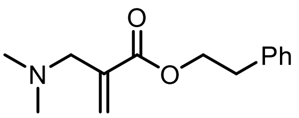

$C_{14}H_{19}NO_2$
MW: 233 g mol^{-1}
Yield: 72%
Colourless oil

The compound was prepared using general procedure D from **1a** (35.2 mg, 0.20 mmol) and Eschenmoser's salt (111 mg, 0.60 mmol). The product was obtained as a colourless oil (33.6 mg, 72%).

Large-scale procedure:
The compound was obtained using general procedure D from **1a** (881 mg, 5.0 mmol) and Eschenmoser's salt (2.78 g, 15 mmol). The product was obtained as a colourless oil (790 mg, 68%).

^{1}H NMR (400 MHz, $CDCl_3$) δ 7.34–7.21 (m, 5H), 6.26–6.24 (m, 1H), 5.73–5.71 (m, 1H), 4.39 (t, *J* = 7.0 Hz, 2H), 3.12 (s, 2H), 3.00 (t, *J* = 7.0 Hz, 2H), 2.24 (s, 6H) ppm.

^{13}C NMR (150 MHz, $CDCl_3$) δ 166.9, 138.1, 137.9, 129.1 (2C), 128.6 (2C), 127.1, 126.7, 65.4, 60.1, 45.5 (2C), 35.3 ppm.

IR (neat) ν_{max}: 2818, 2768, 1714, 1454, 1269, 1178, 1160, 1134, 1032, 699 cm^{-1}.

HRMS (ESI$^+$): exact mass calculated for $[M+H]^+$ ($C_{14}H_{20}NO_2^+$) requires 234.1489, found 234.1489.

2a-d_2: Phenethyl 2-((dimethylamino)methyl)acrylate-d_2

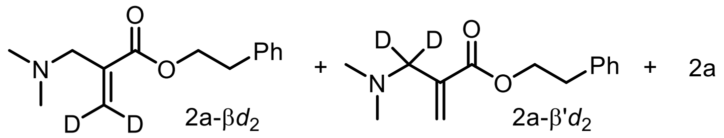

1 : 1 : 0.3

$C_{14}H_{17}D_2NO_2$
MW: 235 g mol^{-1}
Yield: 70%
Colourless oil

The compounds were prepared using general procedure D from **1a-*d*₂** (35.6 mg, 0.20 mmol) and Eschenmoser's salt (111 mg, 0.60 mmol). The isotopomeric mixture was obtained in a ratio of 1:1:0.3 (2a-βd_2:2a-β'd_2:2a) as a colourless oil (33.0 mg, 70%).

^{1}H NMR (400 MHz, CDCl$_3$) δ 7.39–7.15k (m, 5H), 6.25 (s, 0.7H, 2a-β'd_2 + 2a), 5.72 (s, 0.7H, 2a-β'd_2 + 2a), 4.39 (t, *J* = 7.0 Hz, 2H), 3.11 (s, 1.3H 2a-βd_2 + 2a), 3.00 (t, *J* = 7.0 Hz, 2H), 2.23 (s, 6H) ppm.

^{13}C NMR (150 MHz, CDCl$_3$) δ 166.9, 138.1, 137.8 (isotopomer), 129.1 (2C), 128.6 (2C), 127.3, 127.2, 126.7, 65.4, 60.1, 45.5 (2C), 45.4 (2C, isotopomer), 35.2 ppm. *Observed signals of isotopomeric mixture + **2a**.*

IR (neat) ν_{max}: 2943, 2818, 2769, 1714, 1454, 1267, 1177, 1031, 748, 698 cm^{-1}.

HRMS (ESI$^+$): exact mass calculated for $[M+H]^+$ ($C_{14}H_{18}D_2NO_2^+$) requires 236.1614 found 236.1613.

2b: 2,2-Diphenylethyl 2-((dimethylamino)methyl)acrylate

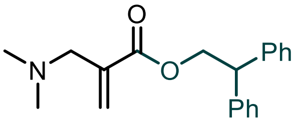

$C_{20}H_{23}NO_2$
MW: 309 g mol^{-1}
Yield: 63%
White solid

The compound was prepared using general procedure D from **1a** (50.5 mg, 0.20 mmol) and Eschenmoser's salt (111 mg, 0.60 mmol). The product was obtained as a white solid (39.2 mg, 63%).

^{1}H NMR (400 MHz, CDCl$_3$) δ 7.33–7.20 (m, 10H), 6.10–6.06 (m, 1H), 5.65–5.61 (m, 1H), 4.71 (d, *J* = 7.6 Hz, 2H), 4.42 (t, *J* = 7.6 Hz, 1H), 3.01 (s, 2H), 2.17 (s, 6H) ppm.

^{13}C NMR (150 MHz, CDCl$_3$) δ 166.8, 141.3 (2C), 137.7, 128.7 (4C), 128.4 (4C), 127.4 (2C), 127.0, 67.1, 60.0, 50.0, 45.4 (2C) ppm.

IR (neat) ν_{max}: 2818, 2769, 1716, 1494, 1452, 1304, 1269, 1177, 1160, 1135, 1033, 698 cm^{-1}.

HRMS (ESI$^+$): exact mass calculated for $[M+H]^+$ ($C_{20}H_{24}NO_2^+$) requires 310.1802, found 310.1807.

2c: 4-(4,4,5,5-Tetramethyl-1,3,2-dioxaborolan-2-yl)benzyl 2-((dimethylamino)methyl)acrylate

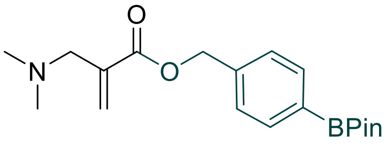

$C_{19}H_{28}BNO_4$
MW: 345 g mol^{-1}
Yield: 25%
White solid

The compound was prepared using general procedure D from **1c** (57.6 mg, 0.20 mmol) and Eschenmoser's salt (111 mg, 0.60 mmol). The product was obtained as a white solid (17 mg, 25%). The isolated yield differs from the measured NMR yield of 79% presumably due to decomposition during purification.

^{1}H NMR (500 MHz, $CDCl_3$) δ 7.81 (d, *J* = 8.1 Hz, 2H), 7.38 (d, *J* = 8.1 Hz, 2H), 6.31 (s, *J* = 0.8 Hz, 1H), 5.75 (d, *J* = 1.4 Hz, 1H), 5.22 (s, 2H), 3.14 (s, 2H), 2.24 (s, 6H), 1.34 (s, 12H) ppm.

^{13}C NMR (126 MHz, $CDCl_3$) δ 166.8, 139.2, 137.7, 135.1 (2C), 127.5, 127.3 (2C), 84.0 (2C), 66.5, 60.2, 45.5 (2C), 25.0 (4C) ppm. *The carbon attached to boron could not be observed due to quadrupolar relaxation*.

IR (neat) v_{max}: 2977, 1720, 1359, 1271, 1143, 1088, 858 cm^{-1}.

HRMS (ESI$^+$): exact mass calculated for $[M+H]^+$ ($C_{19}H_{29}BNO_4^+$) requires 346.2184, found 346.2185.

2d: (1-(Cyanomethyl)cyclopropyl)methyl 2-((dimethylamino)methyl)acrylate

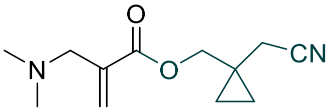

$C_{12}H_{18}N_2O_2$
MW: 222 g mol^{-1}
Yield: 65%
Colourless oil

The compound was prepared using general procedure D from **1d** (33.0 mg, 0.20 mmol) and Eschenmoser's salt (111 mg, 0.60 mmol). The product was obtained as a colourless oil (29.0 mg, 65%).

^{1}H NMR (600 MHz, $CDCl_3$) δ 6.32 (t, *J* = 0.6 Hz, 1H), 5.76 (d, *J* = 1.3 Hz, 1H), 4.09 (s, 2H), 3.13 (s, 2H), 2.55 (s, 2H), 2.24 (s, 6H), 0.78–0.72 (m, 2H), 0.72–0.67 (m, 2H) ppm.

^{13}C NMR (151 MHz, $CDCl_3$) δ 166.7, 137.6, 127.7, 117.8, 69.6, 60.3, 45.5 (2C), 23.6, 17.1, 10.7 (2C).

IR (neat) v_{max}: 1715, 1457, 1303, 1177, 1134, 1028, 989, 854 cm^{-1}.

HRMS(ESI$^+$): exact mass calculated for $[M+H]^+$ ($C_{12}H_{19}N_2O_2^+$) requires 223.1441, found 223.1438.

2e: (3a*R*,5*R*,6*S*,6a*R*)-5-((*S*)-2,2-Dimethyl-1,3-dioxolan-4-yl)-2,2-dimethyltetrahydrofuro[2,3-d][1,3]dioxol-6-yl 2-((dimethylamino)methyl)acrylate

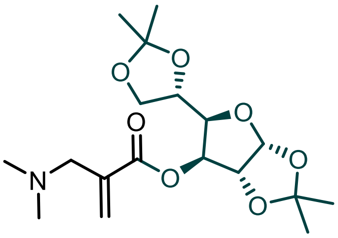

$C_{18}H_{29}NO_7$
MW: 371 g mol^{-1}
Yield: 59%
White solid

The compound was prepared using general procedure D from **1e** (57.6 mg, 0.20 mmol) and Eschenmoser's salt (111 mg, 0.60 mmol). The product was obtained as a white solid (44 mg, 59%).

^{1}H NMR (400 MHz, CDCl$_3$) δ 6.27 (s, 1H), 5.89 (d, *J* = 3.5 Hz, 1H), 5.79 (s, 1H), 5.33 (s, 1H), 4.55 (d, *J* = 3.5 Hz, 1H), 4.31–4.21 (m, 2H), 4.12–4.06 (m, 1H), 4.04–3.99 (m, 1H), 3.18–3.06 (m, 2H), 2.24 (s, 6H), 1.53 (s, 3H), 1.41 (s, 3H), 1.30 (s, 6H) ppm.

^{13}C NMR (151 MHz, CDCl$_3$) δ 165.4, 137.3, 127.8, 112.3, 109.3, 105.1, 83.2, 80.0, 76.4, 72.5, 67.3, 59.8, 45.3 (2C), 26.7, 26.7, 26.2, 25.2 ppm.

IR (neat) ν_{max}: 1725, 1372, 1255, 1213, 1072, 1018, 844 cm^{-1}.

HRMS(ESI$^+$): exact mass calculated for [M+H]$^+$ ($C_{12}H_{19}N_2O_2^+$) requires 372.2017, found 372.2016.

2f: 3-(1,3-Dioxoisoindolin-2-yl)propyl 2-((dimethylamino)methyl)acrylate

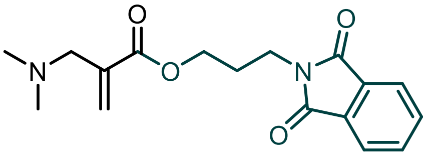

$C_{17}H_{20}N_2O_4$
MW: 316 g mol^{-1}
Yield: 66%
Yellow oil

The compound was prepared using general procedure D from **1f** (51.9 mg, 0.20 mmol) and Eschenmoser's salt (111 mg, 0.60 mmol). The product was obtained as a yellow oil (42.0 mg, 66%).

^{1}H NMR (400 MHz, CDCl$_3$): δ 7.86–7.82 (m, 2H), 7.74–7.69 (m, 2H), 6.29 (s, 1H), 5.77 (s, 1H), 4.26–4.19 (m, 2H), 3.87–3.80 (m, 2H), 3.16 (s, 2H), 2.27 (s, 6H), 2.14–2.05 (m, 2H) ppm.

^{13}C NMR (100 MHz, CDCl$_3$): δ 168.3 (2C), 166.6, 136.9, 134.0 (2C), 132.1 (2C), 128.0, 123.3 (2C), 62.2, 59.6, 45.1 (2C), 35.1, 27.6 ppm.

IR (neat) ν_{max}: 2944, 2819, 2769, 1771, 1704, 1395, 1373, 1178, 1135, 1041, 718 cm^{-1}.

HRMS (ESI$^+$): exact mass calculated for [M+H]$^+$ ($C_{17}H_{21}N_2O_4^+$) requires 317.1496, found 317.1507.

2g: (*S*)-(6-Methoxyquinolin-4-yl)((1*S*,2*R*,4*S*,5*R*)-5-vinylquinuclidin-2-yl)methyl 2-((dimethylamino)methyl)acrylate

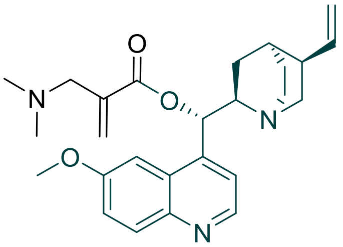

$C_{26}H_{33}N_3O_3$
MW: 435 g mol^{-1}
Yield: 46%
Yellow oil

The compound was prepared using general procedure D at room temperature from **1g** (75.7 mg, 0.20 mmol) and Eschenmoser's salt (111 mg, 0.60 mmol). The product was obtained as a yellow oil (40.1 mg, 46%).

^{1}H NMR (400 MHz, CDCl$_3$) δ 8.72 (d, *J* = 4.5 Hz, 1H), 8.01 (d, *J* = 9.2 Hz, 1H), 7.44–7.34 (m, 3H), 6.54 (d, *J* = 6.7 Hz, 1H), 6.36 (s, 1H), 5.88–5.76 (m, 2H), 4.99 (dd, *J* = 13.7, 8.2 Hz, 2H), 3.96 (s, 3H), 3.43–3.33 (m, 1H), 3.27–3.12 (m, 2H), 3.10–3.00 (m, 2H), 2.73–2.58 (m, 2H), 2.22 (s, *J* = 14.0 Hz, 6H), 1.89–1.80 (m, 2H), 1.78–1.68 (m, 2H), 1.63–1.52 (m, 2H) ppm.

^{13}C NMR (151 MHz, CDCl$_3$) δ 165.9, 158.0, 147.6, 144.9, 144.0, 141.9, 137.9, 131.9, 127.7, 127.1, 122.0, 118.8, 114.6, 101.5, 74.4, 60.2, 59.6, 56.9, 55.8, 45.5 (2C), 42.7, 39.9, 28.0, 27.8, 24.1 ppm.

IR (neat) ν_{max}: 2940, 1725, 1620, 1507, 1404, 1260, 1226, 1177, 1083, 1030, 913, 851, 807 cm^{-1}.

HRMS (ESI$^+$): exact mass calculated for $[M+H^+]^+$ ($C_{26}H_{34}N_3O_3$) requires 436.2595, found 436.2605.

2h: (1*R*,2*S*,5*R*)-2-Isopropyl-5-methylcyclohexyl 2-((dimethylamino)methyl)acrylate

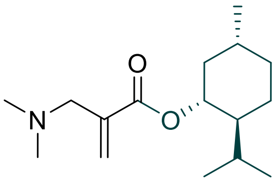

$C_{16}H_{29}NO_2$
MW: 267 g mol^{-1}
Yield: 46%
Colourless oil

The compound was prepared using general procedure D from **1h** (42.1 mg, 0.20 mmol) and Eschenmoser's salt (111 mg, 0.60 mmol). The product was obtained as a colourless oil (24.5 mg, 46%).

^{1}H NMR (600 MHz, CDCl$_3$) δ 6.23 (d, *J* = 0.8 Hz, 1H), 5.71 (d, *J* = 1.4 Hz, 1H), 4.74 (td, *J* = 10.9, 4.4 Hz, 1H), 3.16 (d, *J* = 14.4 Hz, 1H), 3.09 (d, *J* = 14.4 Hz, 1H), 2.24 (s, 6H), 2.06–2.02 (m, 1H), 1.90 (dtd, *J* = 13.9, 7.0, 2.7 Hz, 1H), 1.71–1.65 (m, 2H), 1.54–1.46 (m, 1H), 1.46– 1.40 (m, 1H), 1.12–1.04 (m, 1H), 1.00 (dd, *J* = 23.1, 12.0 Hz, 1H), 0.90 (d, *J* = 5.0 Hz, 3H), 0.89 (d, *J* = 5.4 Hz, 3H), 0.88–0.83 (m, 1H), 0.76 (d, *J* = 7.0 Hz, 3H) ppm.

^{13}C NMR (151 MHz, CDCl$_3$) δ 166.6, 138.3, 126.3, 74.7, 59.9, 47.4, 45.5 (2C), 41.0, 34.4, 31.5, 26.5, 23.7, 22.2, 20.9, 16.6 ppm.

IR (neat) ν_{max}: 2952, 1710, 1455, 1178, 1135, 1036 cm^{-1}.

HRMS (ESI$^+$): exact mass calculated for $[M+H^+]^+$ ($C_{16}H_{30}NO_2$) requires 268.2271, found 268.2264.

2i: *S*-Octyl 2-((dimethylamino)methyl)prop-2-enethioate

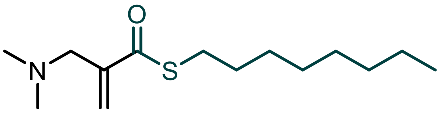

$C_{14}H_{27}NOS$
MW: 257 g mol^{-1}
Yield: 49%
Yellow liquid

The compound was prepared using general procedure D from **1i** (40.1 mg, 0.20 mmol) and Eschenmoser's salt (111 mg, 0.60 mmol). The product was obtained as a yellow liquid (25.2 mg, 49%).

^{1}H NMR (400 MHz, CDCl$_3$): δ 6.19 (s, 1H), 5.69 (s, 1H), 3.17 (s, 2H), 2.92 (t, *J* = 7.4 Hz, 2H), 2.23 (s, 6H), 1.73–1.54 (m, 2H), 1.42–1.20 (m, 10H), 0.92–0.82 (m, 3H) ppm.

^{13}C NMR (100 MHz, CDCl$_3$): δ 193.6, 145.5, 124.5, 60.3, 45.5 (2C), 31.9, 29.5, 29.30, 29.25, 29.1 (2C), 22.8, 14.2 ppm.

IR (neat) ν_{max}: 2924, 2853, 1718, 1662, 1629, 1455, 1265, 971 cm^{-1}.

HRMS (ESI$^+$): exact mass calculated for $[M+H]^+$ ($C_{14}H_{28}NOS^+$) requires 258.1886, found 258.1887.

2j: *N*,*N*-dimethyl-2-(phenylsulfonyl)prop-2-en-1-amine

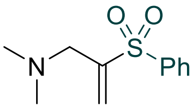

$C_{11}H_{15}NO_2S$
MW: 225 g mol^{-1}
Yield: 40%
Colourless oil

The compound was prepared using general procedure D from (ethenylsulfonyl)-benzene (33.6 mg, 0.20 mmol) and Eschenmoser's salt (111 mg, 0.60 mmol). The product was obtained as a colourless oil (18.1 mg, 40%).

^{1}H NMR (600 MHz, CDCl$_3$) δ 7.89 (d, *J* = 7.6 Hz, 2H), 7.60 (t, *J* = 7.4 Hz, 1H), 7.51 (t, *J* = 7.7 Hz, 2H), 6.48 (s, 1H), 6.03 (s, 1H), 3.08 (s, 2H), 2.04 (s, 6H) ppm.

^{13}C NMR (151 MHz, CDCl$_3$) δ 148.3, 139.6, 133.5, 129.0 (2C), 128.5 (2C), 125.8, 58.2, 45.0 (2C) ppm.

IR (neat) ν_{max}: 1446, 1302, 1141, 1081, 1035, 748, 687, 582 cm^{-1}.

HRMS (ESI$^+$): exact mass calculated for $[M+H]^+$ ($C_{11}H_{17}NO_2S^+$) requires 226.0896, found 226.0893.

3a: 2-((Dimethylamino)methyl)-1-(4-methoxyphenyl)prop-2-en-1-one

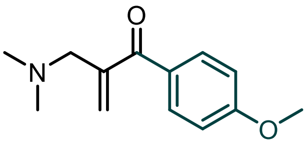

$C_{13}H_{17}NO_2$
MW: 219 g mol^{-1}
Yield: 98%
Pale yellow oil

The compound was prepared using general procedure D from **1'a** (32.4 mg, 0.20 mmol) and Eschenmoser's salt (111 mg, 0.60 mmol). The product was obtained as a pale yellow oil (43.0 mg, 98%).

^{1}H NMR (400 MHz, CDCl$_3$): δ 7.86 (d, *J* = 8.7 Hz, 2H), 6.93 (d, *J* = 8.7 Hz, 2H), 5.89 (s, 1H), 5.64 (s, 1H), 3.87 (s, 3H), 3.31 (s, 2H), 2.28 (s, 6H) ppm.

^{13}C NMR (100 MHz, CDCl$_3$): δ 196.3, 163.4, 145.7, 132.2 (2C), 130.1, 125.0, 113.6 (2C), 61.5, 55.6, 45.7 (2C) ppm.

IR (neat) ν_{max}: 2939, 2817, 2768, 1650, 1596, 1507, 1307, 1253, 1162, 1028, 842, 789, 568 cm^{-1}.

HRMS (ESI$^+$): exact mass calculated for $[M+H]^+$ ($C_{13}H_{18}NO_2^+$) requires 220.1332, found 220.1332.

3b: 1-(4-(*tert*-Butyl)phenyl)-2-((dimethylamino)methyl)prop-2-en-1-one

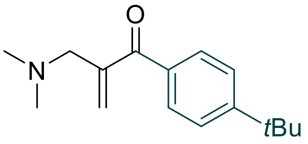

$C_{16}H_{23}NO$
MW: 245 g mol^{-1}
Yield: 90%
Yellowish oil

The compound was prepared using general procedure D from **1'b** (37.7 mg, 0.20 mmol) and Eschenmoser's salt (111 mg, 0.60 mmol). The product was obtained as a yellowish oil (44.4 mg, 90%).

^{1}H NMR (600 MHz, CDCl$_3$) δ 7.81–7.75 (m, 2H), 7.49–7.41 (m, 2H), 5.94 (d, *J* = 1.2 Hz, 1H), 5.71 (s, 1H), 3.32 (s, 2H), 2.28 (s, 6H), 1.33 (s, 9H).

^{13}C NMR (151 MHz, CDCl$_3$) δ 197.2, 156.3, 145.4, 134.7, 129.8 (2C), 126.2, 125.3 (2C), 61.0, 45.7 (2C), 35.2, 31.2 (3C).

IR (neat) ν_{max}: 2962, 1652, 1603, 1268, 1160, 847 cm^{-1}.

HRMS (ESI$^+$): calculated for $[M+H^+]^+$ ($C_{16}H_{24}NO$) requires 246.1852, found 246.1852.

3c: 2-((Dimethylamino)methyl)-1-(4-fluorophenyl)prop-2-en-1-one

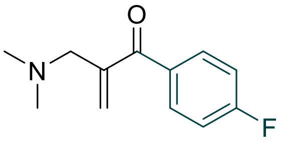

$C_{12}H_{14}FNO$
MW: 207 g mol^{-1}
Yield: 56%
Yellowish oil

The compound was prepared using general procedure D from **1'c** (30 mg, 0.20 mmol) and Eschenmoser's salt (111 mg, 0.60 mmol). The product was obtained as a yellowish oil (23.3 mg, 56%).

^{1}H NMR (600 MHz, CDCl$_3$) δ 7.85 (dd, *J* = 8.8, 5.5 Hz, 2H), 7.12 (app.t, *J* = 8.6 Hz, 2H), 5.96 (d, *J* = 1.1 Hz, 1H), 5.68 (s, 1H), 3.31 (s, 2H), 2.28 (s, 6H) ppm.

^{13}C NMR (151 MHz, CDCl$_3$) δ 196.0, 165.6 (d, *J* = 254.0 Hz), 145.4, 133.7, 132.4 (d, *J* = 9.2 Hz, 2C), 126.4, 115.5 (d, *J* = 21.8 Hz, 2C), 61.1, 45.7 (2C) ppm.

^{19}F NMR (565 MHz, CDCl$_3$) δ -106.11 ppm.

IR (neat) ν_{max}: 1677, 1597, 1505, 1228, 1155, 847 cm^{-1}.

HRMS (ESI$^+$): exact mass calculated for $[M+H]^+$ ($C_{12}H_{15}FNO$) requires 208.1132, found 208.1131.

3d: 2-((Dimethylamino)methyl)-1-(4-(trifluoromethyl)phenyl)prop-2-en-1-one

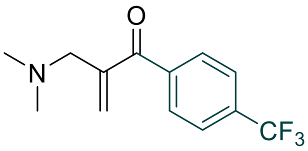

$C_{13}H_{14}F_3NO$
MW: 257 g mol^{-1}
Yield: 74%
Yellowish oil

The compound was prepared using general procedure D from **1'd** (40 mg, 0.20 mmol) and Eschenmoser's salt (111 mg, 0.60 mmol). The product was obtained as a yellowish oil (38.1 mg, 74%).

^{1}H NMR (600 MHz, CDCl$_3$) δ 7.87 (d, *J* = 8.0 Hz, 2H), 7.70 (d, *J* = 8.2 Hz, 2H), 6.06 (s, *J* = 0.9 Hz, 1H), 5.74 (s, 1H), 3.32 (s, 2H), 2.29 (s, 6H) ppm.

^{13}C NMR (151 MHz, CDCl$_3$) δ 196.4, 145.2, 140.7, 133.8 (q, *J* = 32.7 Hz), 129.9 (2C), 128.2, 125.4 (q, *J* = 3.7 Hz, 2C), 123.8 (q, *J* = 272.6 Hz), 60.6, 45.7 (2C) ppm.

^{19}F NMR (377 MHz, CDCl$_3$) δ -63.04 ppm.

IR (neat) ν_{max}: 1669, 1320, 1165, 1124, 1065, 848 cm^{-1}.

HRMS (ESI$^+$): exact mass calculated for $[M+H]^+$ ($C_{13}H_{15}F_3NO$) requires 258.1100, found 258.1106.

3e: 1-(Benzo[d][1,3]dioxol-5-yl)-2-((dimethylamino)methyl)prop-2-en-1-one

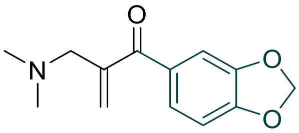

$C_{13}H_{15}NO_3$
MW: 233 g mol^{-1}
Yield: 64%
Yellowish oil

The compound was prepared using general procedure D from **1'e** (35.2 mg, 0.20 mmol) and Eschenmoser's salt (111 mg, 0.60 mmol). The product was obtained as a yellowish oil (30 mg, 64%).

^{1}H NMR (600 MHz, CDCl$_3$) δ 7.44 (d, *J* = 8.1 Hz, 1H), 7.34 (s, 1H), 6.82 (d, *J* = 8.1 Hz, 1H), 6.02 (d, *J* = 0.6 Hz, 2H), 5.86 (s, 1H), 5.62 (s, 1H), 3.29 (s, 2H), 2.26 (s, 6H) ppm.

^{13}C NMR (151 MHz, CDCl$_3$) δ 195.7, 151.7, 148.0, 145.5, 131.8, 126.4, 125.0, 109.6, 107.8, 101.9, 61.5, 45.7 (2C) ppm.

IR (neat) ν_{max}: 1651, 1603, 1486, 1436, 1238, 1096, 1032, 929, 785 cm^{-1}.

HRMS (ESI$^+$): exact mass calculated for [M+H]$^+$ ($C_{13}H_{16}NO_3$) requires 234.1125, found 234.1118.

3f: 2-((Dimethylamino)methyl)-1-(3,5-dimethylphenyl)prop-2-en-1-one

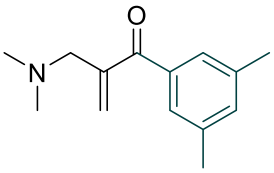

$C_{14}H_{19}NO$
MW: 217 g mol^{-1}
Yield: 84%
Yellowish oil

The compound was prepared using general procedure D from **1'f** (32 mg, 0.20 mmol) and Eschenmoser's salt (111 mg, 0.60 mmol). The product was obtained as a yellowish oil (36.4 mg, 84%).

^{1}H NMR (600 MHz, CDCl$_3$) δ 7.39 (s, 2H), 7.17 (s, 1H), 5.96 (d, *J* = 1.2 Hz, 1H), 5.72 (s, 1H), 3.31 (s, 2H), 2.35 (s, 6H), 2.29 (s,6H) ppm.

^{13}C NMR (151 MHz, CDCl$_3$) δ 198.0, 145.5, 137.9 (2C), 137.6, 134.2, 127.5 (2C), 126.8, 60.8, 45.7 (2C), 21.4 (2C) ppm.

IR (neat) ν_{max}: 1651, 1602, 1454, 1377, 1031, 854, 679 cm^{-1}.

HRMS (ESI+): exact mass calculated for [M+H]$^+$ ($C_{14}H_{20}NO$) requires 218.1539, found 218.1537.

3g: 2-((Dimethylamino)methyl)-1-(thiophen-2-yl)prop-2-en-1-one

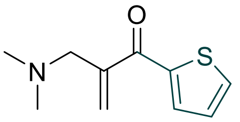

$C_{10}H_{13}NOS$
MW: 195 g mol^{-1}
Yield: 79%
Yellowish oil

The compound was prepared using general procedure D from **1’g** (27.6 mg, 0.20 mmol) and Eschenmoser’s salt (111 mg, 0.60 mmol). The product was obtained as a yellowish oil (31 mg, 79%).

^{1}H NMR (600 MHz, CDCl$_3$) δ 7.69 (dd, *J* = 3.8, 1.1 Hz, 1H), 7.66 (dd, *J* = 4.9, 1.1 Hz, 1H), 7.12 (dd, *J* = 4.9, 3.8 Hz, 1H), 5.87 (s, 1H), 5.86 (d, *J* = 1.1 Hz, 1H), 3.30 (s, 2H), 2.26 (s, 6H) ppm.

^{13}C NMR (151 MHz, CDCl$_3$) δ 189.0, 145.9, 143.7, 134.3, 134.0, 128.0, 124.5, 61.4, 45.6 (2C) ppm.

IR (neat) ν_{max}: 1620, 1513, 410, 1353, 1229, 1032, 848, 720 cm^{-1}.

HRMS (ESI$^+$): exact mass calculated for $[M+H^+]^+$ ($C_{10}H_{14}NOS$) requires 196.0791, found 196.0788.

3h: 1-Cyclohexyl-2-((dimethylamino)methyl)prop-2-en-1-one

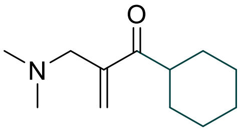

$C_{12}H_{21}NO$
MW: 195 g mol^{-1}
Yield: 85%
Yellowish oil

The compound was prepared using general procedure D from **1’h** (27.6 mg, 0.20 mmol) and Eschenmoser’s salt (111 mg, 0.60 mmol). The product was obtained as a yellowish oil (33.3 mg, 85%). When conducted at r.t., 90% yield was obtained.

^{1}H NMR (600 MHz, CDCl$_3$) δ 6.07 (s, 1H), 5.85 (d, *J* = 1.0 Hz, 1H), 3.10 (s, 2H), 2.99–2.94 (m, 1H), 2.21 (s, 6H), 1.82–1.76 (m, 4H), 1.70–1.67 (m, 1H), 1.43–1.35 (m, 2H), 1.34–1.26 (m, 2H), 1.25–1.18 (m, 1H) ppm.

^{13}C NMR (151 MHz, CDCl$_3$) δ 205.6, 144.8, 125.0, 59.8, 45.7 (2C), 45.6, 29.6 (2C), 26.1, 26.0 (2C) ppm.

IR (neat) ν_{max}: 2929, 1667, 1456, 1258, 1030, 994, 852 cm^{-1}.

HRMS (ESI$^+$): exact mass calculated for $[M+H]^+$ ($C_{12}H_{22}NO$) requires 196.1696, found 196.1701.

3i: 3-((Dimethylamino)methyl)but-3-en-2-one

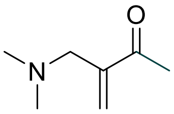

$C_7H_{13}NO$
MW: 127 g mol^{-1}
Yield: 80%
Colourless oil

The compound was prepared using general procedure D at room temperature from methyl vinyl ketone (15.6 mg, 0.20 mmol) and Eschenmoser's salt (111 mg, 0.60 mmol). The product was obtained as a colourless oil (20.3 mg, 80%) without column chromatography.

^{1}H NMR (600 MHz, CDCl$_3$) δ 6.12 (s, 1H), 5.88 (s, 1H), 3.10 (s, 2H), 2.34 (s, 3H), 2.21 (s, 6H) ppm.

^{13}C NMR (151 MHz, CDCl$_3$) δ 199.8, 145.8, 126.6, 59.2, 45.6 (2C), 26.4 ppm.

IR (neat) ν_{max}: 2768, 1708, 1458, 1356, 1098, 849 cm^{-1}.

HRMS (ESI$^+$): exact mass calculated for $[M+H]^+$ ($C_7H_{14}NO$) requires 128.1070, found 128.1070.

3j: 2-((Dimethylamino)methyl)cyclopent-2-en-1-one

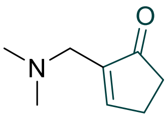

$C_8H_{13}NO$
MW: 139 g mol^{-1}
Yield: 74%
Pale-yellow oil

The compound was prepared using general procedure D from 2-cyclopenten-1-one (16.4 mg, 0.20 mmol) and Eschenmoser's salt (111 mg, 0.60 mmol). The product was obtained as a pale-yellow oil (20.7 mg, 74%).

^{1}H NMR (600 MHz, CDCl$_3$) δ 7.54 (s, 1H), 3.07 (d, *J* = 0.9 Hz, 2H), 2.61 (dd, *J* = 4.5, 2.2 Hz, 2H), 2.44–2.40 (m, 2H), 2.23 (s, 6H) ppm.

^{13}C NMR (151 MHz, CDCl$_3$) δ 209.4, 160.9, 143.0, 53.6, 45.7 (2C), 34.8, 26.8 ppm.

IR (neat) ν_{max}: 1696, 1572, 1454, 1383, 1263, 1175, 1016 cm^{-1}.

HRMS (ESI$^+$): exact mass calculated for $[M+H]^+$ ($C_8H_{14}NO$) requires 140.1070, found 140.1070.

3k: 2-((Dimethylamino)methyl)cyclohex-2-en-1-one

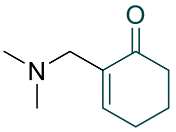

$C_9H_{15}NO$
MW: 153 g mol^{-1}
Yield: 41%
Pale-yellow oil

The compound was prepared using general procedure D from 2-cyclohexen-1-one (20 mg, 0.20 mmol) and Eschenmoser's salt (111 mg, 0.60 mmol). The product was obtained as a pale-yellow oil (12.5 mg, 41%).

^{1}H NMR (600 MHz, CDCl$_3$) δ 6.92 (s, 1H), 3.04 (s, 2H), 2.47–2.37 (m, 4H), 2.21 (s, 6H), 2.05–1.96 (m, 2H) ppm.

^{13}C NMR (151 MHz, CDCl$_3$) δ 199.2, 148.2, 136.3, 57.3, 45.6 (2C), 38.6, 26.2, 23.1 ppm.

IR (neat) ν_{max}: 1669, 1457, 1260, 907, 724 cm^{-1}.

HRMS (ESI$^+$): exact mass calculated for [M+H]$^+$ ($C_9H_{15}NO$) requires 154.1226, found 154.1226.

3l: 2-((dimethylamino)methyl)-1-(pyridin-3-yl)prop-2-en-1-one

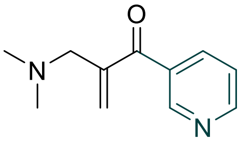

$C_{11}H_{14}N_2O$
MW: 190 g mol^{-1}
Yield: 71%
Pale-yellow oil

The compound was prepared using general procedure D from Ethanone, 1-(3-pyridinyl) (24.2 mg, 0.20 mmol), Eschenmoser's salt (148 mg, 0.80 mmol, 4 equiv.). The product was obtained as a pale-yellow oil (27 mg, 71%).

^{1}H NMR (400 MHz, CDCl$_3$) δ 8.97 (s, 1H), 8.75 (s, 1H), 8.08 (d, *J* = 7.7 Hz, 1H), 7.39 (s, 1H), 6.07 (s, 1H), 5.77 (s, 1H), 3.31 (s, 2H), 2.28 (s, 6H) ppm.

^{13}C NMR (101 MHz, CDCl$_3$) δ 195.8, 153.0, 150.7, 145.3, 136.9, 133.1, 128.1, 123.5, 60.6, 45.7 (2C) ppm.

IR (neat) ν_{max}: 2943, 1658, 1583, 1416, 1026, 990, 704 cm^{-1}.

HRMS (ESI$^+$): calculated for [M+H]$^+$ ($C_{11}H_{14}N_2O$) requires 191.1179, found 191.1182.

General procedure E: Stevens rearrangement

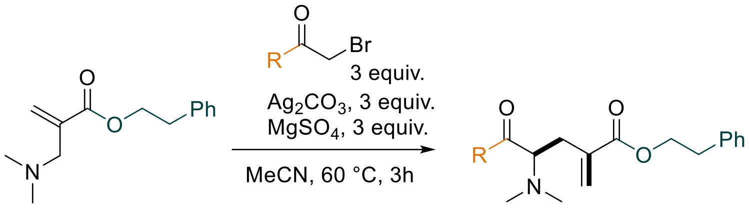

To a flame-dried Schenk tube were added **2a** (0.20 mmol, 1.0 equiv.), Ag_2CO_3 (165 mg, 0.60 mmol, 3.0 equiv.), $MgSO_4$ (72.2 mg, 0.60 mmol, 3.0 equiv.) and MeCN (2 mL, 0.1 M). The mixture was warmed to 60 °C and α-bromo carbonyl (0.60 mmol, 3.0 equiv.) was added. Stirring was continued at 60 °C for 3 h. After cooling to room temperature, the reaction was filtered and washed with EtOAc. The filtrate was extracted with HCl_{aq} (1 M). $NaOH_{aq}$ (1 M) was added to the aqueous layer to reach pH 12. The aqueous layer was extracted three times with CH_2Cl_2. The combined organic layers were dried over K_2CO_3, filtered and the filtrate was concentrated under reduced pressure. The resulting crude material was purified *via* LPLC on silica (for esters) or neutral alox (for ketones) using CH_2Cl_2 and DMA (DMA = CH_2Cl_2:MeOH:NH_4OH (aq.25%) 90:10:0.5; typical gradient: 95/5 to 50/50 CH_2Cl_2/DMA).

6a: 1-Ethyl 5-phenethyl 2-(dimethylamino)-4-methylenepentanedioate

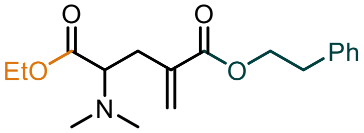

$C_{18}H_{25}NO_4$
MW: 319 g mol^{-1}
Yield: 39%
Colourless oil

The compound was prepared using general procedure E from **2a** (46.7 mg, 0.20 mmol) and ethyl bromoacetate (67 µL, 0.60 mmol).The product was obtained as a colourless oil (24.7 mg, 39%).

^{1}H NMR (600 MHz, CDCl$_3$) δ 7.33–7.28 (m, 2H), 7.24–7.22 (m, 3H), 6.17 (d, *J* = 1.3 Hz, 1H), 5.60 (d, *J* = 1.2 Hz, 1H), 4.41–4.33 (m, 2H), 4.18–4.10 (m, 2H), 3.40 (dd, *J* = 8.3, 7.0 Hz, 1H), 2.99 (t, *J* = 7.0 Hz, 2H), 2.69–2.63 (m, 2H), 2.32 (s, 6H), 1.25 (t, *J* = 7.1 Hz, 3H) ppm.

^{13}C NMR (151 MHz, CDCl$_3$) δ 171.6, 166.8, 138.0, 137.2, 129.1 (2C), 128.7 (2C), 127.6, 126.7, 66.4, 65.4, 60.3, 41.8 (2C), 35.3, 32.1, 14.6 ppm.

IR (neat) ν_{max}: 1716, 1497, 1172, 1031, 698 cm^{-1}.

HRMS (ESI$^+$): exact mass calculated for $[M+H]^+$ ($C_{18}H_{26}NO_4$) requires 320.1856, found 320.1856.

6b: Phenethyl 4-(dimethylamino)-2-methylene-5-oxoheptanoate

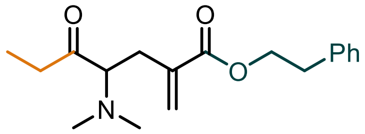

$C_{18}H_{25}NO_3$
MW: 303 g mol^{-1}
Yield: 45%
Colourless oil

The compound was prepared using general procedure E from **2a** (46.7 mg, 0.20 mmol) and 1-bromo-2-butanone (61 µL, 0.60 mmol).The product was obtained as a colourless oil (27.4 mg, 45%).

^{1}H NMR (600 MHz, CDCl$_3$) δ 7.34–7.28 (m, 2H), 7.25–7.21 (m, 3H), 6.15 (d, *J* = 1.0 Hz, 1H), 5.59 (s, 1H), 4.42–4.35 (m, 2H), 3.33 (dd, *J* = 8.9, 4.8 Hz, 1H), 2.99 (t, *J* = 6.9 Hz, 2H), 2.64–2.54 (m, 3H), 2.32 (dq, *J* = 17.8, 7.3 Hz, 1H), 2.27 (s, 6H), 1.00 (t, *J* = 7.3 Hz, 3H) ppm.

^{13}C NMR (151 MHz, CDCl$_3$) δ 211.0, 167.0, 138.0, 137.6, 129.0 (2C), 128.7 (2C), 128.3, 126.7, 71.1, 65.3, 41.9 (2C), 35.2, 35.1, 27.1, 7.8 ppm.

IR (neat) ν_{max}: 3028, 1712, 1497, 1174, 1139, 952, 748, 699 cm^{-1}.

HRMS (ESI$^+$): exact mass calculated for $[M+H]^+$ ($C_{18}H_{26}NO_3$) requires 304.107, found 304.1907.

6c: Phenethyl 4-(dimethylamino)-2-methylene-5-oxo-5-phenylpentanoate

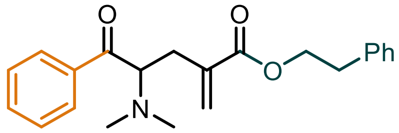

$C_{22}H_{25}NO_3$
MW: 351 g mol^{-1}
Yield: 56%
Colourless oil

The compound was prepared using general procedure E from **2a** (46.7 mg, 0.20 mmol) and phenacyl bromide (119 mg, 0.60 mmol).The product was obtained as a colourless oil (39.0 mg, 56%).

^{1}H NMR (700 MHz, CDCl$_3$) δ 7.96 (d, *J* = 7.3 Hz, 2H), 7.53 (app.t, *J* = 7.4 Hz, 1H), 7.46–7.42 (m, 2H), 7.31–7.27 (m, 2H), 7.24–7.19 (m, 3H), 6.14 (d, *J* = 1.0 Hz, 1H), 5.63 (s, 1H), 4.41–4.32 (m, 3H), 2.97 (t, *J* = 7.0 Hz, 2H), 2.86 (dd, *J* = 13.5, 9.2 Hz, 1H), 2.71 (dd, *J* = 13.5, 4.2 Hz, 1H), 2.34 (s, 6H) ppm.

^{13}C NMR (176 MHz, CDCl$_3$) δ 198.5, 167.0, 137.9, 137.3, 137.3, 133.1, 129.0 (2C), 128.8 (2C), 128.7, 128.65 (4C), 126.7, 65.8, 65.3, 41.4 (2C), 35.2, 26.9 ppm.

IR (neat) ν_{max}: 2937, 1710, 1683, 1448, 1175, 1135, 1043, 948, 749, 696 cm^{-1}.

HRMS (ESI$^+$): exact mass calculated for $[M+H]^+$ ($C_{22}H_{26}NO_3$) requires 352.1907, found 352.1906.

6d: Phenethyl 4-(dimethylamino)-5-(4-methoxyphenyl)-2-methylene-5-oxopentanoate

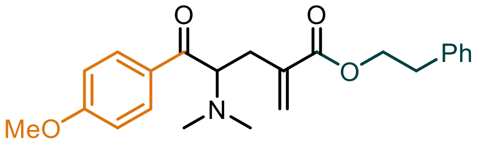

$C_{23}H_{27}NO_4$
MW: 381 g mol^{-1}
Yield: 36%
Orange oil

The compound was prepared using general procedure E from **2a** (46.7 mg, 0.20 mmol) and 2-Bromo-4'-methoxyacetophenone (137 mg, 0.60 mmol).The product was obtained as an orange oil (27.2 mg, 36%).

^{1}H NMR (600 MHz, $CDCl_3$) δ 7.96 (d, *J* = 8.9 Hz, 2H), 7.31–7.27 (m, 2H), 7.23–7.19 (m, 3H), 6.91 (d, *J* = 8.9 Hz, 2H), 6.12 (d, *J* = 1.4 Hz, 1H), 5.61 (d, *J* = 1.0 Hz, 1H), 4.39–4.34 (m, 2H), 4.31 (dd, *J* = 9.2, 4.4 Hz, 1H), 3.86 (s, 3H), 2.97 (t, *J* = 7.0 Hz, 2H), 2.84 (dd, *J* = 13.5, 9.2 Hz, 1H), 2.70 (dd, *J* = 13.5, 4.3 Hz, 1H), 2.33 (s, 6H) ppm.

^{13}C NMR (151 MHz, $CDCl_3$) δ 197.2, 167.1, 163.5, 138.0, 137.4, 131.1 (2C), 130.3, 129.0 (2C), 128.7 (2C), 128.6, 126.7, 113.8 (2C), 65.7, 65.3, 55.6, 41.5 (2C), 35.2, 27.2 ppm.

IR (neat) ν_{max}: 1711, 1672, 1597, 1240, 1166, 1028, 731, 699 cm^{-1}.

HRMS (ESI$^+$): exact mass calculated for $[M+H]^+$ ($C_{23}H_{28}NO_4$) requires 382.2013, found 382.2016.

6e: Phenethyl 4-(dimethylamino)-2-methylene-5-(naphthalen-2-yl)-5-oxopentanoate

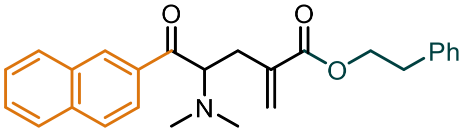

$C_{26}H_{27}NO_3$
MW: 401 g mol^{-1}
Yield: 43%
Colourless oil

The compound was prepared using general procedure E from **2a** (46.7 mg, 0.20 mmol) and 2-bromo-2'-acetonaphthone (153 mg, 0.60 mmol).The product was obtained as a colourless oil (34.7 mg, 43%).

^{1}H NMR (600 MHz, $CDCl_3$) δ 8.54 (s, 1H), 8.02 (dd, *J* = 8.6, 1.4 Hz, 1H), 7.97 (d, *J* = 8.1 Hz, 1H), 7.87 (app.t, *J* = 7.9 Hz, 2H), 7.60 (dd, *J* = 10.9, 4.0 Hz, 1H), 7.55 (t, *J* = 7.5 Hz, 1H), 7.30– 7.27 (m, 2H), 7.22–7.18 (m, 3H), 6.15 (s, 1H), 5.67 (s, 1H), 4.52 (dd, *J* = 9.2, 4.4 Hz, 1H), 4.44–4.34 (m, 2H), 2.96 (t, *J* = 7.0 Hz, 2H), 2.92 (dd, *J* = 13.6, 9.3 Hz, 1H), 2.78 (dd, *J* = 13.5, 4.3 Hz, 1H), 2.40 (s, 6H) ppm.

^{13}C NMR (151 MHz, $CDCl_3$) δ 198.6, 167.1, 137.9, 137.4, 135.7, 134.7, 132.7, 130.4, 129.9, 129.0 (2C), 128.7, 128.6 (2C), 128.5, 128.4, 127.8 (2C), 126.8, 126.7, 124.6, 65.4, 41.5 (2C), 35.2, 27.3 ppm.

IR (neat) ν_{max}: 2936, 1711, 1678, 1466, 1275, 1181, 1137, 817, 749, 699 cm^{-1}.

HRMS (ESI$^+$): exact mass calculated for $[M+H^+]^+$ ($C_{26}H_{28}NO_3$) requires 402.2064, found 402.2064.

General procedure F:

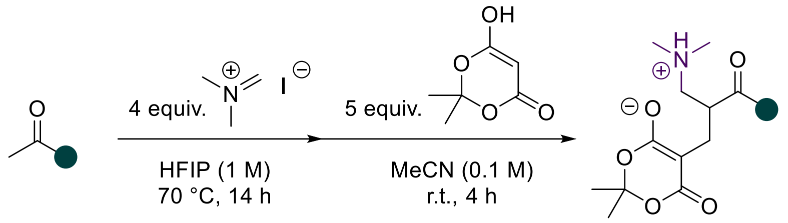

In an oven-dried vial were mixed methyl ketone (1 equiv.) and Eschenmoser's salt (4 equiv.). HFIP (1 M) was added and the mixture was stirred at 70 °C for 14 h upon which it was allowed to cool to room temperature. CH_2Cl_2 was added and the excess of Eschenmoser's salt was quenched with 5 mL $NaOH_{aq}$ (1 M) solution. The phases were separated and the aqueous layer was extracted three times with 10 mL CH_2Cl_2. The combined organic phases were dried over K_2CO_3, filtered and the solvent was removed under reduced pressure. The crude material was dissolved in MeCN (0.1 M) and Meldrum's acid (5 equiv.) was added. The solution was stirred at room temperature for 4 h upon which it was diluted with CH_2Cl_2, H_2O and $NaOH_{aq}$ (0.1 M) until a pH of 7 was reached. The phases were separated and the aq. Phase was extracted with CH_2Cl_2. The combined organic phases were dried over $MgSO_4$, filtered and the solvent was removed under reduced pressure. The crude material was triturated with Et_2O to yield the analytically pure product.

7a: 5-(2-((Dimethylammonio)methyl)-3-oxo-3-phenylpropyl)-2,2-dimethyl-4-oxo-4H-1,3-dioxin-6-olate

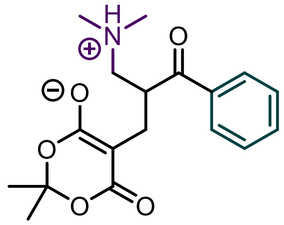

$C_{18}H_{23}NO_5$
MW: 333 g mol^{-1}
Yield: 58%
Orange solid

The compound was prepared using general procedure F from acetophenone (58.3 µL, 0.5 mmol).The product was obtained as an orange solid (96 mg, 58%).

^{1}H NMR (600 MHz, DMSO-d$_6$): δ 10.45 (bs, 1H), 8.15–8.11 (m, 2H), 7.63 (app.t, J = 7.4 Hz, 1H), 7.53 (app.t, J = 7.9 Hz, 2H), 4.15–4.08 (m, 1H), 3.28–3.16 (m, 2H), 2.79 (s, 6H), 1.65 (s, 6H) ppm. *2 H not reported due to overlap with the residual solvent peak at 2.50 ppm.*

^{13}C NMR (150 MHz, DMSO-d$_6$): δ 199.5, 166.3 (2C), 135.8, 133.0, 128.7 (2C), 128.4 (2C), 100.0, 68.0, 56.2, 42.7 (2C), 30.7, 25.7 (2C), 25.3 ppm.

IR (neat) ν_{max}: 1678, 1560, 1351, 1253, 1179, 1122, 946, 783, 703 cm^{-1}.

HRMS (ESI$^+$): exact mass calculated for $[M+H]^+$ ($C_{18}H_{24}NO_5^+$) requires 334.1649, found 334.1656.

7b: 5-(2-((Dimethylammonio)methyl)-3-(naphthalen-1-yl)-3-oxopropyl)-2,2-dimethyl-4-oxo-4H-1,3-dioxin-6-olate

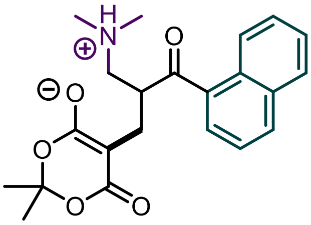

$C_{22}H_{25}NO_5$
MW: 383 g mol^{-1}
Yield: 53%
Orange solid

The compound was prepared using general procedure F from 1-acetonaphthone (85.1 µL, 0.5 mmol).The product was obtained as an orange solid (102 mg, 53%).

^{1}H NMR (400 MHz, DMSO-d$_6$): δ 10.60 (bs, 1H), 8.52–8.45 (m, 1H), 8.43 (d, *J* = 7.2 Hz, 1H), 8.15 (d, *J* = 7.2 Hz, 1H), 8.02–7.96 (m, 1H), 7.65–7.54 (m, 3H), 4.23–4.13 (m, 1H), 2.82 (s, 6H), 1.49 (s, 6H) ppm. *2 H not reported due to overlap with the residual water peak at 3.34 and 2 H not reported due to overlap with the residual solvent peak at 2.50 ppm.*

^{13}C NMR (100 MHz, DMSO-d$_6$): δ 203.2, 166.3 (2C), 134.2, 133.6, 132.8, 130.3, 128.9, 128.2, 127.4, 126.4, 126.2, 124.6, 100.0, 68.3, 56.6, 45.4 (2C), 33.4, 25.6 (2C), 25.0 ppm.

IR (neat) ν_{max}: 1685, 1559, 1482, 1191, 1114, 924, 766, 745 cm^{-1}.

HRMS (ESI$^+$): exact mass calculated for $[M+H]^+$ ($C_{22}H_{26}NO_5^+$) requires 384.1805, found 384.1808.

7c: 5-(2-((Dimethylammonio)methyl)-3-oxo-3-(pyren-1-yl)propyl)-2,2-dimethyl-4-oxo-4H-1,3-dioxin-6-olate

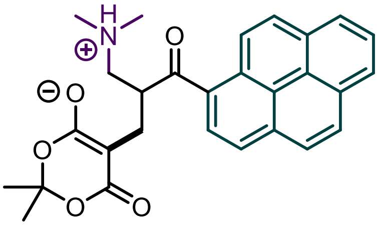

$C_{28}H_{27}NO_5$
MW: 457 g mol^{-1}
Yield: 68%
Yellow solid

The compound was prepared using general procedure F from 1-acetopyrene (89 mg, 0.36 mmol).The product was obtained as a yellow solid (114 mg, 68%).

^{1}H NMR (400 MHz, DMSO-d$_6$): δ 10.66 (bs, 1H), 8.89 (d, *J* = 8.2 Hz, 1H), 8.79 (d, *J* = 9.3 Hz, 1H), 8.43–8.36 (m, 3H), 8.35–8.24 (m, 3H), 8.17–8.12 (m, 1H), 4.40 (bs, 1H), 3.52–3.40 (m, 2H), 2.89 (s, 6H), 2.69–2.56 (m, 2H), 1.50 (s, 6H) ppm.

^{13}C NMR (100 MHz, DMSO-d$_6$): δ 203.6, 166.4 (2C), 133.4, 131.1, 130.6, 130.0, 129.7, 129.5, 128.8, 127.5, 127.2, 126.7, 126.4, 126.0, 125.6, 124.2, 124.1, 123.4, 100.0, 68.3, 56.7, 45.8 (2C), 34.4, 25.6 (2C), 25.0 pm.

IR (neat) ν_{max}: 1714, 1671, 1558, 1414, 1114, 849 cm^{-1}.

HRMS (ESI$^+$): exact mass calculated for $[M+H]^+$ ($C_{28}H_{28}NO_5^+$) requires 458.1962, found 458.1962.

7d: 5-(2-((dimethylammonio)methyl)-3-(4-nitrophenyl)-3-oxopropyl)-2,2-dimethyl-4-oxo-4H-1,3-dioxin-6-olate

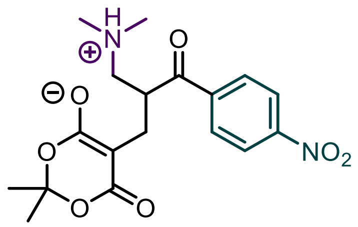

$C_{18}H_{22}N_2O_7$
MW: 378 g mol^{-1}
Yield: >99%
Orange solid

The compound was prepared using general procedure F from *p*-nitroacetophenone (82.6 mg, 0.5 mmol).The product was obtained as an orange solid (191 mg, >99%).

^{1}H NMR (400 MHz, CDCl$_3$): δ 13.30 (bs, 1H), 8.54 (d, *J* = 8.9 Hz, 2H), 8.17 (d, *J* = 8.9 Hz, 2H), 4.20–4.11 (m, 1H), 3.62 (app.dd, *J* = 13.2, 5.4 Hz, 1H), 3.33 (app.dd, *J* = 13.2, 6.5 Hz, 1H), 2.87–2.73 (m, 1H), 2.81 (s, 6H), 2.51 (app.dd, *J* = 15.0, 9.0 Hz, 1H), 1.65 (s, 6H) ppm.

^{13}C NMR (100 MHz, CDCl$_3$): δ 199.0, 168.5 (2C), 150.6, 139.8, 130.6 (2C), 123.9 (2C), 102.8, 70.8, 56.8, 45.4 (2C), 44.1, 25.8, 25.7 (2C) ppm.

IR (neat) ν_{max}: 1676, 1578, 1518, 1384, 1326, 1218, 1109, 973, 700 cm^{-1}.

HRMS (ESI$^+$): exact mass calculated for $[M+H]^+$ ($C_{18}H_{23}N_2O_7^+$) requires 379.1500, found 379.1502.

Characterisation of by-products

4: Phenethyl 2-((dimethylamino)methyl)acrylate

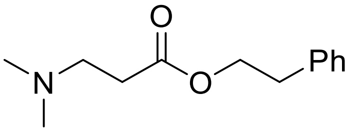

$C_{13}H_{19}NO_2$
MW: 221 g mol^{-1}
Yield: 75%
Colourless oil

The product was prepared using general procedure D from **1a** (352 mg, 2.0 mmol) adding 15 equivalents of H_2O (541 µL). The product was obtained as a colourless oil (330 mg, 75%).

^{1}H NMR (400 MHz, CDCl$_3$) δ 7.34–7.19 (m, 5H), 4.31 (t, *J* = 7.1 Hz, 2H), 2.94 (t, *J* = 7.1 Hz, 2H), 2.58 (t, *J* = 7.0 Hz, 2H), 2.45 (dd, *J* = 10.9, 3.9 Hz, 2H), 2.22 (s, 6H) ppm.

^{13}C NMR (101 MHz, CDCl$_3$) δ 172.5, 137.9, 129.0 (2C), 128.6 (2C), 126.6, 65.0, 54.8, 45.3 (2C), 35.2, 33.0 ppm.

IR (neat) ν_{max}: 1732, 1497, 1166, 1137, 1029, 748, 698 cm^{-1}.

HRMS (ESI$^+$): exact mass calculated for $[M+H^+]^+$ ($C_{15}H_{24}NO_2$) requires 222.1489, found 222.1489.

S1: Phenethyl 2-(hydroxymethyl)acrylate

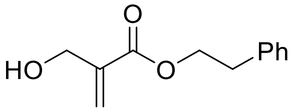

$C_{12}H_{14}O_3$
MW: 206 g mol^{-1}
Yield: 31%
Colourless oil

Obtained during Steven's rearrangement optimisation.

^{1}H NMR (700 MHz, CDCl$_3$) δ 7.33–7.28 (m, 2H), 7.26–7.20 (m, 3H), 6.24 (s, 1H), 5.82 (d, *J* = 1.1 Hz, 1H), 4.41 (t, *J* = 6.9 Hz, 2H), 4.30 (d, *J* = 1.9 Hz, 2H), 3.00 (t, *J* = 6.9 Hz, 2H), 2.18 (brs, 1H) ppm.

^{13}C NMR (176 MHz, CDCl$_3$) δ 166.3, 139.5, 137.8, 129.0 (2C), 128.7 (2C), 126.8, 126.1, 65.5, 62.7, 35.2.

IR (neat) ν_{max}: 3508, 2957, 1712, 1154, 748, 698 ppm cm^{-1}.

HRMS (ESI$^+$): exact mass calculated for $[M+Na]^+$ ($C_{12}H_{14}O_3Na$) requires 229.0835, found 229.0837.

S2: Phenethyl 2-(acetoxymethyl)acrylate

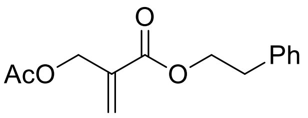

$C_{14}H_{16}O_4$
MW: 248
Yield: 34%
Colourless oil

Obtained during Steven's rearrangement optimisation.

^{1}H NMR (600 MHz, $CDCl_3$) δ 7.35–7.29 (m, 2H), 7.29–7.19 (m, 3H), 6.34 (d, *J* = 0.9 Hz, 1H), 5.83 (dd, *J* = 2.6, 1.4 Hz, 1H), 4.78 (s, 2H), 4.40 (t, *J* = 7.0 Hz, 2H), 2.99 (t, *J* = 7.0 Hz, 2H), 2.08 (s, 3H) ppm.

^{13}C NMR (151 MHz, $CDCl_3$) δ 170.5, 165.2, 137.8, 135.5, 129.1 (2C), 128.7 (2C), 127.8, 126.8, 65.6, 62.6, 35.2, 21.0 ppm.

IR (neat) ν_{max}: 1718, 1225, 1154, 1047, 699 cm^{-1}.

HRMS (ESI$^+$): calculated for $[M+Na]^+$ ($C_{14}H_{16}O_4Na$) requires 271.0941, found 271.0951.

S3: Phenethyl 3-(pyrrolidin-1-yl)propanoate

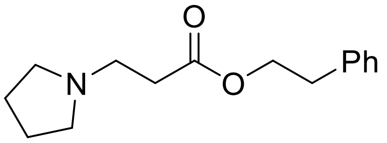

$C_{15}H_{21}NO_2$
MW: 247
Yield: 46%
Colourless oil

The compound was prepared using general procedure D from **1a** (35.26 mg, 0.20 mmol) and 1-methylenepyrrolidin-1-ium iodide[11] (127 mg, 0.60 mmol). The product was obtained as a colourless oil (22.8 mg, 46%).

^{1}H NMR (600 MHz, $CDCl_3$) δ 7.32–7.27 (m, 2H), 7.25–7.19 (m, 3H), 4.30 (t, *J* = 7.0 Hz, 2H), 2.94 (t, *J* = 7.0 Hz, 2H), 2.74 (t, *J* = 7.6 Hz, 2H), 2.56–2.44 (m, 6H), 1.82–1.72 (m, 4H) ppm.

^{13}C NMR (151 MHz, $CDCl_3$) δ 172.6, 138.0, 129.0 (2C), 128.6 (2C), 126.7, 65.0, 54.1 (2C), 51.5, 35.2, 34.3, 23.6 (2C).

IR (neat) ν_{max}: 2958, 2786, 1732, 1497, 1454, 174, 1141, 1051, 748.

HRMS (ESI$^+$): exact mass calculated for $[M+H^+]^+$ ($C_{15}H_{22}NO_2$) requires 248.1645, found 248.1642.

[11] The corresponding iminium was obtained according to a reported procedure: C. Rochin, O. Babot, J. Dunoguès, F. Duboudin, *Synthesis* **1986**, *3*, 228–229.

Mechanistic and Kinetic studies

Control experiment

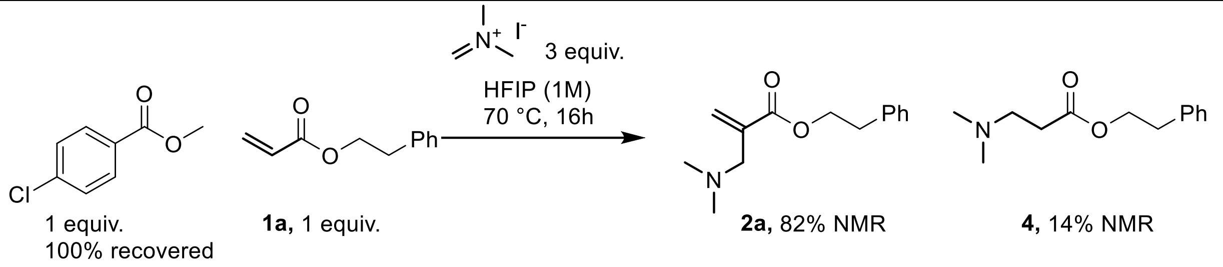

1a (35.2 mg, 0.20 mmol, 1.0 equiv.) and methyl 4-chlorobenzoate (34.1 mg, 0.20 mmol, 1.0 equiv.) were placed in an oven-dried vial charged with a magnetic stir bar. Then, 0.2 mL HFIP (1 M) and Eschenmoser's salt (0.60 mmol, 111 mg, 3.0 equiv.) were added. The vial was capped and placed in an oil bath at 70 °C. The mixture was stirred for 14 h upon which it was allowed to cool to room temperature. CH_2Cl_2 was added and the excess of Eschenmoser's salt was quenched with 5 mL $NaOH_{aq}$ (1 M)solution. The phases were separated and the aqueous layer was extracted three times with 10 mL CH_2Cl_2. The combined organic phases were dried over K_2CO_3, filtered and the solvent was removed under reduced pressure. Mesitylene (27.8 µL, 0.20 mmol, 1.0 equiv.) was added to the crude residue and ^{1}H-NMR was measured. The full recovery of methyl 4-chlorobenzoate and the presence of **2a** in 82% NMR yield validated the use of methyl 4-chlorobenzoate as an inert internal standard in our reaction.

Kinetics–General

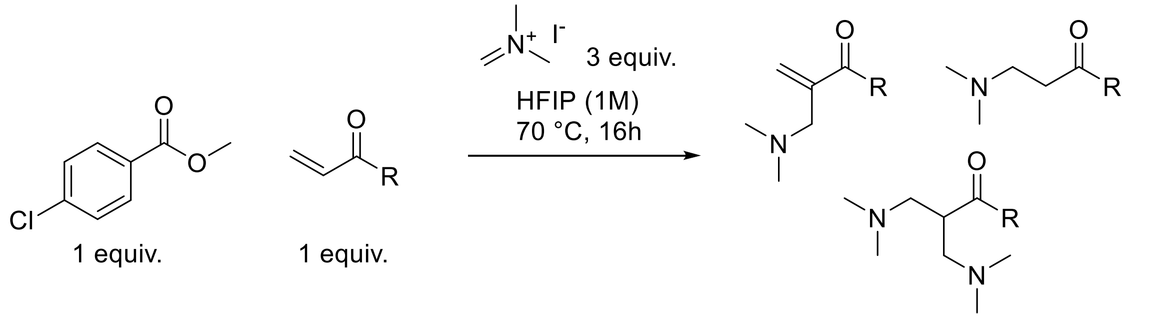

Michael acceptor (1.0 mmol, 1.0 equiv.) and methyl 4-chlorobenzoate (171 mg, 1.0 mmol, 1.0 equiv.) were placed in an oven-dried vial charged with a magnetic stir bar. Then, 1 mL HFIP (1 M) and iminium salt (3.0 mmol, 3.0 equiv.) were added. The vial was capped, placed in an oil bath at 70 °C and the reaction was followed over time. For each measurement, 50–100 µL of the reaction mixture were collected *via* a 1 mL syringe and diluted with 0.5 mL of $CDCl_3$. This mixture was either directly used for ^{1}H-NMR measurement, or added to 1 mL $NaOH_{aq}$ (1 M)solution in a vial. The vial was capped and the heterogeneous mixture was vigorously shaken for ca. 30 s. The organic layer was collected with a syringe and used as such for ^{1}H-NMR measurement.

Kinetic experiment with 1a with or without workup

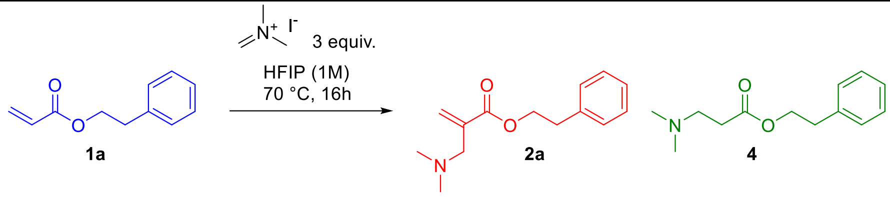

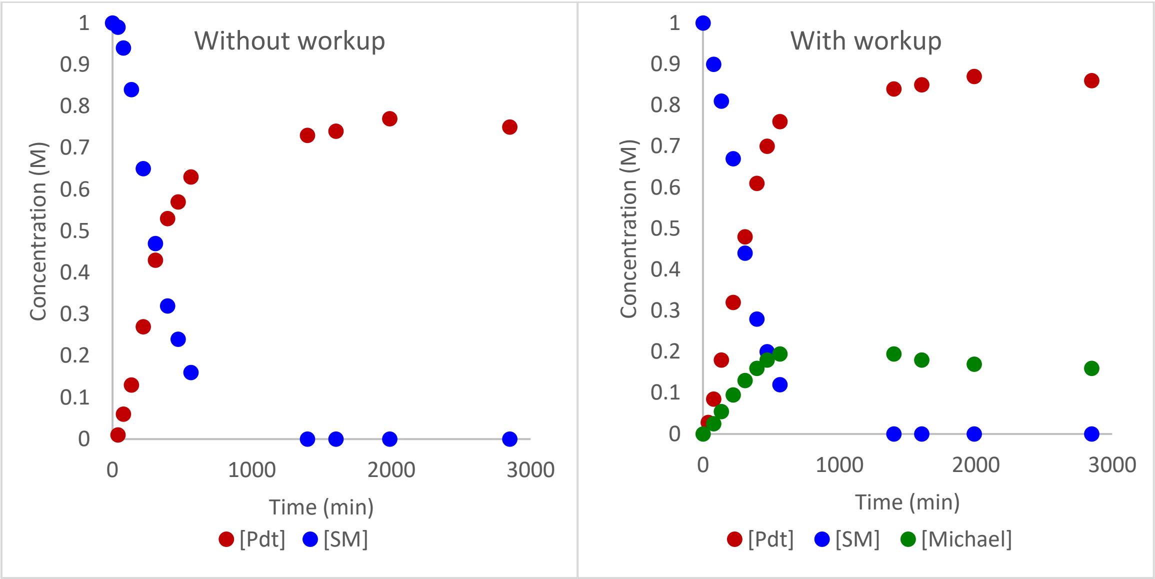

*Figure S1: Kinetic experiment with **1a** with or without workup*

Kinetic experiment with 1’a with workup with iminium iodide and iminium chloride

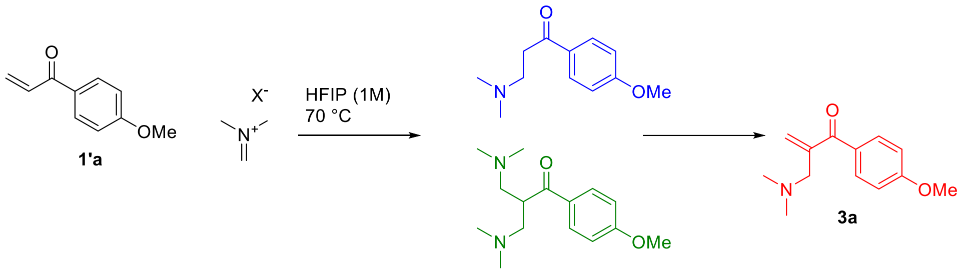

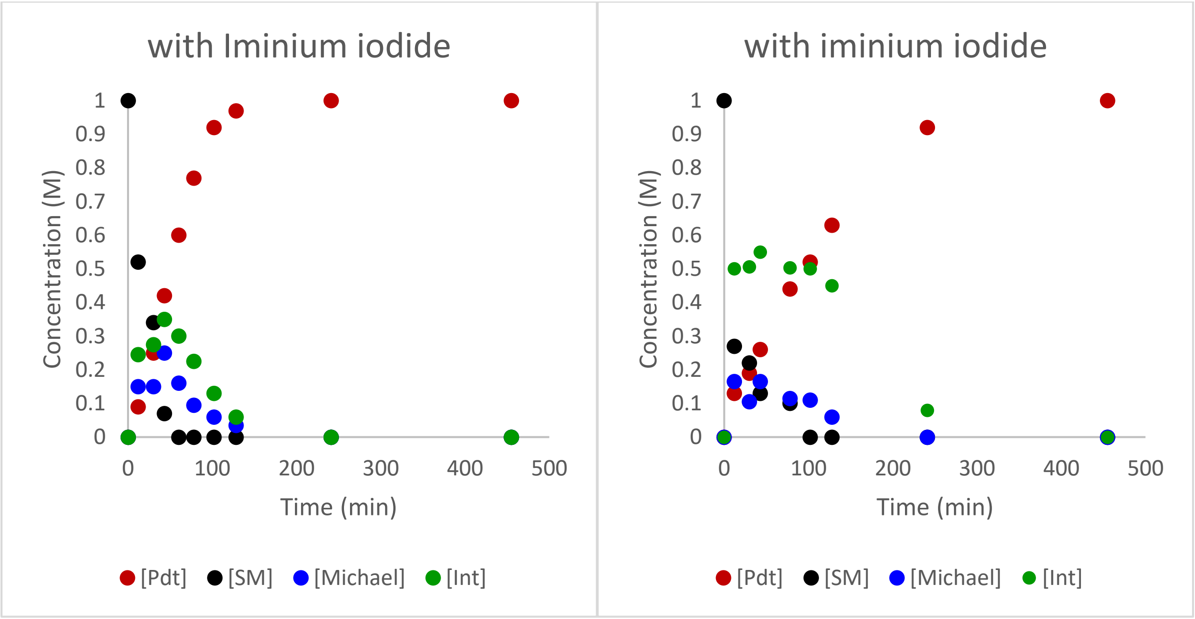

*Figure S2: Kinetic experiment with **1’a** with workup with iminium iodide and iminium chloride*

Kinetic experiment using 4 as starting material

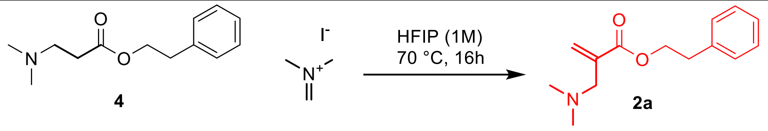

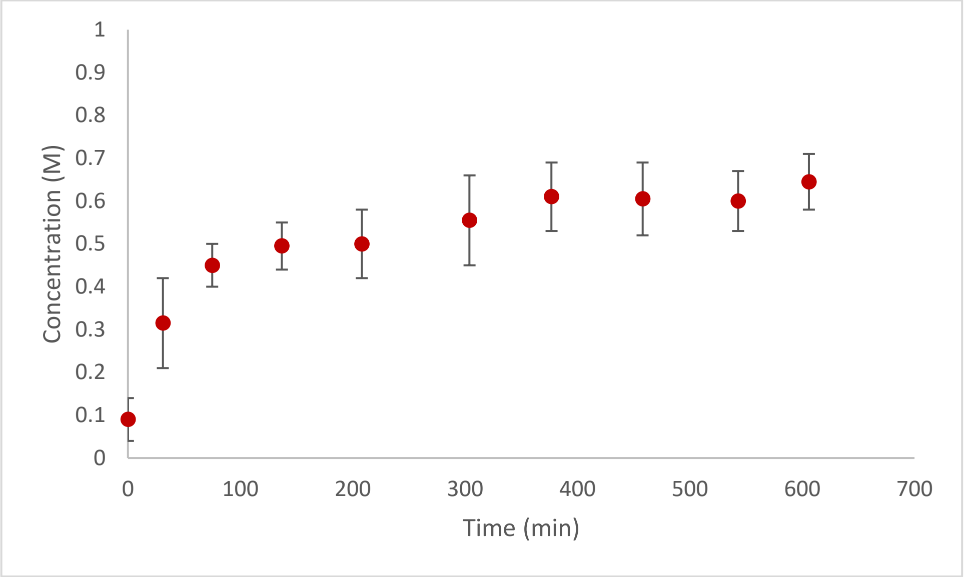

*Figure S3: Kinetic experiment using **4** as starting material*

Determination of the partial order in Michael acceptor

The concentration in Michael acceptor was varied to determine its partial order in the reaction according to a reported method. [12] This graphical analysis uses a variable normalisation of the time scale to enable the visual comparison of entire concentration reaction profiles. Whenever the curves plotted for different concentrations are aligned, the good partial order has been hypothesised. For all experiments, the concentration of Eschenmoser's salt was kept at 3 M. The concentration of Michael acceptor was varied: 1 M, 0.75 M, 0.5 M. For each hypothesised partial order (a), was plotted the concentration of product against the variable time scale normalised for the Michael acceptor (Equation S1).

$$\Sigma A^a \Delta t = \sum_{i=1}^{n} \left(\frac{[A]_i + [A]_{i-1}}{2} \right)^a (t_i - t_{i-1})$$

Equation S1: A represents the Michael acceptor; a is the hypothesized partial order in Michael acceptor, t is the reaction time in min; concentrations are expressed in M.

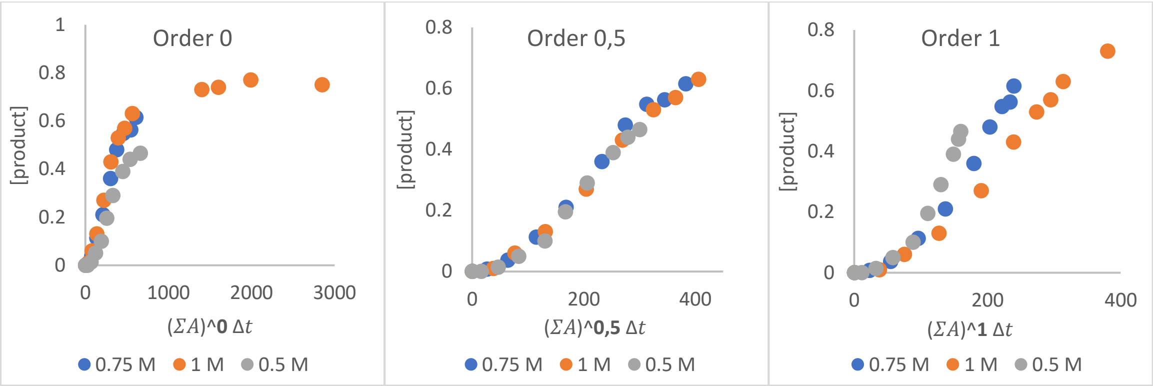

Figure S4: Determination of the partial order in acrylate

[12] J. Burés, *Angew. Chem. Int. Ed.* **2016**, *52*, 16084–16087

5: Phenethyl (*Z*)-2-((dimethylamino)methyl)-3-(4-nitrophenyl)acrylate

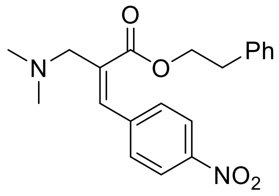

$C_{20}H_{22}N_2O_4$
MW: 354 g mol^{-1}
Yield: 8%
Orange oil

The compound was prepared using general procedure D from **1a** (35.26 mg, 0.20 mmol) and *N*-methyl-*N*-(4-nitrobenzylidene)methanaminium iodide[13] (184 mg, 0.60 mmol). The product was obtained as an orange oil (6 mg, 8%).

^{1}H NMR (700 MHz, CDCl$_3$) δ 8.24 (d, *J* = 8.8 Hz, 2H), 7.80 (d, *J* = 8.7 Hz, 2H), 7.76 (s, 1H), 7.33 (t, *J* = 7.5 Hz, 2H), 7.29 – 7.24 (m, 3H), 4.47 (t, *J* = 7.0 Hz, 2H), 3.21 (s, 2H), 3.05 (t, *J* = 7.0 Hz, 2H), 2.19 (s, 6H) ppm.

^{13}C NMR (176 MHz, CDCl$_3$) δ 167.9, 147.7, 141.9, 140.3, 137.9, 134.1, 131.3 (2C), 129.1 (2C), 128.7 (2C), 126.8, 123.7 (2C), 65.9, 54.4, 45.1 (2C), 35.3 ppm.

IR (neat) ν_{max}: 1708, 1518, 1343, 1240, 1100, 852, 747, 699 cm^{-1}.

HRMS (ESI) (m/z): calculated for $[M+H]^+$ ($C_{20}H_{23}N_2O_4$) requires 355.1652, found 355.1658.

[13] The corresponding iminium was obtained according to a reported procedure: M. Arend, N. Risch, *Synlett* **1997**, *1997*, 974–976.

Computations

The conformational space of all molecules has been initially searched using meta-dynamics simulations based on tight-binding quantum chemical calculations as implemented in CREST.[14,15]

The structures located with CREST have then been subjected to PBE0-D3BJ/def2-SVP[16,17,18,19,20] geometry optimization. The nature of all stationary points (minima and transition states) was verified through the computation of the vibrational frequencies. The thermal corrections to the Gibbs free energies were combined with the single point energies calculated at the PBE0-D3BJ/def2-TZVP level of theory to yield Gibbs free energies ("G_{298}") at 298.15 K. All energies are reported in kcal mol^{-1}.

The DFT calculations have been performed with the Gaussian 16 program package.[21]

The polarizable continuum model (PCM) with SMD parameters[22,23] was applied to consider solvent effects for both geometries and energies. SMD parameters of isopropanol are available in the used software package, while the solvation model for HFIP was considered as: ε=16.7; n^2=1.626; α=0.57; β=0.25; γ=30.13; Φ=0; Ψ=0.6.[24,25,26]

Free energies in solution have been corrected to a reference state of 1 mol l^{-1} at 298.15 K through the addition of RTln(24.46) = +7.925 kJ mol^{-1} to the gas phase (1 atm) free energies.

An additional file has been provided with the compilation of the cartesian coordinates for all calculated species.

---

[14] P. Pracht, F. Bohle, S. Grimme, *Phys. Chem. Chem. Phys.* **2020**, *22*, 7169–7192.
[15] S. Grimme, *J. Chem. Theory Comput.* **2019**, *15*, 2847–2862.
[16] J. P. Perdew, K. Burke, M. Ernzerhof, *Phys. Rev. Lett.* **1996**, *77*, 3865–3868.
[17] J. P. Perdew, K. Burke, M. Ernzerhof, *Phys. Rev. Lett.* **1997**, *78*, 1396.
[18] C. Adamo, V. Barone, *J. Chem. Phys.* **1999**, *110*, 6158.
[19] S. Grimme, J. Antony, S. Ehrlich, H. Krieg, *J. Chem. Phys.* **2010**, *132*, 154104.
[20] S. Grimme, S. Ehrlich, L. Goerigk, *J. Comput. Chem.* **2011**, *32*, 1456–1465.
[21] M. J. Frisch, G. W. Trucks, H. B. Schlegel, G. E. Scuseria, M. A. Robb, J. R. Cheeseman, G. Scalmani, V. Barone, G. A. Petersson, H. Nakatsuji, X. Li, M. Caricato, A. V. Marenich, J. Bloino, B. G. Janesko, R. Gomperts, B. Mennucci, H. P. Hratchian, J. V. Ortiz, A. F. Izmaylov, J. L. Sonnenberg, D. Williams-Young, F. Ding, F. Lipparini, F. Egidi, J. Goings, B. Peng, A. Petrone, T. Henderson, D. Ranasinghe, V. G. Zakrzewski, J. Gao, N. Rega, G. Zheng, W. Liang, M. Hada, M. Ehara, K. Toyota, R. Fukuda, J. Hasegawa, M. Ishida, T. Nakajima, Y. Honda, O. Kitao, H. Nakai, T. Vreven, K. Throssell, J. A. Montgomery, Jr., J. E. Peralta, F. Ogliaro, M. J. Bearpark, J. J. Heyd, E. N. Brothers, K. N. Kudin, V. N. Staroverov, T. A. Keith, R. Kobayashi, J. Normand, K. Raghavachari, A. P. Rendell, J. C. Burant, S. S. Iyengar, J. Tomasi, M. Cossi, J. M. Millam, M. Klene, C. Adamo, R. Cammi, J. W. Ochterski, R. L. Martin, K. Morokuma, O. Farkas, J. B. Foresman, and D. J. Fox, *Gaussian 16, Revision C.01*, Gaussian, Inc., Wallingford CT, **2019**.
[22] E. Cancès, B. Mennucci, J. Tomasi, *J. Chem. Phys.* **1997**, *107*, 3032–3041.
[23] A. V. Marenich, C. J. Cramer, D. G. Truhlar, *J. Phys. Chem. B* **2009**, DOI 10.1021/jp810292n.
[24] L. Eberson, M. P. Hartshorn, O. Persson, *J. Chem. Soc. Perkin Trans. 2* **1995**, 1735–1744.
[25] E. Mayans, G. Ballano, J. Sendros, M. Font-Bardia, J. L. Campos, J. Puiggalí, C. Cativiela, C. Alemán, *ChemPhysChem* **2017**, *18*, 1888–1896.
[26] P. Winget, D. M. Dolney, D. J. Giesen, C. J. Cramer, D. G. Truhlar, *Minnesota solvent Descr. database* **1999**, https://comp.chem.umn.edu/solvation/mnsddb.pdf.

Bohme's and Eschenmoser's salts dissociation

The calculations were performed applying the described computational approach (*vide supra*) for molecular clusters consisting of the salt $(CH_3)NCH_2X$ (X = Cl or I) and three HFIP solvent molecules. The global minima structures of the clusters (salt*3HFIP) obtained after DFT re-optimization of the sampled conformations for dissociated and associated states were compared.

Formation of enol

Following the initial step presented in the proposed mechanism (Michael addition), the zwitterionic species can be converted reversibly in the Michael adduct by a solvent-assisted proton transfer, as discussed in the main text. Apart from this transformation, the possibility to form an enol was also studied computationally, as shown in Figure S5.

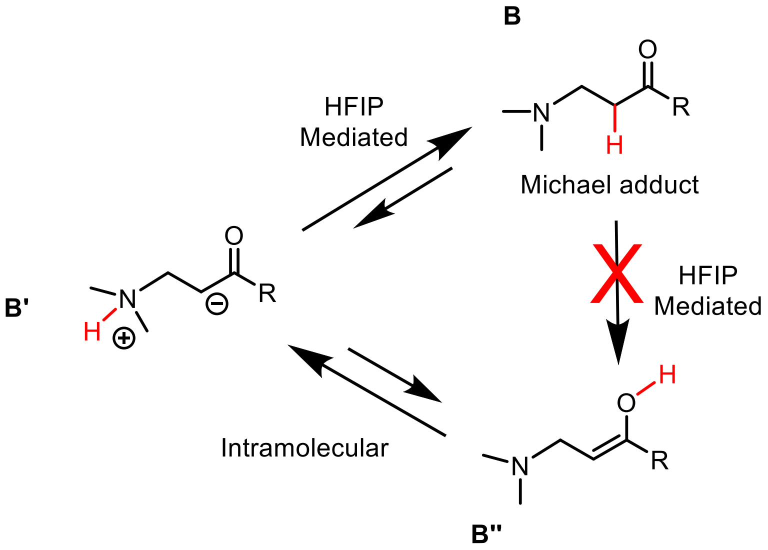

Figure S5: Computationally studied proton transfers leading to the formation of the enol species

The intermolecular proton transfer from the zwitterionic species **B'** to form the enol **B''** has negligible activation barriers for both the ester (R = OCH_2Ph) and the ketone (R = C_6H_5OMe). However, this process is endergonic: ΔG_{ester}(**B'**→**B''**) = 2.6 kcal mol^{-1} and ΔG_{ketone}(**B'**→**B''**) = 2.7 kcal mol^{-1}. Contrarily the HFIP-mediated proton transfer of the Michael adduct **3** to form the enol **B''** is highly unfavorable both kinetically ($\Delta G^{\ddagger}_{ester}$(**B**→**B''**) = 41.7 kcal mol^{-1} and $\Delta G^{\ddagger}_{ketone}$(**B**→**B''**) = 33.9 kcal mol^{-1}) and thermodynamically (ΔG_{ester}(**B**→**B''**) = 22.0 kcal mol^{-1} and ΔG_{ketone}(**B**→**B''**) = 14.3 kcal mol^{-1}). These results strongly suggest that the next step, the attack of the iminium, is performed by the zwitterionic species **B'** and not an enol **B''**, as presented in the proposed mechanism.

Solvent interactions

The solvent-mediated proton transfers presented in the manuscript do not include an explicit solvent model. To verify if additional solvent interactions could affect the obtained results, the transition state for the HFIP/isopropanol-mediated proton transfer of the diamine intermediate **C** was computed including a second solvent molecule. Conformational search was performed for hydrogen bonds established by the added solvent molecule to three acceptor groups in the transition state structure: the idle amine, the oxygen of the solvent molecule mediating the proton transfer and the carbonyl of the ester or ketone groups. The Gibbs free energy comparison of the most stable transition state structures of each type of H-bond is presented in Figure S6.

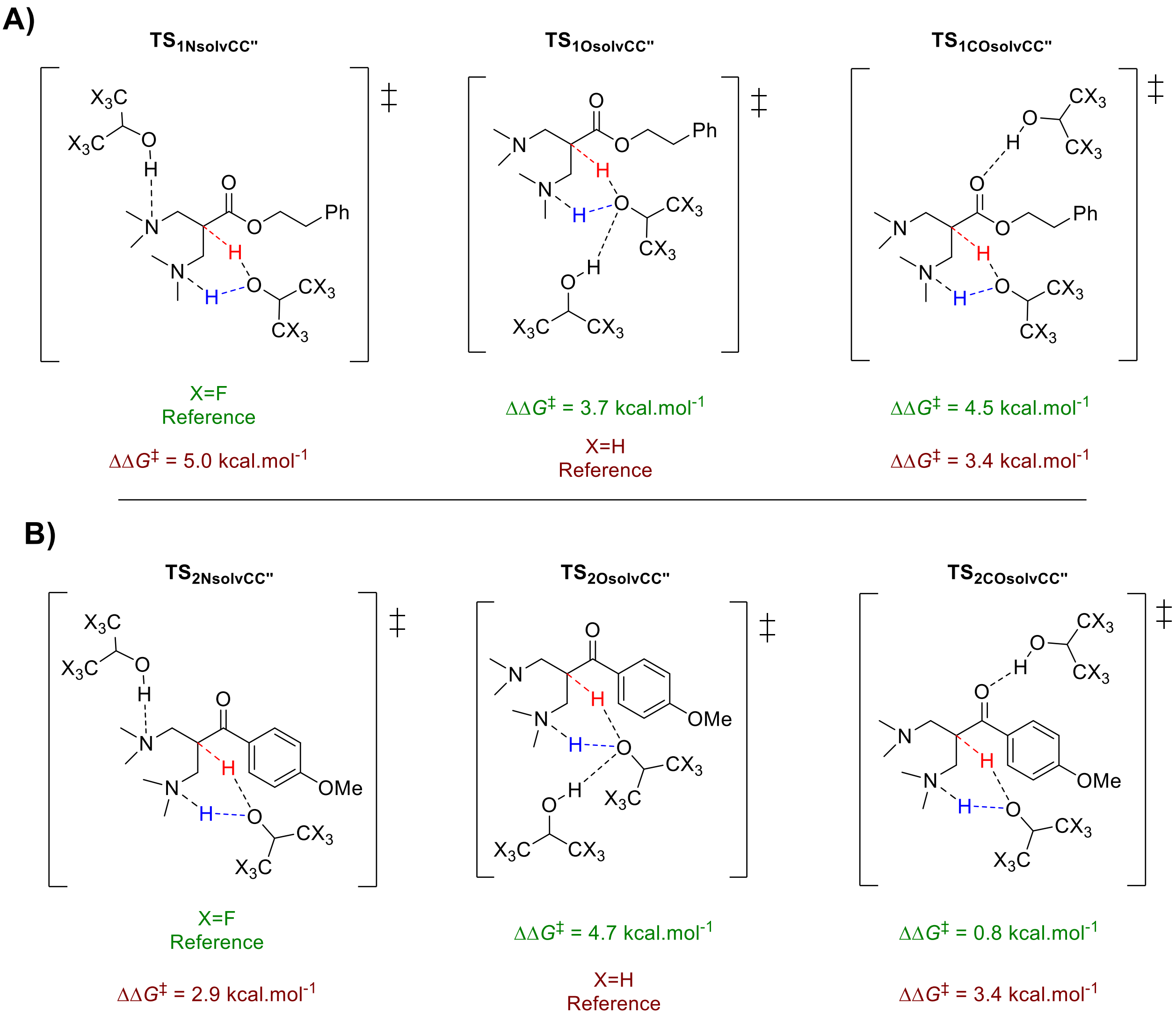

Figure S6: Comparison of the computed transition state structures containing explicit solvation for both HFIP (green) and isopropanol (brown) systems. A) ester; B) ketone.

The obtained results show that in both systems, an additional HFIP molecule establishes the strongest interaction with the free amine (H-bond), and isopropanol with the oxygen atom of the isopropanol molecule that is actively mediating the proton transfer. These results strongly suggest that interactions between solvent, and specially HFIP, and the carbonyl groups of the substrates do not significantly affect the kinetics of this step, and the implicit solvation model used is appropriate for this system. As

discussed in the manuscript (Scheme 2B), further addition of solvent molecules in the model would result in a sequence of HFIP-HFIP or isopropanol-isopropanol H-bonds.

X-ray Analysis

The X-ray intensity data were measured on Bruker D8 Venture diffractometer equipped with multilayer monochromator, Mo K/α INCOATEC micro focus sealed tube and Oxford cooling system. The structures were solved by *Patterson Method, Direct Methods and Dual Space*. Non-hydrogen atoms were refined with *anisotropic displacement parameters*. Hydrogen atoms were inserted at calculated positions and refined with riding model. The following software was used: *Bruker SAINT software package*[27] using a narrow-frame algorithm for frame integration, *SADABS*[28] for absorption correction, *OLEX2*[29] for structure solution, refinement, molecular diagrams and graphical user-interface, *Shelxle*[30] for refinement and graphical user-interface *SHELXS-2015*[31] for structure solution, *SHELXL-2015*[32] for refinement, *Platon*[33] for symmetry check. Experimental data and CCDC-Codes Experimental data (Available online: http://www.ccdc.cam.ac.uk/conts/retrieving.html) can be found in Table S5. Crystal data, data collection parameters, and structure refinement details are given in Tables S6 to S11. Asymmetric Unit visualized in Figure S7 to Figure S10.

Table S5 Experimental parameter and CCDC-Code.

**Sample**	**Machine**	**Source**	**Temp.**	**Detector Distance**	**Time/ Frame**	**#Frames**	**Frame width**	**CCDC**
	Bruker		[K]	[mm]	[s]		[°]	
**7a**	D8	Mo	126	30	1	3466	0.360	2079100
**7c**	D8	Mo	100	30	55	2401	0.360	2079101
**7d**	D8	Mo	100	30	20	1823	0.500	2079099

27 Bruker SAINT v8.38B Copyright © 2005-2019 Bruker AXS

28 Sheldrick, G. M. (1996). *SADABS.* University of Göttingen, Germany.

29 Dolomanov, O.V., Bourhis, L.J., Gildea, R.J, Howard, J.A.K. & Puschmann, H. , OLEX2, (2009), J. Appl. Cryst. 42, 339-341

30 C. B. Huebschle, G. M. Sheldrick and B. Dittrich, ShelXle: a Qt graphical user interface for SHELXL, J. Appl. Cryst., 44, (2011) 1281-1284

31 Sheldrick, G. M. (2015). *SHELXS v 2016/4* University of Göttingen, Germany.

32 Sheldrick, G. M. (2015). *SHELXL v 2016/4* University of Göttingen, Germany.

33 A. L. Spek, Acta Cryst. 2009, D65, 148-155

7a: 5-(2-((Dimethylammonio)methyl)-3-oxo-3-phenylpropyl)-2,2-dimethyl-4-oxo-4H-1,3-dioxin-6-olate

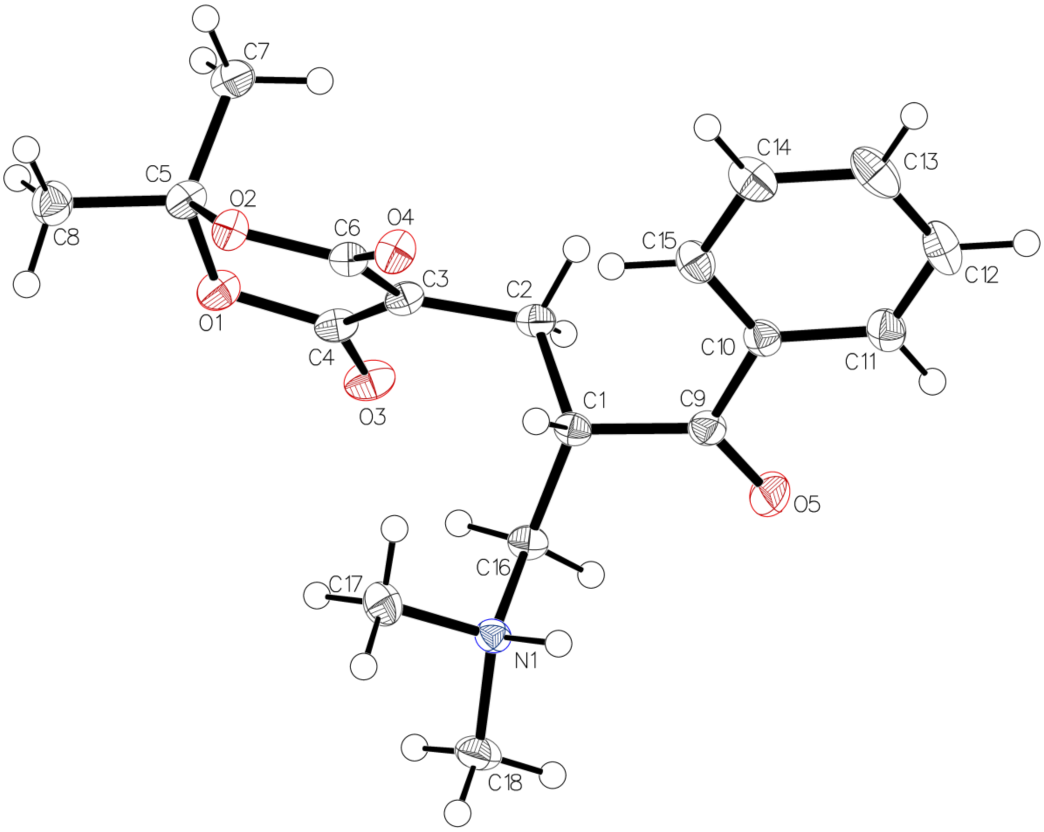

Figure S7: Asymmetric Unit drawn with 50% displacement ellipsoid. The bond precision for C-C single bonds is 0.0021Å.

Table S6 ***Sample and crystal data.***

Radiation [Å]	MoKα (λ = 0.71073)	Z	4	Measurement method	\f and \w scans
Crystal habit	clear colourless block	a [Å]	10.7514(3)		
Crystal size [mm^3]	0.325 × 0.264 × 0.187	b [Å]	12.1336(3)	Abs. correction type	multiscan
Empirical formula	C18H23NO5	c [Å]	13.1864(4)	Abs. correction Tmin	0.6859
Formula weight [g/mol]	333.37	α [°]	90	Abs. correction Tmax	0.7460
Temperature [K]	126.0	β [°]	90	Density (calculated) [g/cm3]	1.287
Crystal system	Orthorhombic	γ [°]	90	Absorption coefficient [mm^{-1}]	0.094
Space group	P212121	Volume [$Å^3$]	1720.21(8)	F (000) [e^-]	712.0

Table S7 Data collection and structure refinement.

2Θ range for data collection [°]	4.562 to 60.454	Index ranges		Goodness-of-fit on F^2	1.045
Reflections collected	100117	h	-15 ≤ h ≤ 15	Diff. peak and hole [$e^-Å^{-3}$]	0.21/-0.17
Data / restraints / parameters	5068/0/221	k	-17 ≤ k ≤ 17		
Refinement method	Direct Methods	l	-18 ≤ l ≤ 18	Function minimized	Σ w $(F_o^2 - F_c^2)^2$
		all data	R1 = 0.0416, wR2 = 0.0787	Weighting scheme	where
		I>2σ(I)	R1 = 0.0327, wR2 = 0.0754	$w=1/[\sigma^2(Fo^2) + (0.0401P)^2 + 0.2101P]$	$P=(F_o^2+2F_c^2)/3$

7c: 5-(2-((dimethylammonio)methyl)-3-oxo-3-(pyren-1-yl)propyl)-2,2-dimethyl-4-oxo-4H-1,3-dioxin-6-olate

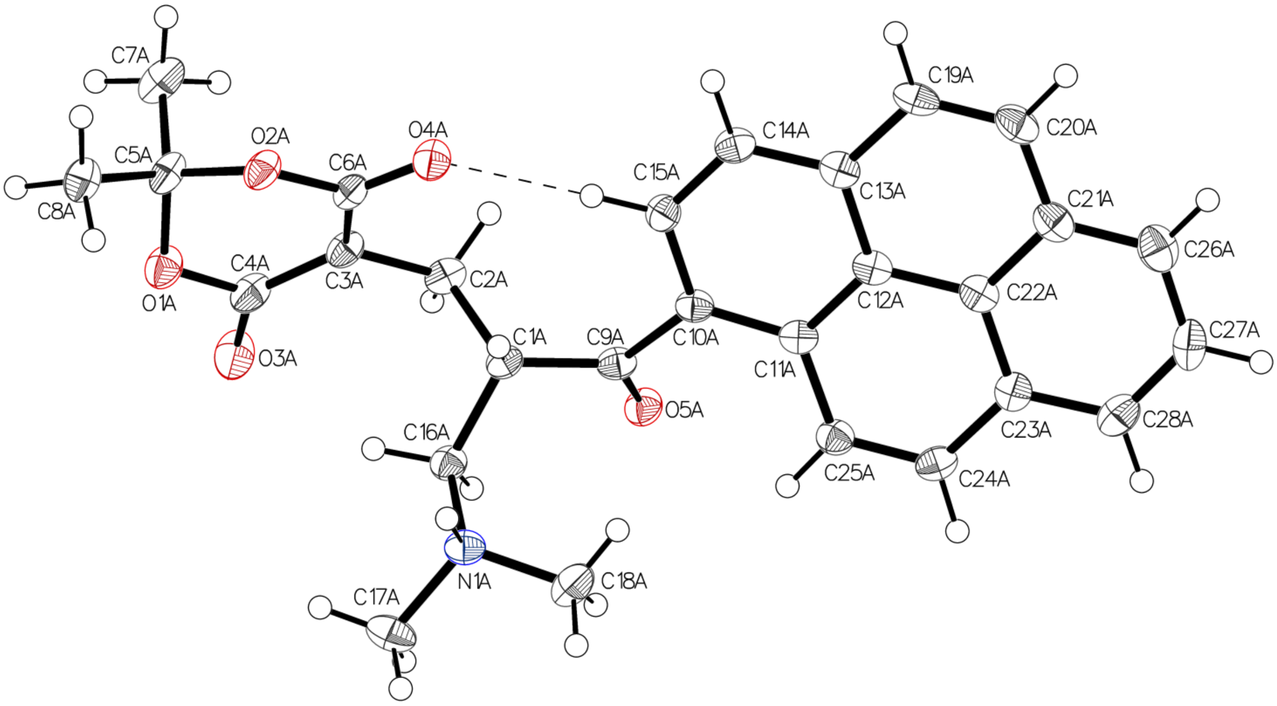

Figure S8: Asymmetric Unit drawn with 50% displacement ellipsoid. The bond precision for C-C single bonds is 0.0046Å. Second independent part (B) omitted for clarity.

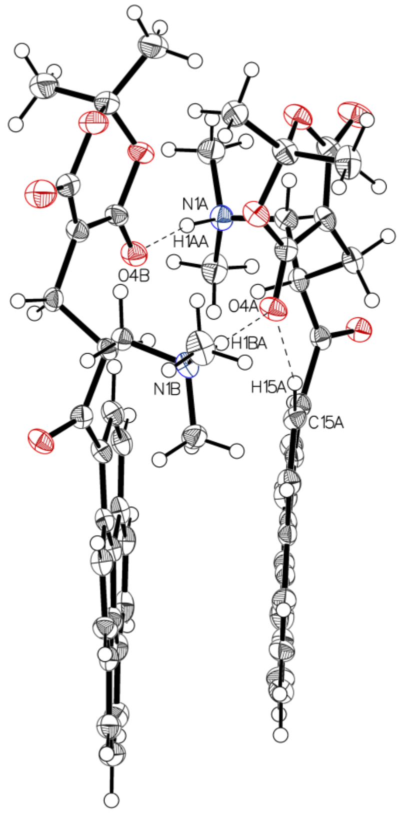

Figure S9: Interactions between two independent parts

.

Table S8 ***Sample and crystal data.***

Radiation [Å]	MoKα (λ = 0.71073)	Z	8	Measurement method	\f and \w scans
Crystal habit	clear yellow needle	a [Å]	31.389(6)		
Crystal size [mm^3]	0.08 × 0.04 × 0.02	b [Å]	16.604(3)	Abs. correction type	multiscan
Empirical formula	C28H27NO5	c [Å]	10.471(2)	Abs. correction Tmin	0.6764
Formula weight [g/mol]	457.50	α [°]	90	Abs. correction Tmax	0.7452
Temperature [K]	100.0	β [°]	90	Density (calculated) [g/cm3]	1.114
Crystal system	Orthorhombic	γ [°]	90	Absorption coefficient [mm^{-1}]	0.076
Space group	Pna21	Volume [$Å^3$]	5457.3(17)	F (000) [e^-]	1936.0

Table S9 Data collection and structure refinement.

2Θ range for data collection [°]	4.676 to 51.37	Index ranges		Goodness-of-fit on F^2	1.033
Reflections collected	142481	h	-38 ≤ h ≤ 38	Diff. peak and hole [$e^-Å^{-3}$]	0.15/-0.18
Data / restraints / parameters	10276/1/621	k	-20 ≤ k ≤ 20		
Refinement method	Dual Space	l	-12 ≤ l ≤ 12	Function minimized	Σ w $(F_o^2 - F_c^2)^2$
		all data	R1 = 0.0599, wR2 = 0.1061	Weighting scheme	where
		I>2σ(I)	R1 = 0.0424, wR2 = 0.0999	$w=1/[\sigma^2(Fo^2) + (0.0550P)^2]$	$P=(F_o^2+2F_c^2)/3$

7d: 5-(2-((Dimethylammonio)methyl)-3-(4-nitrophenyl)-3-oxopropyl)-2,2-dimethyl-4-oxo-4H-1,3-dioxin-6-olate

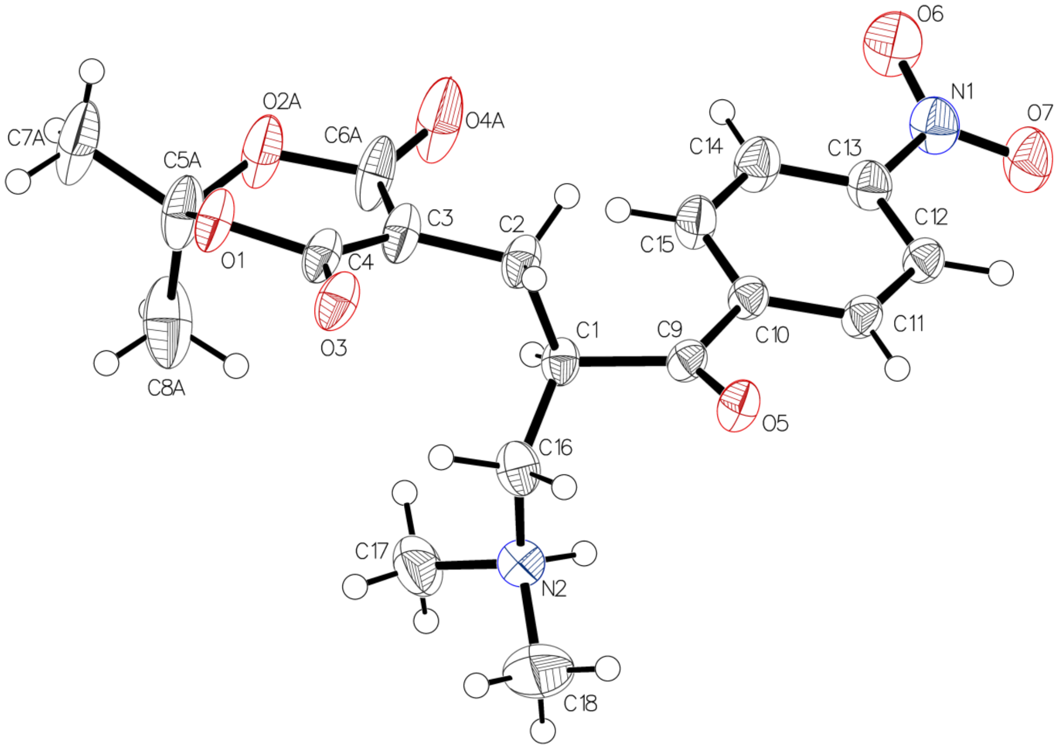

Figure S10: Asymmetric Unit drawn with 50% displacement ellipsoid. The bond precision for C-C single bonds is 0.0018Å. Second part (B) omitted for clarity. Main residue disorder 22%.

Table S10 ***Sample and crystal data.***

Radiation [Å]	MoKα (λ = 0.71073)	Z	4	Measurement method	\f and \w scans
Crystal habit	clear yellow block	a [Å]	7.0624(3)		
Crystal size [mm³]	0.2 × 0.14 × 0.12	b [Å]	15.9596(11)	Abs. correction type	multiscan
Empirical formula	C18H22N2O7	c [Å]	19.8071(10)	Abs. correction Tmin	0.6382
Formula weight [g/mol]	378.37	α [°]	90	Abs. correction Tmax	0.7460
Temperature [K]	100.0	β [°]	90.549(3)	Density (calculated) [g/cm3]	1.126
Crystal system	Monoclinic	γ [°]	90	Absorption coefficient [mm^{-1}]	0.087
Space group	P21/n	Volume [Å³]	2232.4(2)	F (000) [e^-]	800.0

Table S11 Data collection and structure refinement.

2Θ range for data collection [°]	4.84 to 60.066	Index ranges		Goodness-of-fit on F^2	1.030
Reflections collected	80437	h	$-9 \le h \le 9$	Diff. peak and hole [$e^{-}Å^{-3}$]	0.34/-0.24
Data / restraints / parameters	6508/25/272	k	$-22 \le k \le 22$		
Refinement method	Patterson Method	l	$-27 \le l \le 27$	Function minimized	$\Sigma w (F_o^2 - F_c^2)^2$
		all data	R1 = 0.0640, wR2 = 0.1603	Weighting scheme	where
		I>2σ(I)	R1 = 0.0521, wR2 = 0.1496	$w=1/[\sigma^2(Fo^2) + (0.0822P)^2 + 0.6767P]$	$P=(F_o^2+2F_c^2)/3$

NMR analysis in deuterated HFIP

0.27 mmol of Eschenmoser's or Böhme's salt were dissolved in 0.6 mL of HFIP-d_2. The mixture was monitored by ^{1}H NMR for 10 days at room temperature followed by 4 days at 75 °C without showing any change.

Böhme's salt spectrum

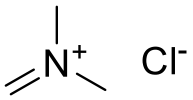

^{1}H NMR (600 MHz, HFIP-d2) δ 7.76 (iminium-CH_2), 5.23 (OH-HFIP), 4.98 (acetal-CH_2), 4.51 (chloro-amine, CH_2), 4.41 (m, CH-HFIP), 3.74 (iminium-NMe_2), 2.79 (chloro-amine, NMe_2), 2.78 (dimethylamine).

^{13}C NMR (151 MHz, HFIP-d2) δ 166.91 (m, iminium-CH_2), 120.66 (q, J = 281.2 Hz, HFIP-CF_3), 89.57 (acetal CH_2), 79.33(chloro-amine, CH_2), 68.18 (m, HFIP), 48.11(iminium-NMe_2), 37.41 (chloro-amine, NMe_2), 34.23 (dimethylamine).

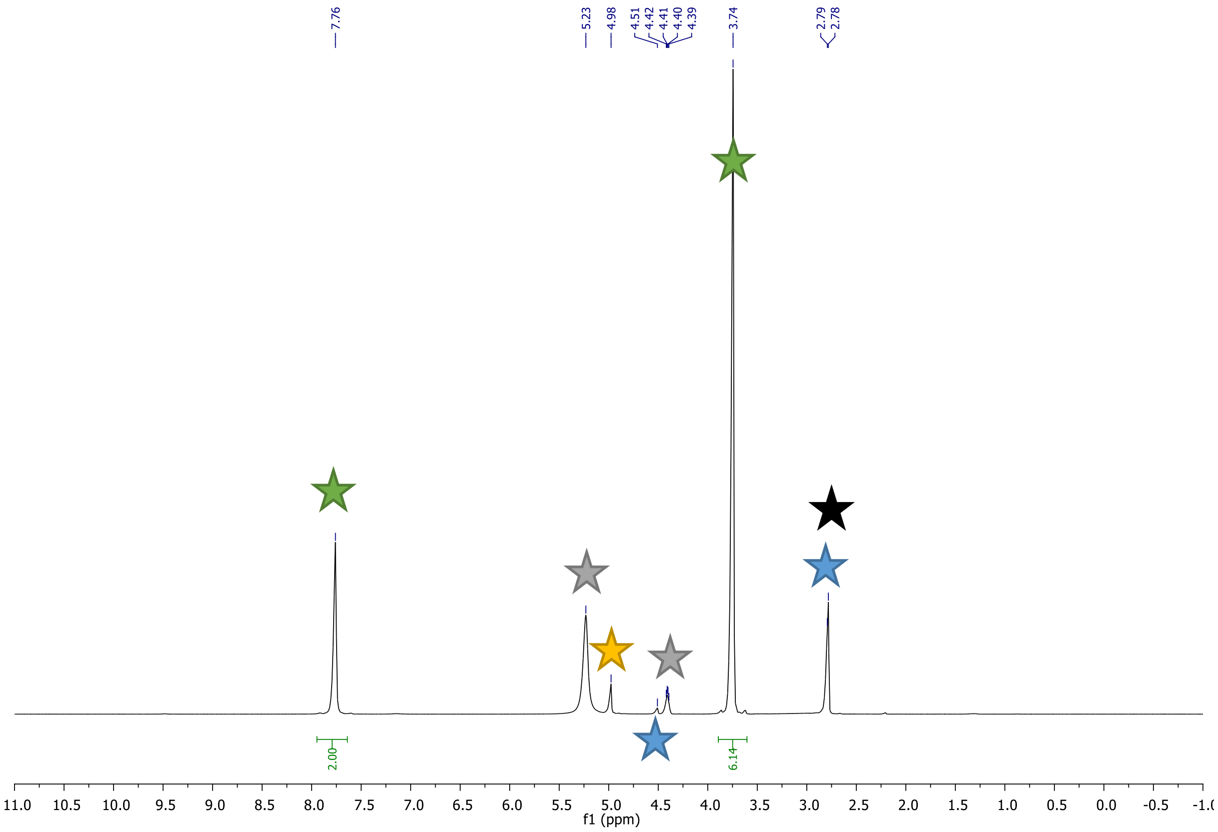
7.76
5.23
4.98
4.51
4.42
4.41
4.40
4.39
3.74
2.79
2.78
2.00
6.14
f1 (ppm)

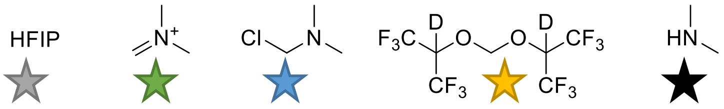
HFIP

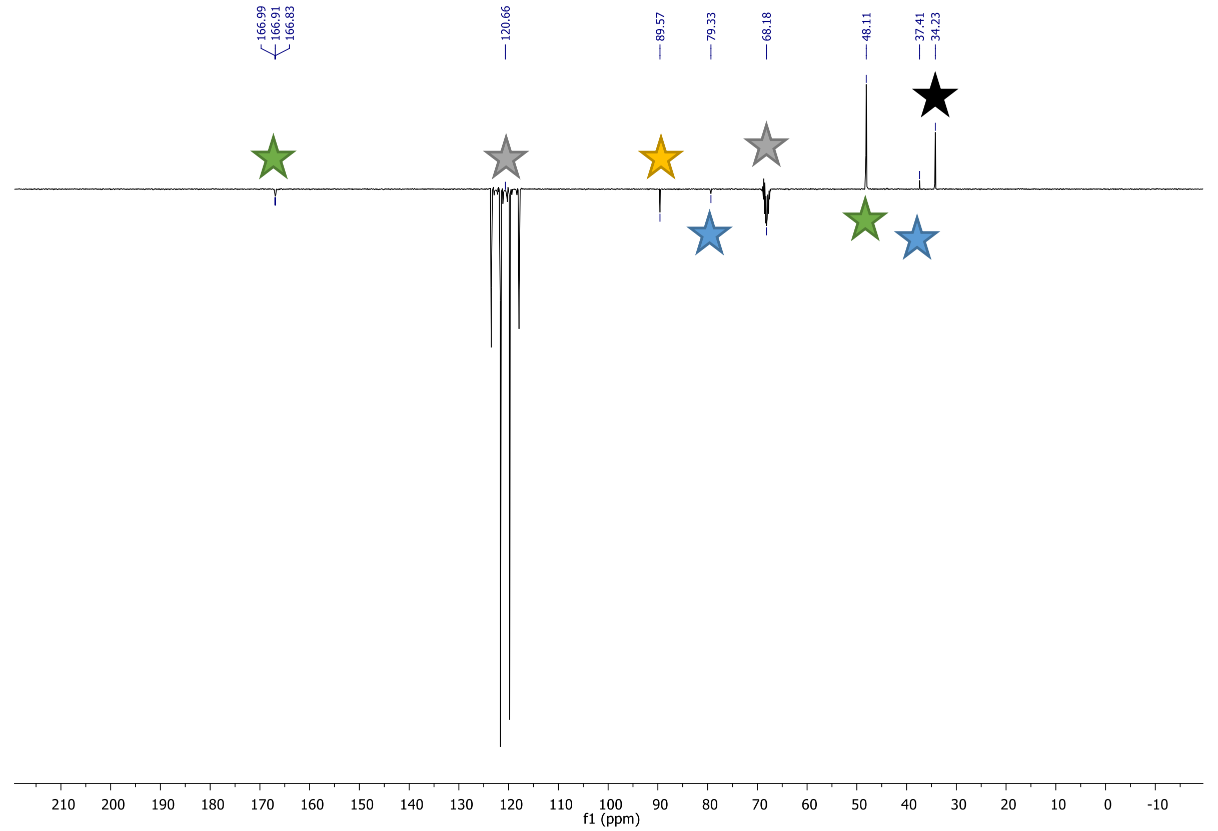
166.99
166.91
166.83
120.66
89.57
79.33
68.18
48.11
37.41
34.23
f1 (ppm)

Eschenmoser's salt spectrum

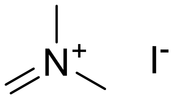

^{1}H NMR (600 MHz, HFIP-d2) δ 7.76 (iminium-CH_2), 4.97 (acetal-CH_2), 4.85(OH-HFIP), 4.41(CH-HFIP), 3.71(iminium-NMe_2), 3.08, 2.77 (dimethylamine).

^{13}C NMR (151 MHz, HFIP-d2) δ 166.30 (iminium-CH_2), 120.66(q, J = 281.2 Hz, HFIP-CF_3), 89.62 (acetal-CH_2), 68.18 (m, HFIP), 50.46, 48.32(iminium-NMe_2), 34.41(dimethylamine). 21.40.

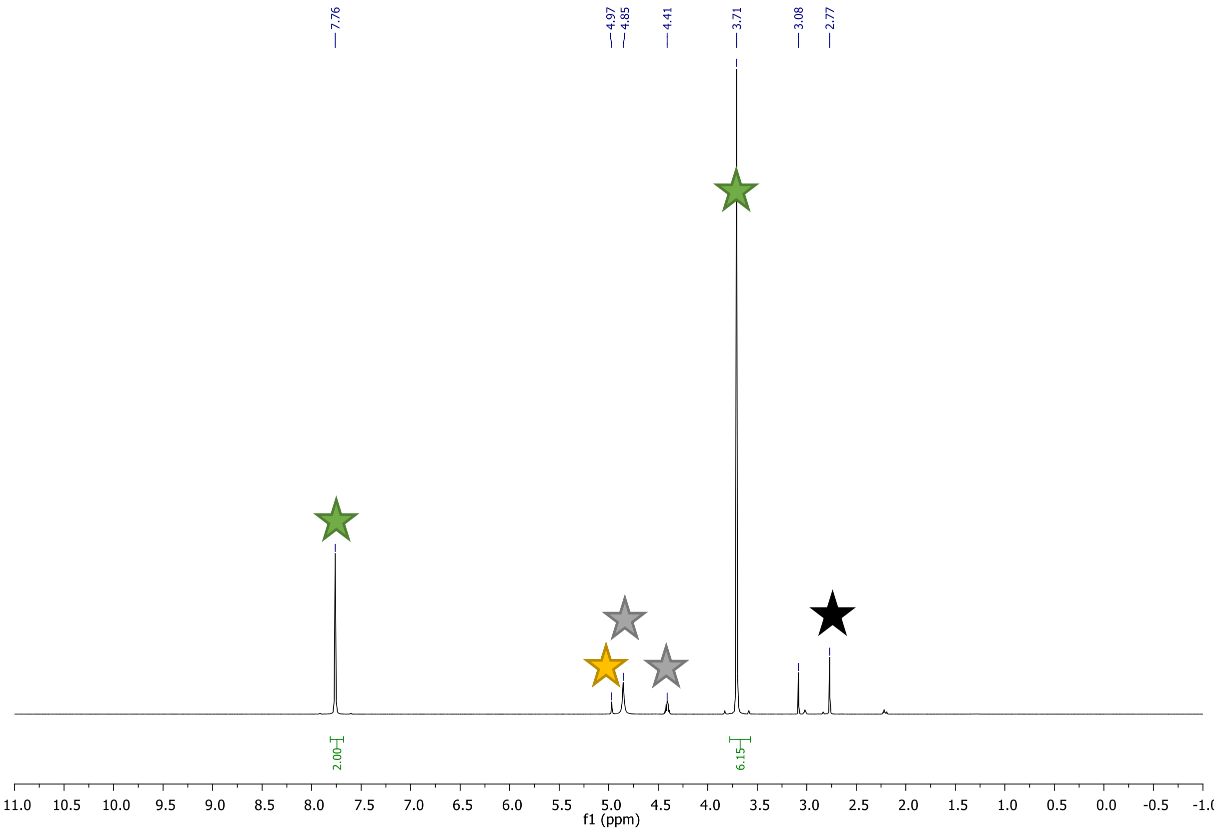
7.76
4.97
4.85
4.41
3.71
3.08
2.77
2.00
6.15
11.0 10.5 10.0 9.5 9.0 8.5 8.0 7.5 7.0 6.5 6.0 5.5 5.0 4.5 4.0 3.5 3.0 2.5 2.0 1.5 1.0 0.5 0.0 -0.5
f1 (ppm)

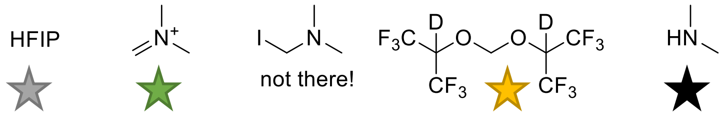
HFIP
not there!

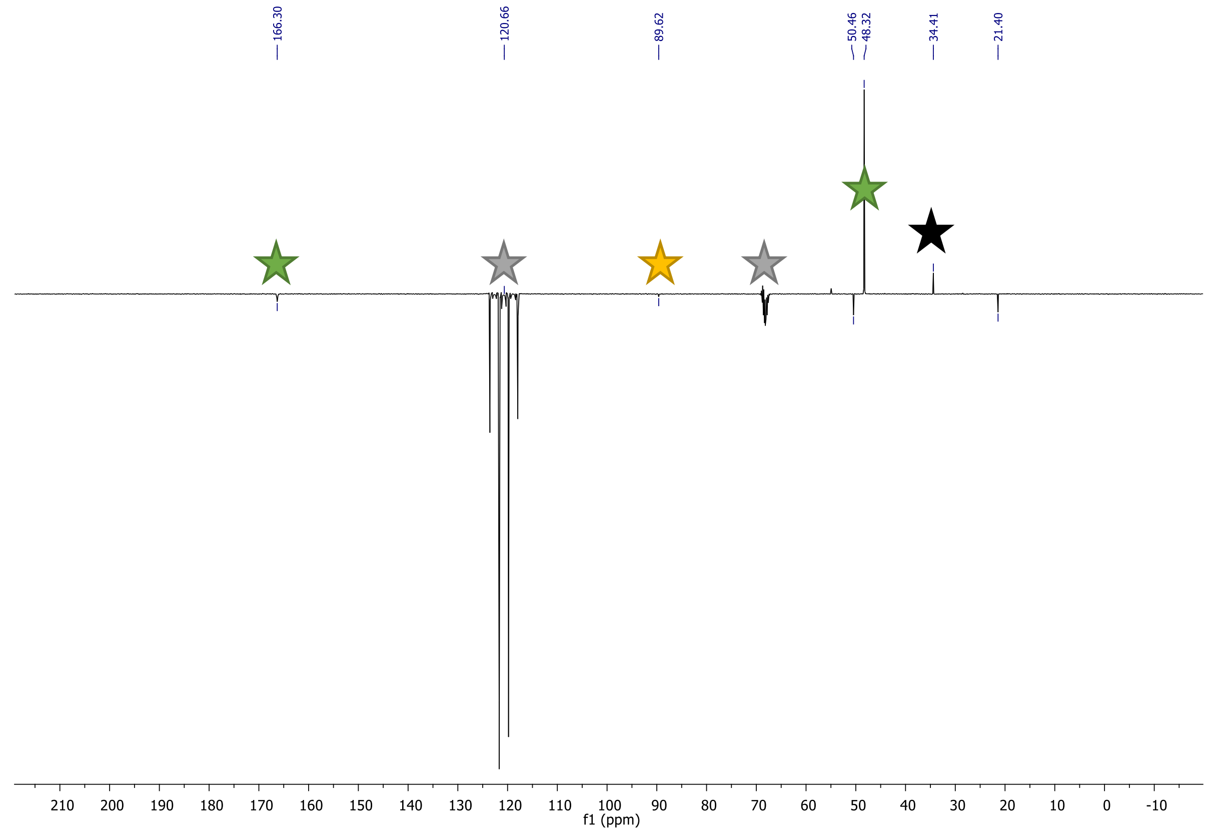
166.30
120.66
89.62
50.46
48.32
34.41
21.40
210 200 190 180 170 160 150 140 130 120 110 100 90 80 70 60 50 40 30 20 10 0 -10
f1 (ppm)

NMR spectra

1a-d_2: Phenethyl acrylate-3,3-d_2

^{1}H NMR (400 MHz, $CDCl_3$)

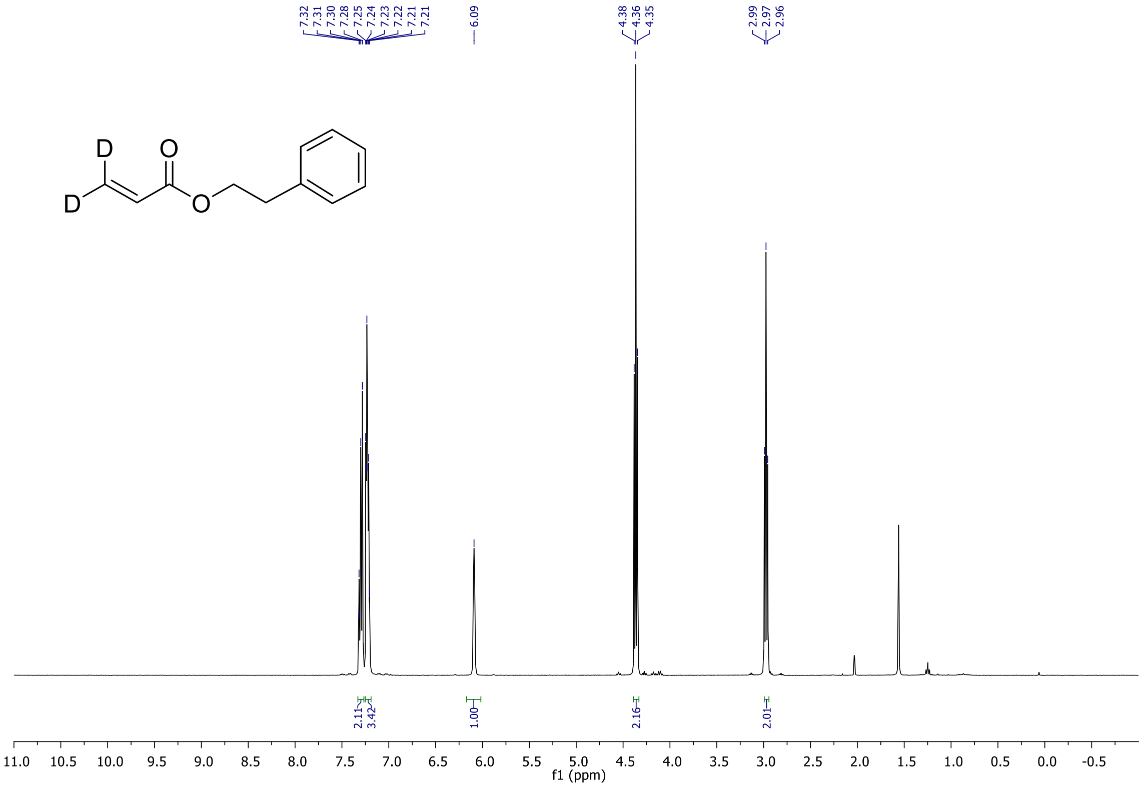

^{13}C NMR (101 MHz, $CDCl_3$)

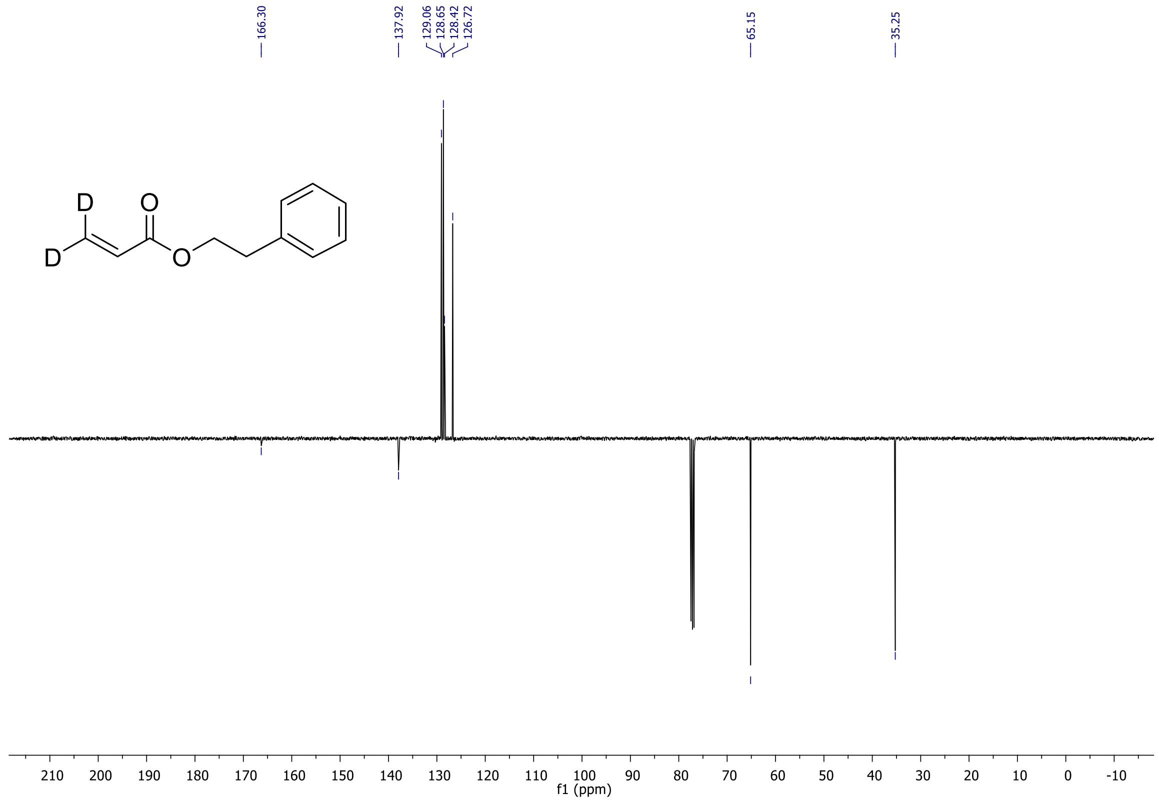

1b: 2,2-Diphenylethyl acrylate

^{1}H NMR (400 MHz, $CDCl_3$)

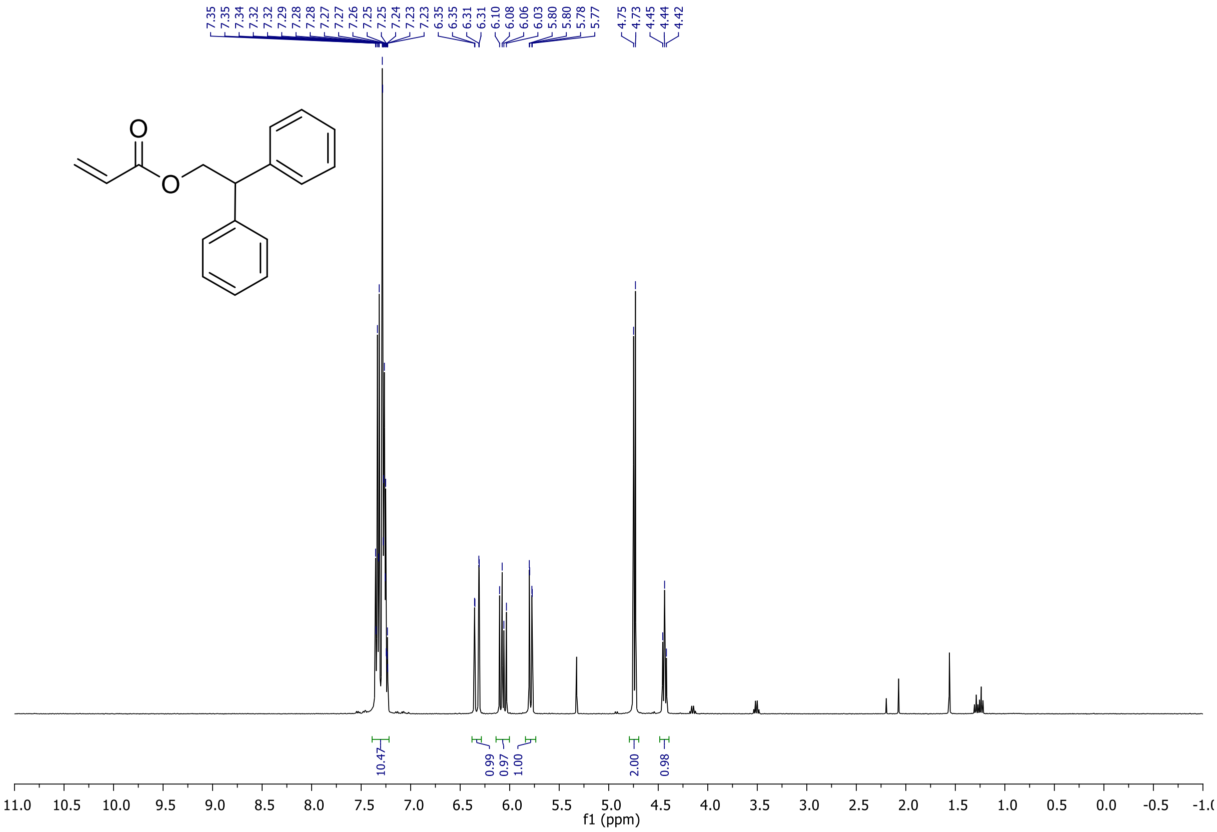

^{13}C NMR (101 MHz, $CDCl_3$)

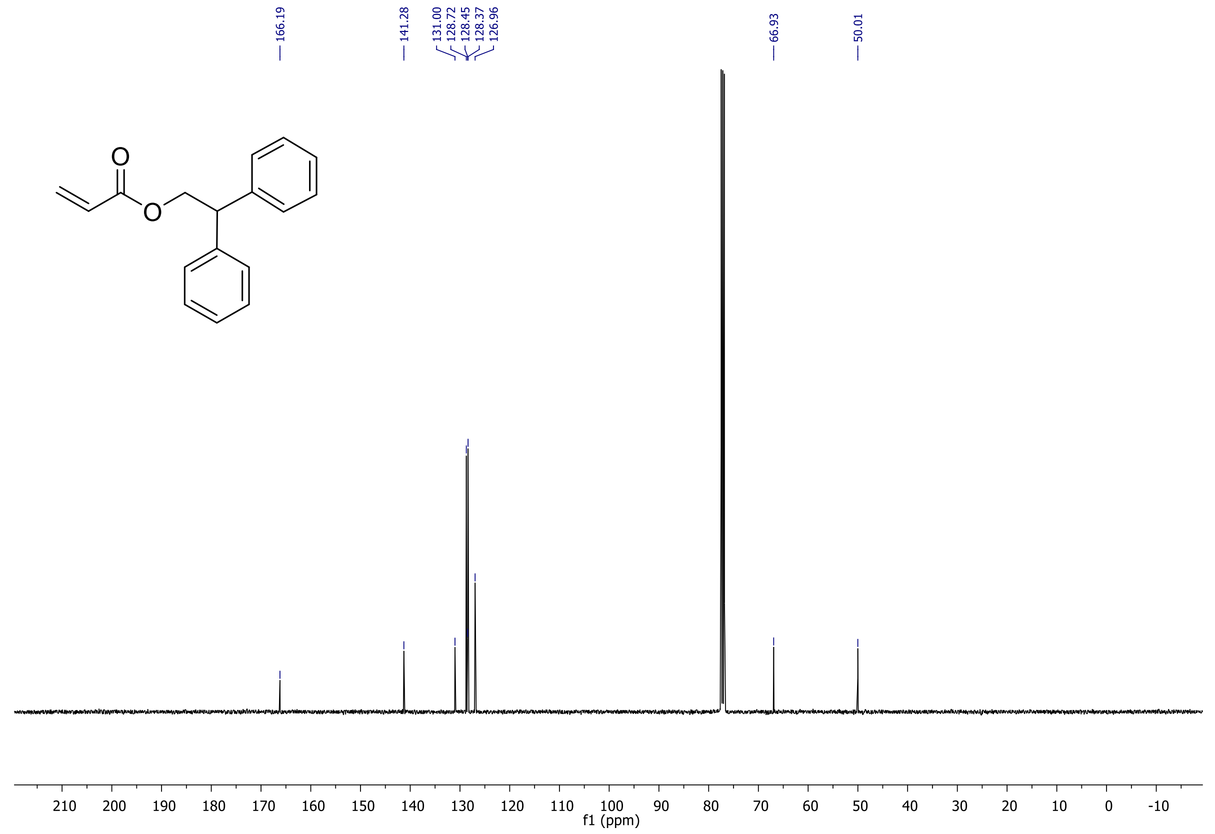

1d: (1-(Cyanomethyl)cyclopropyl)methyl acrylate

^{1}H NMR (400 MHz, $CDCl_3$)

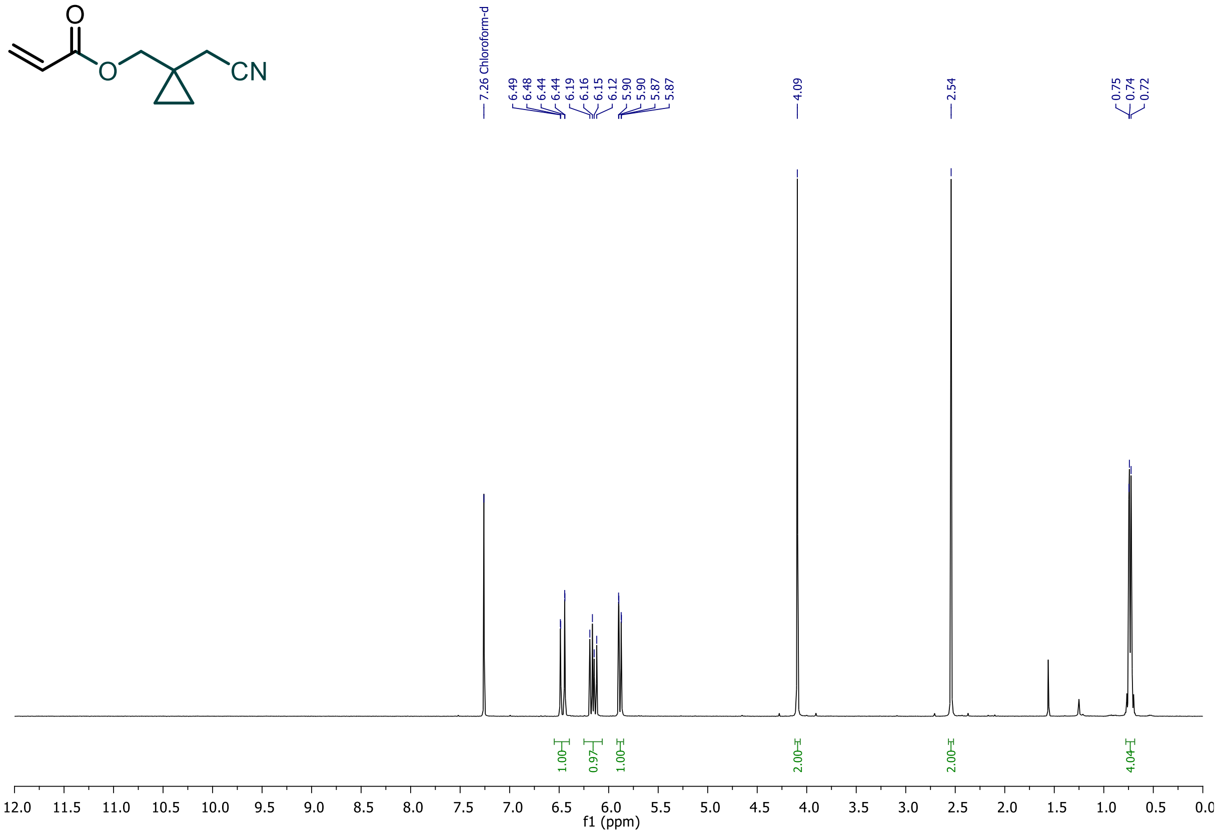

^{13}C NMR (101 MHz, $CDCl_3$)

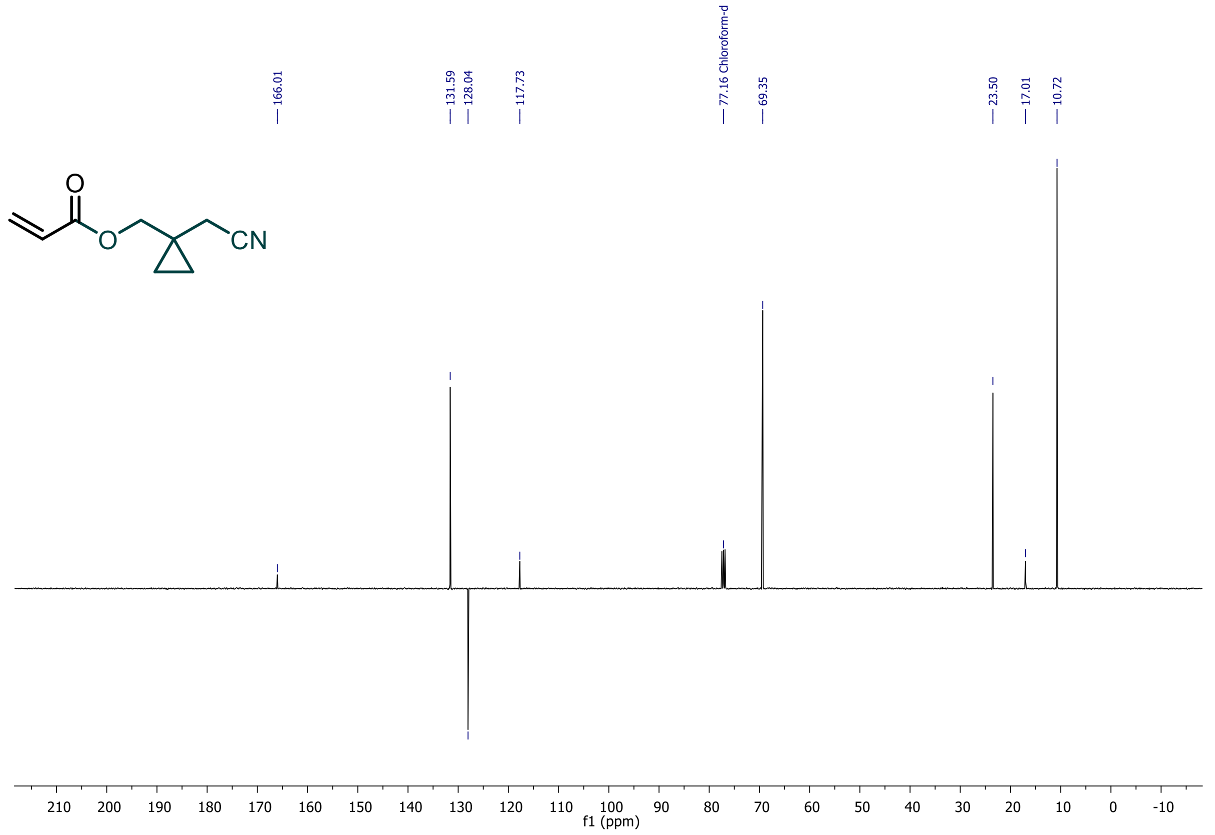

1f: 3-(1,3-Dioxoisoindolin-2-yl)propyl acrylate

^{1}H NMR (400 MHz, CDCl$_3$)

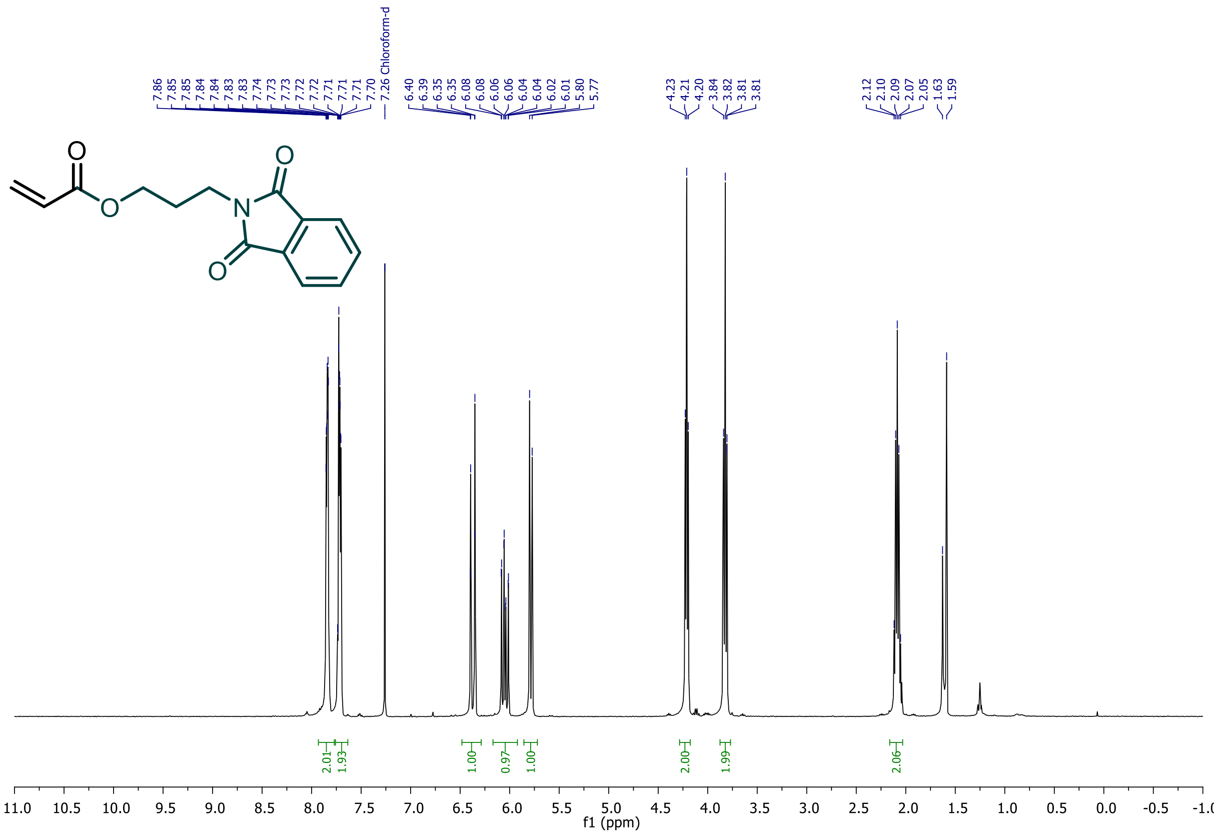

^{13}C NMR (100 MHz, CDCl$_3$)

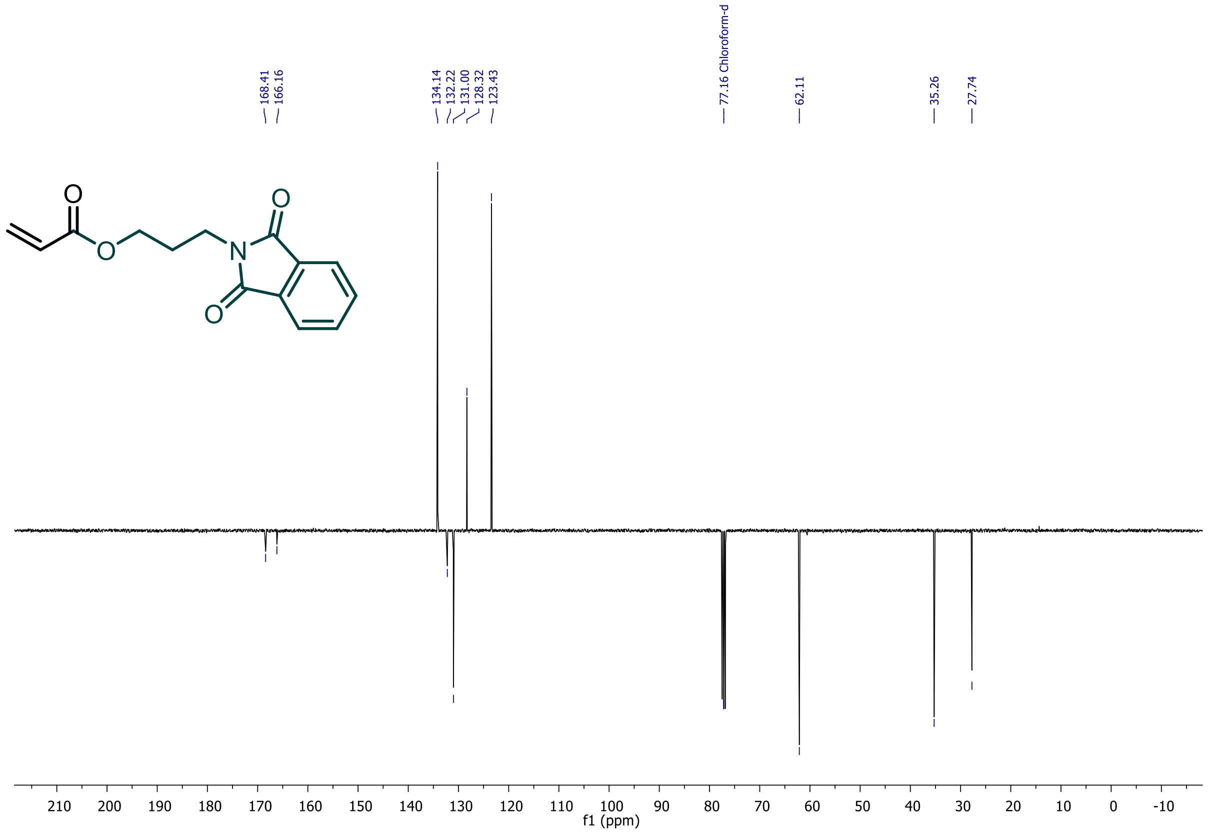

1g: (1*R*)-(6-Methoxyquinolin-4-yl)((1*S*,4*S*,5*R*)-5-vinylquinuclidin-2-yl)methyl acrylate

^{1}H NMR (400 MHz, CDCl$_3$)

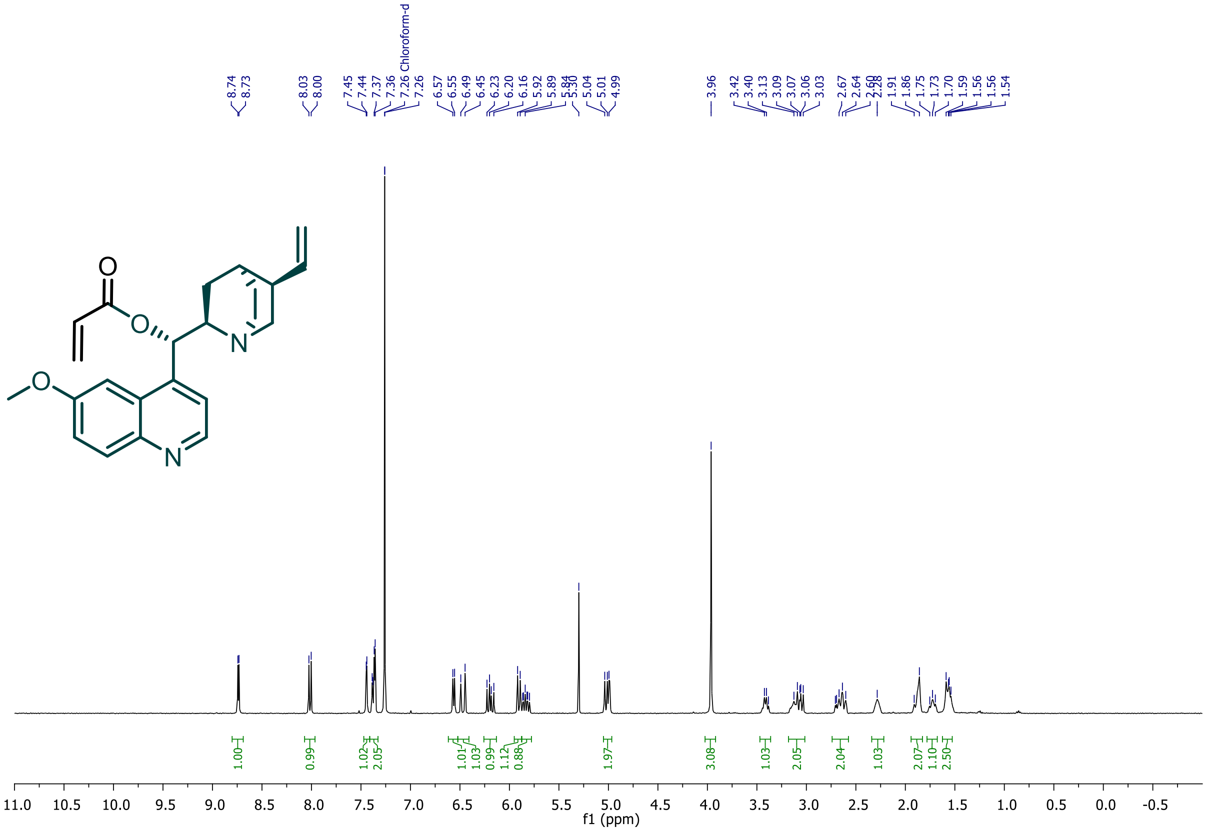

^{13}C NMR (101 MHz, CDCl$_3$)

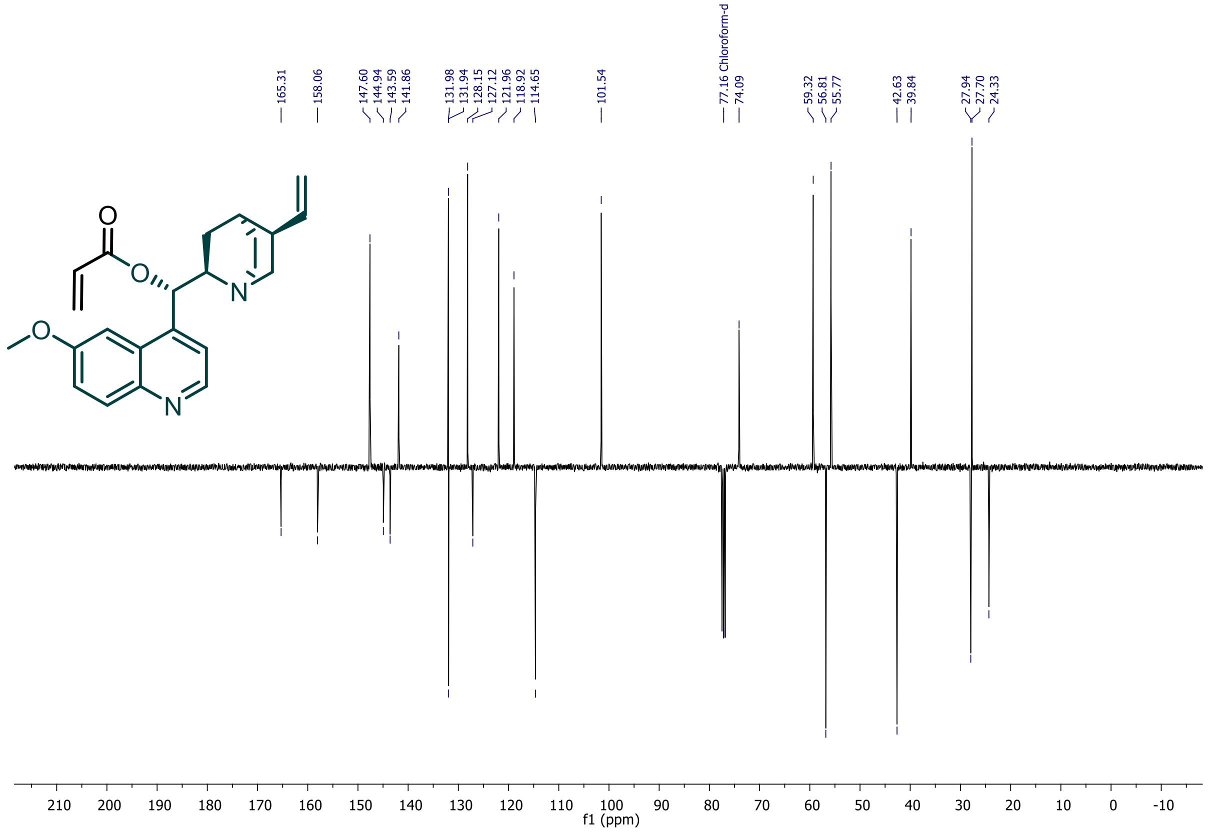

1i: *S*-Octyl prop-2-enethioate

^{1}H NMR (400 MHz, $CDCl_3$)

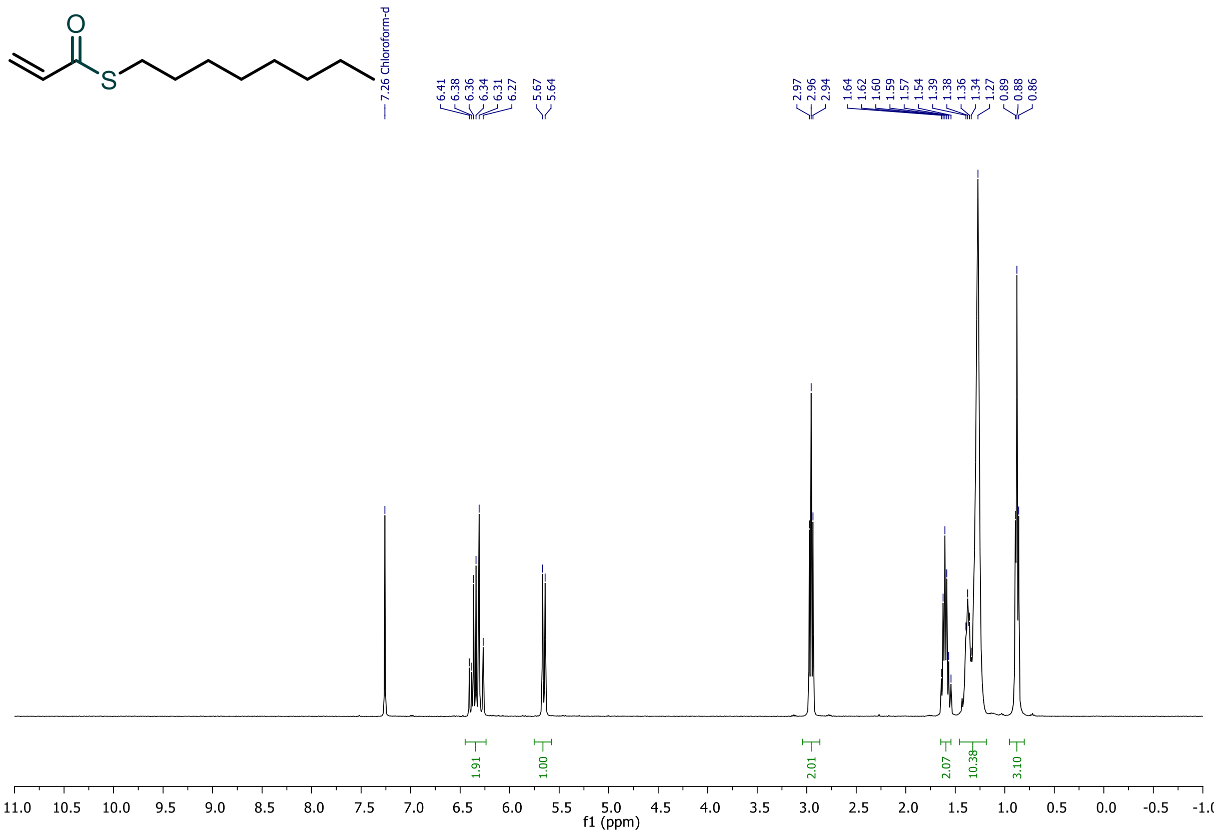

^{13}C NMR (100 MHz, $CDCl_3$)

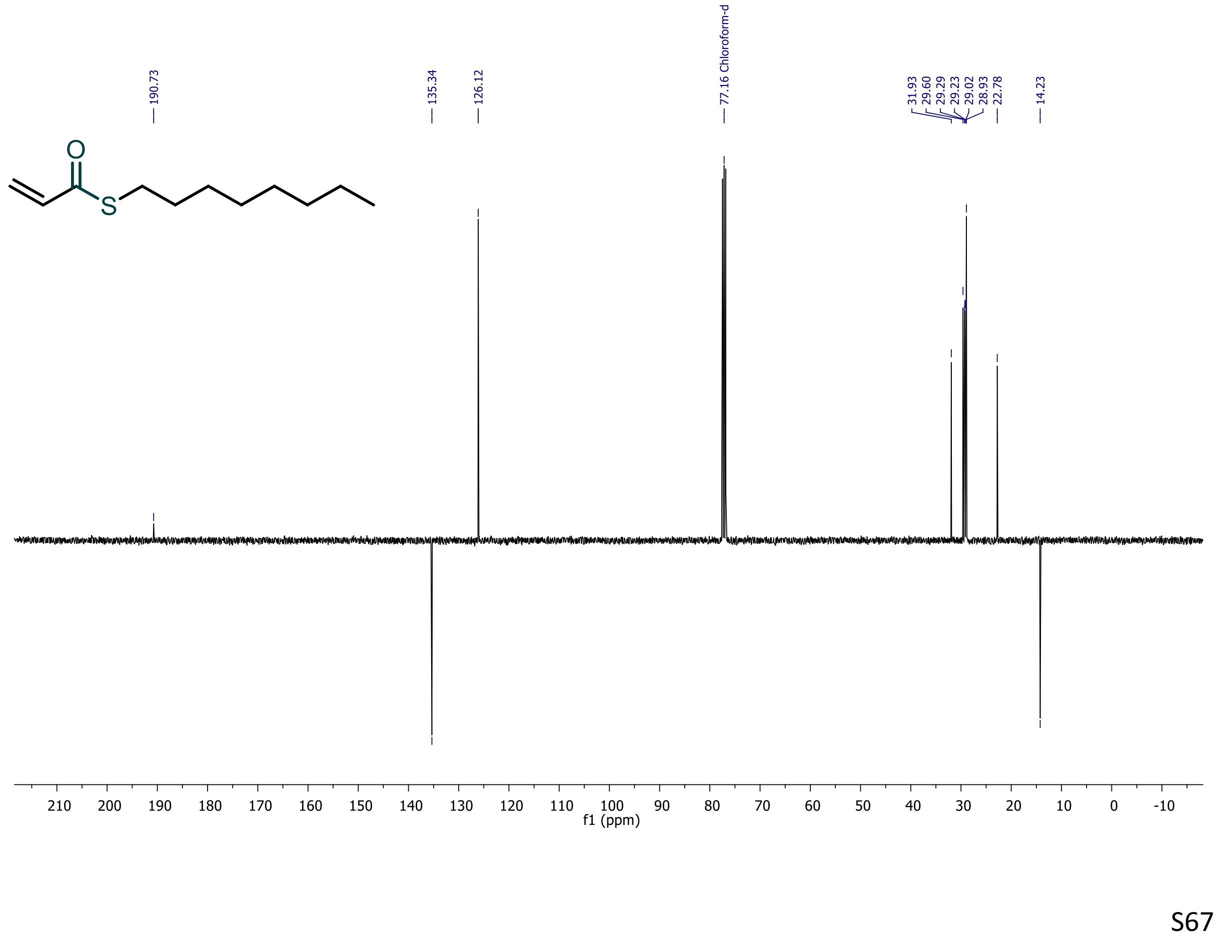

2a: Phenethyl 2-((dimethylamino)methyl)acrylate

^{1}H NMR (400 MHz, $CDCl_3$)

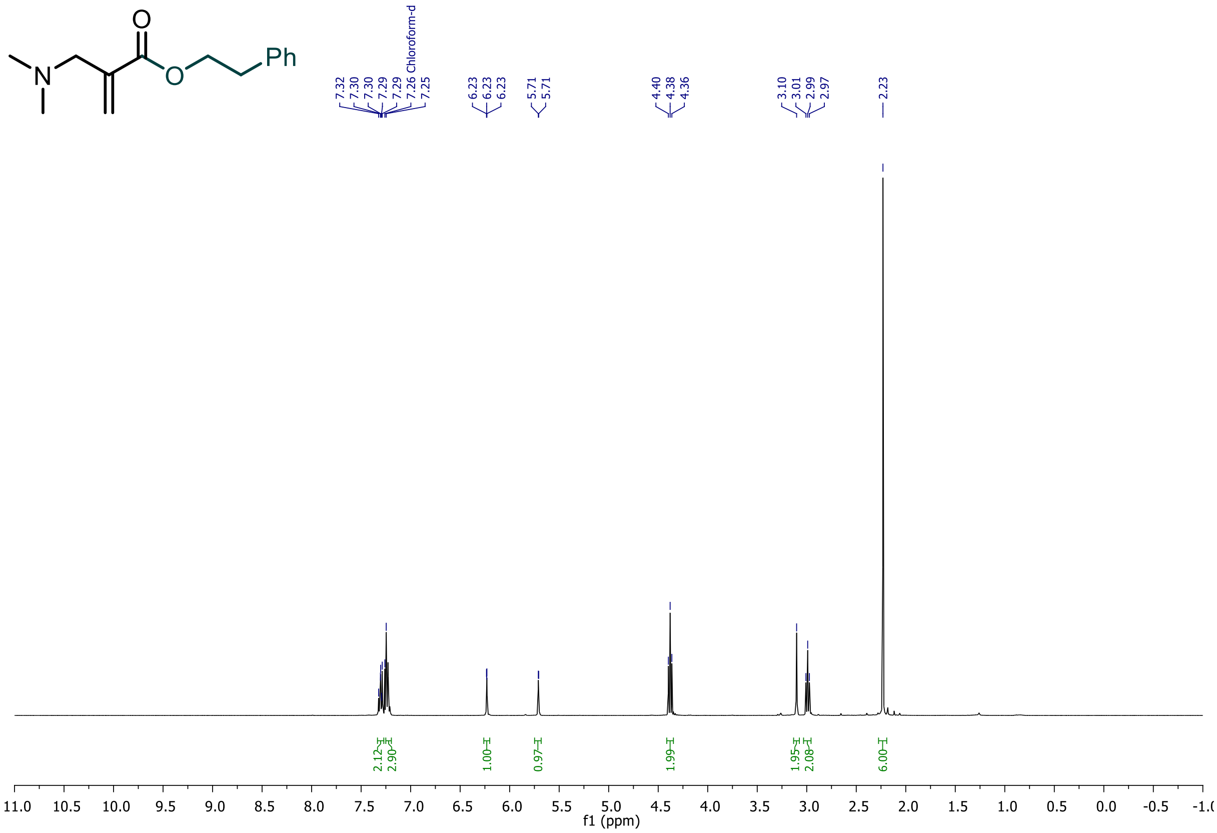

^{13}C NMR (150 MHz, $CDCl_3$)

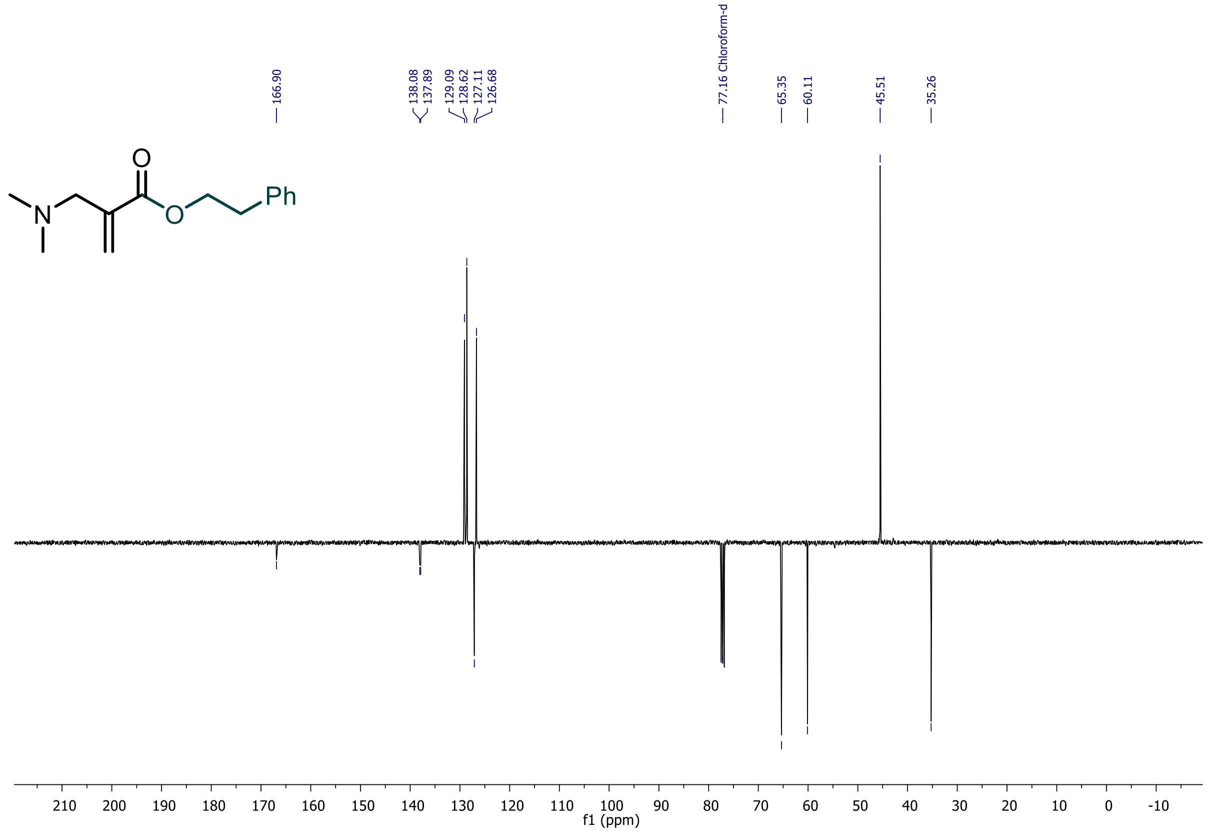

2a-d_2: Phenethyl 2-((dimethylamino)methyl)acrylate-d_2

^{1}H NMR (400 MHz, $CDCl_3$)

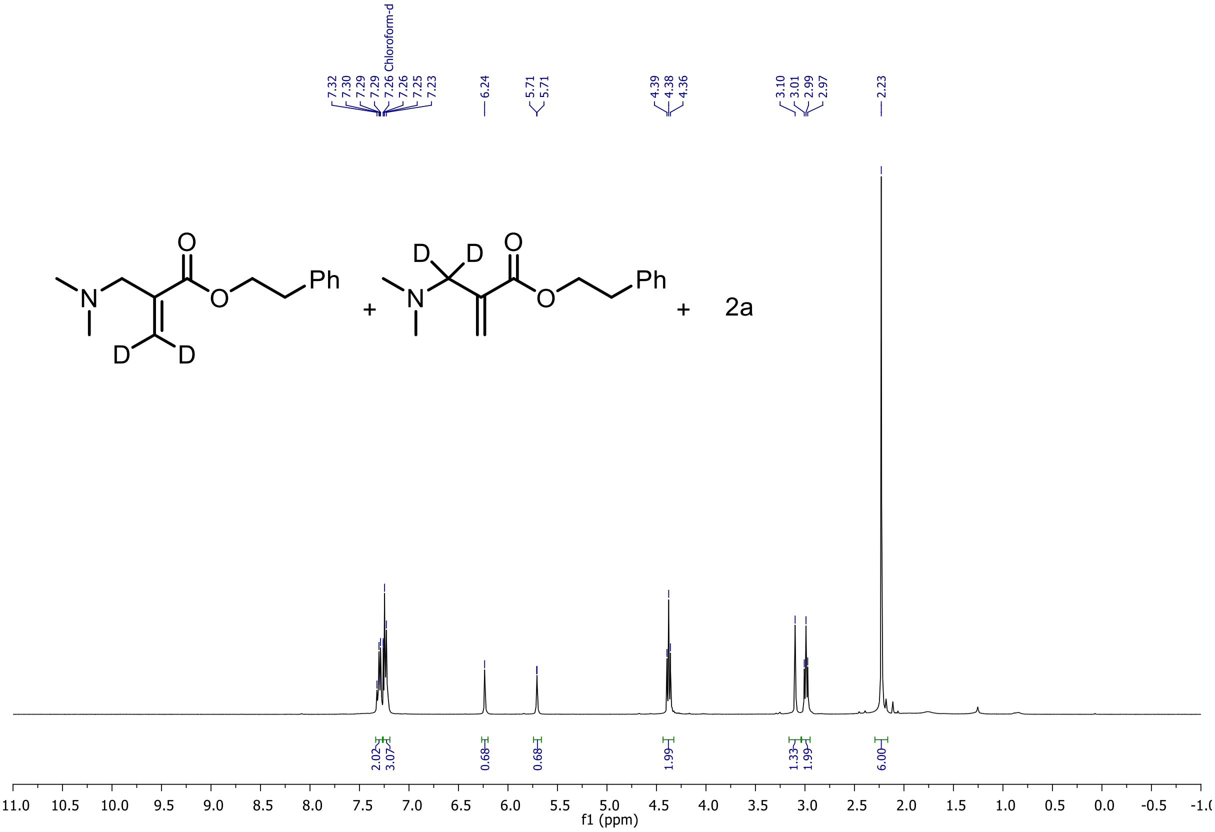

^{13}C NMR (150 MHz, $CDCl_3$)

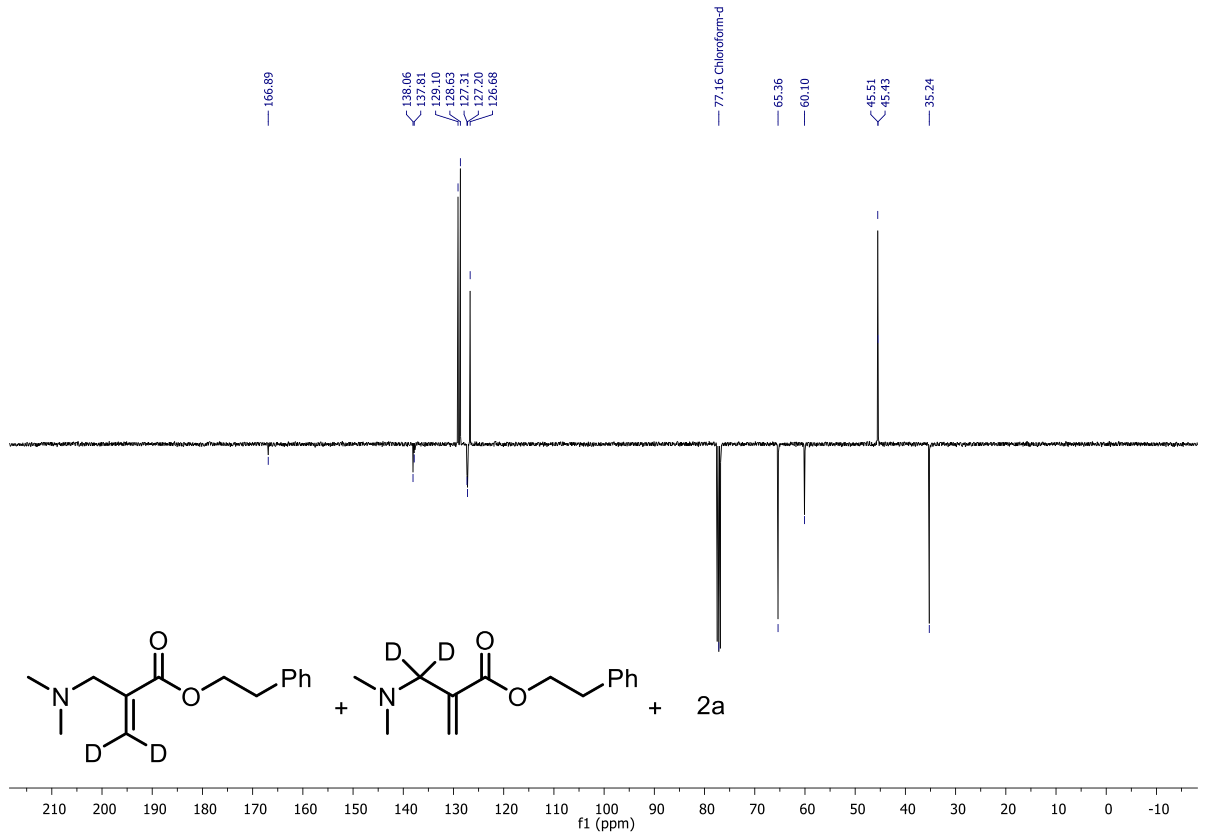

2b: 2,2-Diphenylethyl 2-((dimethylamino)methyl)acrylate

^{1}H NMR (400 MHz, $CDCl_3$)

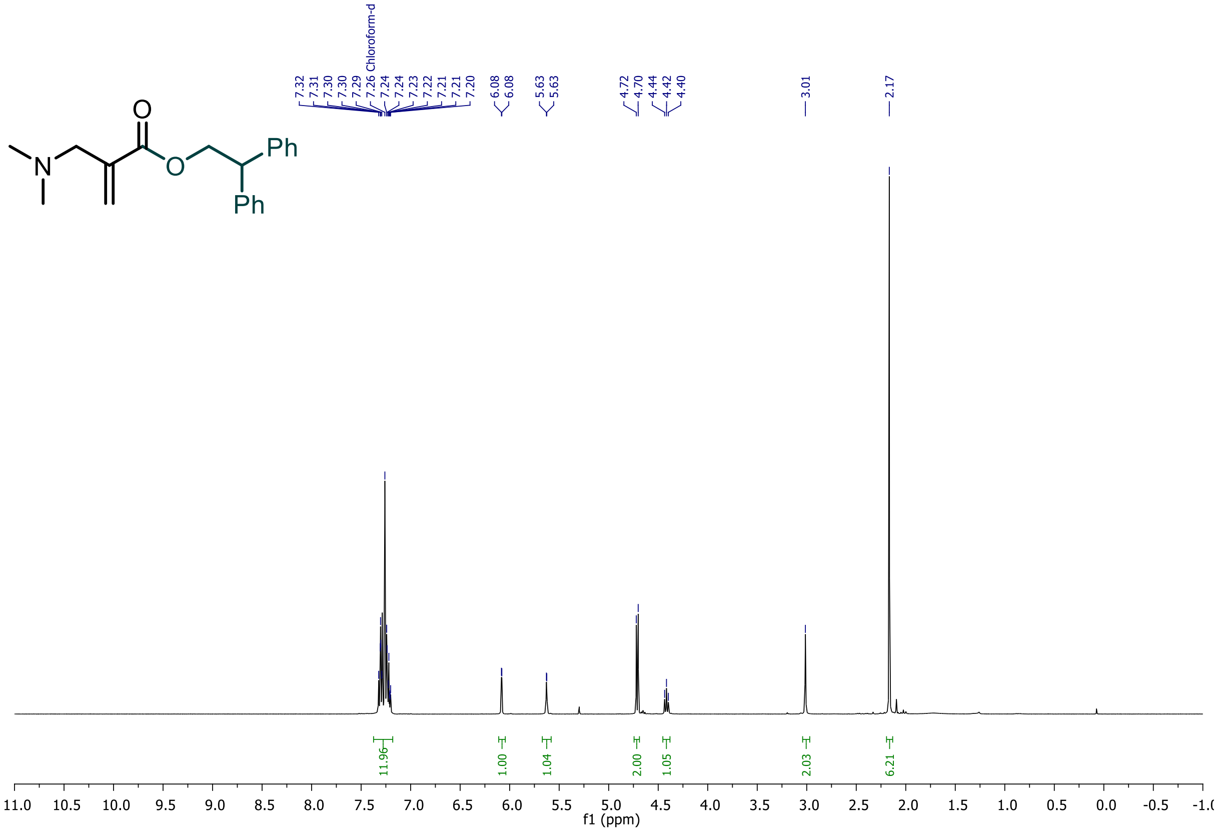

^{13}C NMR (150 MHz, $CDCl_3$)

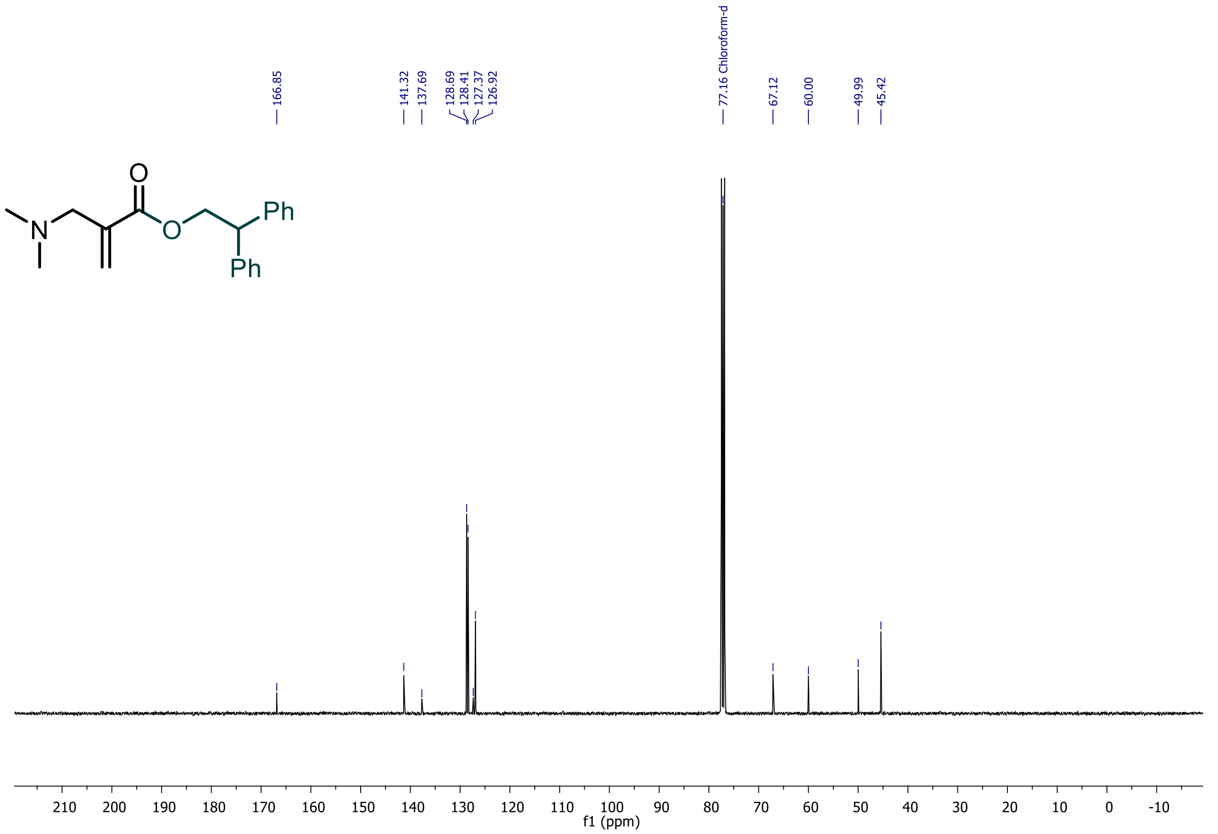

2c: 4-(4,4,5,5-Tetramethyl-1,3,2-dioxaborolan-2-yl)benzyl 2-((dimethylamino)methyl)acrylate

^{1}H NMR (500 MHz, $CDCl_3$)

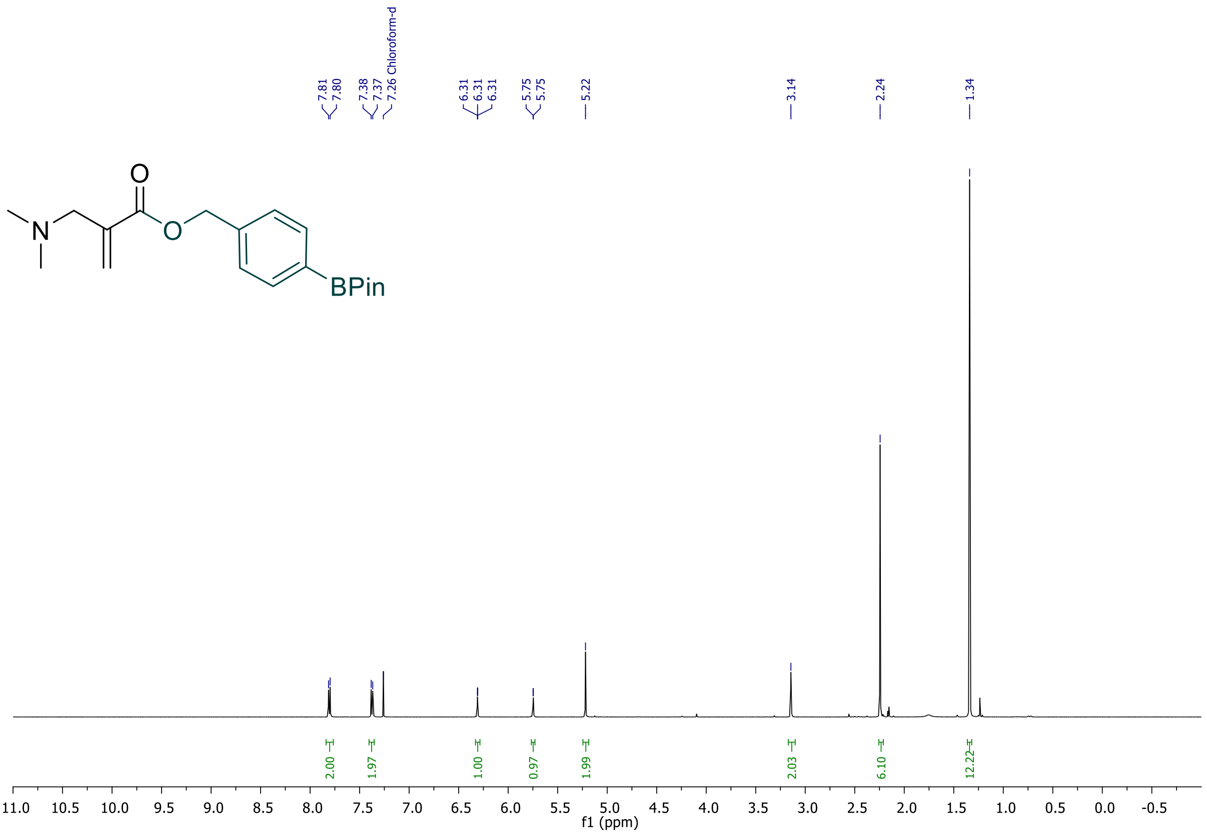

^{13}C NMR (126 MHz, $CDCl_3$)

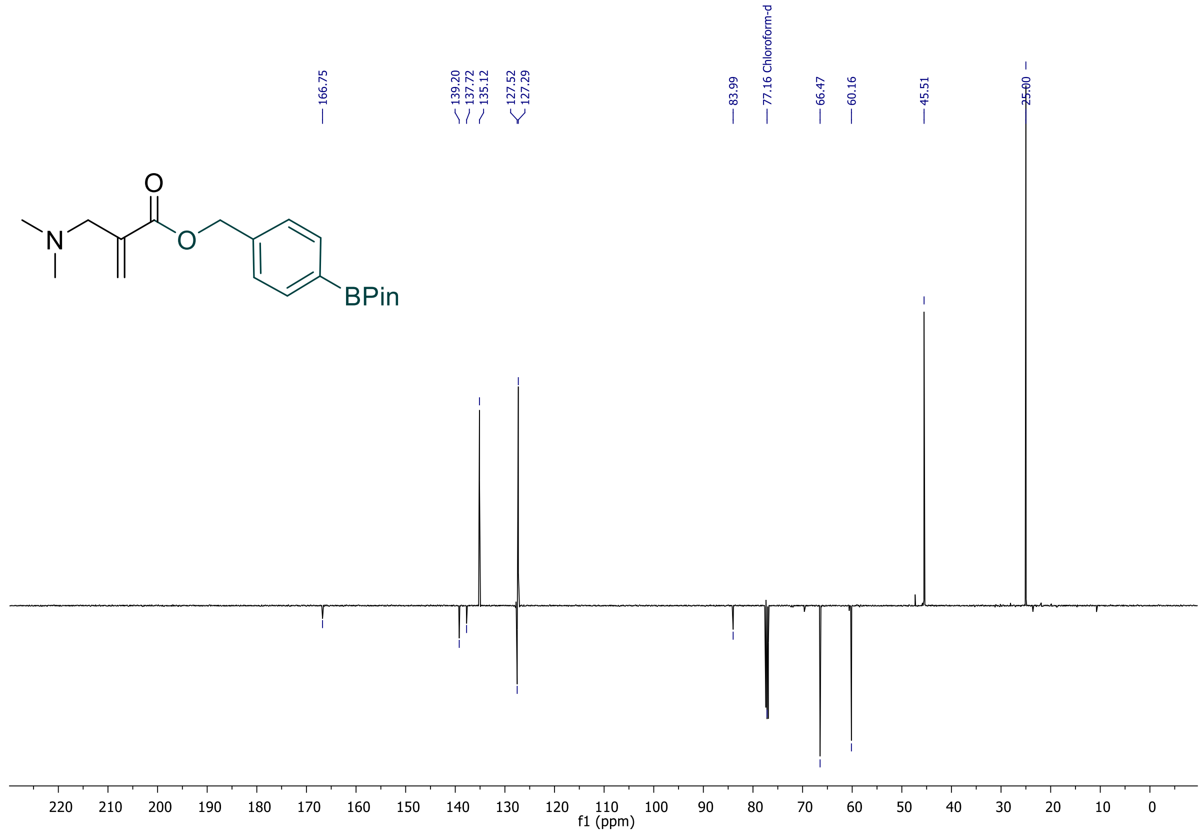

2d: (1-(Cyanomethyl)cyclopropyl)methyl 2-((dimethylamino)methyl)acrylate

^{1}H NMR (600 MHz, $CDCl_3$)

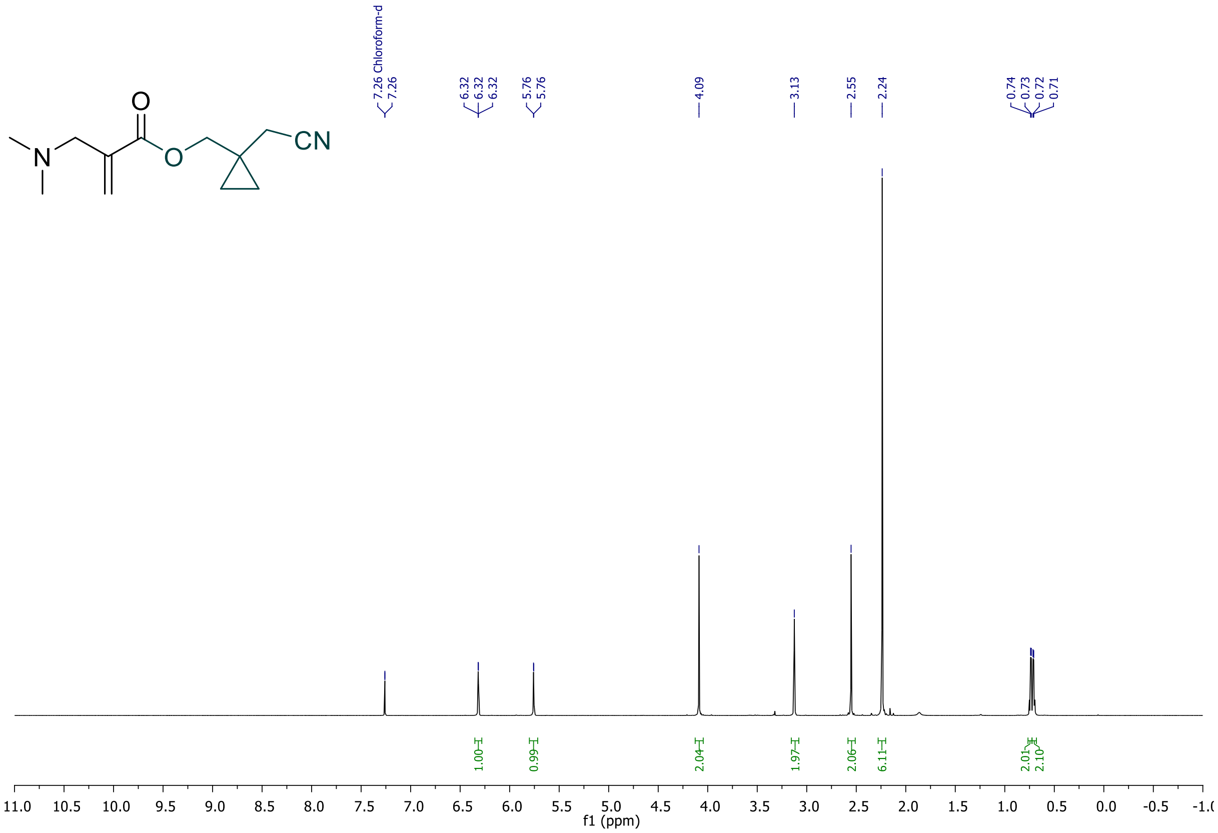

^{13}C NMR (151 MHz, $CDCl_3$)

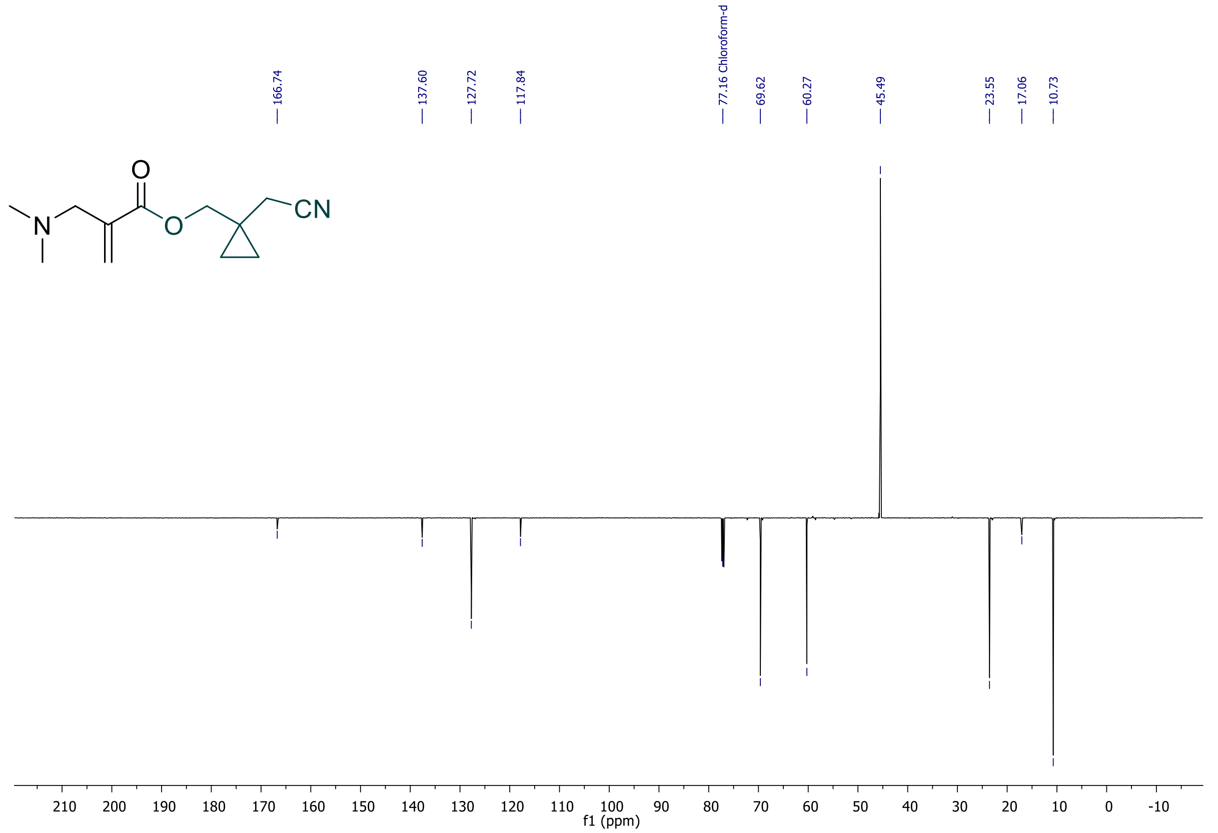

2e: (3a*R*,5*R*,6*S*,6a*R*)-5-((*S*)-2,2-Dimethyl-1,3-dioxolan-4-yl)-2,2-dimethyltetrahydrofuro[2,3-d][1,3]dioxol-6-yl 2-((dimethylamino)methyl)acrylate

^{1}H NMR (400 MHz, $CDCl_3$)

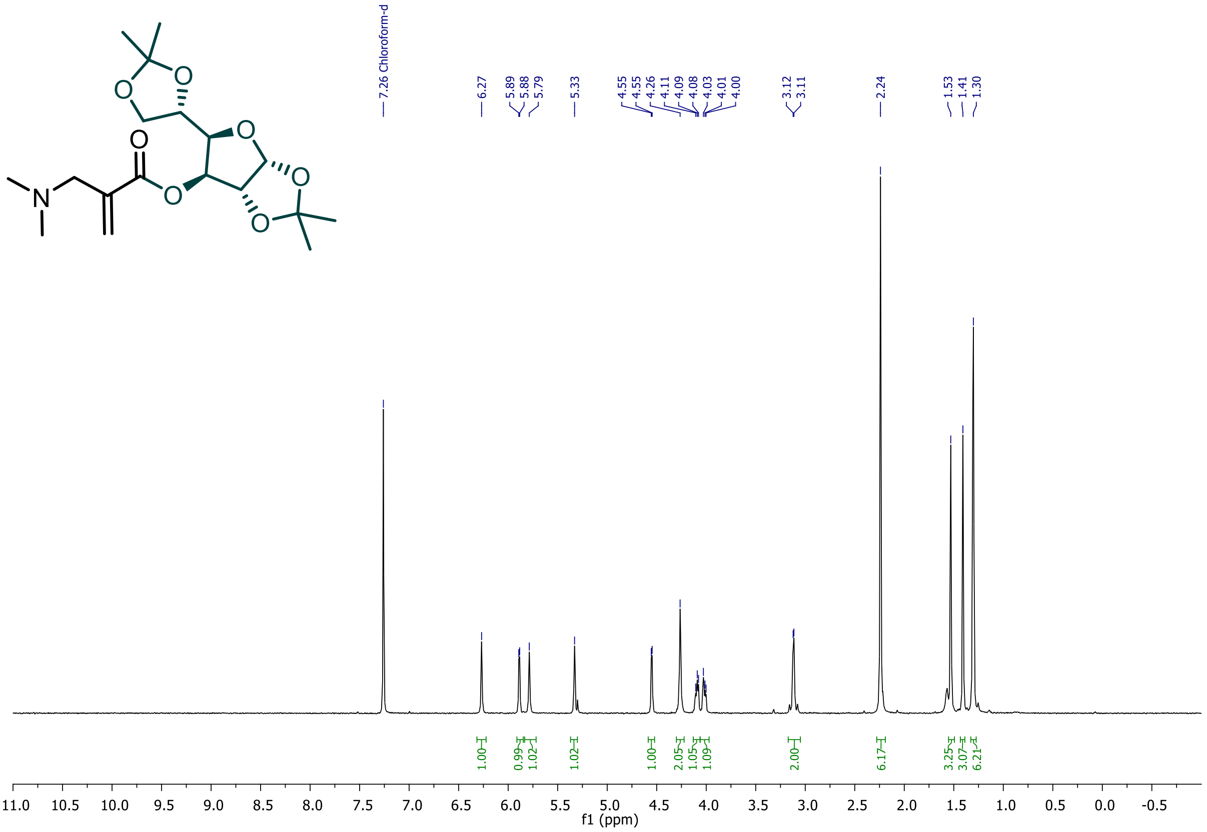

^{13}C NMR (151 MHz, $CDCl_3$)

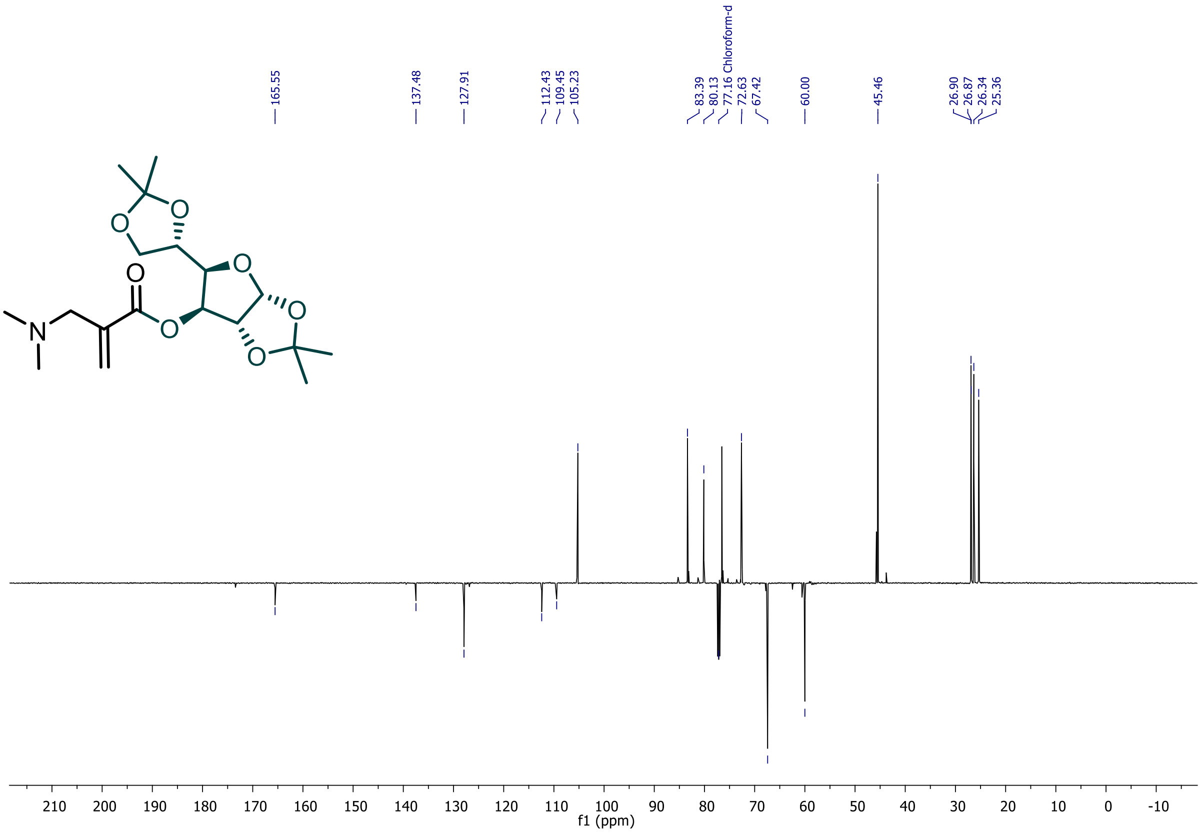

2f: 3-(1,3-Dioxoisoindolin-2-yl)propyl 2-((dimethylamino)methyl)acrylate

^{1}H NMR (400 MHz, $CDCl_3$)

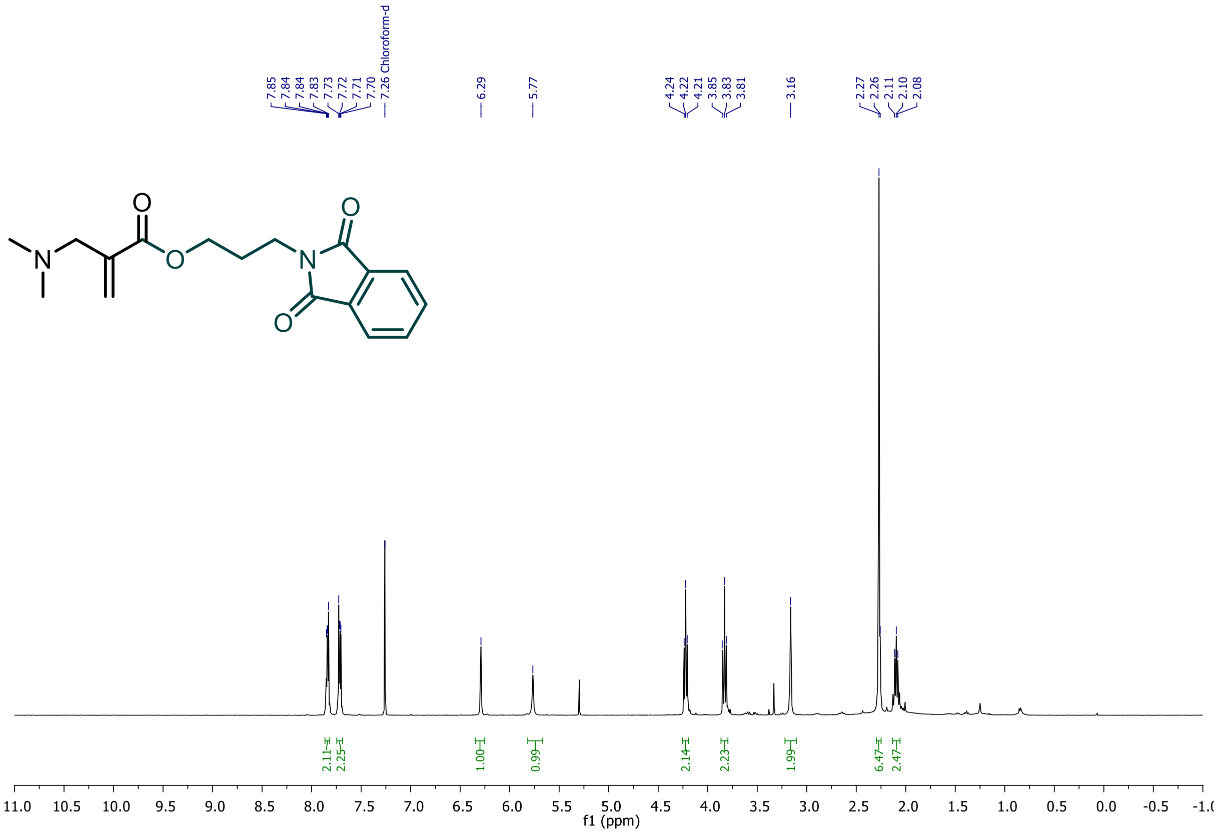

^{13}C NMR (100 MHz, $CDCl_3$)

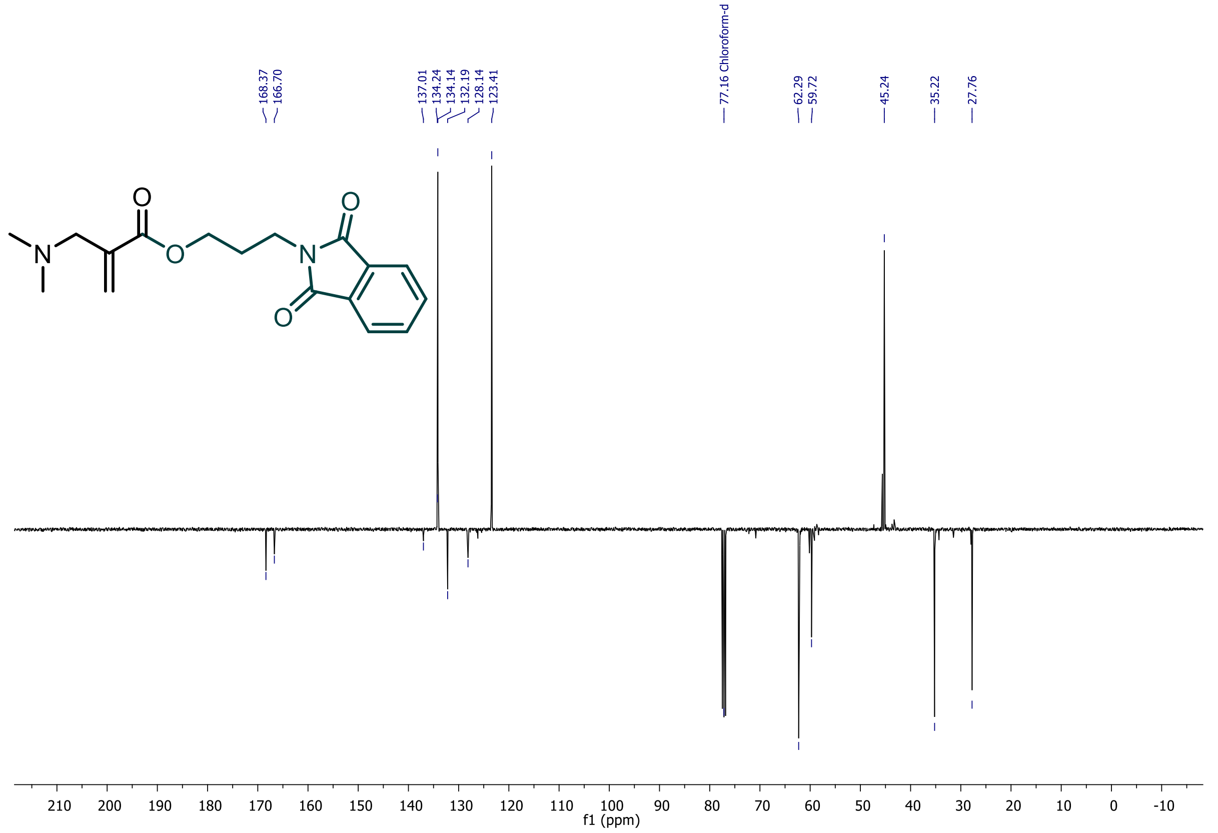

2g: (*S*)-(6-Methoxyquinolin-4-yl)((1S,2R,4S,5R)-5-vinylquinuclidin-2-yl)methyl 2-((dimethylamino)methyl)acrylate

^{1}H NMR (400 MHz, $CDCl_3$)

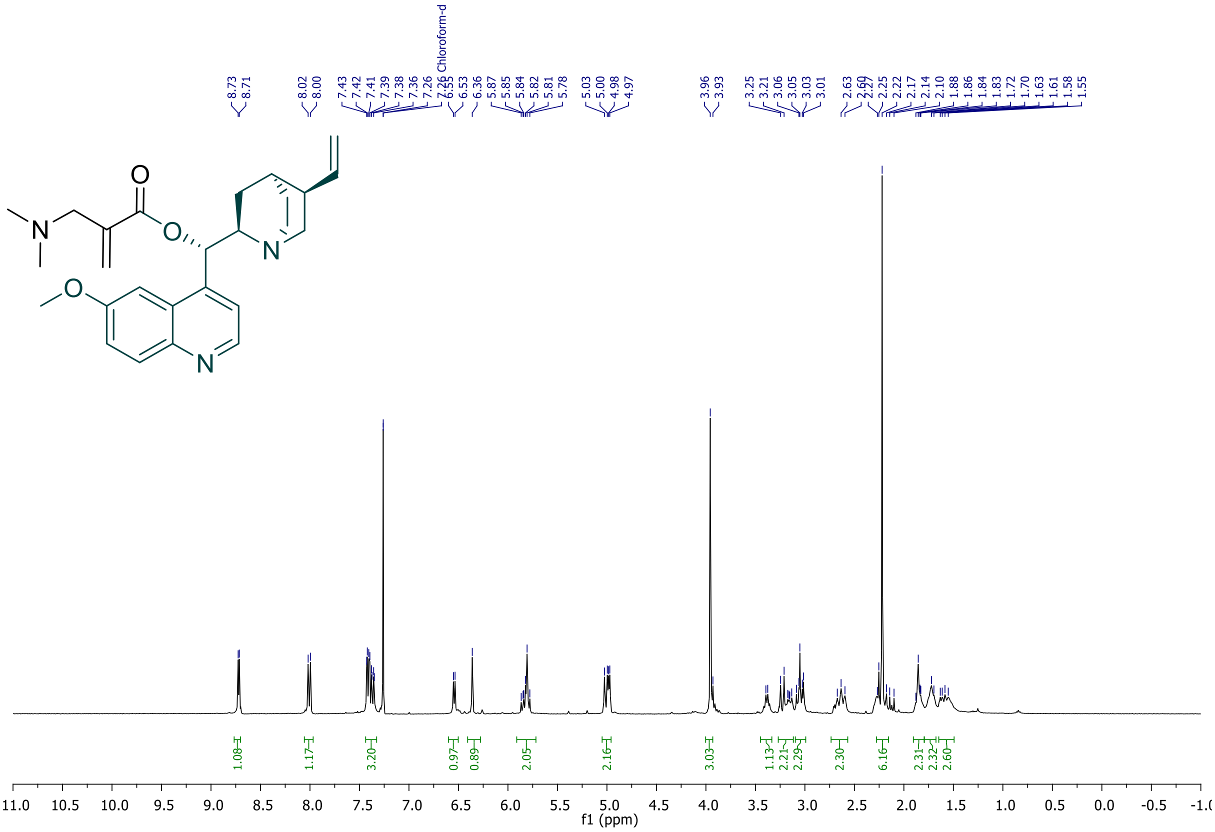

^{13}C NMR (151 MHz, $CDCl_3$)

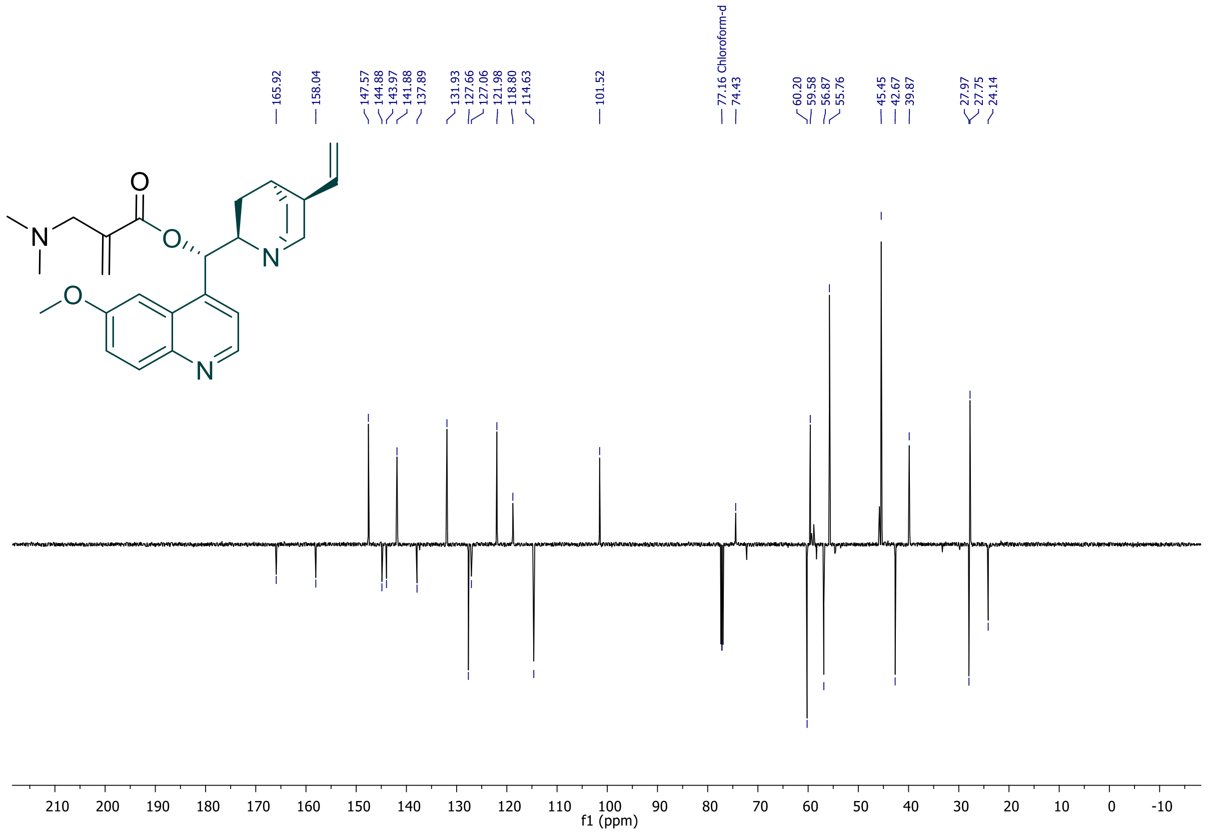

2h: (1*R*,2*S*,5*R*)-2-Isopropyl-5-methylcyclohexyl 2-((dimethylamino)methyl)acrylate

^{1}H NMR (600 MHz, $CDCl_3$)

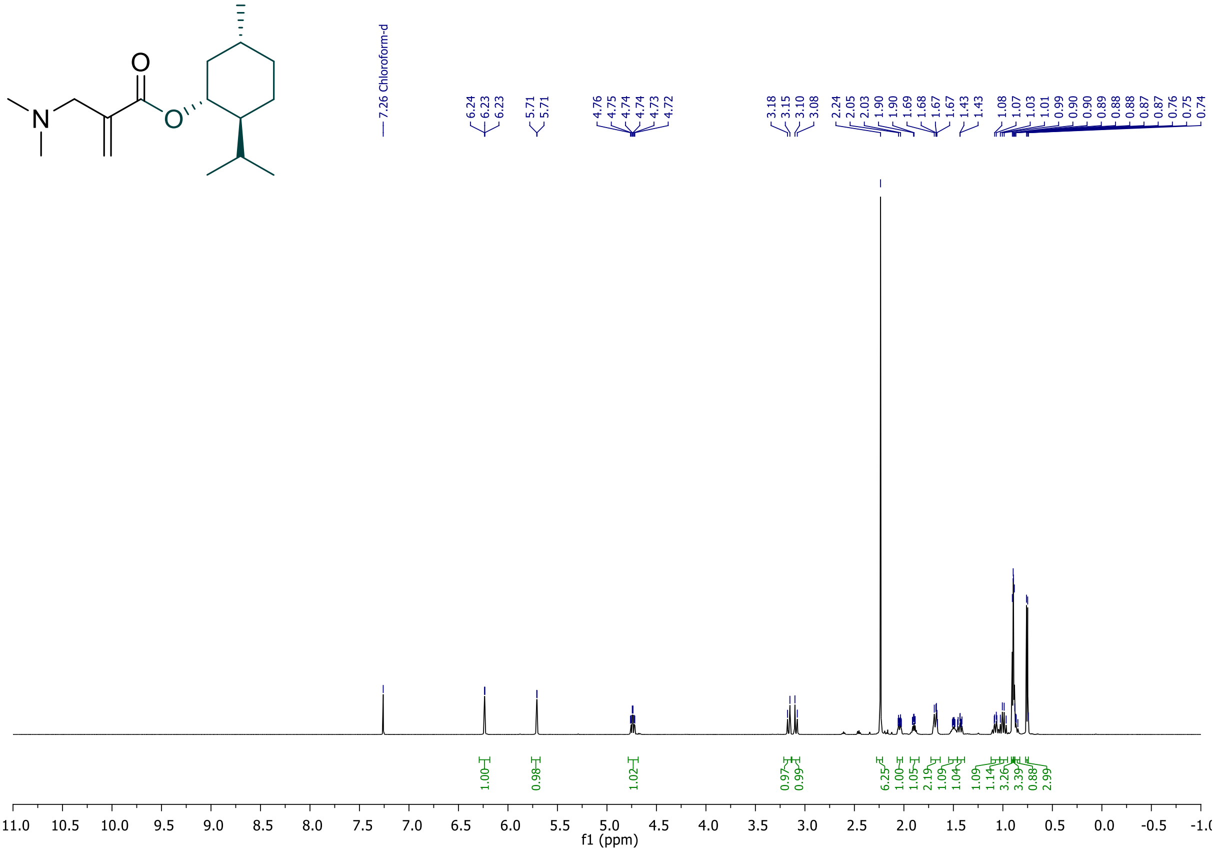

^{13}C NMR (151 MHz, $CDCl_3$)

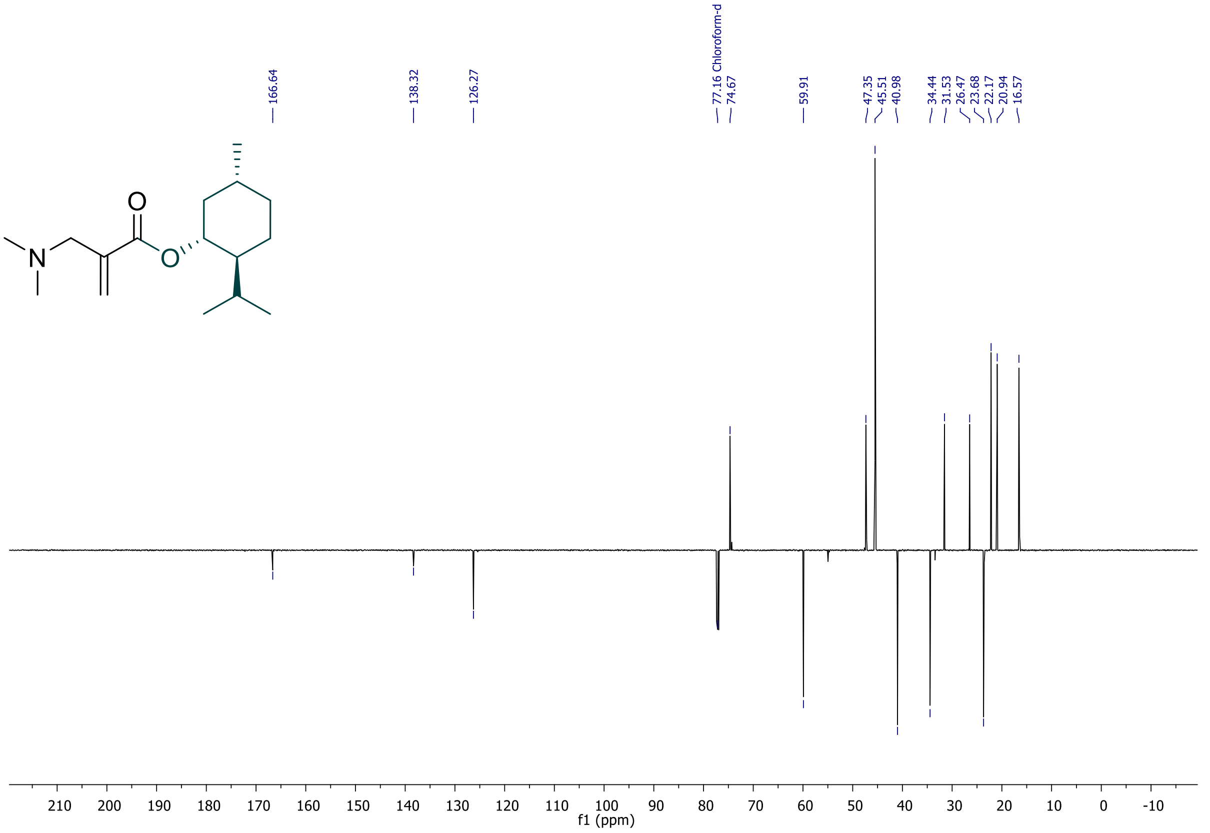

2i: *S*-Octyl 2-((dimethylamino)methyl)prop-2-enethioate

^{1}H NMR (400 MHz, CDCl$_3$)

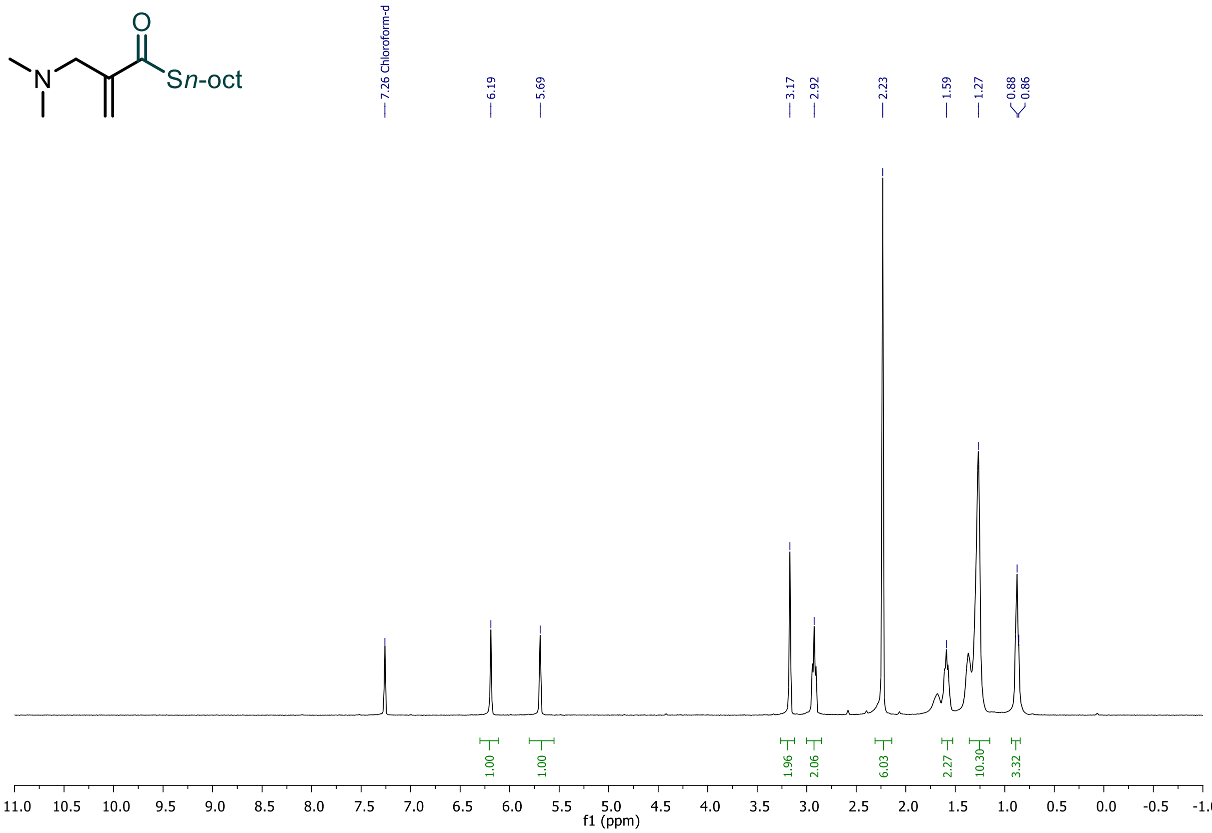

^{13}C NMR (100 MHz, CDCl$_3$)

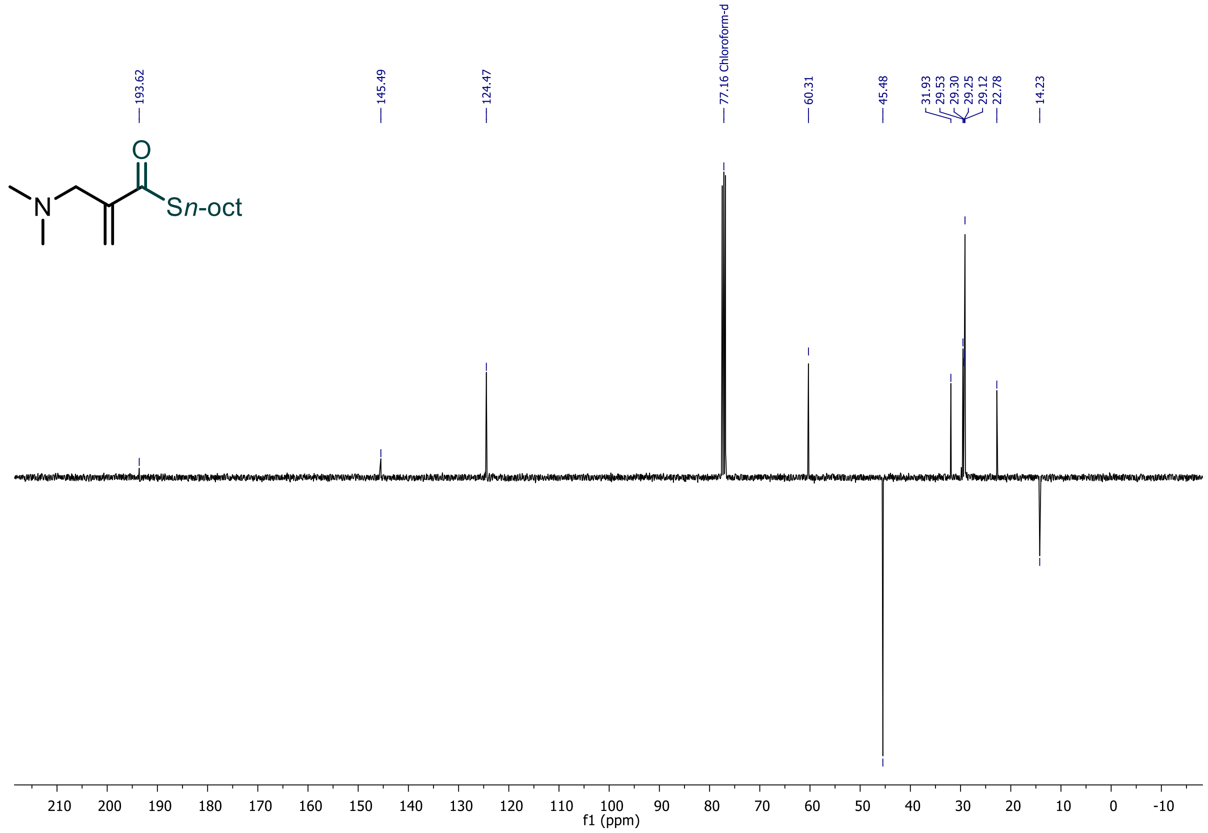

2j: *N*,*N*-Dimethyl-2-(phenylsulfonyl)prop-2-en-1-amine

^{1}H NMR (600 MHz, $CDCl_3$)

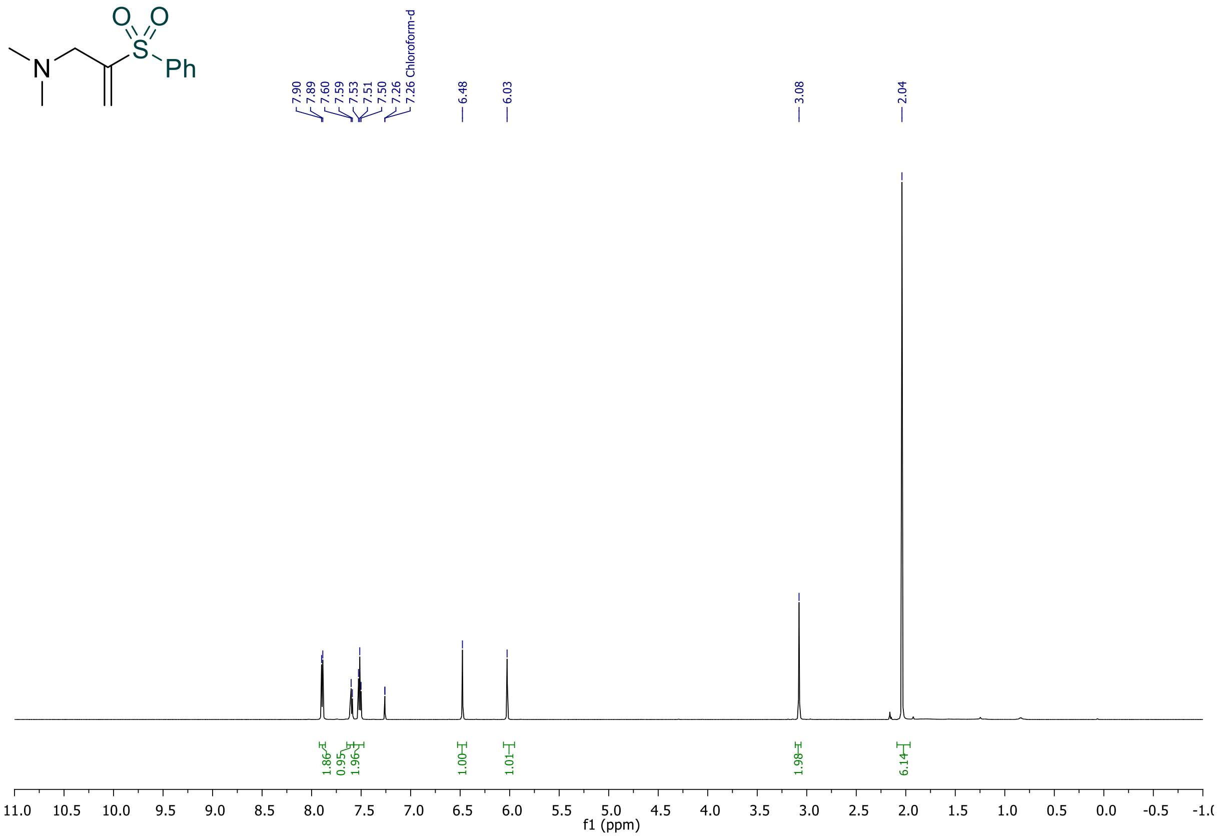

^{13}C NMR (151 MHz, $CDCl_3$)

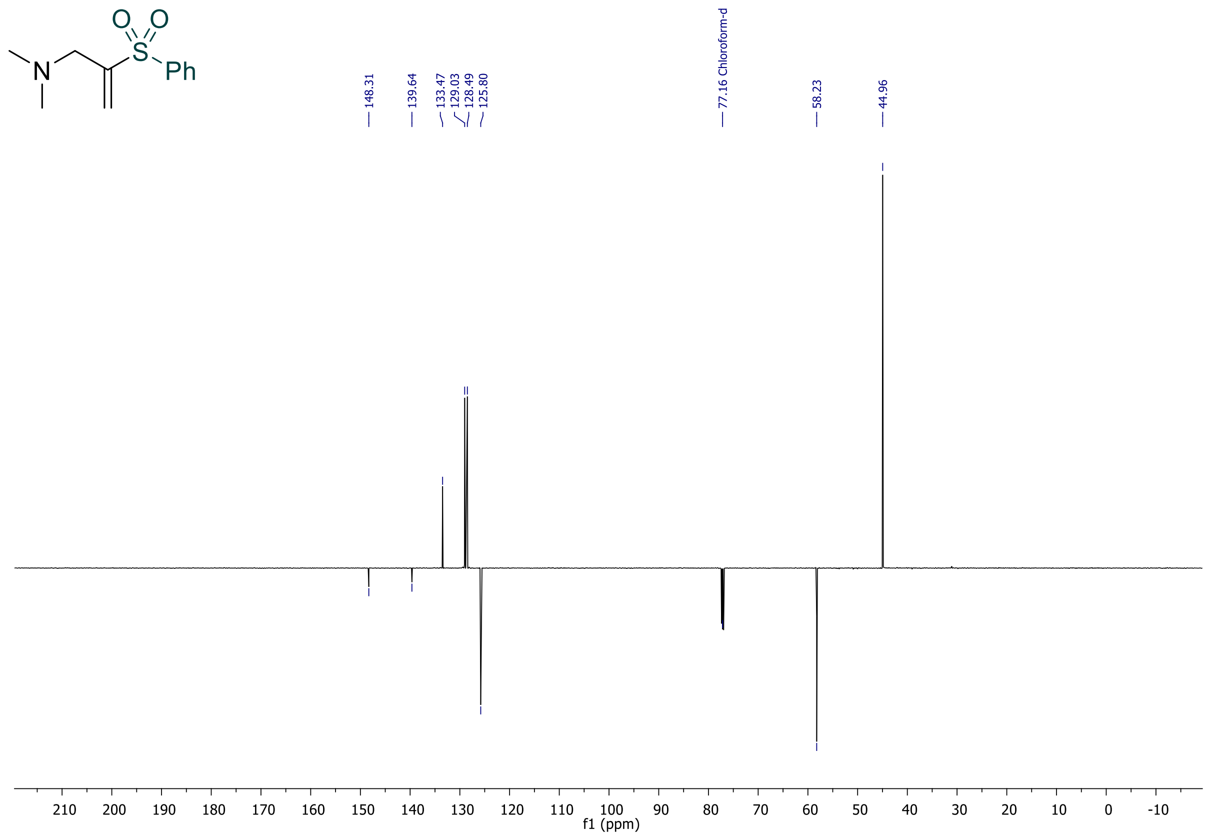

3a: 2-((Dimethylamino)methyl)-1-(4-methoxyphenyl)prop-2-en-1-one

^{1}H NMR (400 MHz, $CDCl_3$)

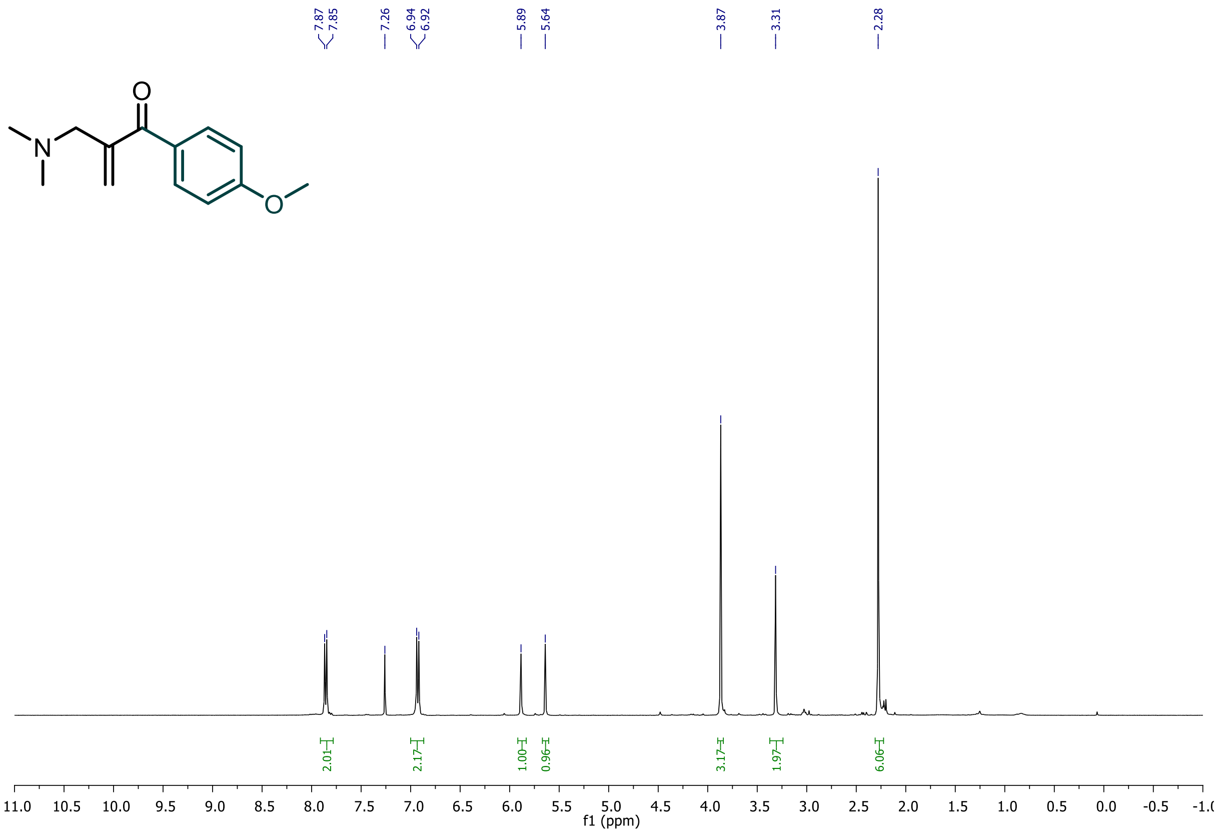

^{13}C NMR (100 MHz, $CDCl_3$)

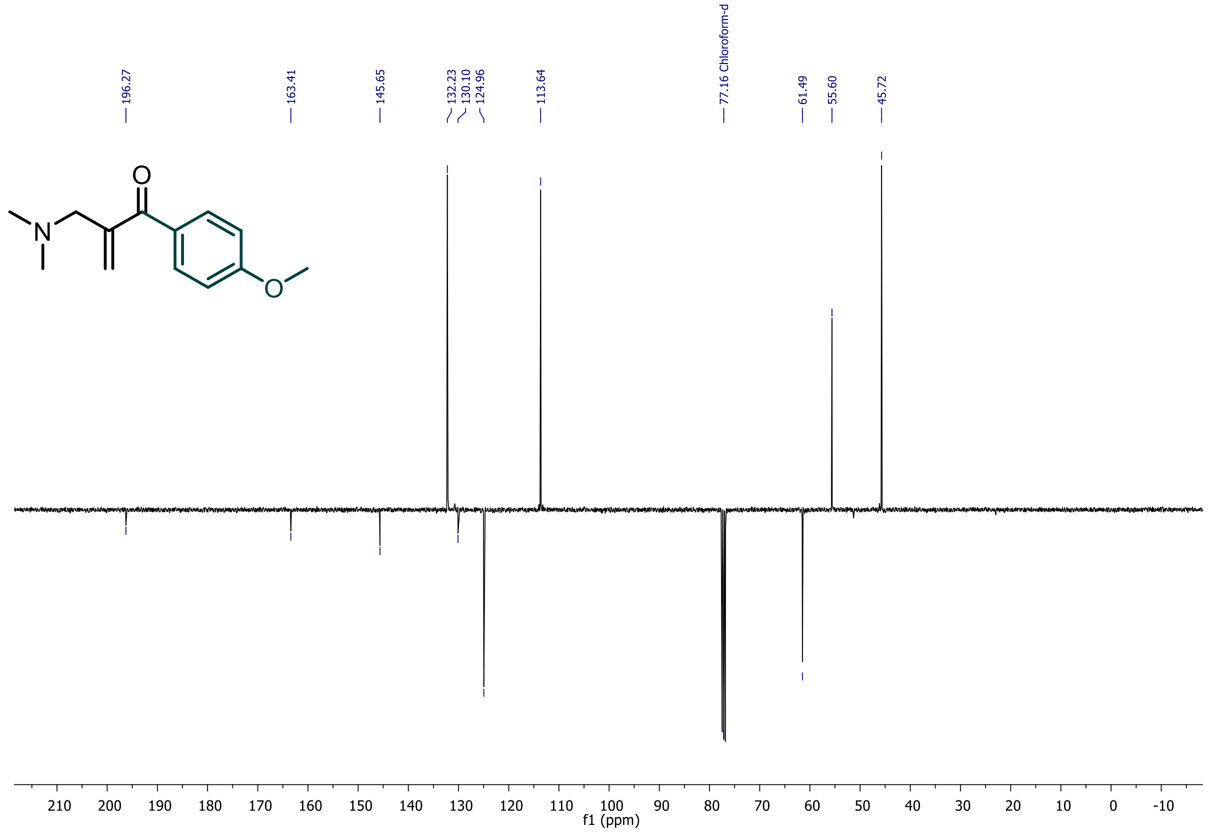

3b: 1-(4-(*tert*-Butyl)phenyl)-2-((dimethylamino)methyl)prop-2-en-1-one

^{1}H NMR (600 MHz, $CDCl_3$)

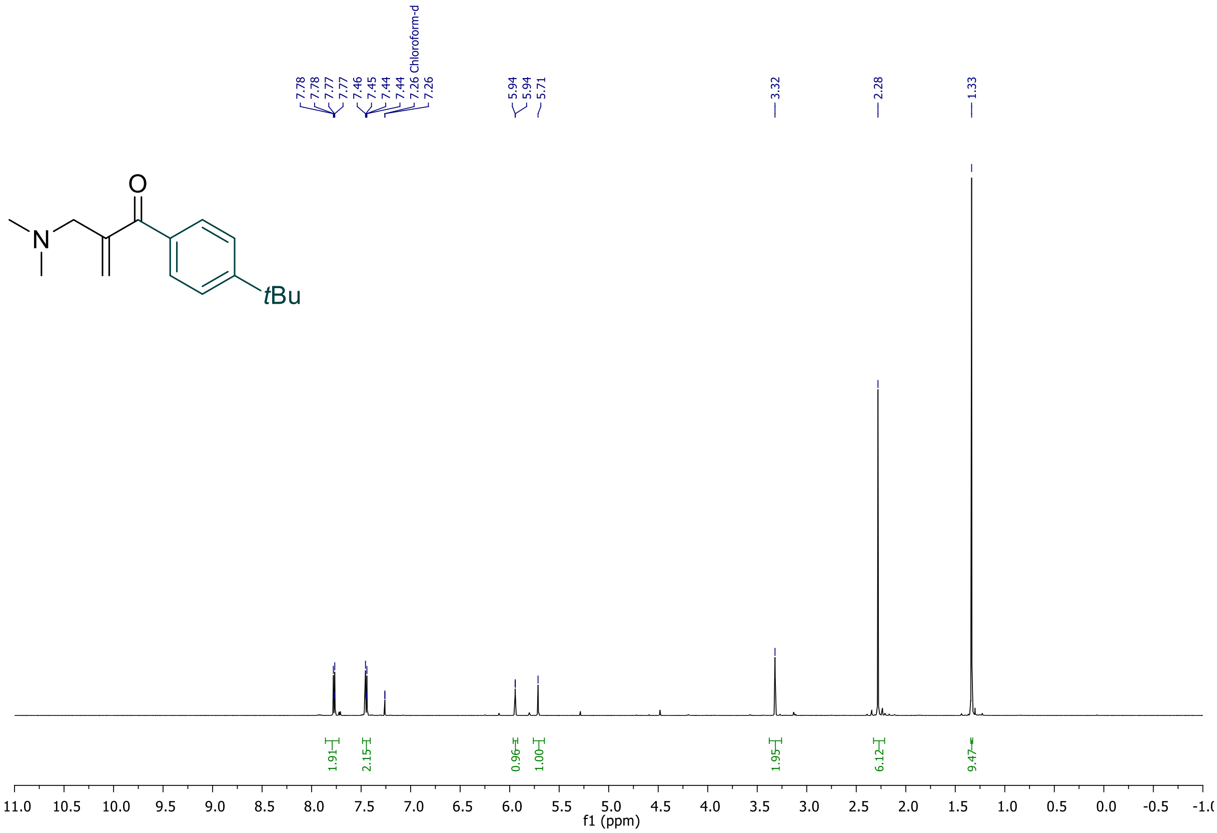

^{13}C NMR (151 MHz, $CDCl_3$)

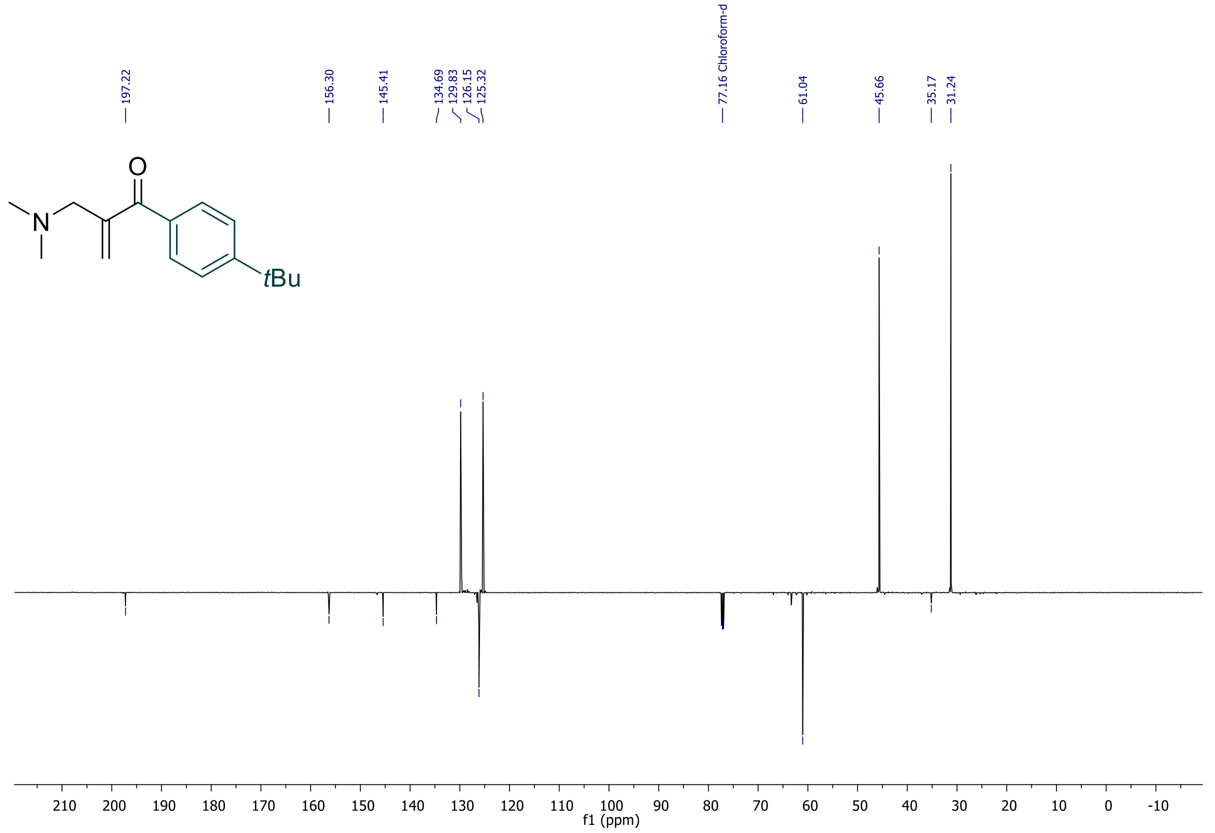

3c: 2-((Dimethylamino)methyl)-1-(4-fluorophenyl)prop-2-en-1-one

^{1}H NMR (600 MHz, $CDCl_3$)

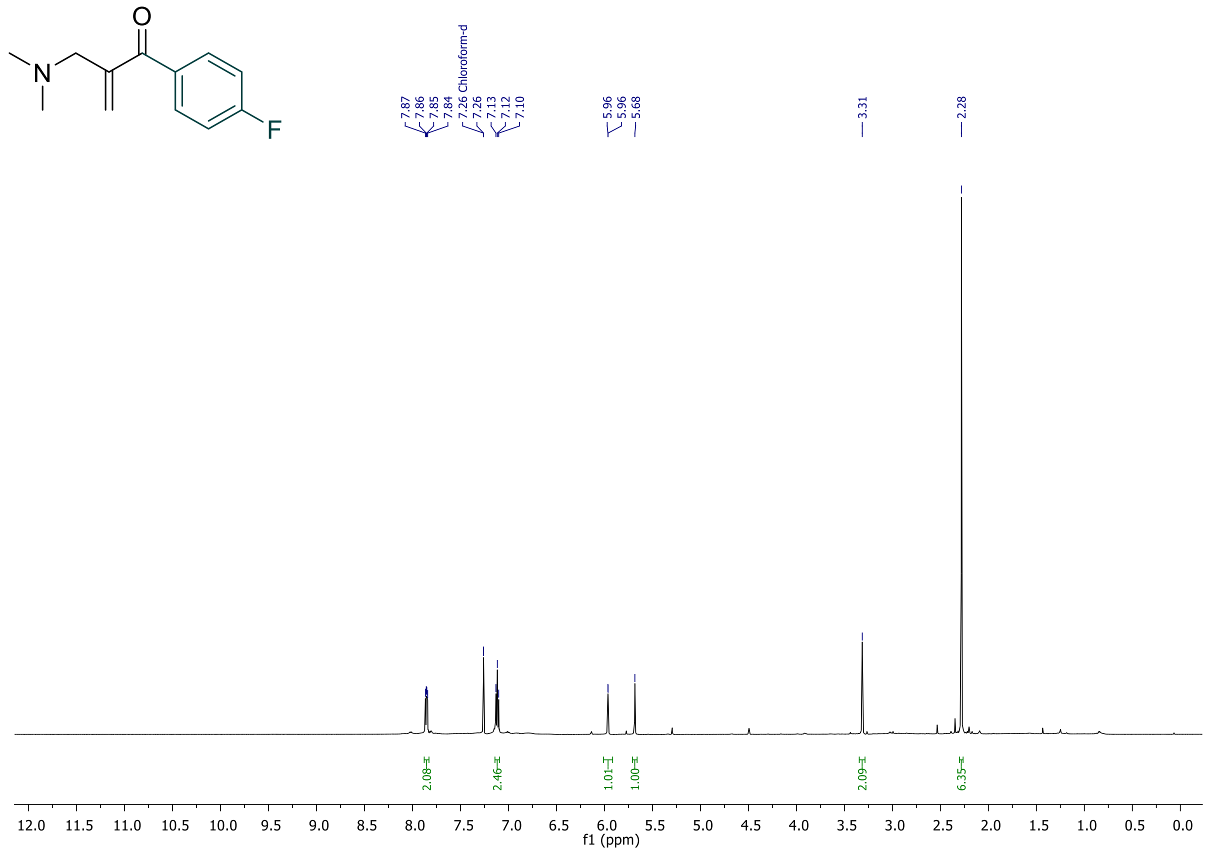

^{13}C NMR (151 MHz, $CDCl_3$)

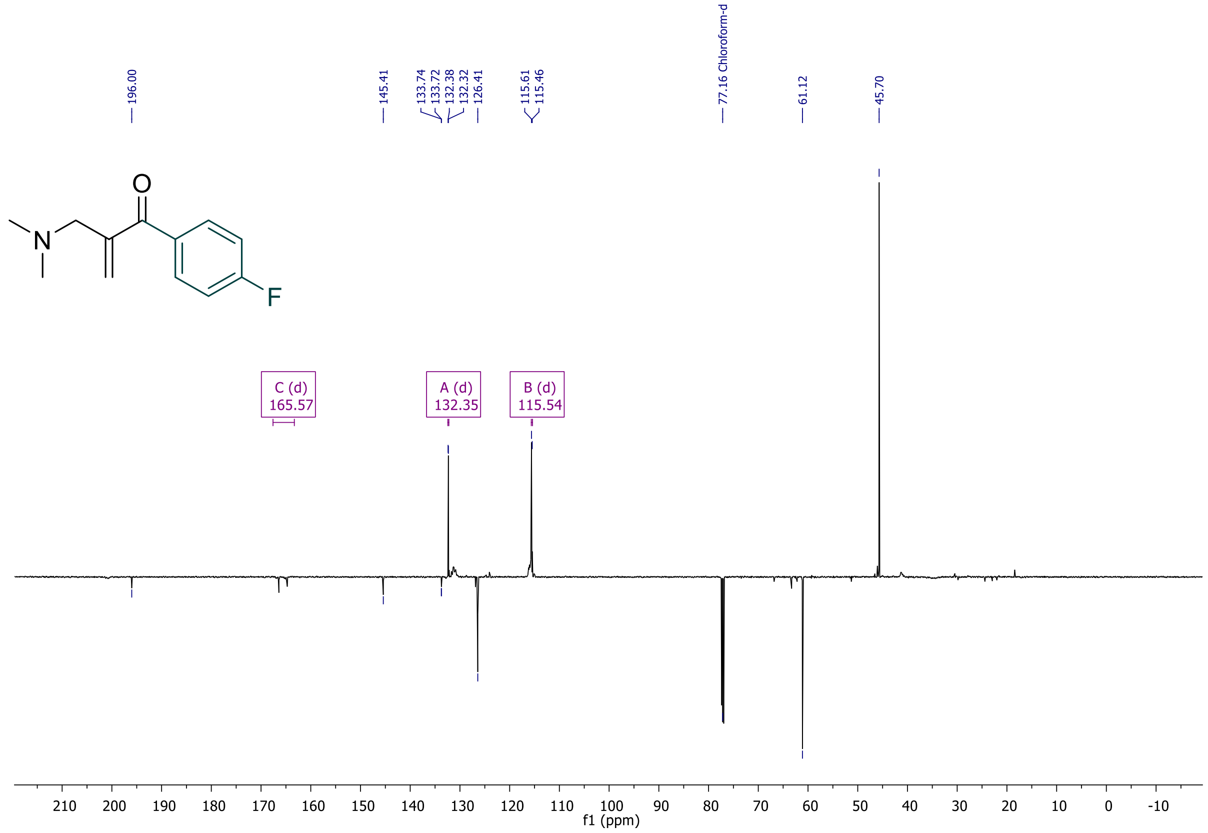

^{19}F NMR (565 MHz, $CDCl_3$)

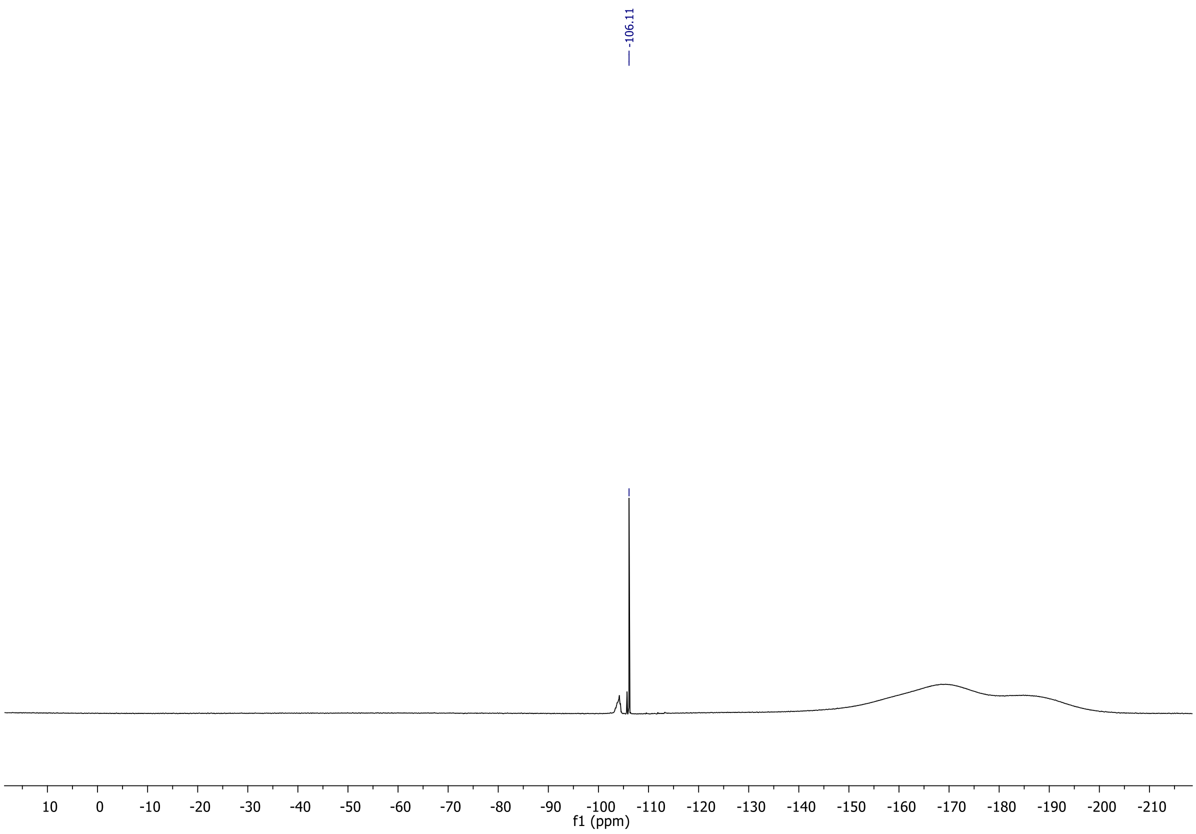

3d: 2-((Dimethylamino)methyl)-1-(4-(trifluoromethyl)phenyl)prop-2-en-1-one

^{1}H NMR (600 MHz, $CDCl_3$)

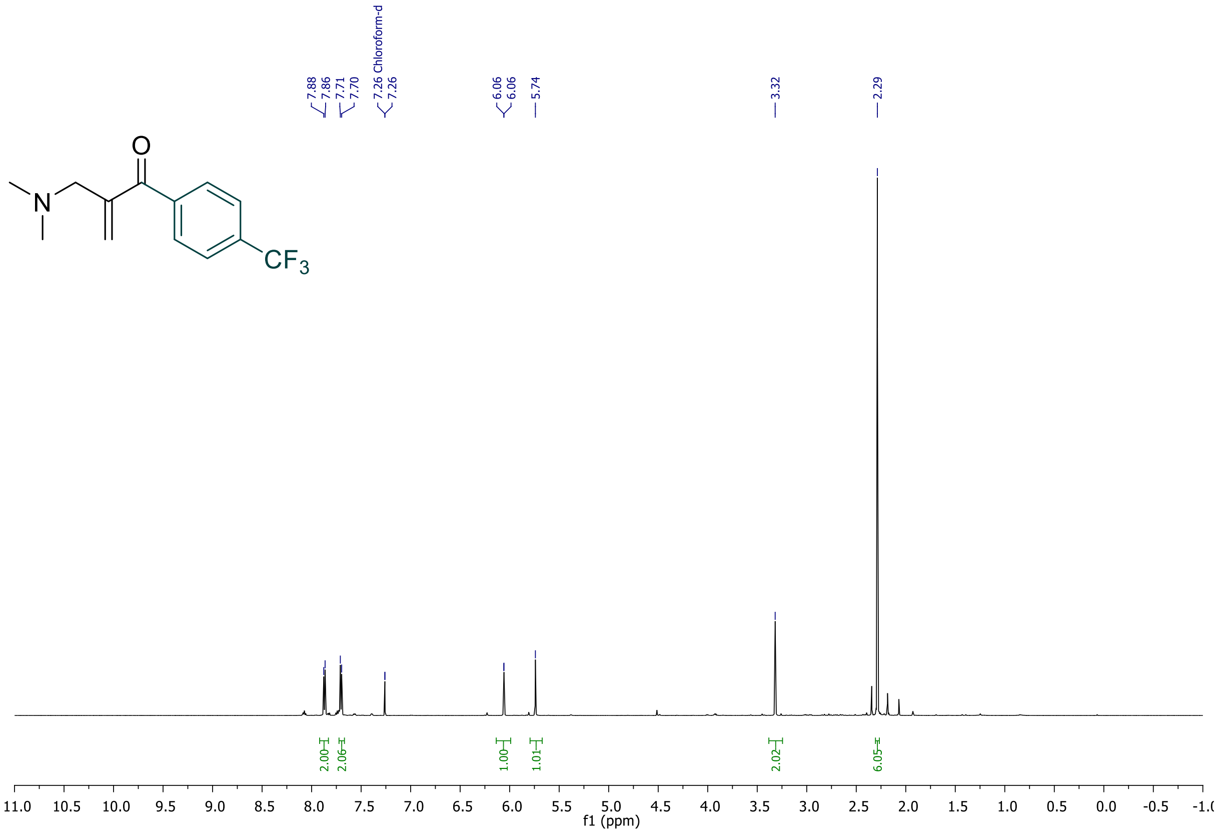

^{13}C NMR (151 MHz, $CDCl_3$)

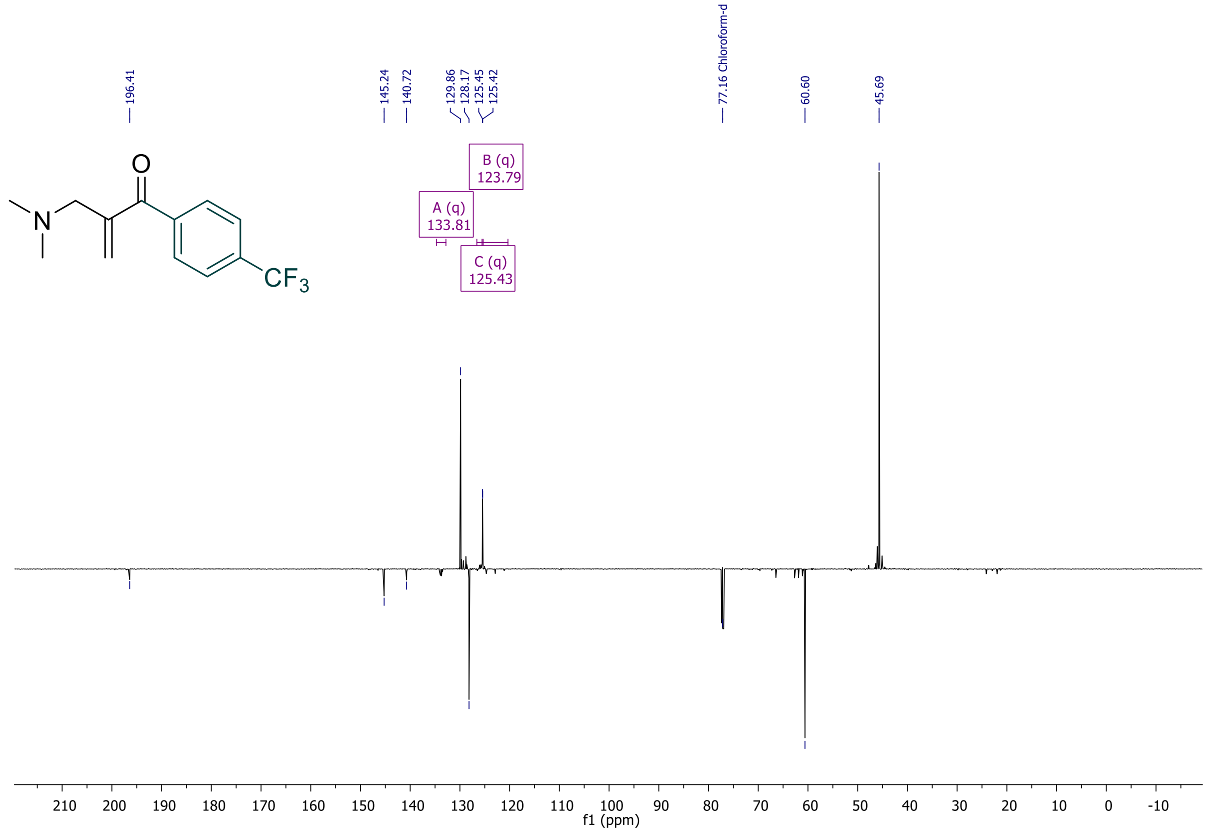

^{19}F NMR (377 MHz, $CDCl_3$)

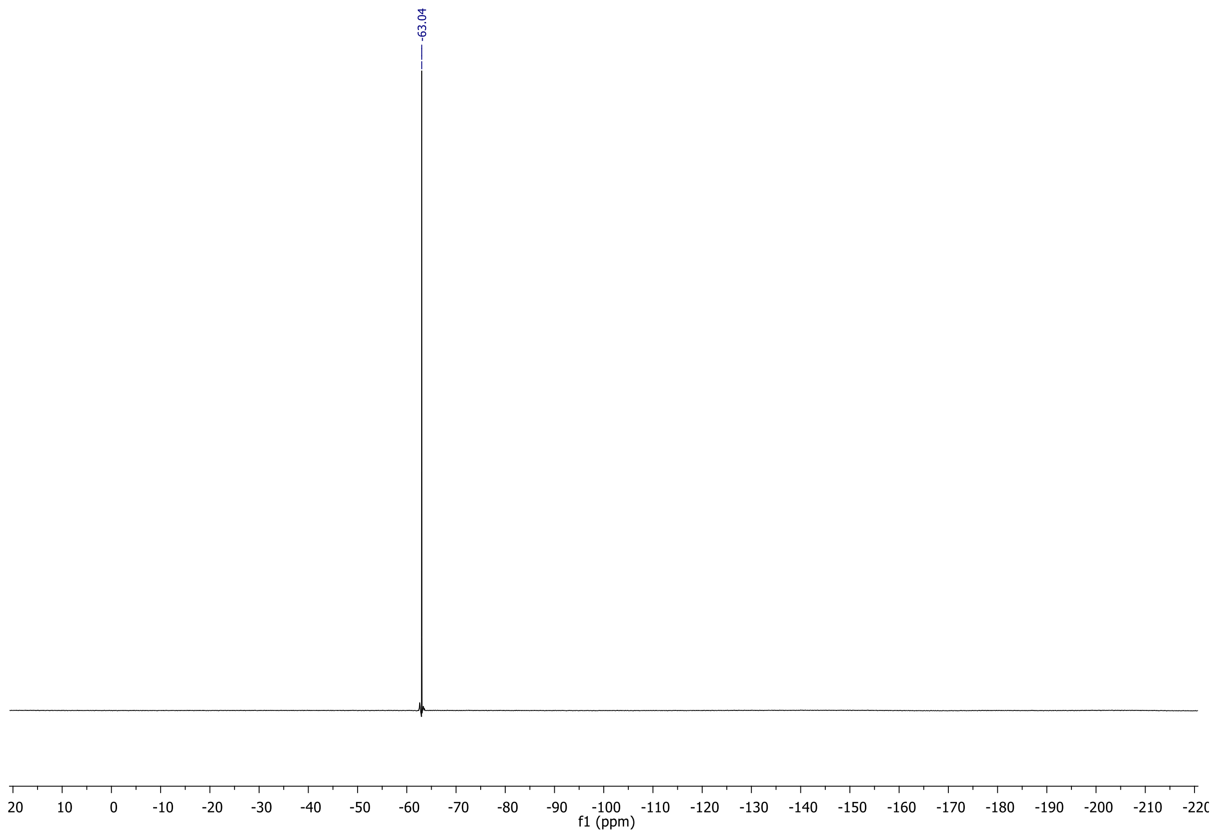

3e: 1-(Benzo[d][1,3]dioxol-5-yl)-2-((dimethylamino)methyl)prop-2-en-1-one

^{1}H NMR (600 MHz, CDCl$_3$)

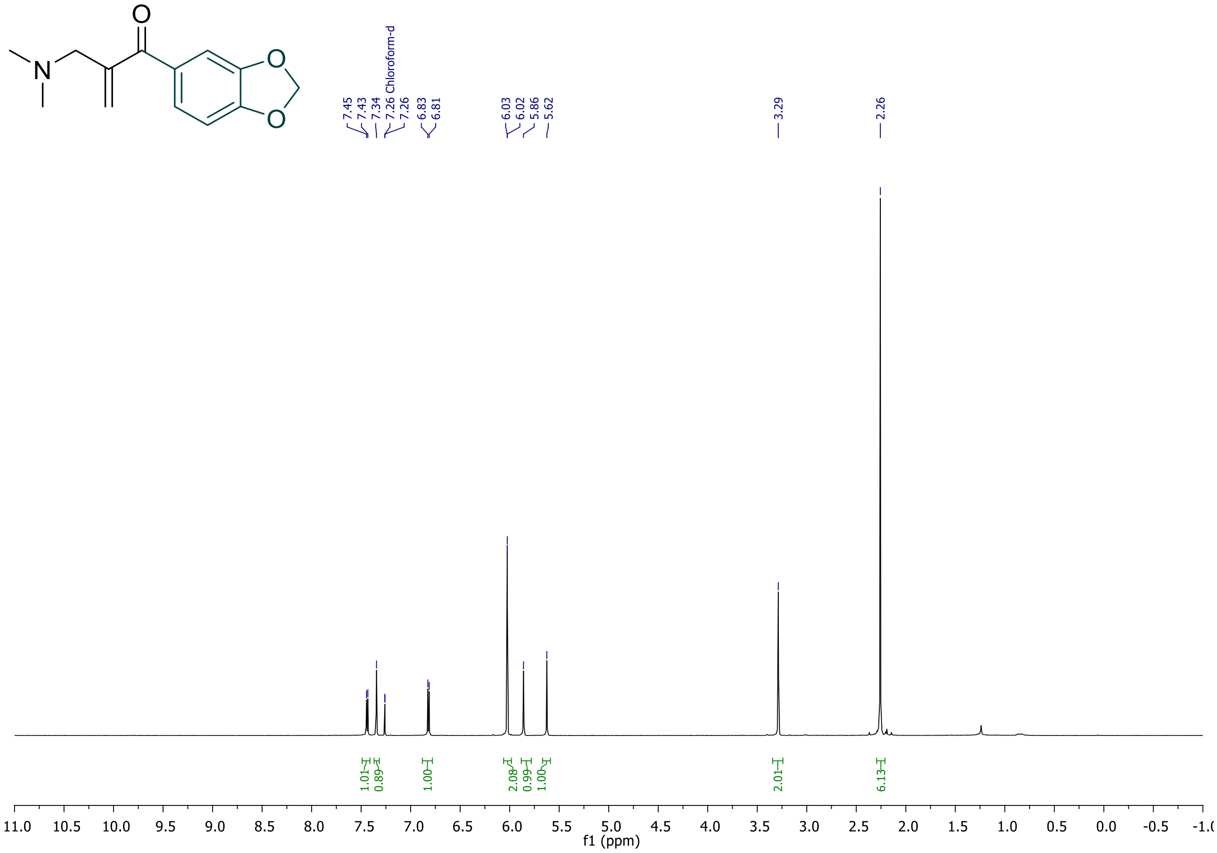

^{13}C NMR (151 MHz, CDCl$_3$)

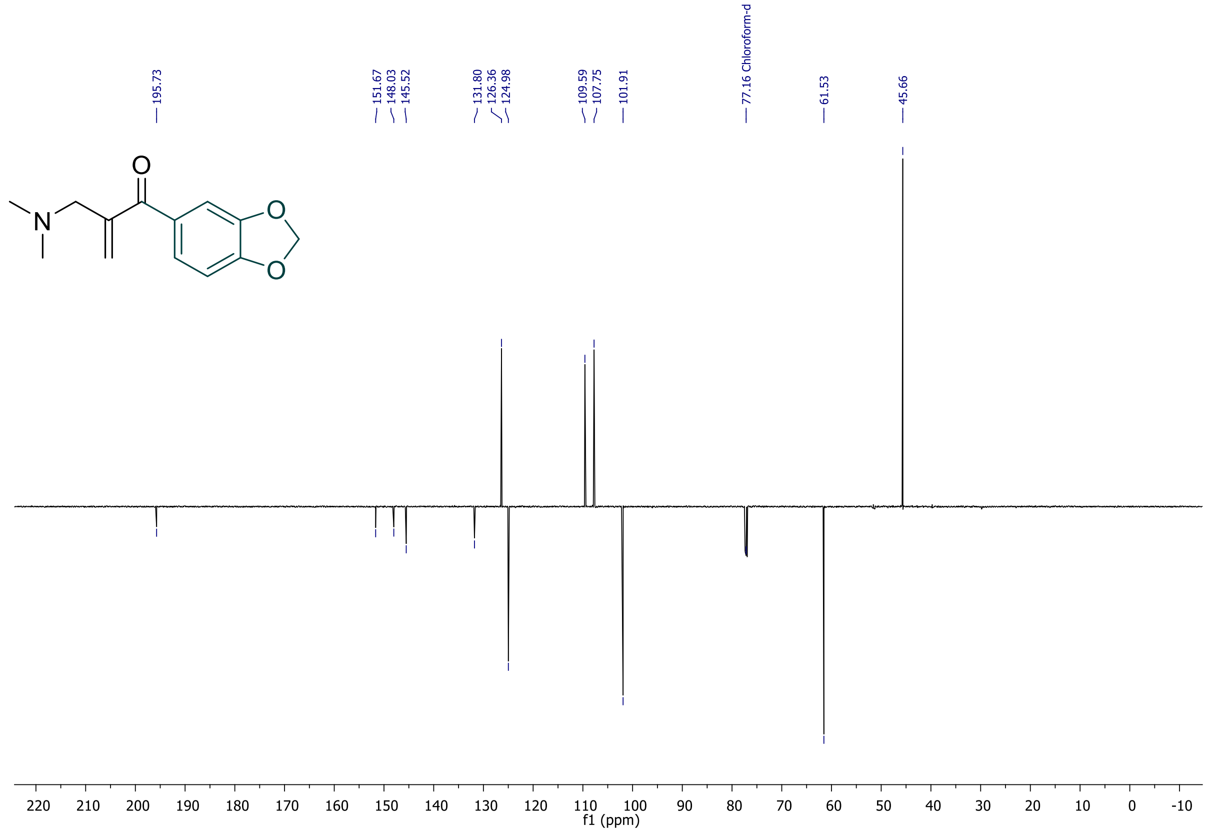

3f: 2-((Dimethylamino)methyl)-1-(3,5-dimethylphenyl)prop-2-en-1-one

^{1}H NMR (600 MHz, $CDCl_3$)

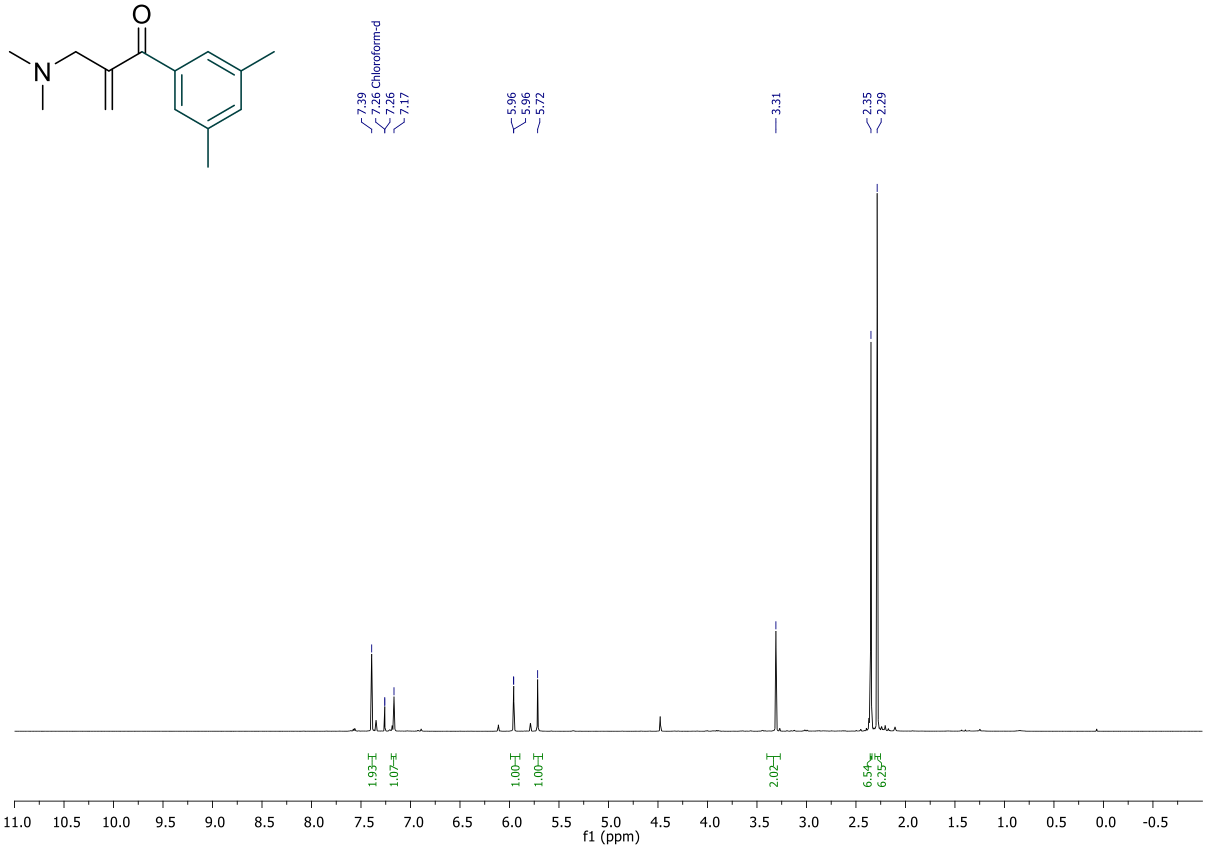

^{13}C NMR (151 MHz, $CDCl_3$)

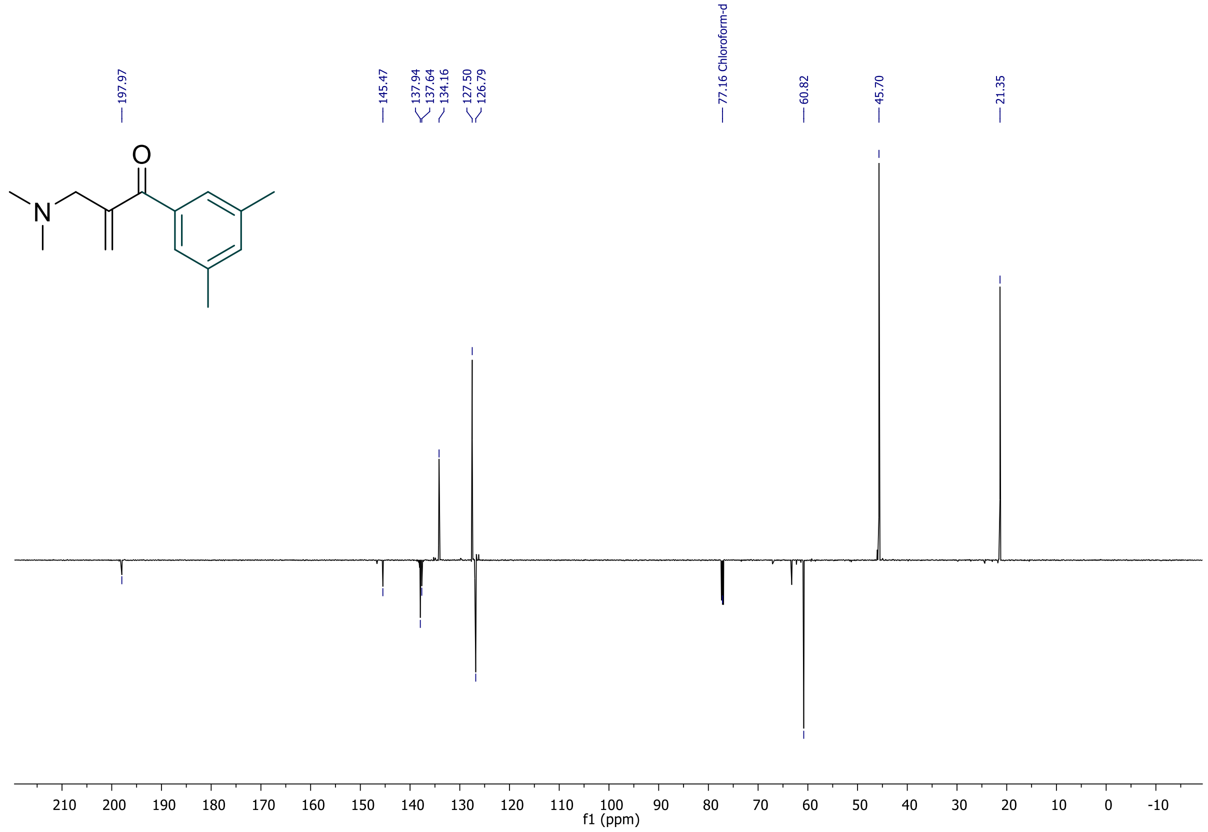

3g: 2-((Dimethylamino)methyl)-1-(thiophen-2-yl)prop-2-en-1-one

^{1}H NMR (600 MHz, $CDCl_3$)

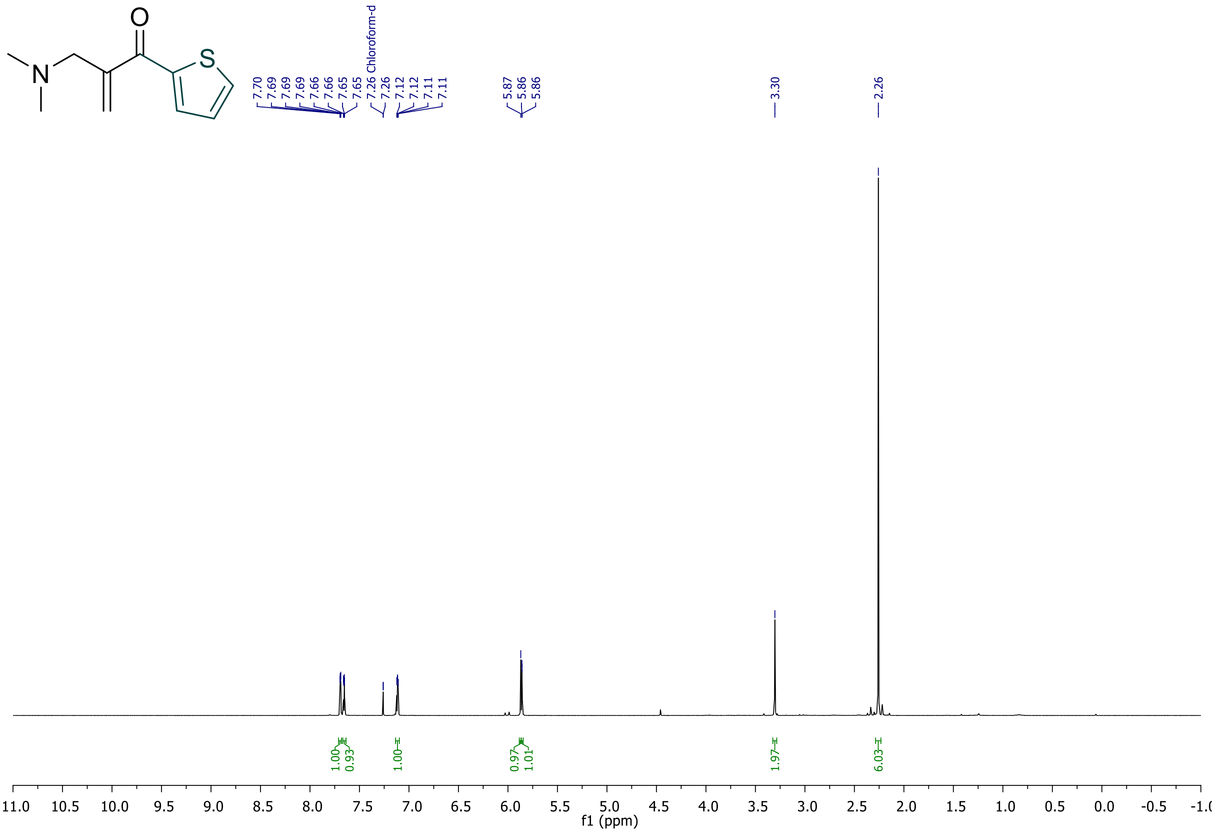

^{13}C NMR (151 MHz, $CDCl_3$)

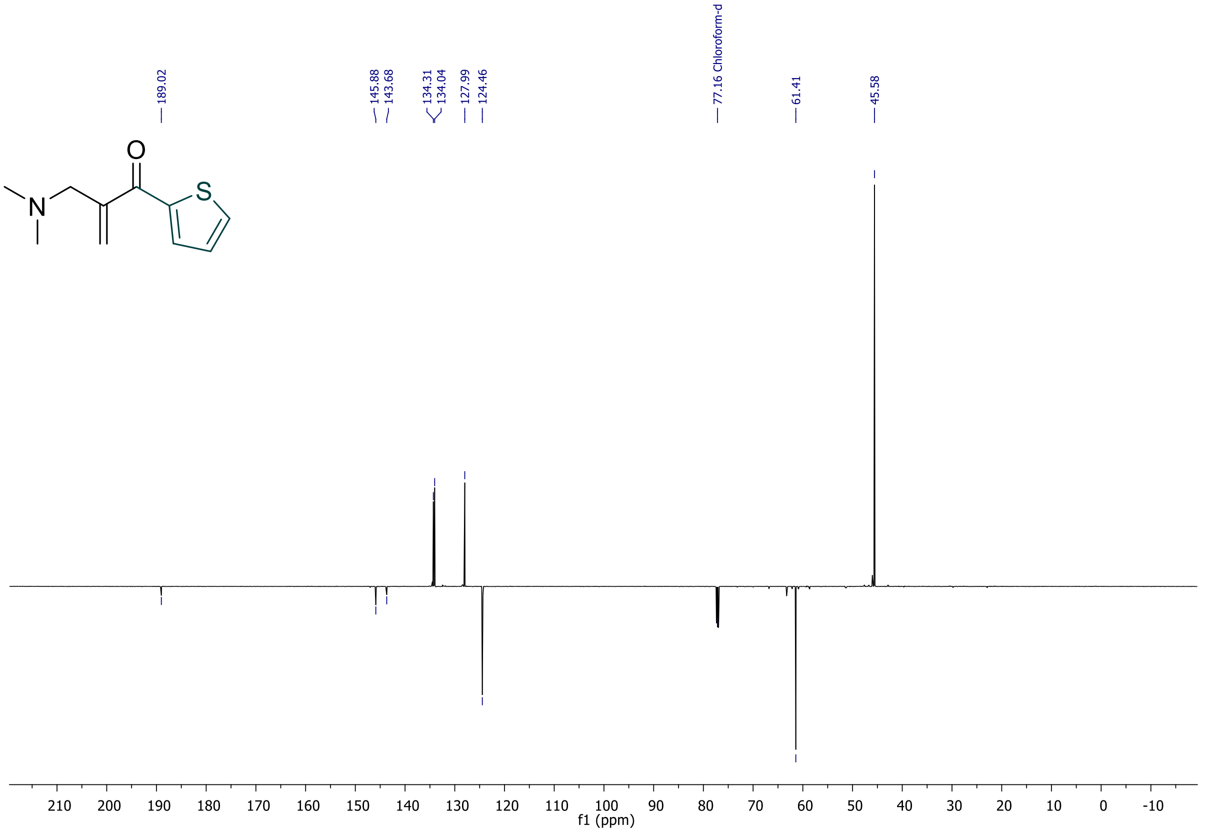

3h: 1-Cyclohexyl-2-((dimethylamino)methyl)prop-2-en-1-one

^{1}H NMR (600 MHz, $CDCl_3$)

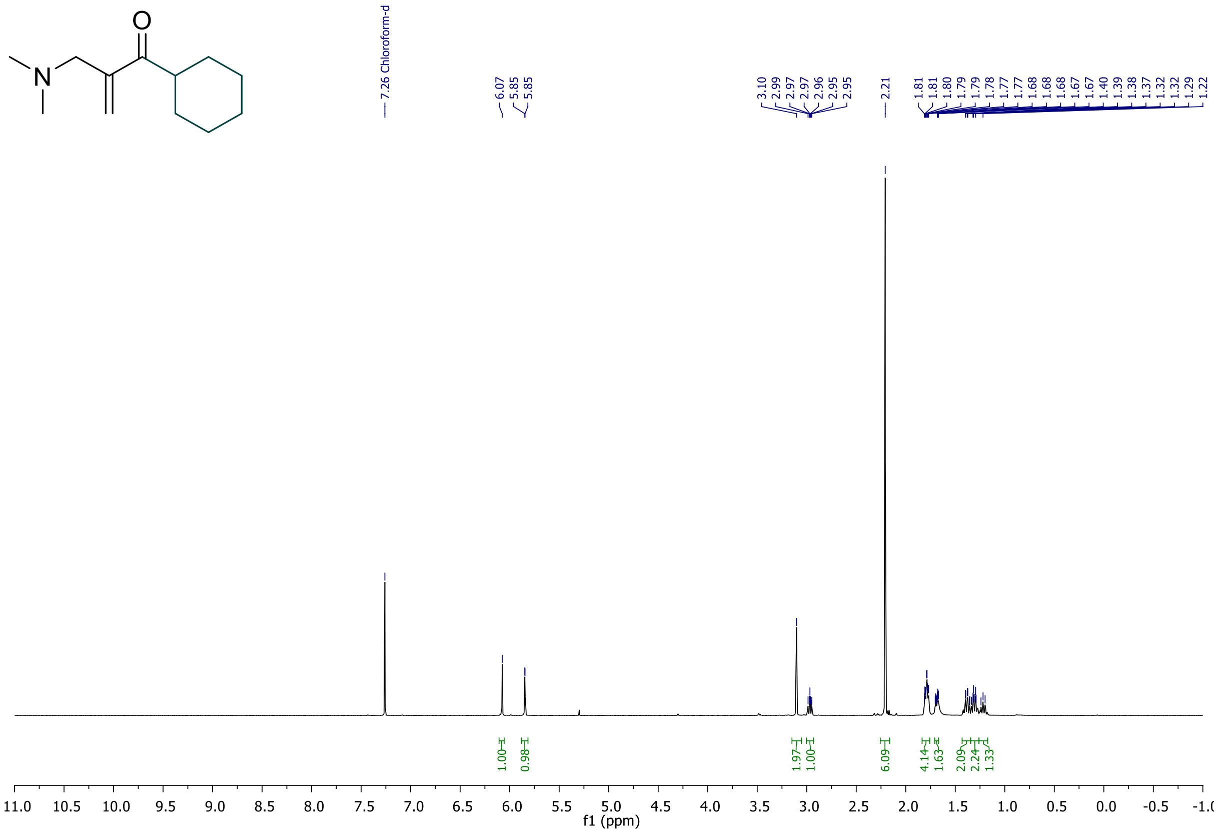

^{13}C NMR (151 MHz, $CDCl_3$)

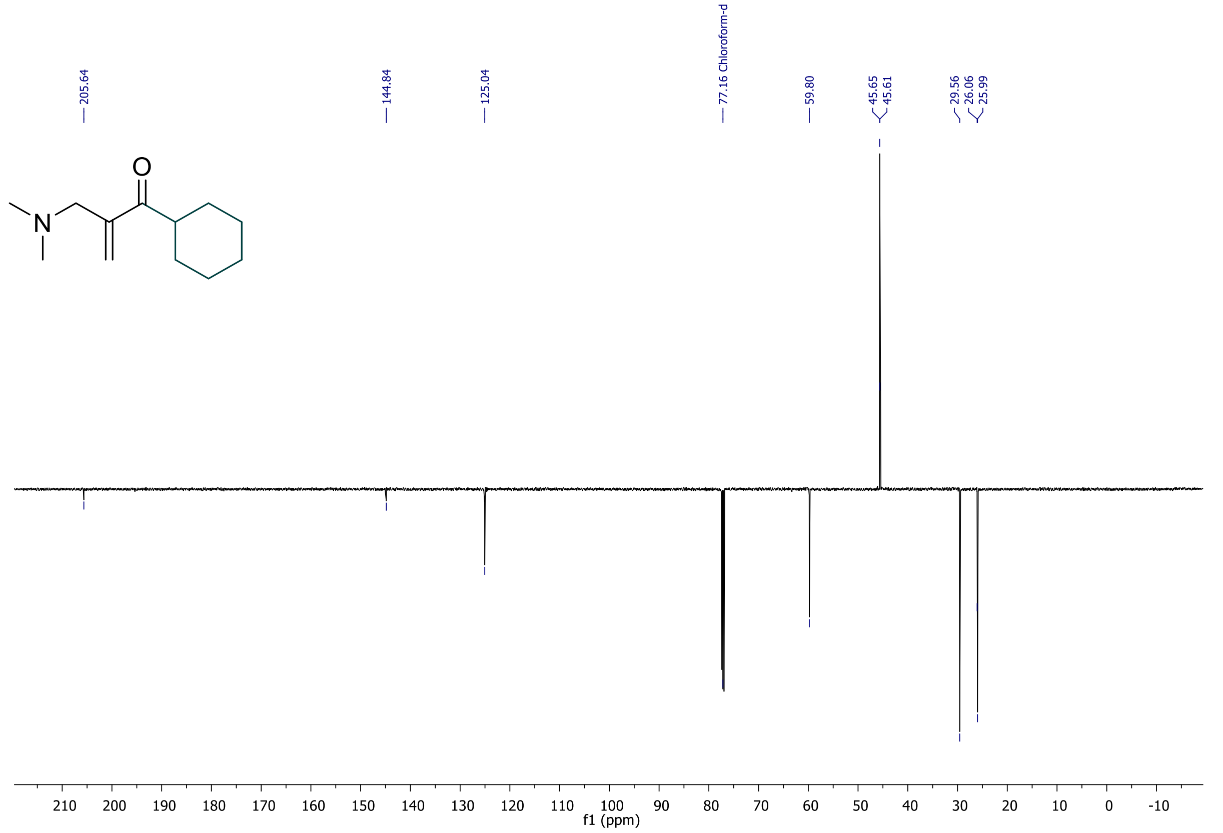

3i: 3-((Dimethylamino)methyl)but-3-en-2-one

^{1}H NMR (600 MHz, $CDCl_3$)

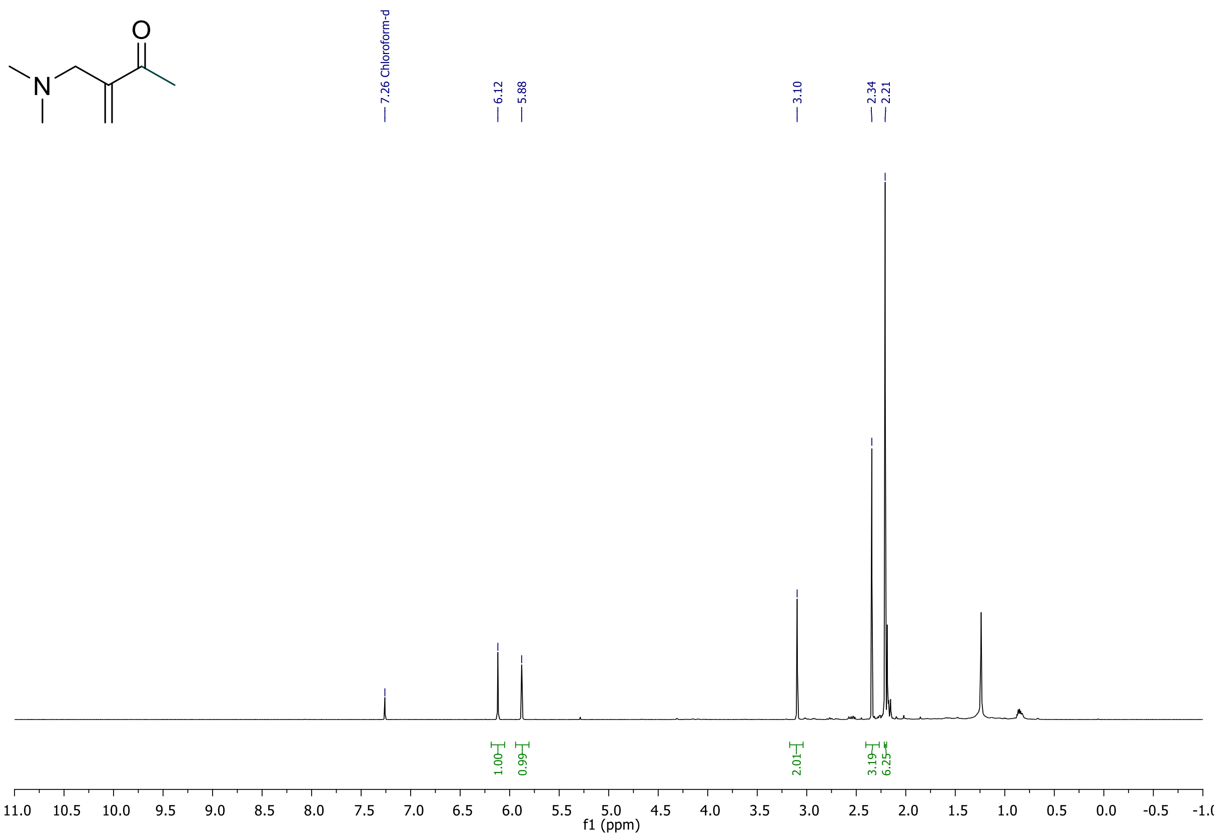

^{13}C NMR (151 MHz, $CDCl_3$)

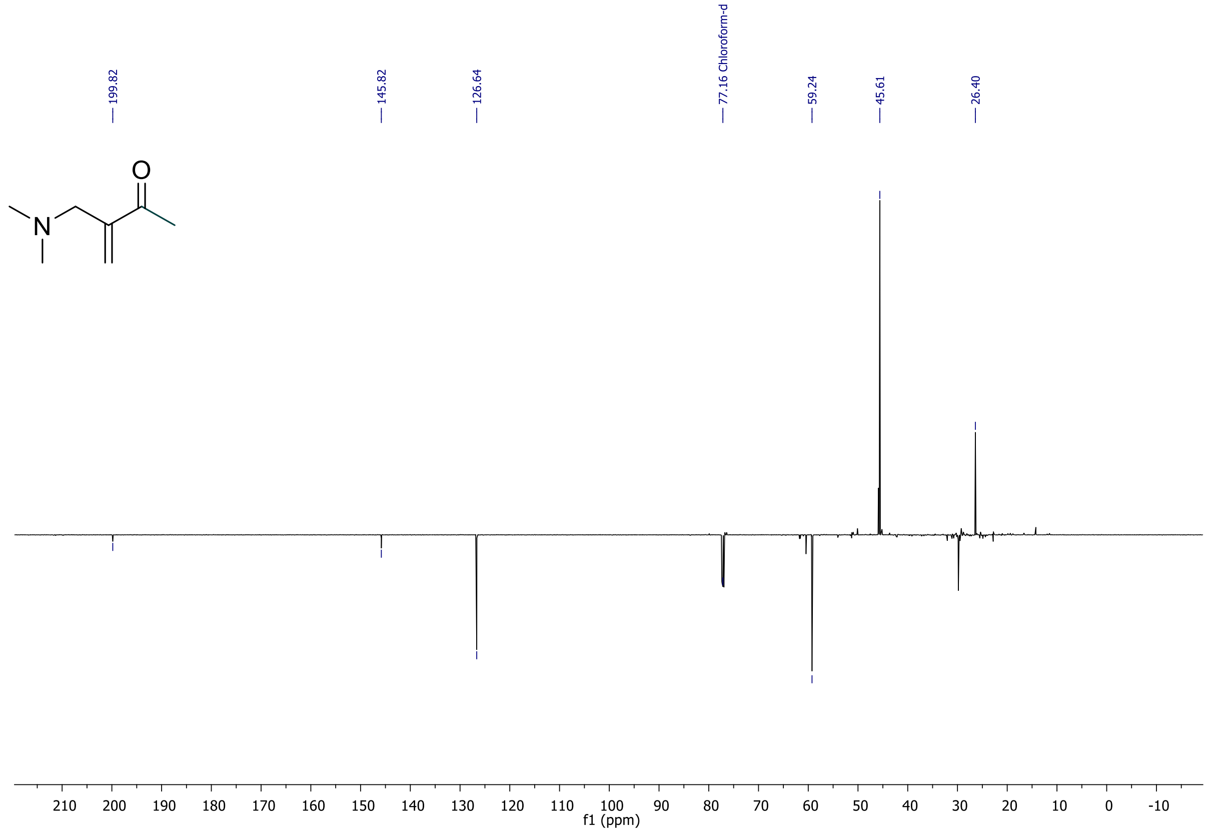

3j: 2-((Dimethylamino)methyl)cyclopent-2-en-1-one

^{1}H NMR (600 MHz, CDCl$_3$)

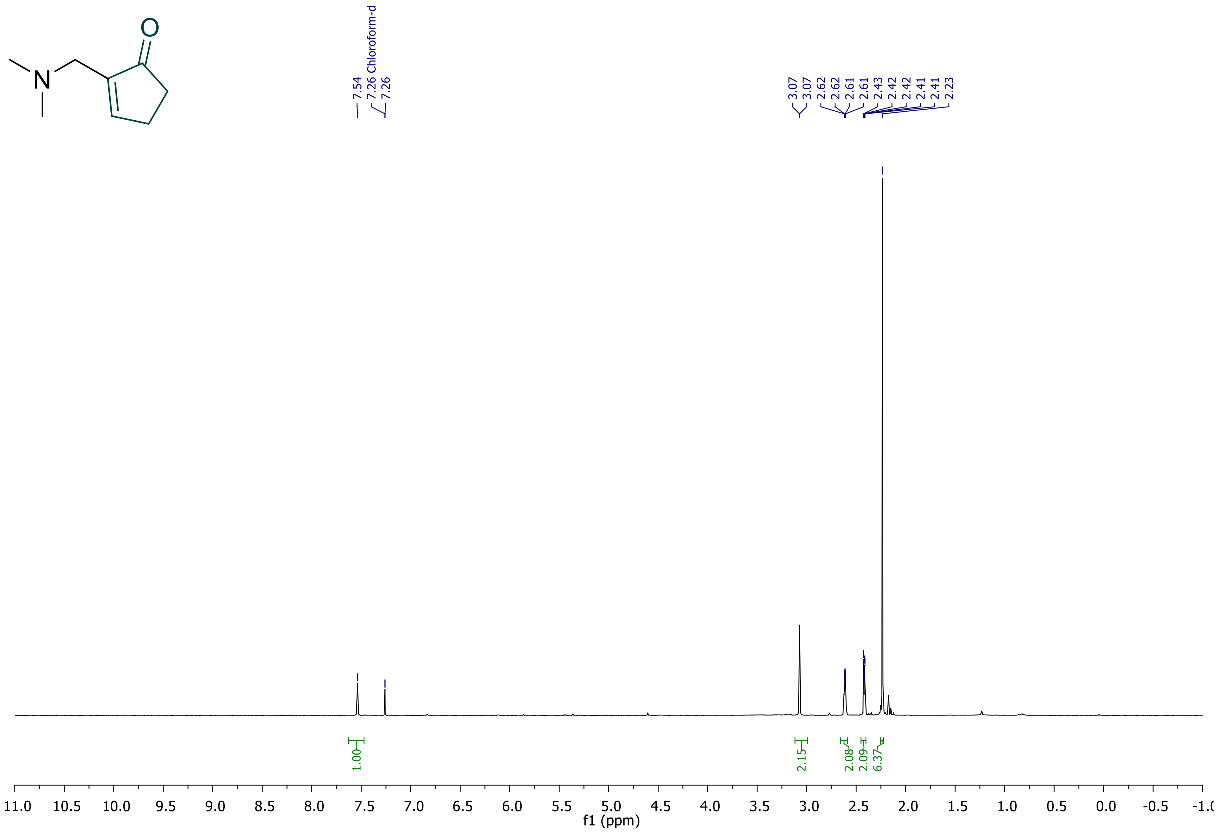

^{13}C NMR (151 MHz, CDCl$_3$)

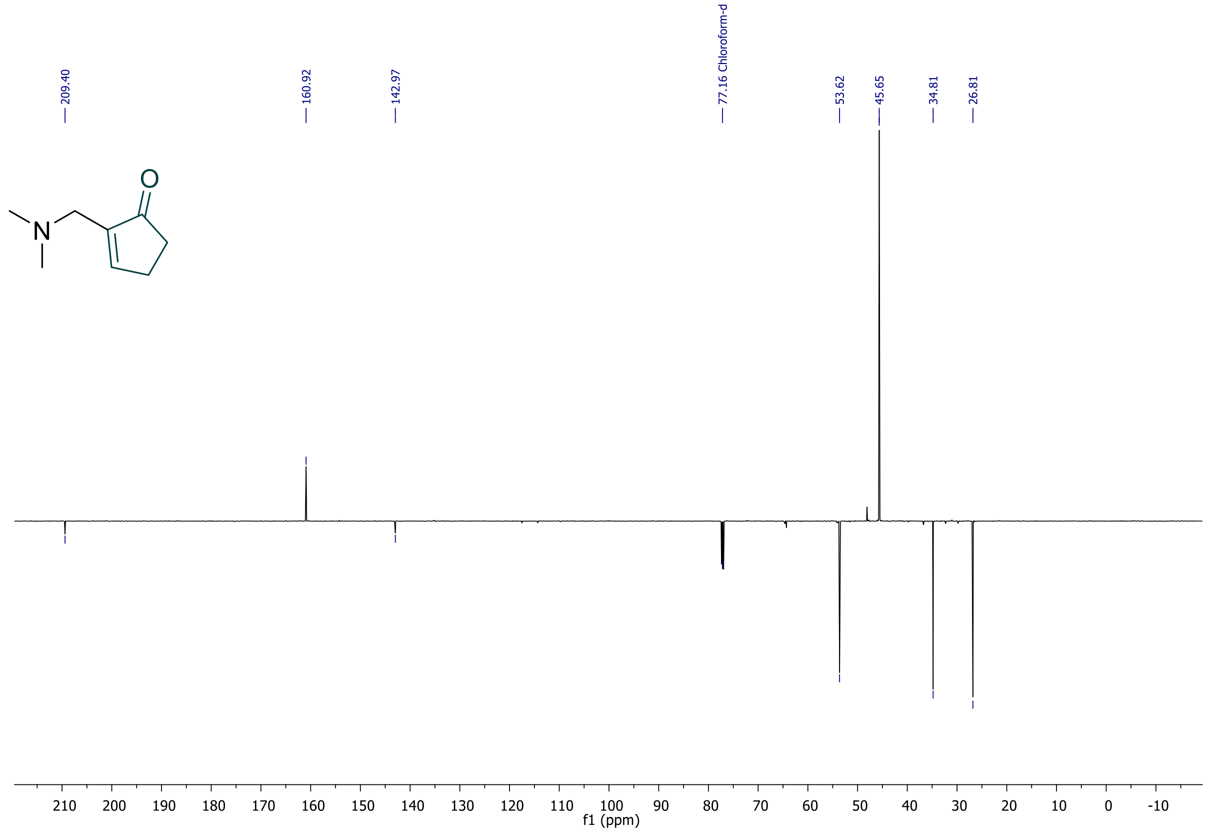

3k: 2-((Dimethylamino)methyl)cyclohex-2-en-1-one

^{1}H NMR (600 MHz, $CDCl_3$)

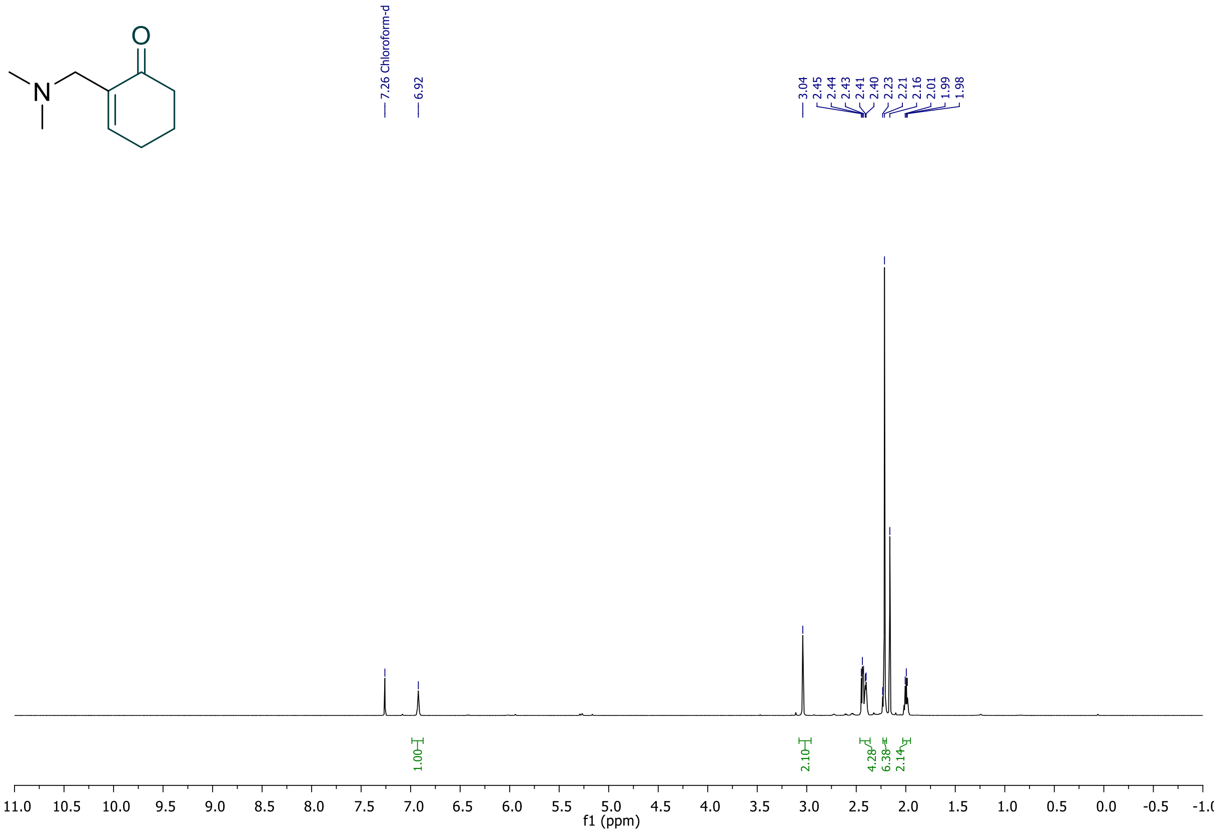

^{13}C NMR (151 MHz, $CDCl_3$)

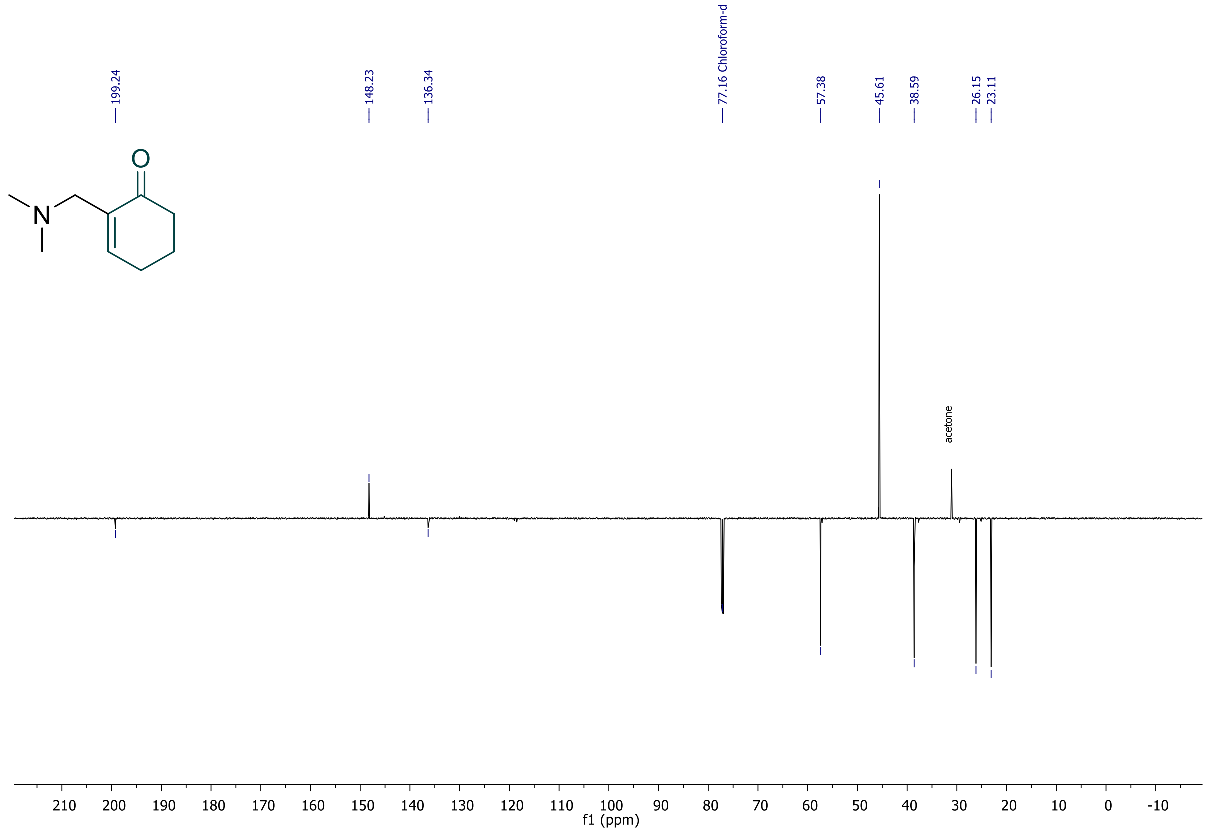

3l: 2-((Dimethylamino)methyl)-1-(pyridin-3-yl)prop-2-en-1-one

^{1}H NMR (400 MHz, $CDCl_3$)

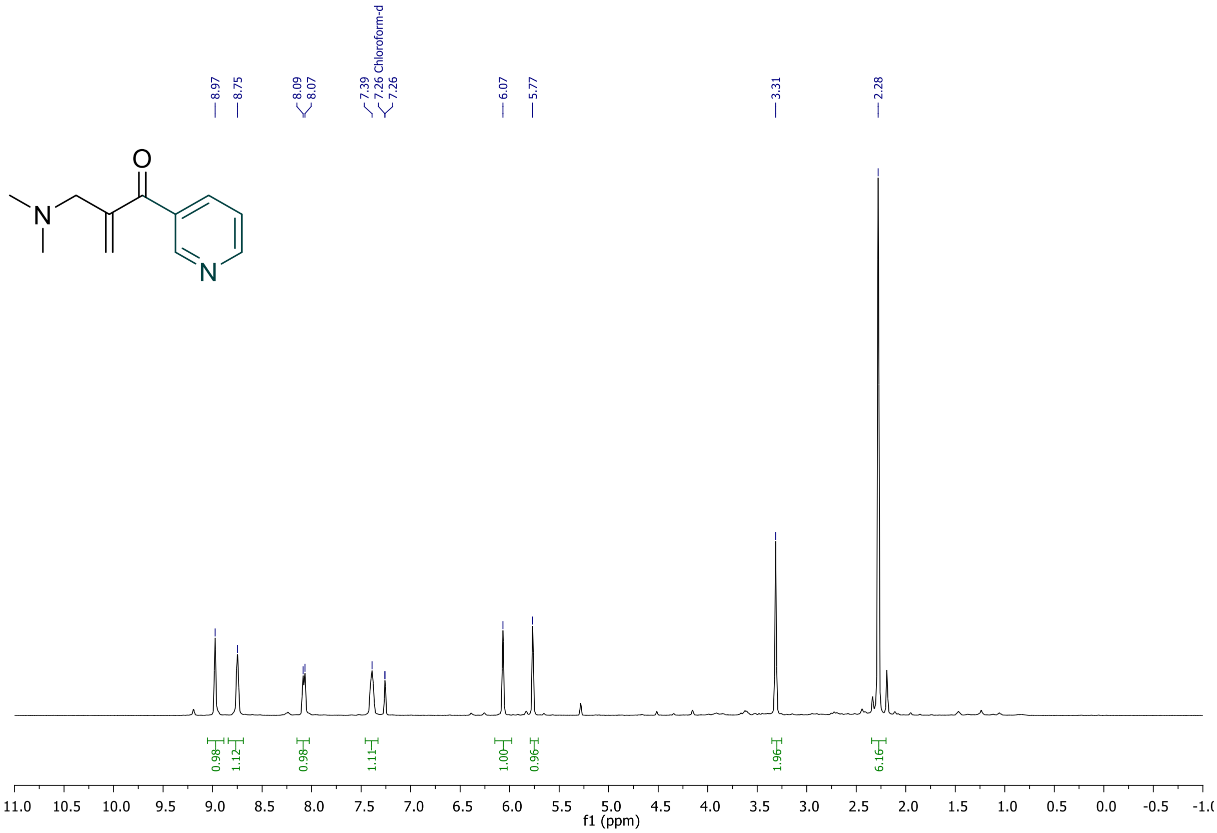

^{13}C NMR (101 MHz, $CDCl_3$)

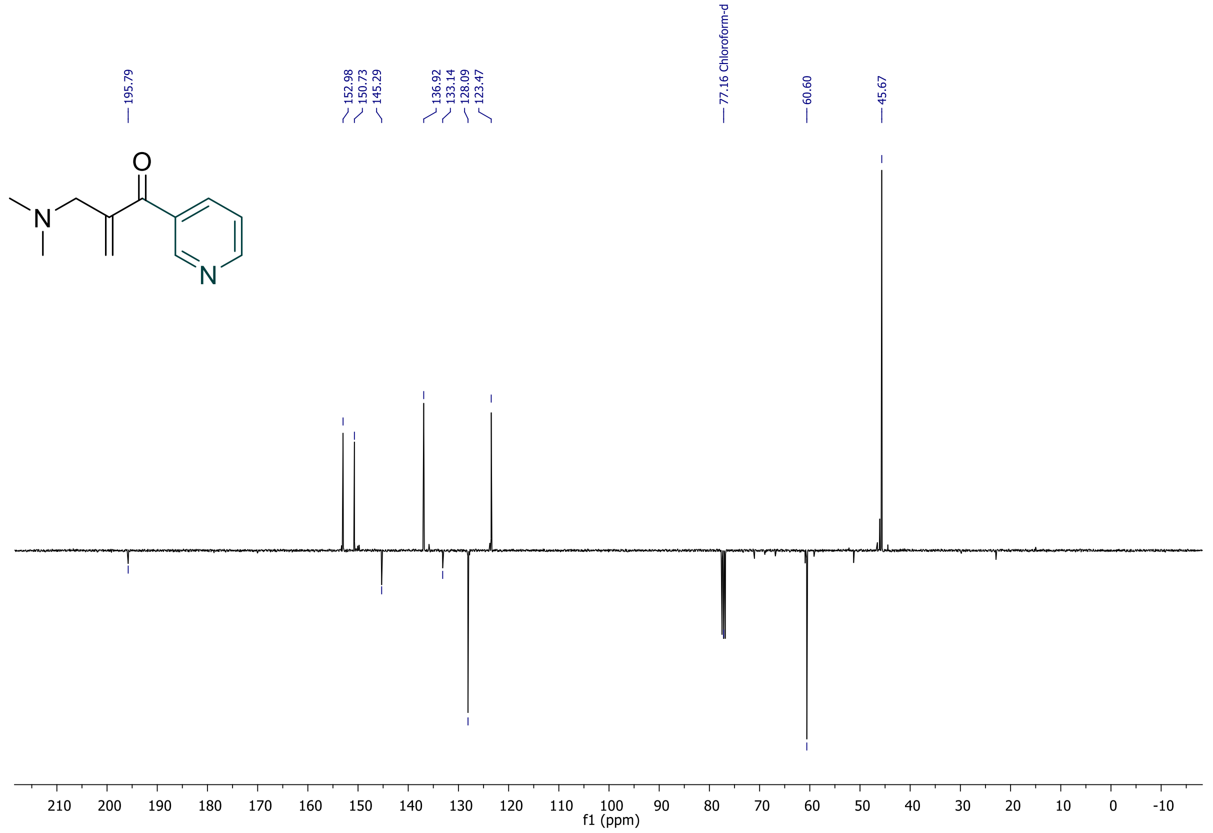

6a: 1-Ethyl 5-phenethyl 2-(dimethylamino)-4-methylenepentanedioate

^{1}H NMR (600 MHz, $CDCl_3$)

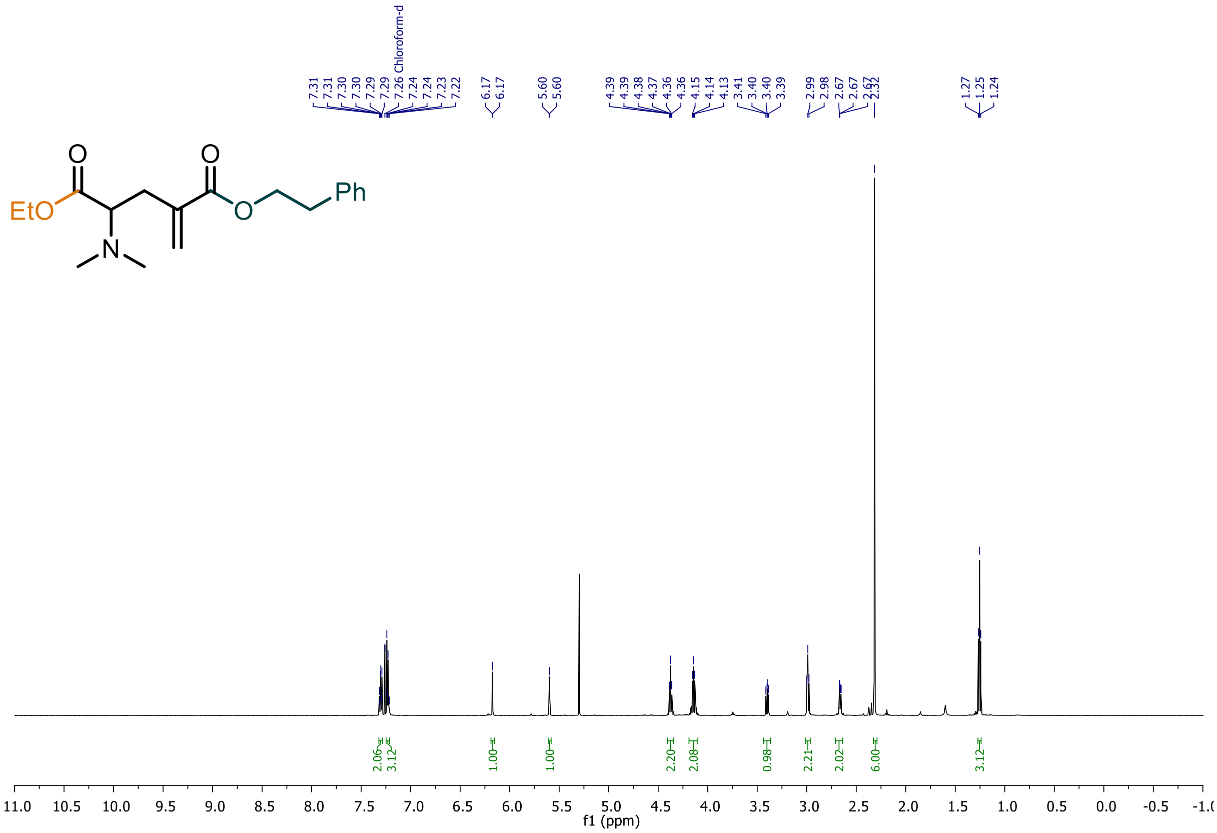

^{13}C NMR (151 MHz, $CDCl_3$)

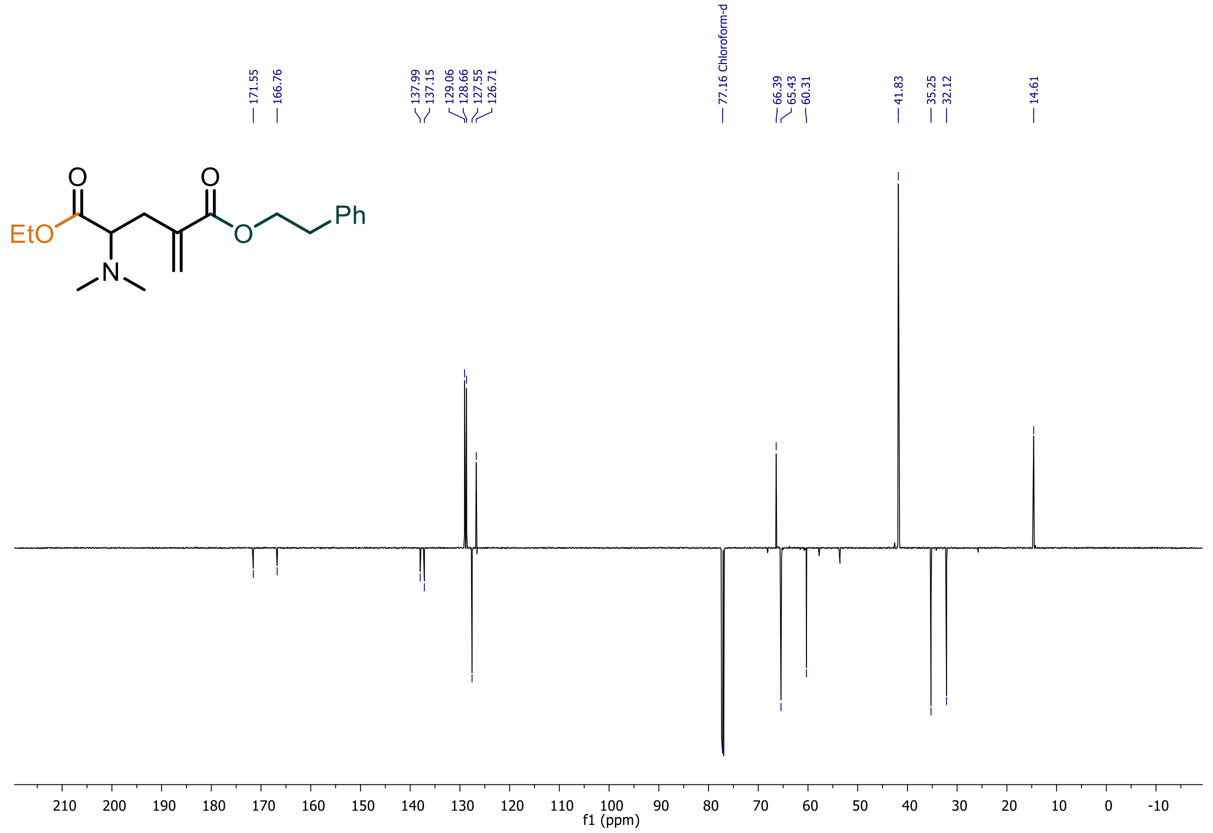

6b: Phenethyl 4-(dimethylamino)-2-methylene-5-oxoheptanoate

^{1}H NMR (600 MHz, CDCl$_3$)

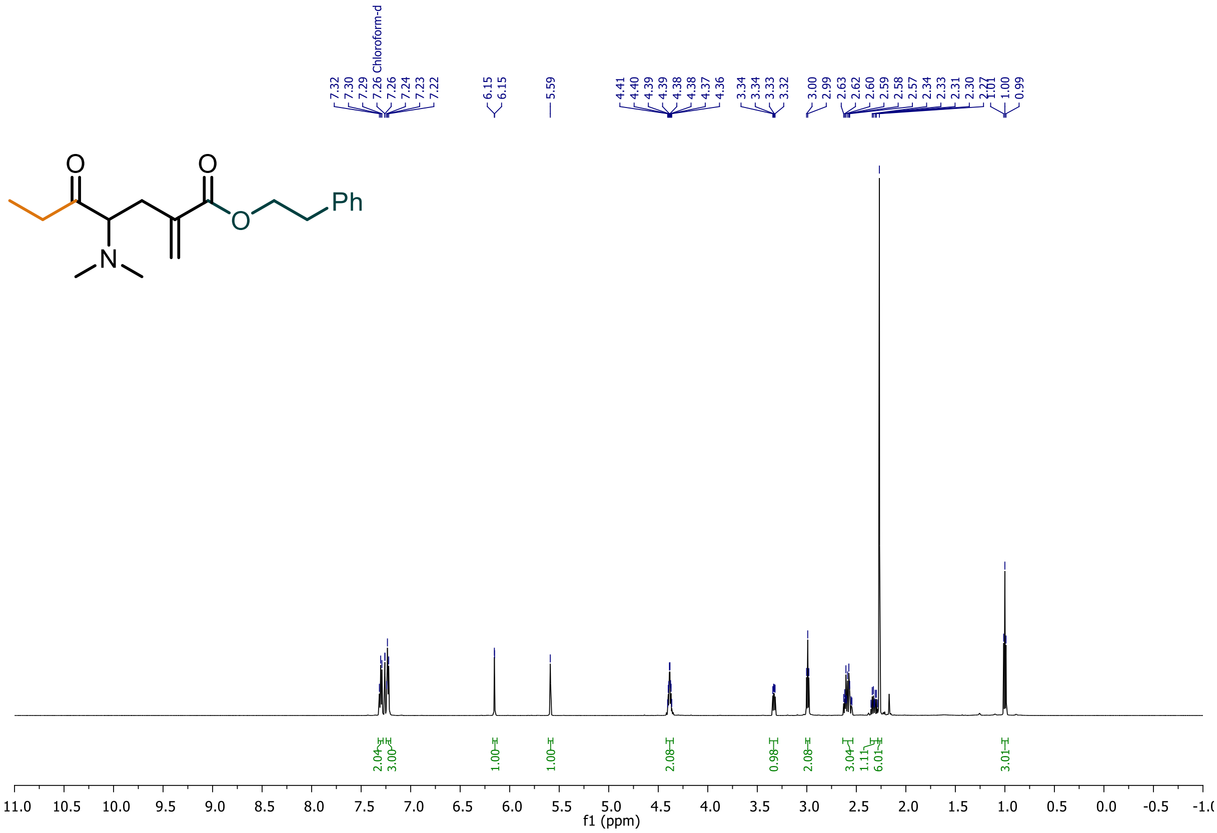

^{13}C NMR (151 MHz, CDCl$_3$)

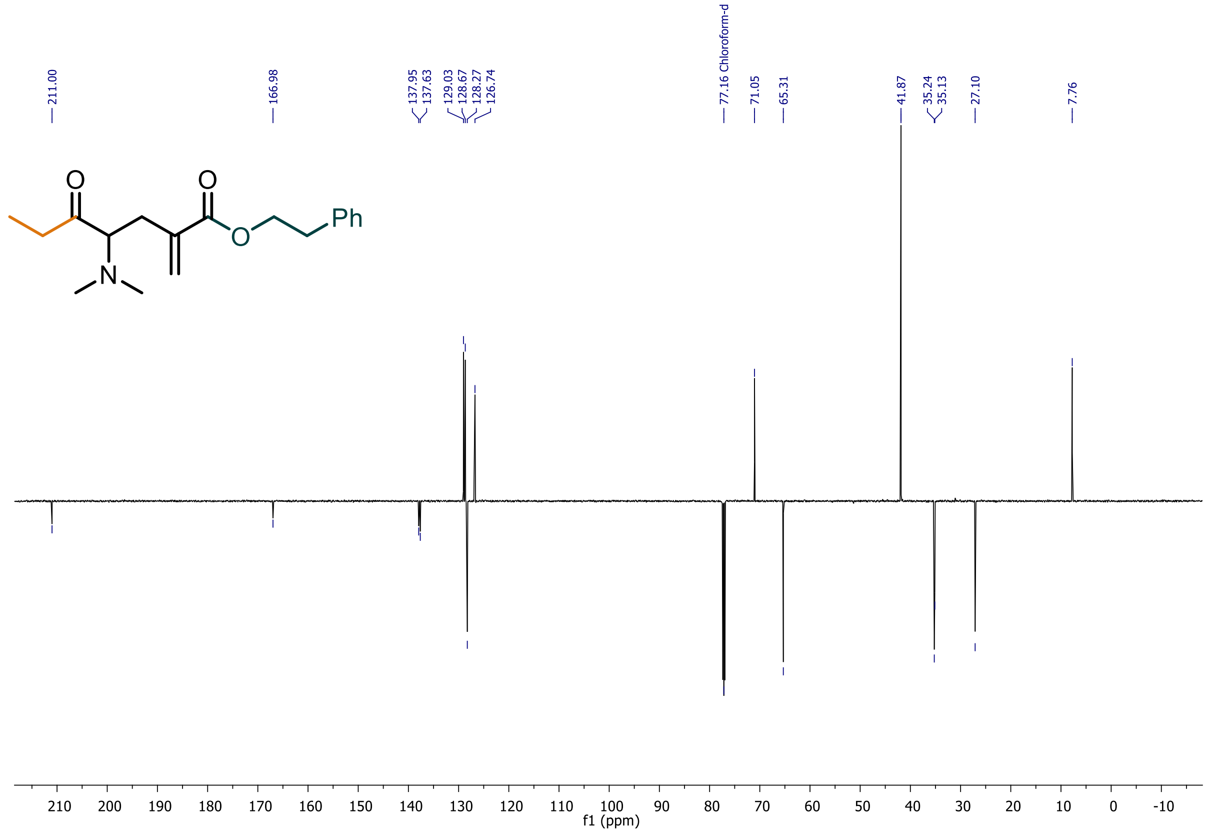

6c: Phenethyl 4-(dimethylamino)-2-methylene-5-oxo-5-phenylpentanoate

^{1}H NMR (700 MHz, $CDCl_3$)

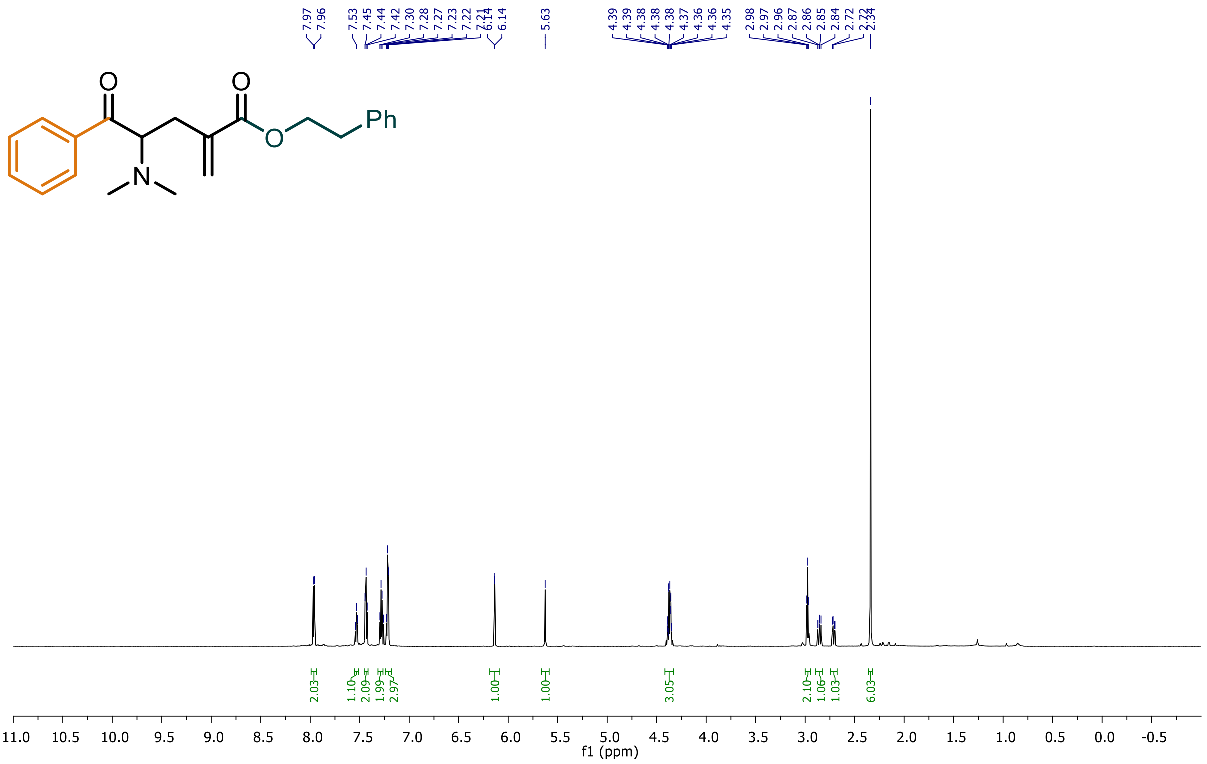

^{13}C NMR (176 MHz, $CDCl_3$)

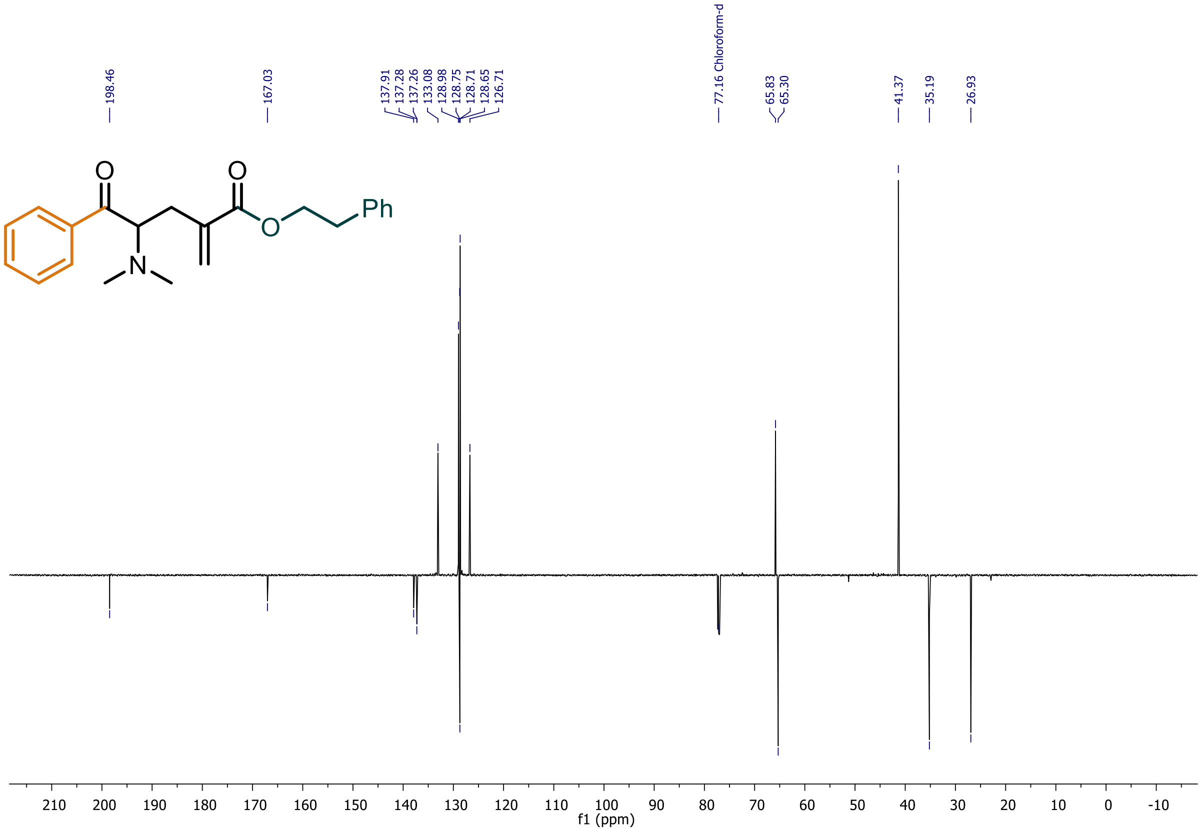

6d: Phenethyl 4-(dimethylamino)-5-(4-methoxyphenyl)-2-methylene-5-oxopentanoate

^{1}H NMR (600 MHz, $CDCl_3$)

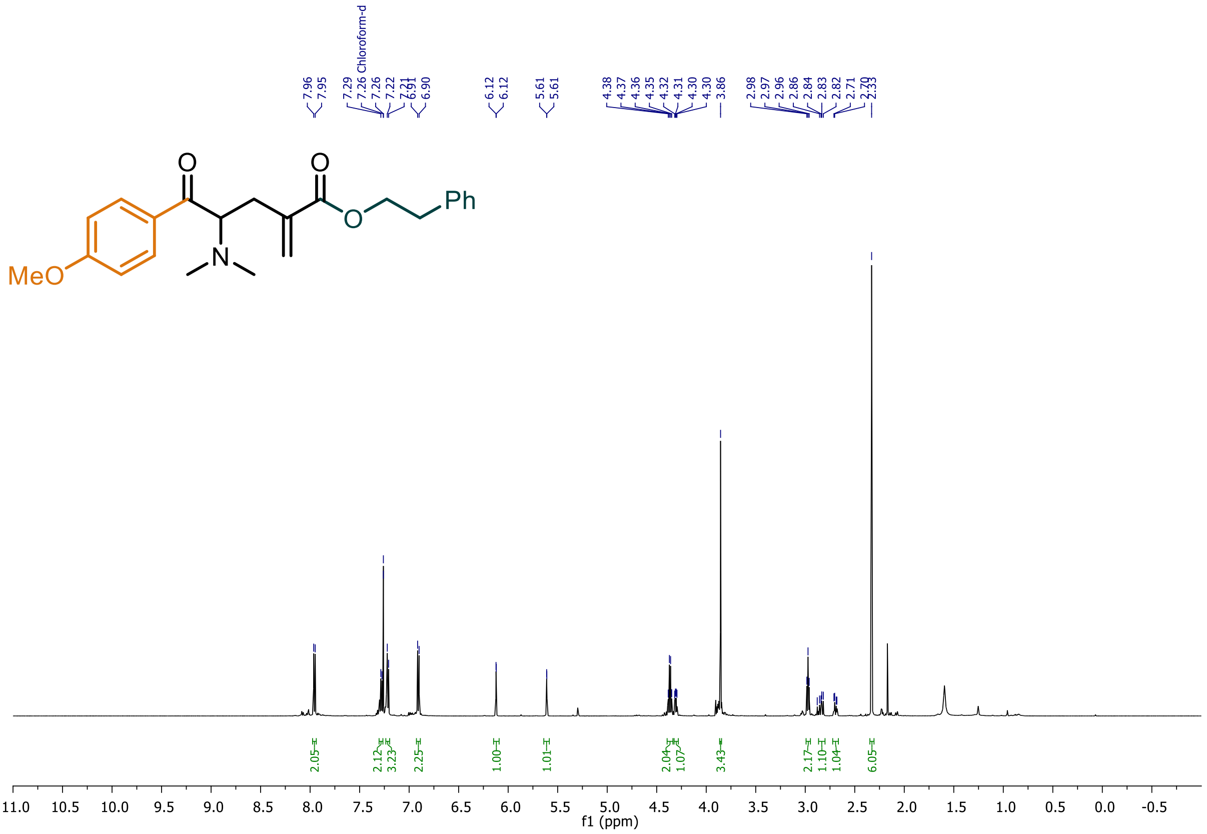

^{13}C NMR (151 MHz, $CDCl_3$)

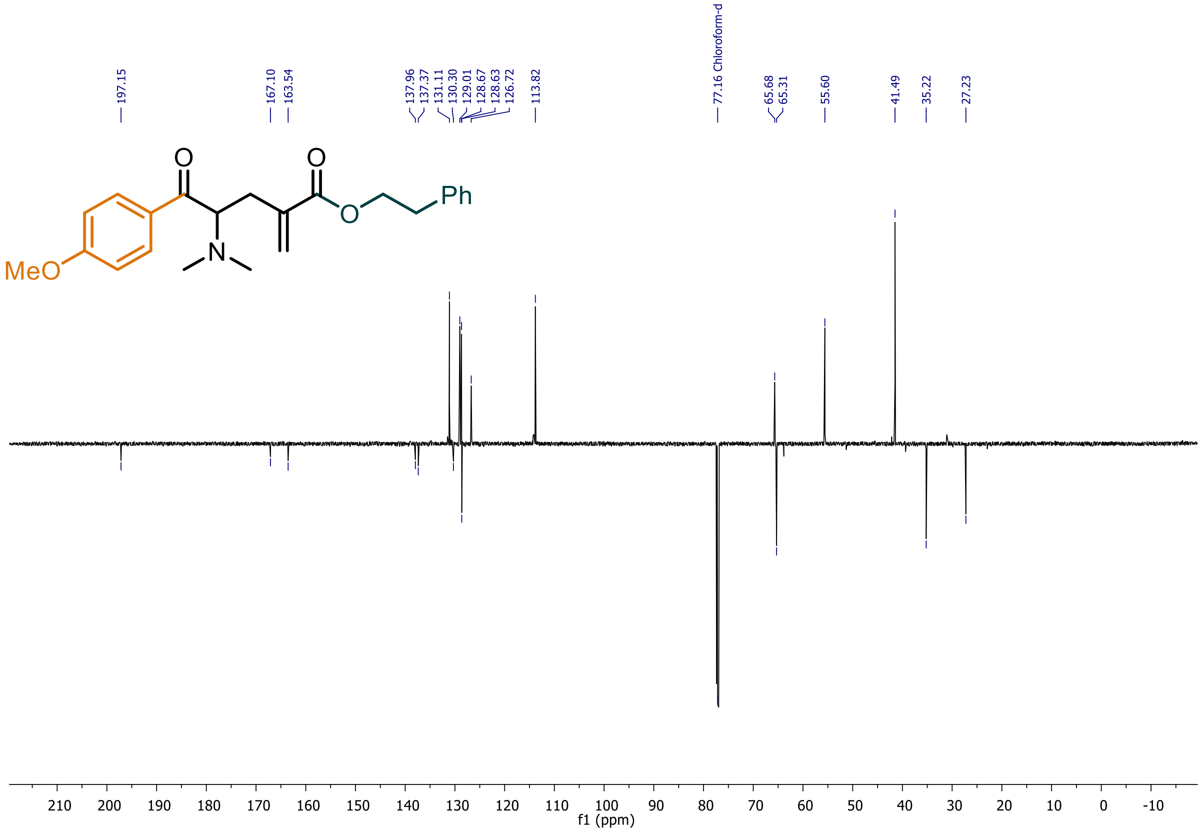

6e: Phenethyl 4-(dimethylamino)-2-methylene-5-(naphthalen-2-yl)-5-oxopentanoate

^{1}H NMR (600 MHz, CDCl$_3$)

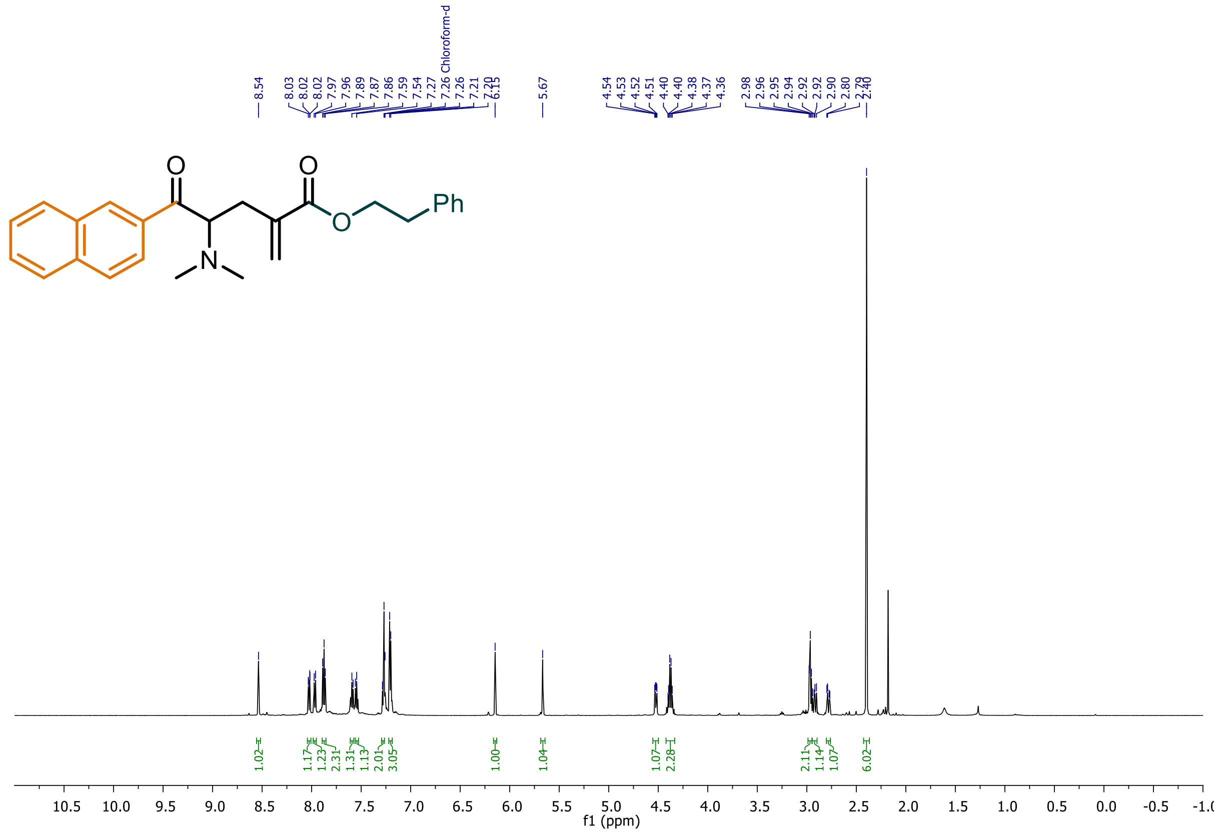

^{13}C NMR (151 MHz, CDCl$_3$)

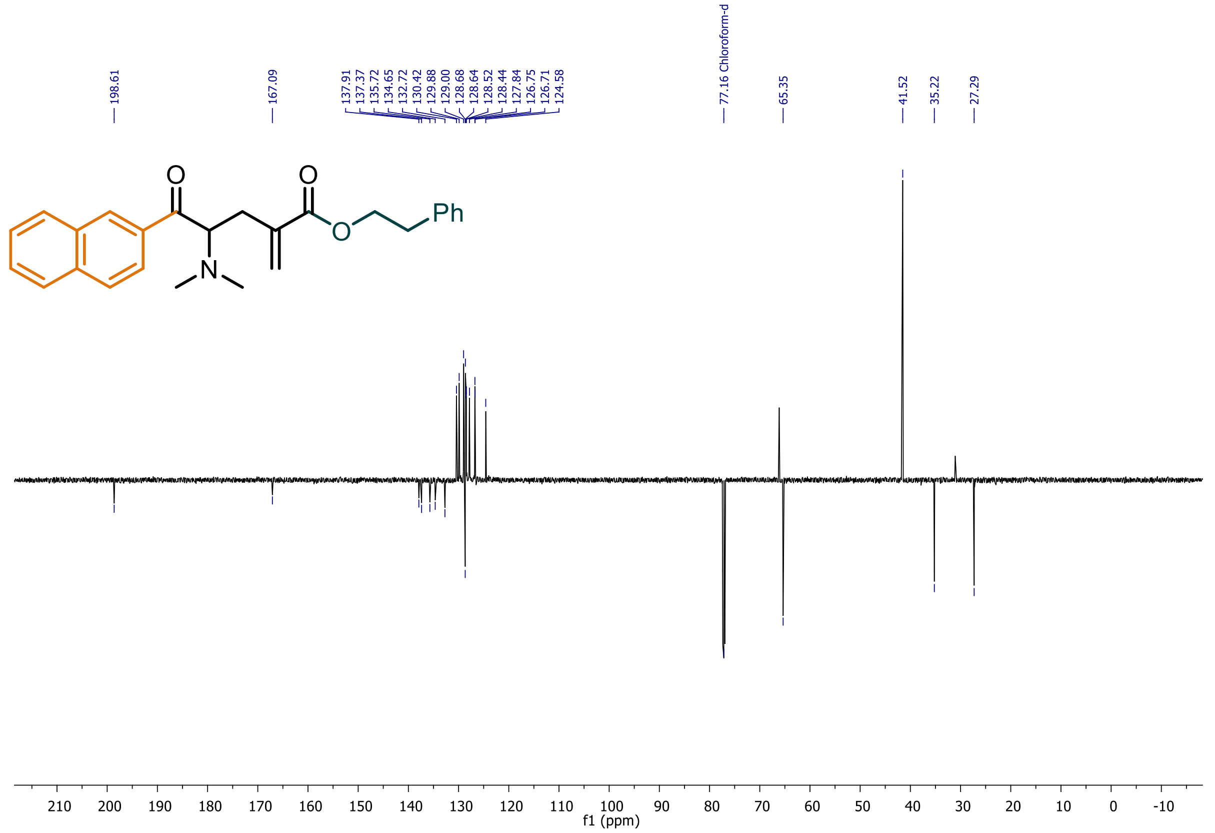

7a: 5-(2-((Dimethylammonio)methyl)-3-oxo-3-phenylpropyl)-2,2-dimethyl-4-oxo-4H-1,3-dioxin-6-olate

^{1}H NMR (600 MHz, DMSO-d$_6$):

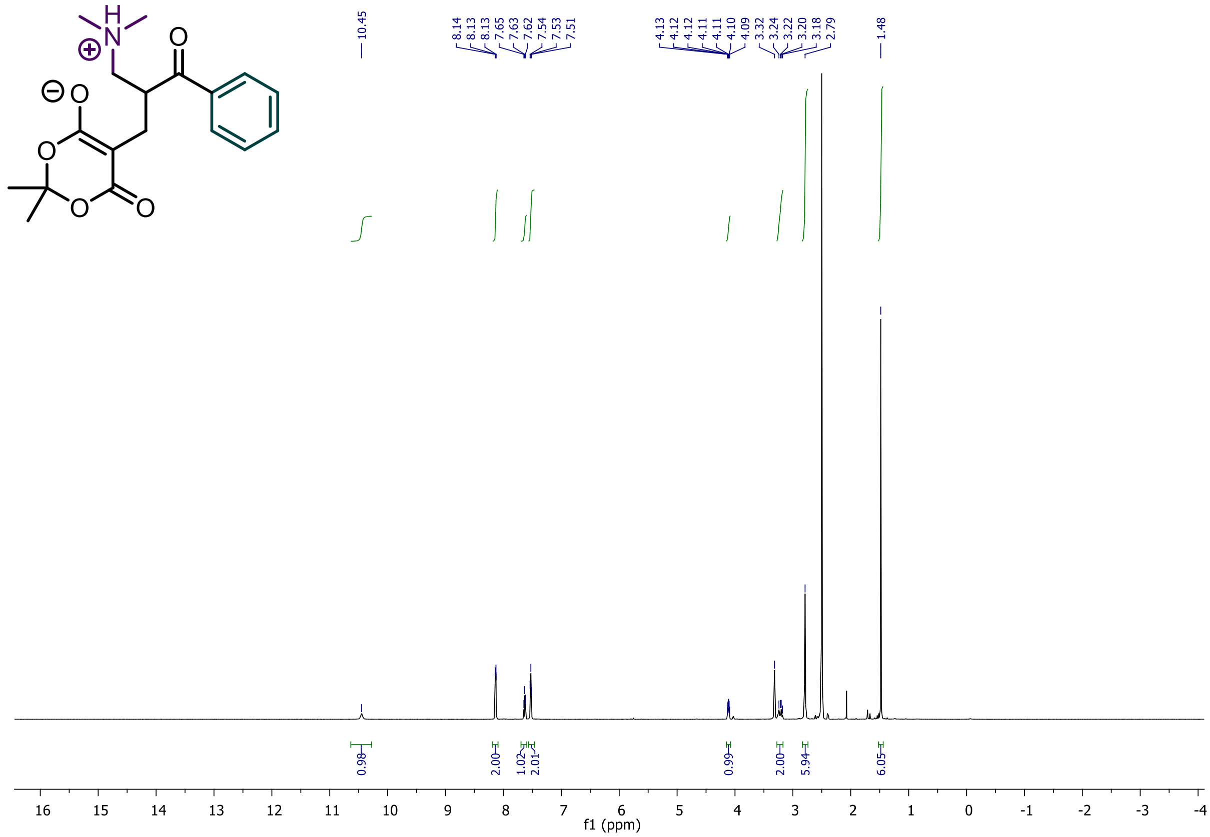

^{13}C NMR (150 MHz, DMSO-d$_6$):

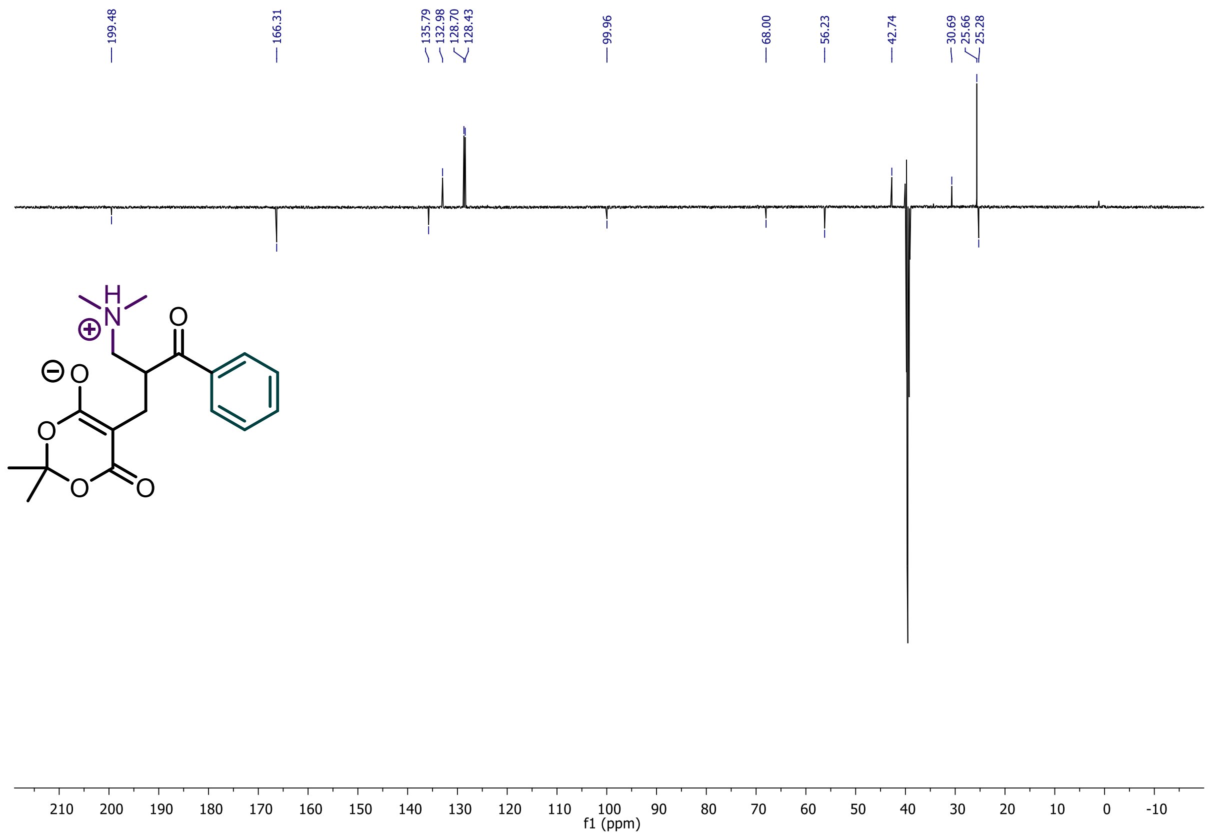

7b: 5-(2-((Dimethylammonio)methyl)-3-(naphthalen-1-yl)-3-oxopropyl)-2,2-dimethyl-4-oxo-4H-1,3-dioxin-6-olate

^{1}H NMR (400 MHz, DMSO-d$_6$):

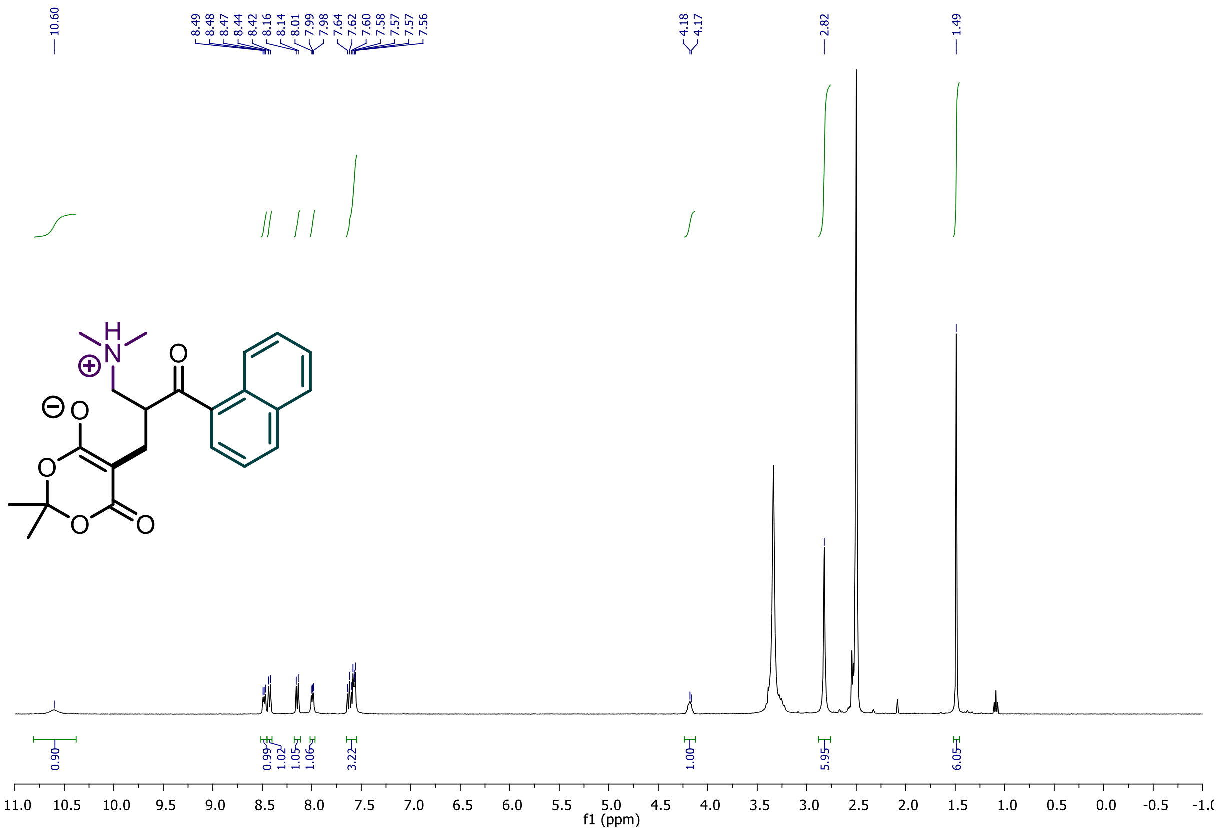

^{13}C NMR (100 MHz, DMSO-d$_6$):

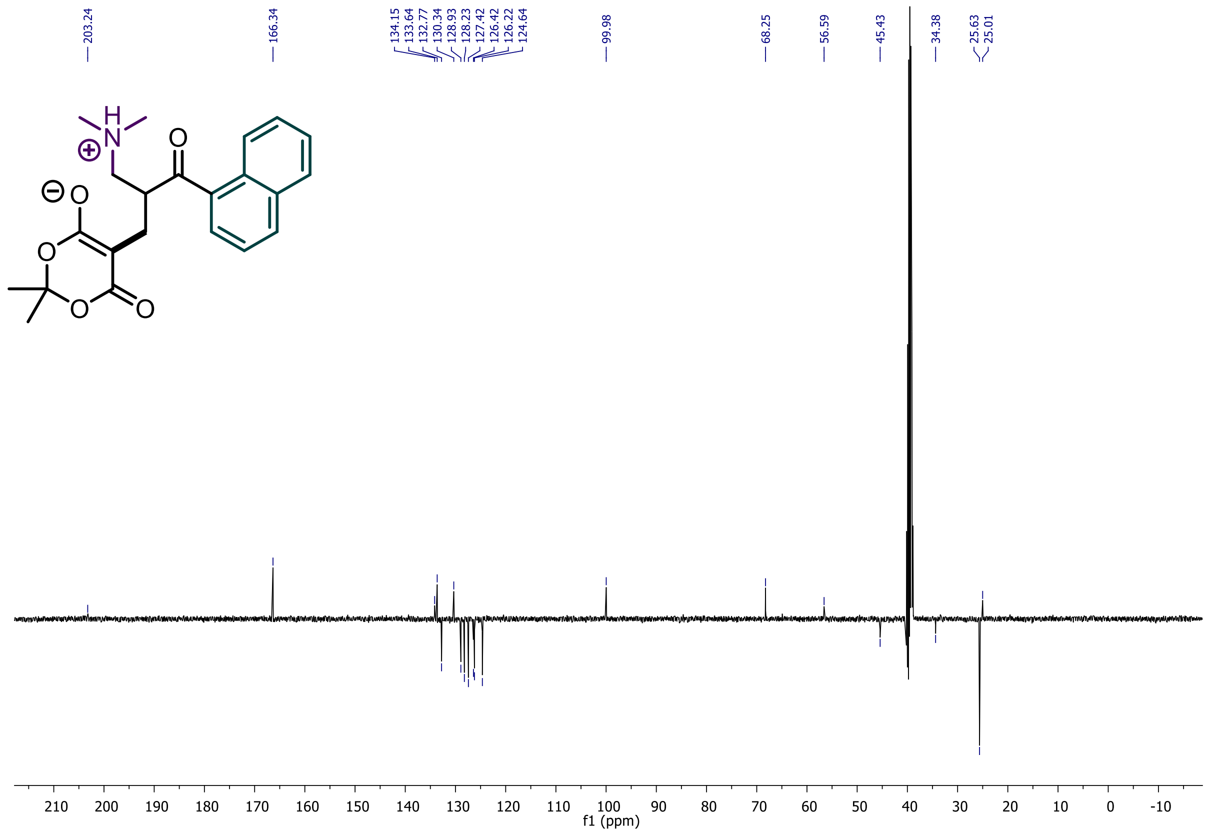

7c: 5-(2-((Dimethylammonio)methyl)-3-oxo-3-(pyren-1-yl)propyl)-2,2-dimethyl-4-oxo-4H-1,3-dioxin-6-olate

^{1}H NMR (400 MHz, DMSO-d$_6$):

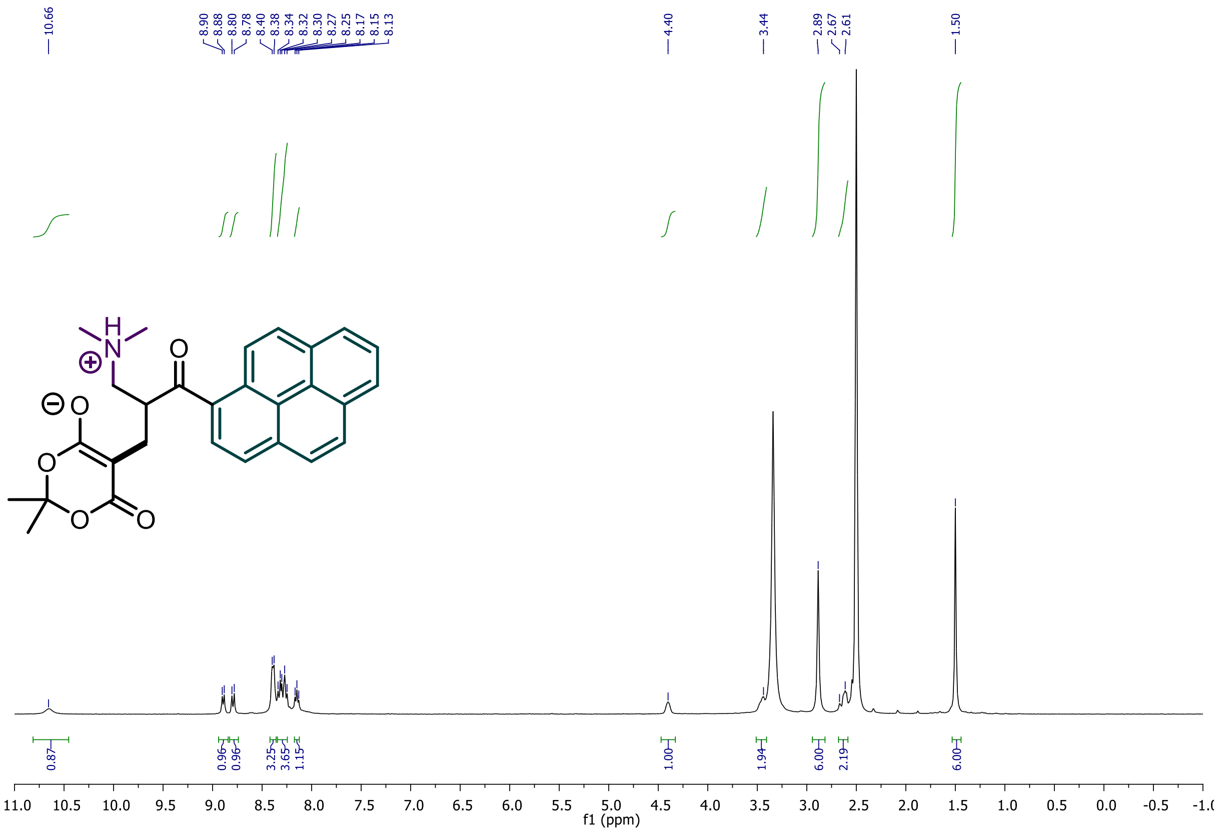

^{13}C NMR (100 MHz, DMSO-d$_6$):

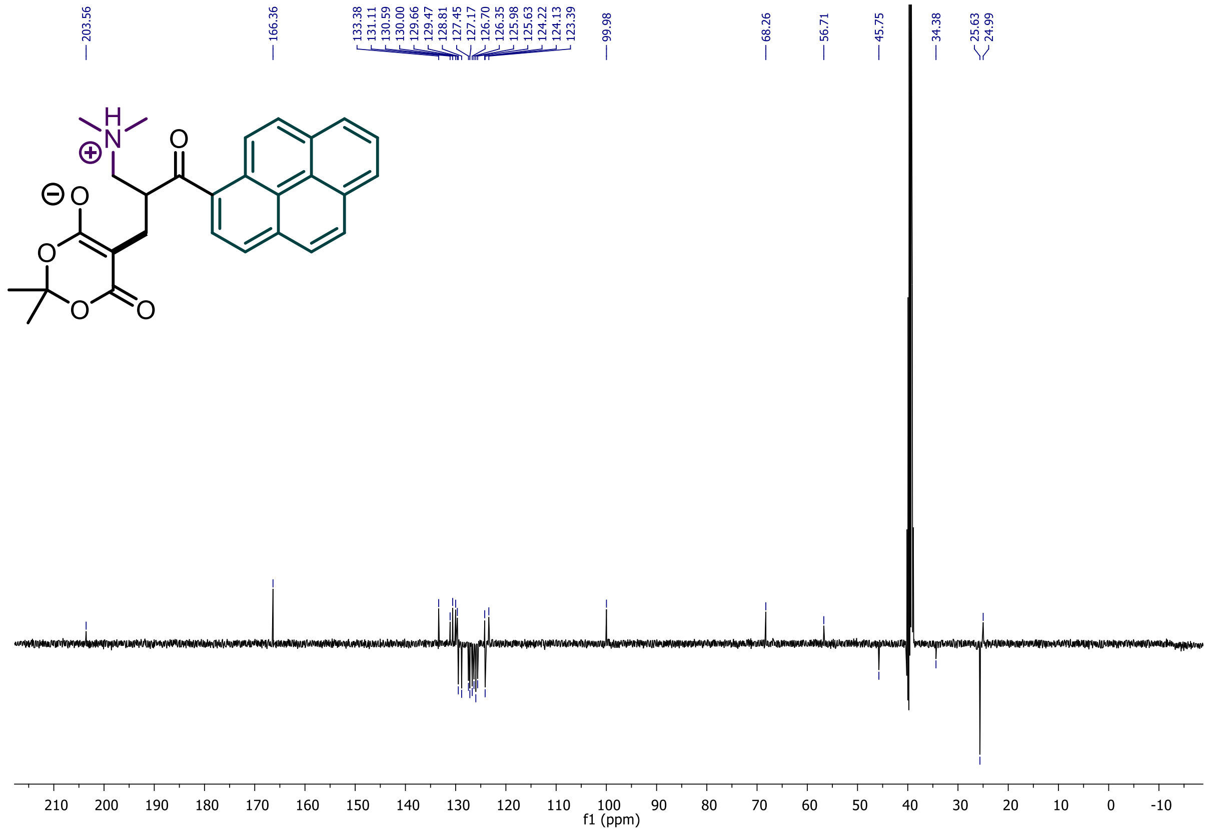

7d: 5-(2-((Dimethylammonio)methyl)-3-(4-nitrophenyl)-3-oxopropyl)-2,2-dimethyl-4-oxo-4H-1,3-dioxin-6-olate

^{1}H NMR (400 MHz, $CDCl_3$):

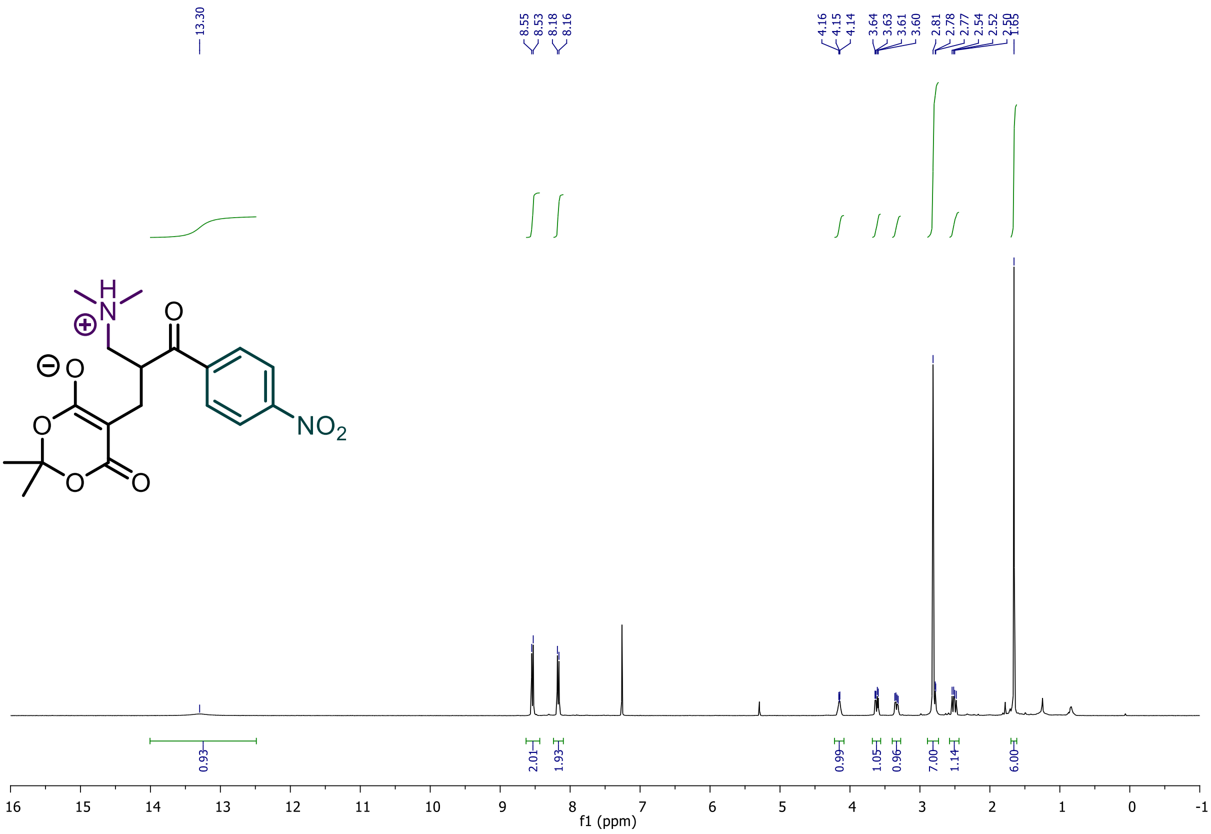

^{13}C NMR (100 MHz, $CDCl_3$):

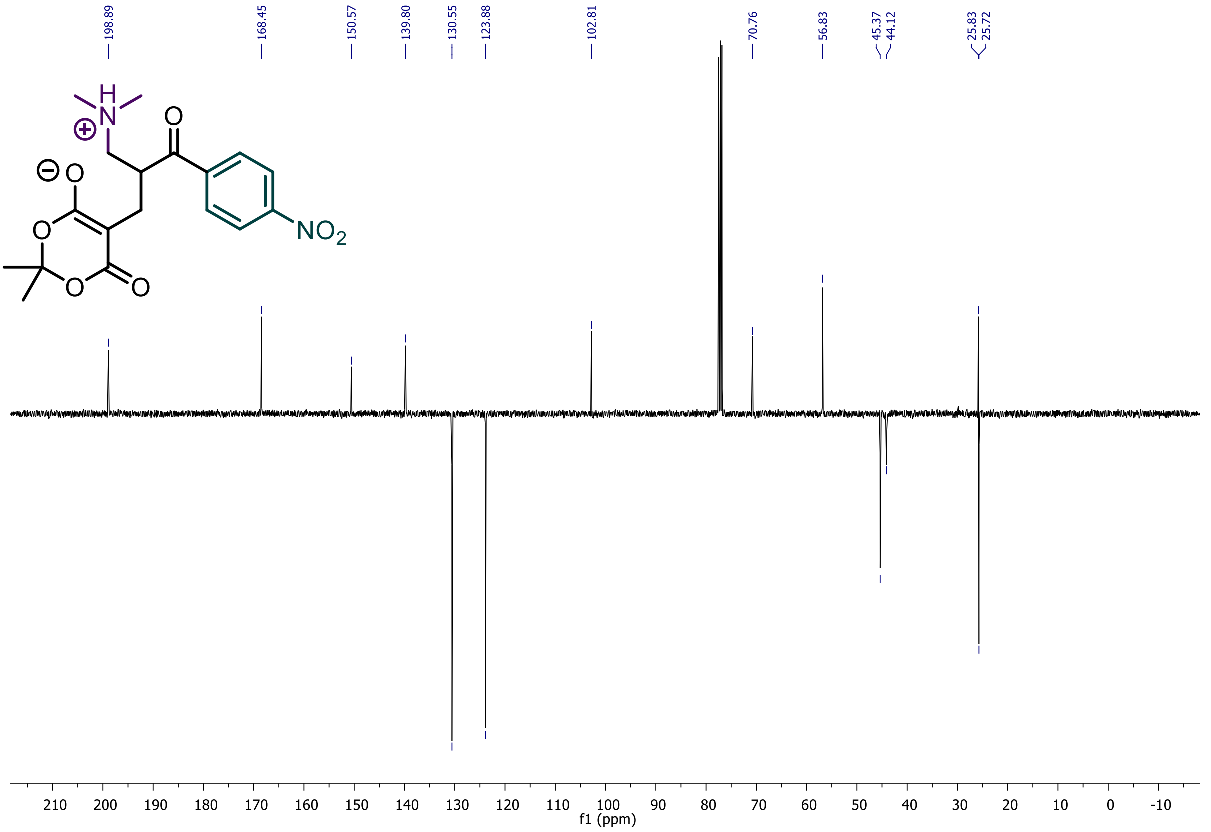

4: Phenethyl 2-((dimethylamino)methyl)acrylate

^{1}H NMR (400 MHz, $CDCl_3$)

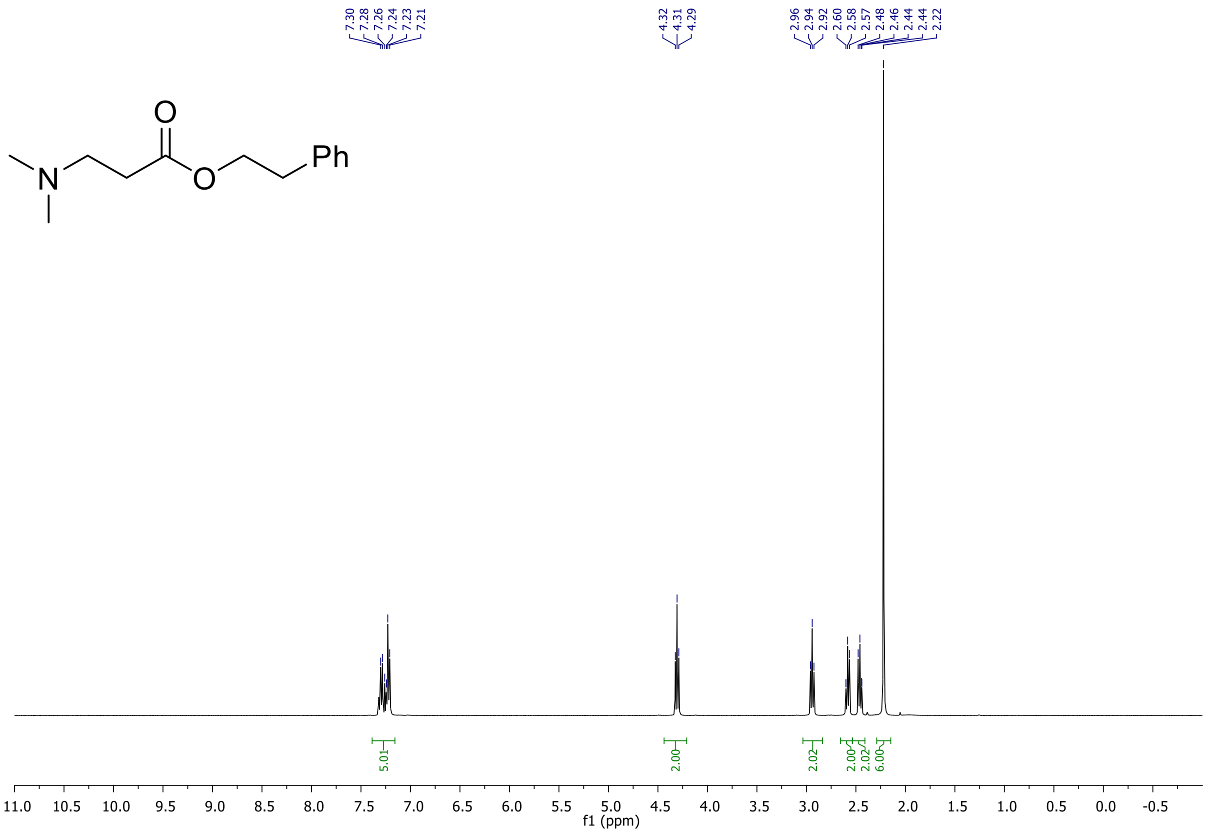

^{13}C NMR (101 MHz, $CDCl_3$)

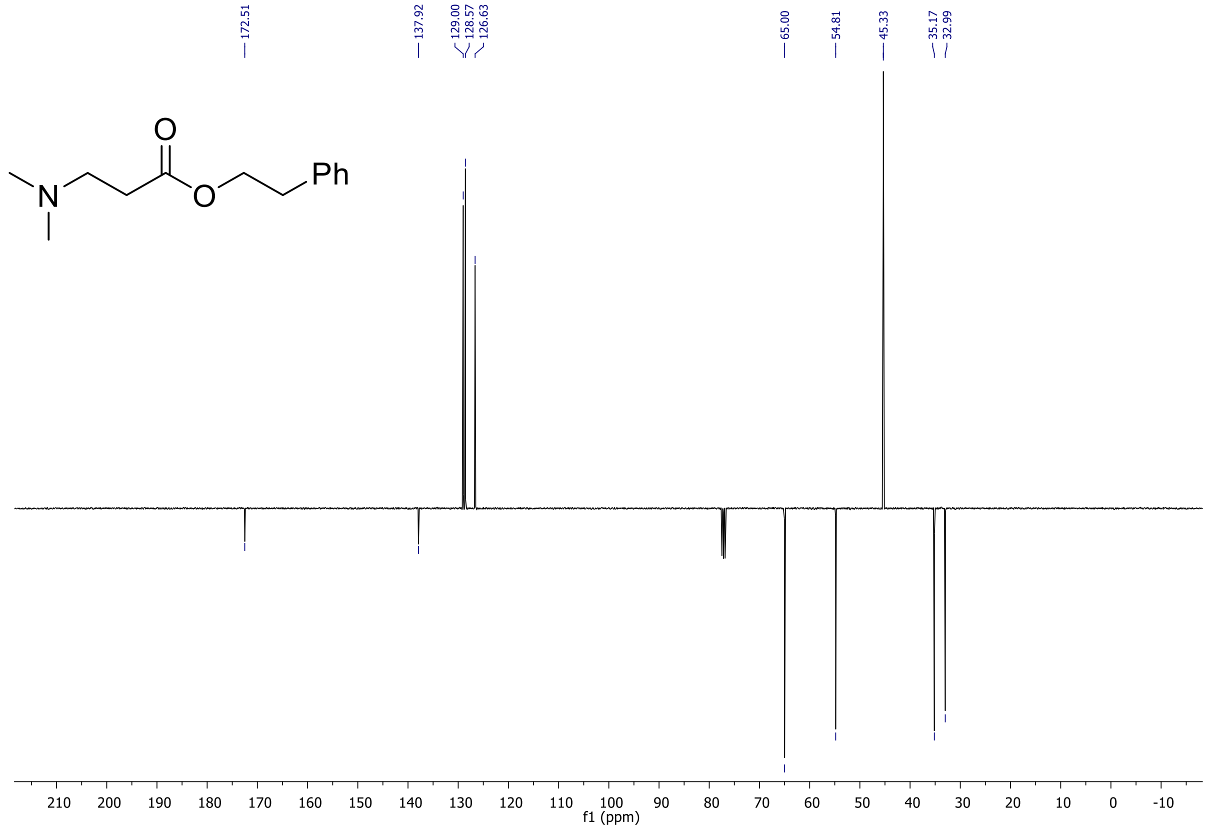

S1: Phenethyl 2-(hydroxymethyl)acrylate

^{1}H NMR (700 MHz, $CDCl_3$)

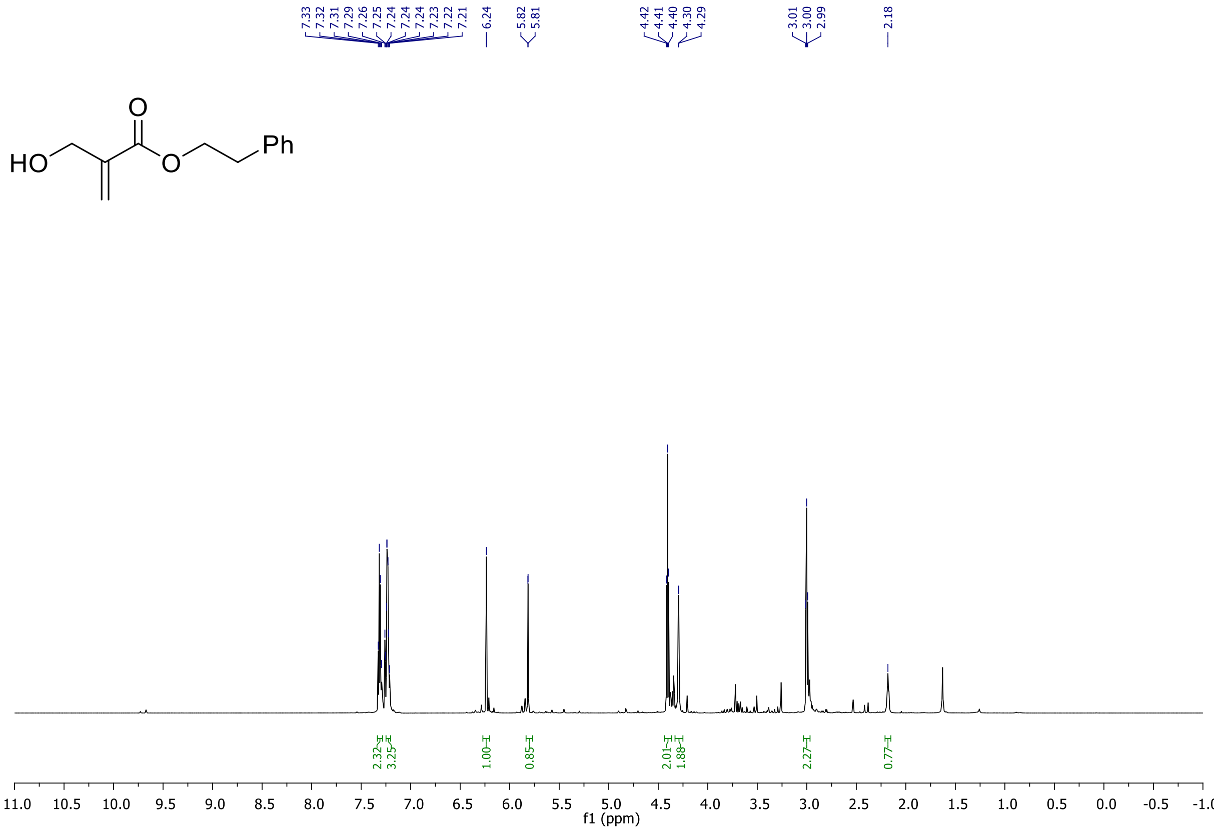

^{13}C NMR (176 MHz, $CDCl_3$)

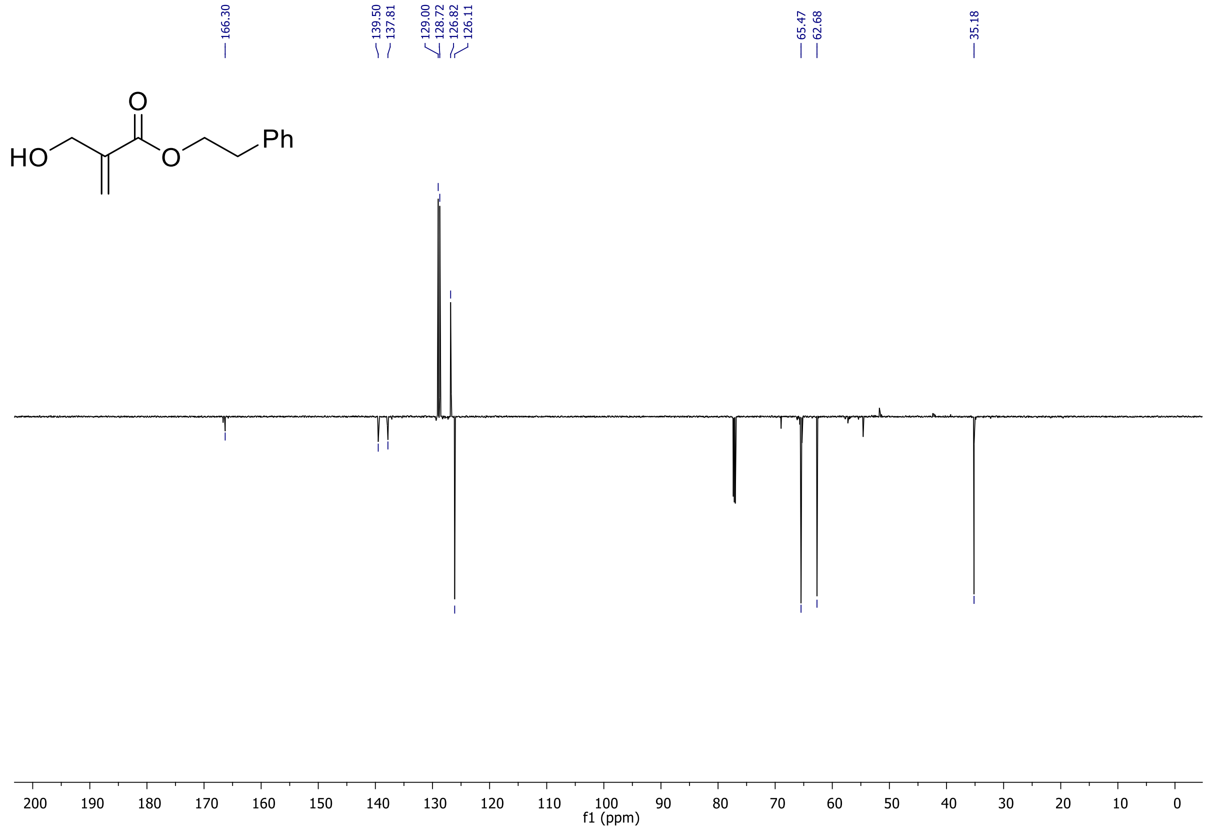

S2: Phenethyl 2-(acetoxymethyl)acrylate

^{1}H NMR (600 MHz, $CDCl_3$)

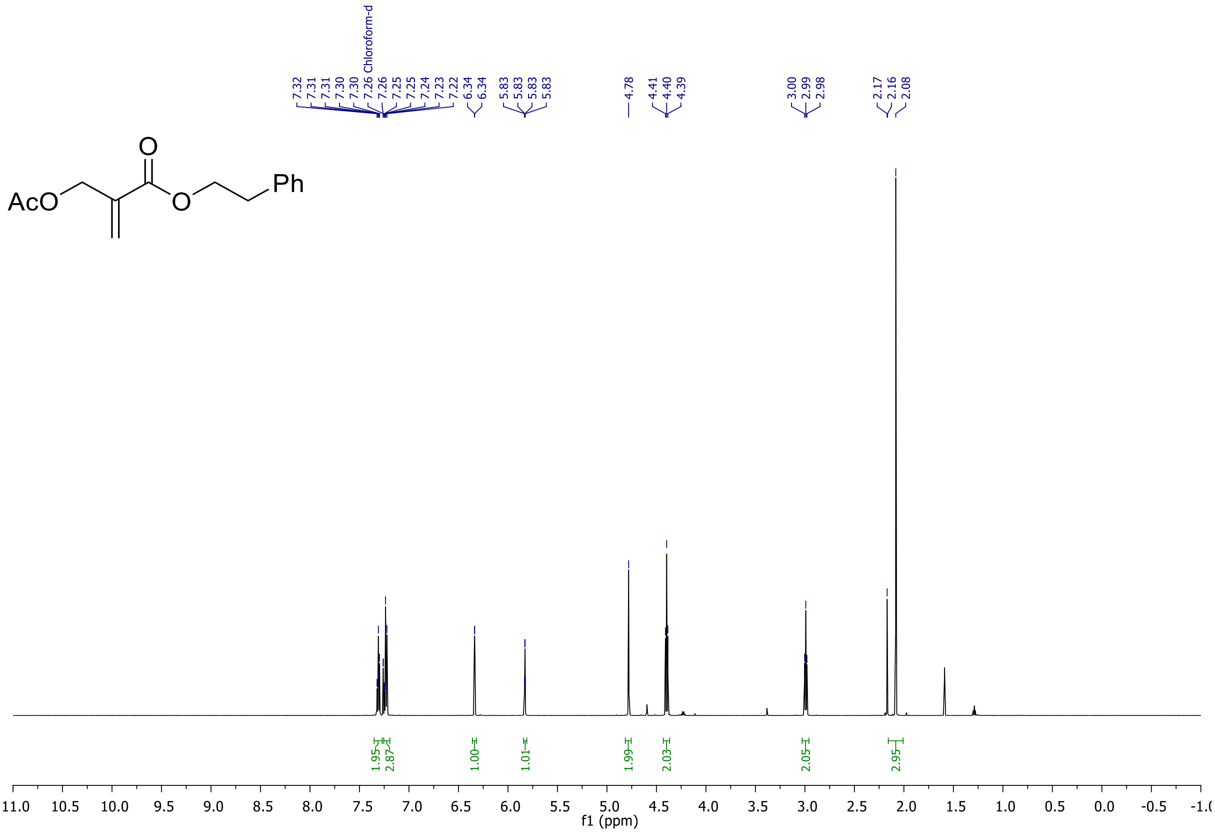

^{13}C NMR (151 MHz, $CDCl_3$)

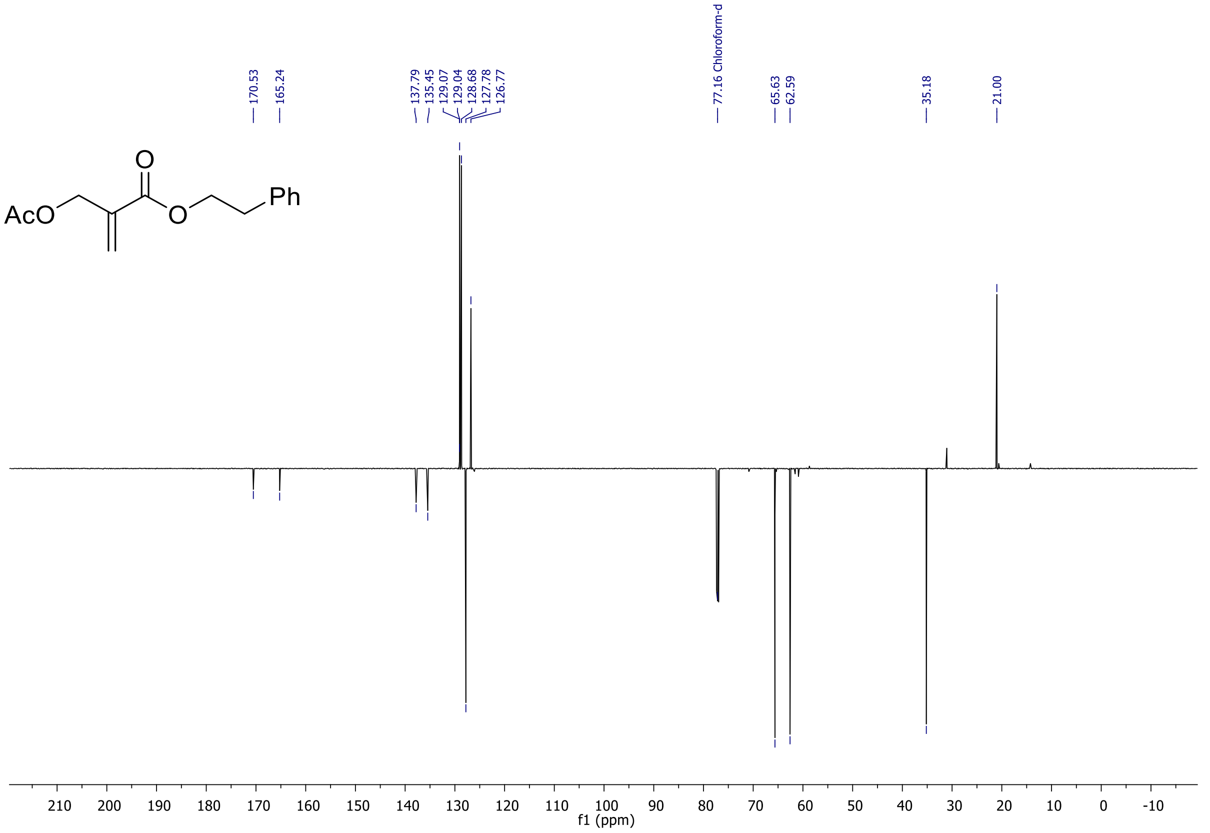

S3: Phenethyl 3-(pyrrolidin-1-yl)propanoate

^{1}H NMR (600 MHz, $CDCl_3$)

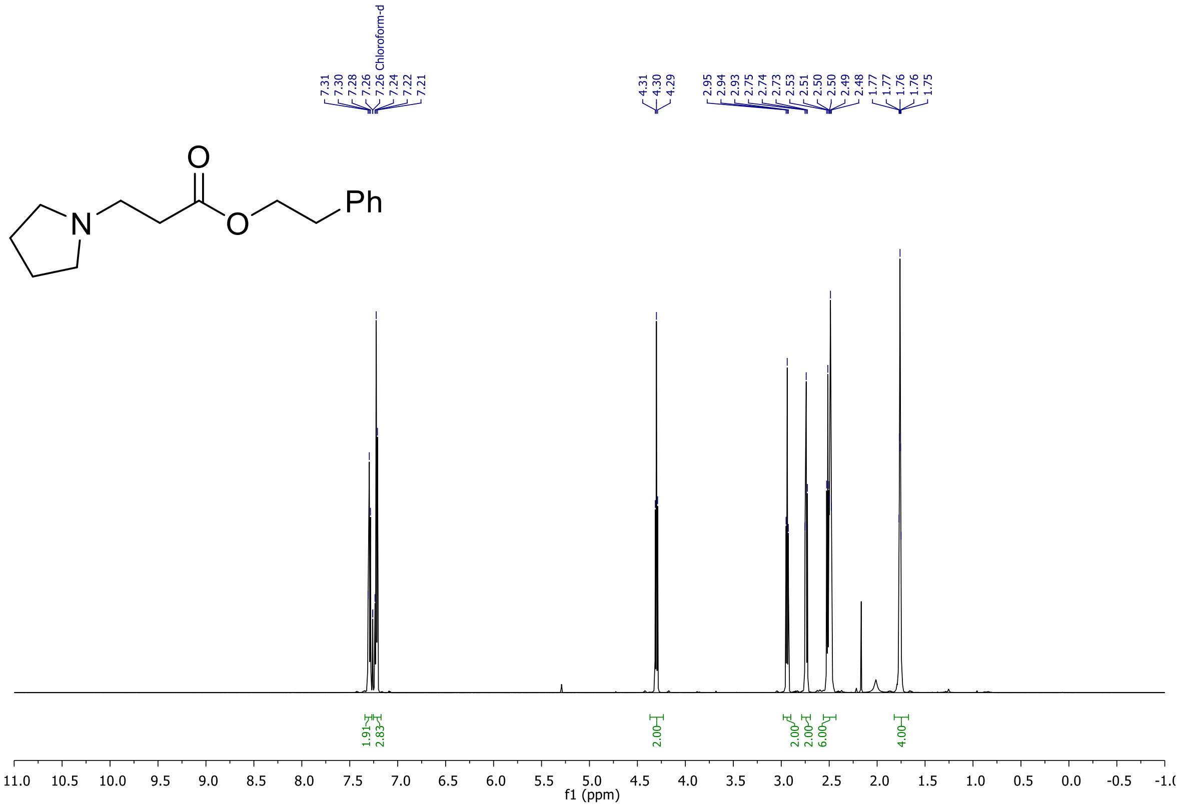

^{13}C NMR (151 MHz, $CDCl_3$)

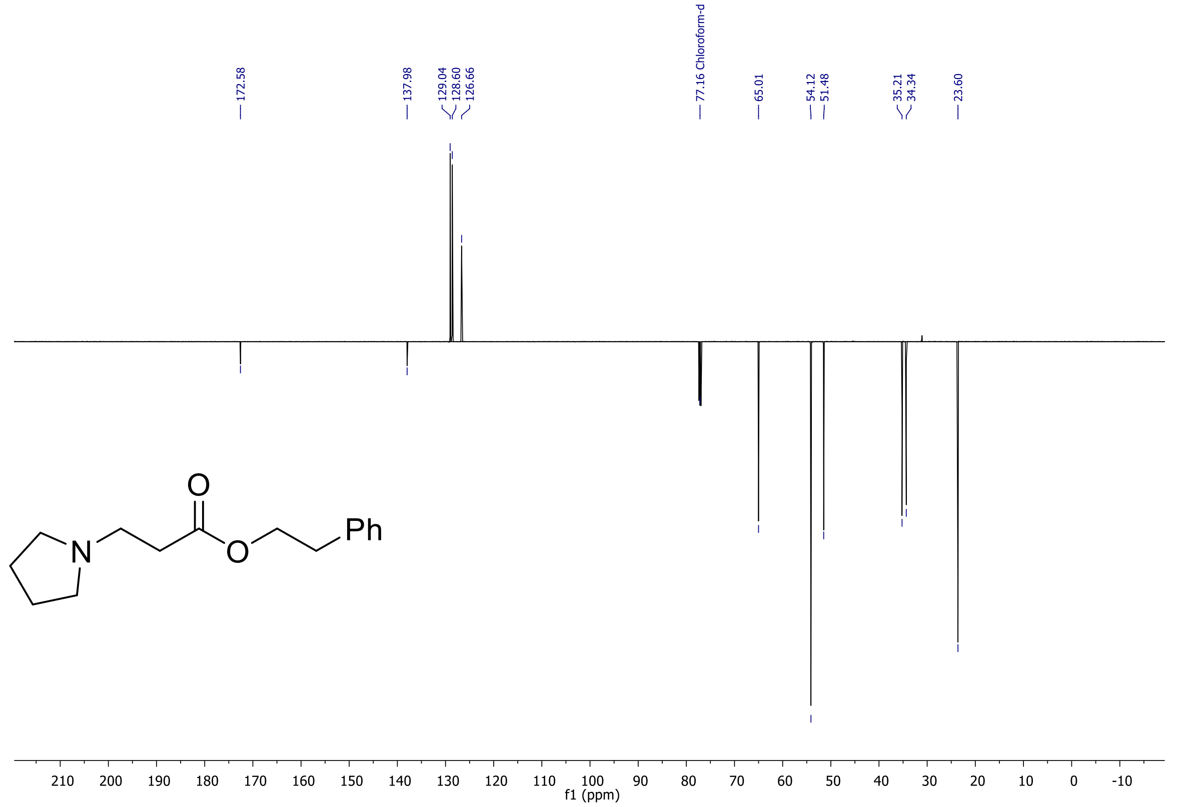

5: Phenethyl (*Z*)-2-((dimethylamino)methyl)-3-(4-nitrophenyl)acrylate

^{1}H NMR (700 MHz, $CDCl_3$)

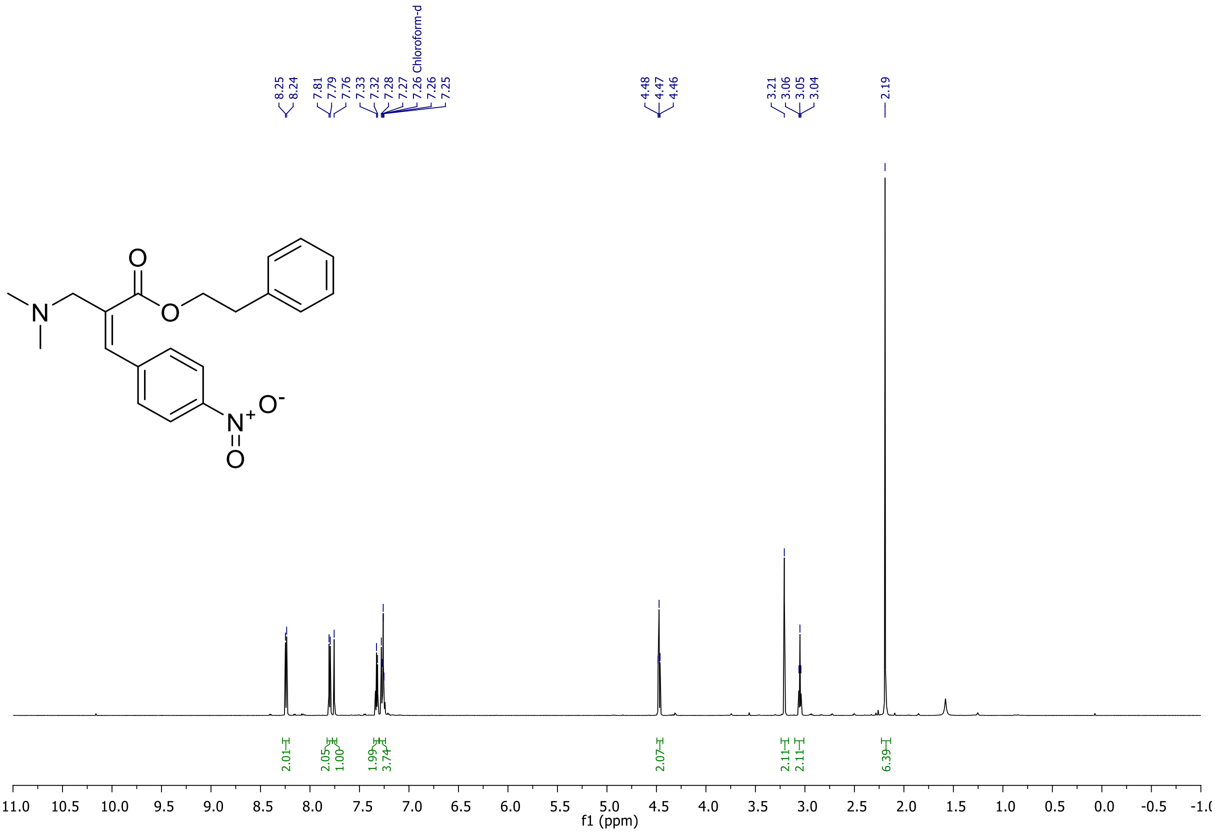

^{13}C NMR (176 MHz, $CDCl_3$)

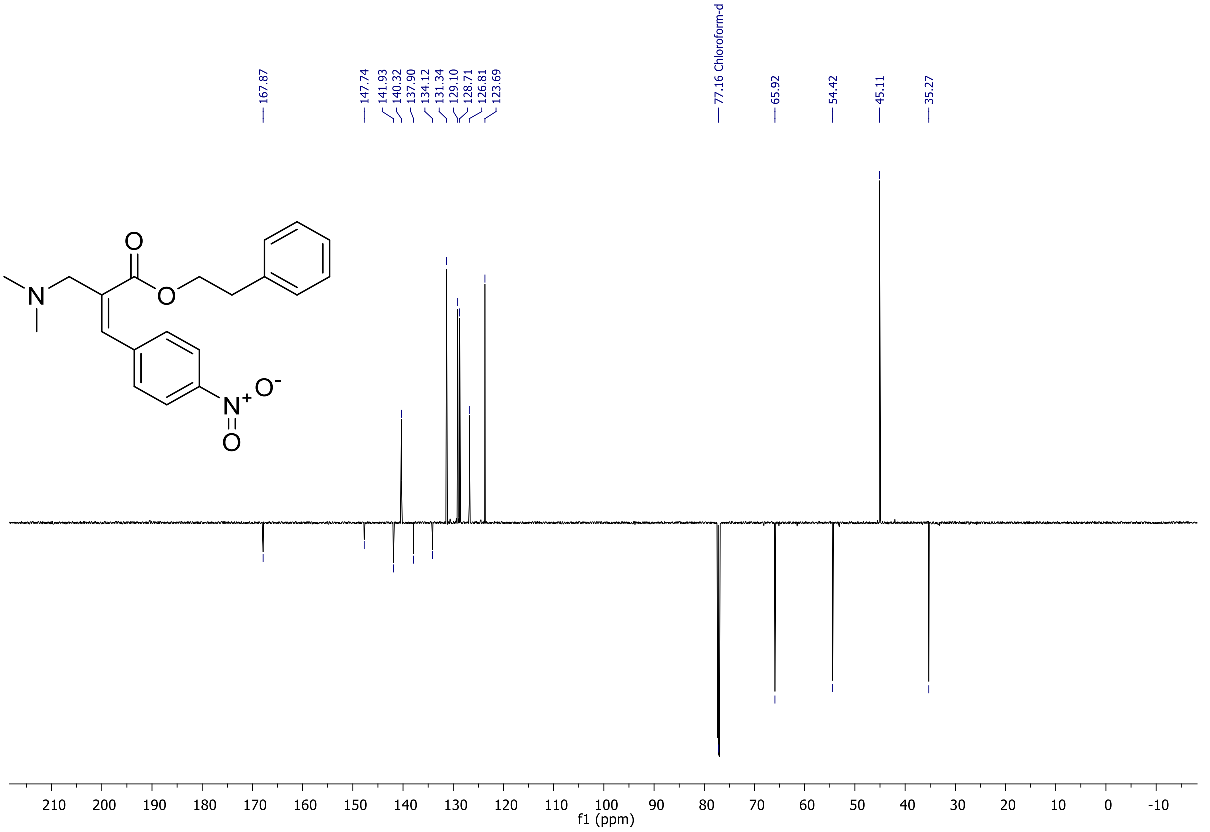

Supplement: Supplementary file 4 — Supporting Information [file ANIE-61-0-s005.pdf]
